# The effectiveness of varenicline versus nicotine replacement therapy on long-term smoking cessation in primary care: a prospective cohort study of electronic medical records

**DOI:** 10.1093/ije/dyx109

**Published:** 2017-06-26

**Authors:** Gemma MJ Taylor, Amy E Taylor, Kyla H Thomas, Timothy Jones, Richard M Martin, Marcus R Munafò, Frank Windmeijer, Neil M Davies

**Affiliations:** dyx109-1 Medical Research Council Integrative Epidemiology Unit,; dyx109-2 School of Social and Community Medicine, Barley House,; dyx109-3 UK Centre for Tobacco and Alcohol Studies,; dyx109-4 School of Social and Community Medicine, Canynge Hall, University of Bristol, Bristol, UK,; dyx109-5 National Institute for Health Research Collaboration for Leadership in Applied Health Research and Care West (NIHR CLAHRC West) at University Hospitals Bristol NHS Foundation Trust, Bristol, UK and; dyx109-6 Department of Economics, University of Bristol, Bristol, UK

**Keywords:** Smoking cessation, tobacco, varenicline, nicotine replacement therapy, effectiveness, primary care, causal, instrumental variable, cohort, electronic medical records

## Abstract

**Background:**

There is limited evidence about the effectiveness of varenicline and nicotine replacement therapy (NRT) for long-term smoking cessation in primary care, or whether the treatment effectiveness differs by socioeconomic position (SEP). Therefore, we estimated the long-term effectiveness of varenicline versus NRT (> 2 years) on smoking cessation, and investigated whether effectiveness differs by SEP.

**Methods:**

This is a prospective cohort study of electronic medical records from 654 general practices in England, within the Clinical Practice Research Datalink, using three different analytical methods: multivariable logistic regression, propensity score matching and instrumental variable analyses. Exposure was prescription of varenicline versus NRT, and the primary outcome was smoking cessation at 2 years’ follow-up; outcome was also assessed at 3, 6, and 9 months, and at 1 and 4 years after exposure. SEP was defined using the Index of Multiple Deprivation.

**Results:**

At 2 years, 28.8% (N = 20 362/70 610) of participants prescribed varenicline and 24.3% (N = 36 268/149 526) of those prescribed NRT quit; adjusted odds ratio was 1.26 [95% confidence interval (CI): 1.23 to 1.29], P < 0.0001. The association persisted for up to 4 years and was consistent across all analyses. We found little evidence that the effectiveness of varenicline differed greatly by SEP. However, patients from areas of higher deprivation were less likely to be prescribed varenicline; adjusted odds ratio was 0.91 (95% CI: 0.90 to 0.92), P < 0.0001.

**Conclusions:**

Patients prescribed varenicline were more likely to be abstinent up to 4 years after first prescription than those prescribed NRT. In combination with other evidence, the results from this study may be used to update clinical guidelines on the use of varenicline for smoking cessation.

**Key Messages**

- This is the largest study to date investigating the effectiveness of varenicline versus NRT for smoking cessation in primary care settings.
- Varenicline is more effective than NRT for smoking cessation up for to 4 years in primary care settings.
- Varenicline’s effectiveness does not appear to be altered by socioeconomic position.

## Introduction

Tobacco is the world’s leading preventable cause of serious illness and premature death.^1^ One in two smokers will die from their addiction unless they stop smoking.^2^ To date, there are only three full-scale randomized controlled trials (RCTs) which have compared the effects of varenicline and nicotine replacement therapy (NRT) on smoking cessation.^3–6^ Baker and colleagues found that varenicline had similar effects as did NRT on smoking abstinence at 26 weeks; the odds ratio was 1.3 (95% confidence interval, 0.9 to 1.9).^3^ Aubin and colleagues also reported similar effects between the two medicines at 12 months [1.4 (95% confidence interval 0.99 to 1.99)].^4^ In contrast, Anthenelli and colleagues (2016) reported that those treated with varenicline achieved higher rates of abstinence compared with NRT at 24 weeks; odds ratio (and 95% confidence interval) were 1.5 (1.3 to 1.8).^5^ Cahill and colleagues conducted a network meta-analysis of RCTs which suggested that varenicline is the most efficacious smoking cessation medicine at up to 12 months; odds ratio (and 95% confidence interval) were 1.6 (1.3 to 1.9).^7^ However, the efficacy of treatments in trial settings may differ from their effectiveness in everyday clinical settings because of variation in treatment delivery and participant characteristics. Moreover, abstinence at 6 to 12 months does not necessarily guarantee longer-term abstinence (> 24 months). A systematic review of RCTs found that 30% of participants recorded as quitting at 12 month follow-up relapsed in subsequent years.^8^

Furthermore, we do not know whether the effectiveness of smoking cessation medications differs by socioeconomic position (SEP). Smoking is a major contributor to health inequalities between the richest and poorest in society.^9^^,^^10^ There is evidence that smokers in more deprived areas in the UK are more likely to receive advice to quit from their general practitioner.^11^ Nevertheless, observational studies have found that smokers from disadvantaged backgrounds are much less likely quit,^10^ even after accessing treatment from specialist stop-smoking services.^12^ However, there is little evidence from RCTs about whether the effectiveness of smoking cessation medications differs by SEP, and trials are typically underpowered to detect treatment effect heterogeneity.

In this study we aimed to: (i) estimate the long-term effectiveness of prescribing varenicline versus NRT on smoking cessation in primary care; and (ii) examine whether the effectiveness differed by SEP.

## Methods

We conducted a prospective cohort study using electronic medical records from 654 general practices in England. This research was conducted according to the principles of the Declaration of Helsinki and we followed STROBE reporting guidelines.^13^ The study protocol was published in advance^14^ and is available via the Open Science Framework (https://osf.io/g9ch2/) and ClinicalTrials.gov (ID:NCT02681848). It was approved by the Independent Scientific Advisory Committee (ISAC) for MHRA Database Research (https://www.cprd.com/isac/) (protocol number: 15_107R).

### Data source and population

We obtained data from the Clinical Practice Research Datalink (CPRD) GOLD [www.cprd.com], a medical database containing data on over 13 million patients across the UK. Registered patients are representative of the UK’s demography.^15^

### Code lists

We defined variables using medical and product codes within the CPRD. Validated lists were used where available, and where unavailable, code lists were agreed upon by field experts (R.M.M., D.R., K.H.T.) and by using the British National Formulary and the International Classification of Diseases. Code lists are available at [https://github.com/nmdavies/varenicline effectiveness/].

### Patients

Included patients were aged 18 years and over, and were prescribed NRT or varenicline. We included patients with no breaks in their records, with complete information on year of birth, registration date and sex; and patients from practices with continuous recording of data. We excluded patients who registered with their practice within 365 days of their first recorded prescription, to ensure availability of baseline data and data to define the first prescription of smoking cessation medication.

### Variables

#### Exposure

Treatment was defined as prescription of varenicline, and control as prescription of NRT (e.g. patches, gum, lozenges, sprays and inhalers). Prescriptions used to define treatment groups occurred after 1 September 1 2006 until 30 September 30 2015, with no previous evidence of use of a related product during 18 months before the first prescription was issued. We used the first treatment episode^16^ to ensure that exposure groups were ‘new users’ of the medication and time of treatment allocation was similar to baseline in a clinical trial, which is the time of randomization.^17^ We did not model treatment switching because this is likely to be strongly related to patient characteristics.

#### Outcome

The primary outcome was having an electronic medical record indicating smoking at 2-year follow-up. Smoking status was also assessed at 3, 6 and 9 months, and at 1 and 4 years after the first prescription. GPs recorded their patients’ smoking status as current, former or never smoker in their electronic medical records; these data were repeatedly recorded over time as part of a UK nationwide incentive programme,^18^ and these smoking records are highly comparable to smoking prevalence as reported in representative population surveys.^19^ We determined each patient’s smoking status by using their most recent smoking record identified between cohort entry and each follow-up period (e.g. 3 months, 6 months, 1 year). In our primary analysis, patients with missing smoking data were assumed to be continuing smokers.^20^ For statistical analyses, smoking status was defined as smoker (0) or quit (1).

#### Covariates

Covariates included patients’ age at time of prescription, sex, days registered in the CPRD, mental health history (bipolar, depression, neurotic, anxiety disorders, self-harm or other mental health disorders), previous use of psychotropic medications (antidepressants, antipsychotics, hypnotics/anxiolytics or other psychotropic medications), drug or alcohol misuse,^21^ mean number of GP visits 1 year preceding first prescription, body mass index (BMI), SEP and major chronic illness (Charlson Index^22^). SEP was recorded based on patient postcode at the lower-layer super-output area level, and measured using the index of multiple deprivation (IMD) which is the official measure of deprivation in England; and was recorded using twentiles (i.e. 1 = lowest level of deprivation, 20 = highest level).

### Follow up

Patients were followed up at 3, 6, and 9 months and 1, 2 and 4 years after exposure.

### Statistical analysis

Analyses were conducted using Stata 14. All scripts are available online [https://github.com/nmdavies/varenicline effectiveness/]. To investigate the effects of varenicline versus NRT on smoking cessation, we conducted a multivariable adjusted logistic regression. Models were estimated using cluster robust standard errors, which accounted for potential clustering of patients between physicians. Differences in the effectiveness of varenicline by SEP were investigated by stratifying patients on level of deprivation non-imputed data, low deprivation as indicated by an IMD rank of 1 to 10, and high deprivation as indicated by an IMD score of 11 to 20, and conducting partially adjusted logistic regression models for age, sex and year of smoking cessation medication prescription (see Supplementary Methods for further details, available as Supplementary data at *IJE* online).

To address potential residual confounding and selection bias (i.e. varenicline users may have previously had a failed a quit attempt using NRT, and thus are more likely to have been prescribed varenicline), we repeated all analyses using: propensity score matched logistic regressions; and instrumental variable regressions using physicians’ prescribing preferences as the instrument.^23^ Instrumental variable analysis uses variables which are: related to the exposure, independent of confounders, and have no direct effects on the outcome.^24^ If the multivariable adjusted regression results suffer from residual confounding, they will differ compared with results from the instrumental variable models (see Supplementary material for further details of the methods, available as Supplementary data at *IJE* online). We examined the extent of confounding variables across all three analysis types (e.g. association between the exposure or instrument, and baseline covariates).^25–28^

### Missing baseline covariate data

To increase efficiency and minimize selection bias, we used multivariable multiple imputation to impute data for patients missing BMI and IMD values.^29^ The imputation procedure produced 20 imputed datasets, and the imputation model included all exposures and covariates.^30^

### Sensitivity analysis: missing outcome data

It was possible that our ascertainment of outcome, i.e. in which participants with missing smoking status medical records were classed as continuing smokers,^20^ might lead to misclassification bias. To examine this possibility we conducted a sensitivity analysis in which we imputed missing outcome data using multivariate multiple imputation. The imputation procedure produced 20 imputed datasets, and the imputation model included all exposures and covariates.^30^ We compared the effect estimates derived from the sensitivity analysis (missing outcome data = imputed) with those derived from the main analysis (missing outcome data = continuing smoker).

### Comparison with other studies

We used a random effects meta-analysis to compare our multivariable logistic regression estimate with estimates reported by the systematic review and those derived from subsequently published RCTs of varenicline versus NRT.^3–5^^,^^7^

## Results

### Population characteristics

A total of 287 079 patients were prescribed smoking cessation medications during the study period. Of these, 149 526 patients prescribed NRT and 70 610 patients prescribed varenicline were eligible for analysis. Supplementary Figure 1 (available as Supplementary data at *IJE* online) presents the number of patients excluded and reasons for exclusion.^14^ Of those prescribed NRT, a range of products was prescribed including patches, gum, oral spray, nasal spray, oral film, inhaler, lozenges and microtab (see Supplementary Table 1, available as Supplementary data at *IJE* online, for list of NRT products prescribed); 34 396 (23%) of the patients prescribed NRT were prescribed more than one nicotine product. On average, patients who were prescribed varenicline were issued 2.8 [standard deviation (SD) = 1.6] prescriptions for varenicline in the 3 months following their first eligible prescription, and received an average of 107.5 (SD = 120.7) tablets; 49.7% (*N* = 35 076/70 610) of these patients received a full course of varenicline (i.e. ≥ 12 weeks) (Chapter 4, British National Formulary). Patients who were initially prescribed NRT were issued 2.5 (SD = 2.2) prescriptions for NRT on average during the 3 months after their first eligible prescription. At the time of prescription, patients’ mean age was 45.8 years (SD = 14.9); 52.6% of the cohort were women. Baseline data indicated that this cohort was similar to other studies of smokers from the UK and other developed nations.^21^ The median patient had an IMD score of 12, indicating that they lived in the 60–65% most deprived areas in England (Table 1); 35.8% of patients showed evidence of a major comorbidity.^22^ The number of patients with mental health morbidities or prescribed psychotropic medications was consistent with the prevalence of mental illness found in cohorts of smokers.^31^ Patients prescribed NRT were more likely to be older and to have a history of comorbidities.

**Table 1:** Baseline characteristics of cohort and by exposure group. Data are the number (%) of patients unless otherwise specified

Characteristic	NRT (*N* = 149526)	Varenicline (*N* = 70610)	Whole sample (*N* = 220136)
Age at time of first prescription^a^	46.4 (15.4)	44.5 (13.2)	45.8 (14.9)
Sex (female)	53.7% (80 348)	50.2% (35 466)	52.6% (115 814)
Index of multiple deprivation score (IMD)^†^^b^	12	12	12
Mean number of GP visits 1 year before first prescription^a^	7.9 (7.4)	6.3 (6.1)	7.4 (7.0)
BMI^†a^	26.4 (6.4)	26.5 (5.9)	26.4 (6.1)
Year of first prescription^b^	2009	2010	2009
Days of history^a^	3158.7 (1892.1)	3283.9 (1976.6)	3198.9 (1920.5)
Comorbidity ever (Charlson Index^22^)	37.6% (56 274)	31.9% (22 523)	35.8% (78 797)
Alcohol misuse ever	8.3% (12 422)	6.0 (4 199)	7.6% (16 621)
Drug misuse ever	3.1% (4 595)	1.9% (1 357)	2.7% (5 952)
Bipolar ever	1% (1 464)	< 1% (160)	< 1% (1 624)
Depression ever	35.0% (52 233)	29.2% (20 615)	33.1% (72 848)
Neurotic disorder ever	24.7% (36 921)	20.1% (14 189)	23.2% (51 110)
Self-harm ever	10.6% (15 903)	8.7% (6 169)	10.0% (22 072)
Other rare mental disorder ever	6.9% (10 343)	4.0% (2 832)	6.0% (13 175)
Antidepressant prescription ever	50.1% (74 921)	43.1% (30 435)	47.9% (105 356)
Antipsychotic prescription ever	20.0% (29 873)	14.8% (10 459)	18.3% (40 332)
Hypnotics/anxiolytics prescription ever	21.1% (31 513)	17.6% (12 415)	20.0% (43 928)
Other psychotropic medication	< 1% (473)	< 1% (120)	< 1% (593)

Missing BMI and IMD values were imputed using multiple imputation.^29^ See Supplementary material for comparison of imputed and raw data, available as Supplementary data at *IJE* online (Supplementary Table 2).

^†^Missing data: BMI data were missing for 14.2% (*N* = 31169); IMD data were missing for 43.3% (*N* = 95 355). ^a^Data presented are mean and standard deviation.

^b^Data presented are median.

### The association of varenicline or NRT prescriptions and smoking cessation

**Figure 1:**
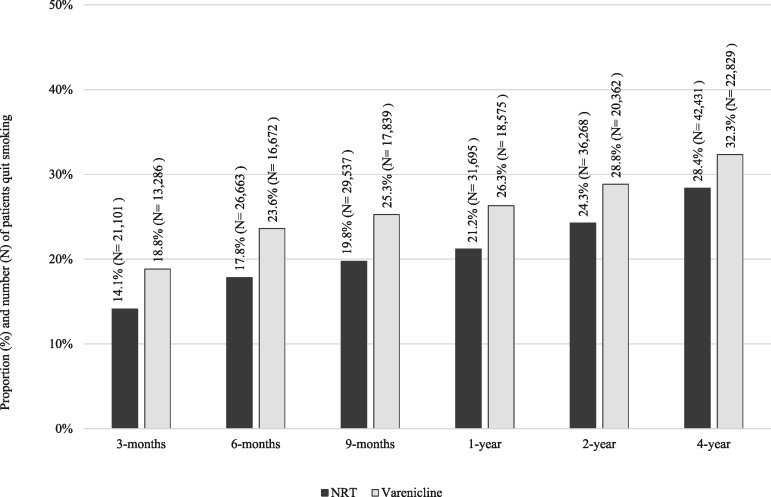
Absolute quit rates by treatment group at 3, 6 and 9 months and 1, 2 and 4 years after exposure, *N* = 220 136.

**Figure 2:**
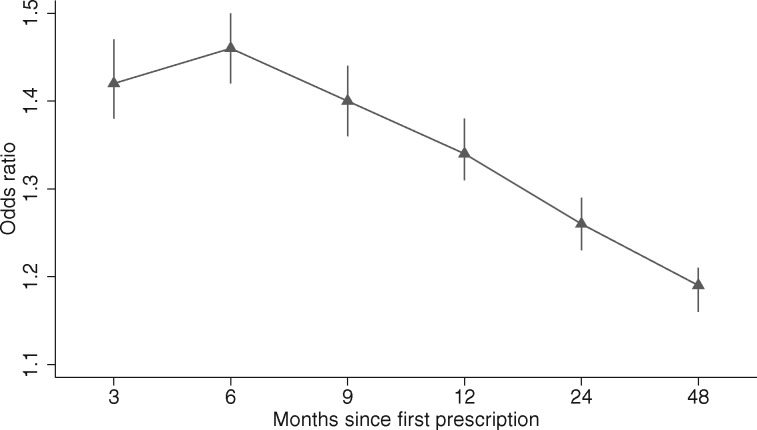
The association of prescribing varenicline and smoking cessation at 3,6 and 9 months and 1, 2 and 4 years after first prescription. Fully multivariable adjusted logistic regression model: odds ratio and 95% confidence intervals presented. The difference in smoking cessation rates peaks at 6 months and declines over the following 3.5 years, *N* = 220 136.

Patients prescribed varenicline were more likely to quit smoking than those prescribed NRT, at all follow-ups (Figure 1). Partially and fully adjusted multivariable regression models indicated that varenicline was associated with increased odds of quitting smoking at all follow-ups, and the association attenuated slightly at 4-year follow-up; however, the direction and precision of the association remained consistent over time (Figure 2). Table 2 presents fully and partially adjusted odds ratios and 95% confidence intervals for the association, by prescription and follow-up. At 2 years, 28.8% (*N* = 20 362/70 610) of participants prescribed varenicline quit, and 24.3% (*N* = 36 268/149 526) of those prescribed NRT quit; the fully adjusted odds ratio and 95% confidence interval at 2-year follow-up were 1.26 (1.23 to 1.29), *P* < 0.001.

**Table 2:** Multivariable logistic regression models: partial and fully adjusted odds ratios and 95% confidence intervals for the association between varenicline versus NRT and smoking cessation at 3, 6 and 9 months and 1, 2 and 4 years after exposure, *N* = 220136^a^

Model	3 months	6 months	9 months	1 year	2 years	4 years
	
	Odds ratio (95% confidence interval)					
Partial adjusted^b^	1.47	1.50	1.44	1.38	1.30	1.23
	(1.42 to 1.52)	(1.46 to 1.55)	(1.40 to 1.48)	(1.35 to 1.42)	(1.27 to 1.33)	(1.21 to 1.26)
Fully adjusted^c^	1.42	1.46	1.40	1.34	1.26	1.19
	(1.38 to 1.47)	(1.42 to 1.50)	(1.36 to 1.44)	(1.31 to 1.38)	(1.23 to 1.29)	(1.16 to 1.21)

^a^Missing BMI and IMD values were imputed using multiple imputation.^29^

^b^Partial adjusted models were adjusted for: age, sex and year of prescription.

^c^Fully adjusted models were adjusted for: age, sex, days in history, IMD, number of GP visits 1 year preceding first prescription, BMI, year of first prescription, history of major physical morbidity (Charlson Index), alcohol misuse, drug misuse, bipolar, depression, neurotic disorder, self-harm, other mental disorder, antidepressant prescription ever, antipsychotic prescription ever, hypnotics/anxiolytics prescription ever, other psychotropic medication.

The propensity score balanced the treatment groups’ baseline covariates (see Supplementary Figures 2 and 3 for bias assessment, available as Supplementary data at *IJE* online), and in the instrumental variable model we found that the instrument was more weakly associated with the covariates than the patients’ actual prescription (see Supplementary Figure 4, available as Supplementary data at *IJE* online, for bias assessment). Furthermore, the propensity score matched and instrumental variable models estimates were entirely consistent with the fully adjusted logistic regression estimates (see Supplementary Tables 3 and 4 and Supplementary Figure 5, available as Supplementary data at *IJE* online).

### Differences in the effects of varenicline by SEP

In our sample, patients from the most deprived areas were less likely to be prescribed varenicline compared with those from the least deprived areas [age- and sex-adjusted odds ratio 0.91 (95% confidence interval: 0.90 to 0.92), *P* < 0.0001 (Supplementary Figure 6, available as Supplementary data at *IJE* online]. Varenicline was slightly more effective in patients from the least deprived areas at 3 months to 1 year after first prescription, but this difference attenuated by the 2 and 4 years’ follow-up, as shown in

**Figure 3:**
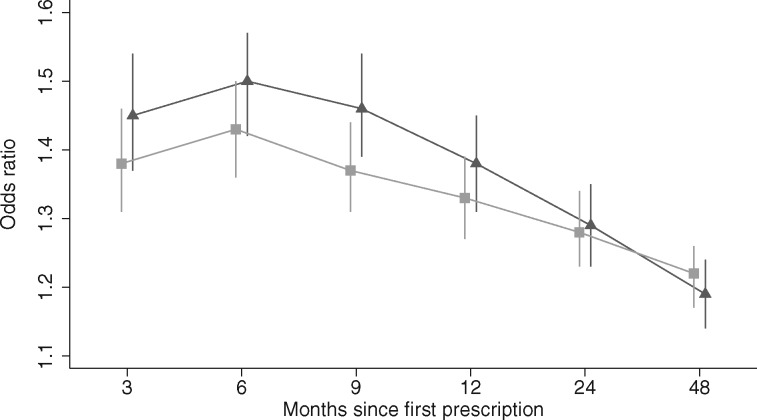
The effectiveness of varenicline stratified by socioeconomic position. Partial adjusted odds ratios and 95% confidence intervals for the association of prescription of varenicline versus NRT and smoking cessation at 3, 6 and 9 months and 1, 2 and 4 years after exposure, by level of deprivation as measured by the Index of Multiple Deprivation Score (IMD). IMD is an increasing measure of neighbourhood disadvantage; models were adjusted for age, sex and year of first prescription. Missing IMD values were not imputed and patients with missing IMD data were excluded from all analyses, to ensure comparability of results across samples. Legend: □ Patients from least deprived areas (IMD scores 1 to 10), *N* = 52 534. ▴ Patients from most deprived areas (IMD scores 11 to 20), *N* = 72 247.

Figure 3 and Supplementary Tables 5 and 6 (available as Supplementary data at *IJE* online).

### Sensitivity analyses

The proportions of missing data were similar between exposure groups at all follow-ups (Supplementary Table 7, available as Supplementary data at *IJE* online). At 2 year’s follow-up, 20.3% (44 737/220 136) of patients were missing smoking status data; these patients were on average, younger, male, visited the GP fewer times per year and had fewer comorbidities (Supplementary Table 8, available as Supplementary data at *IJE* online). Effect estimates derived from the main analysis (missing outcome data = continuing smoker) were similar to estimates derived from models in which missing outcome data were imputed (Supplementary Table 9 and Supplementary Figure 7, available as Supplementary data at *IJE* online).

### Comparison with other studies

**Figure 4:**
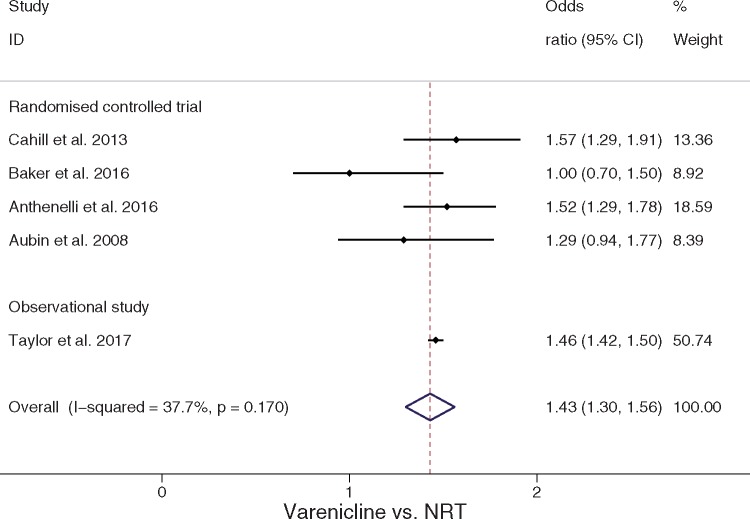
Random effects meta-analysis comparing effect estimates (odds ratio and 95% confidence interval) from existing studies examining the effect of varenicline versus NRT for smoking cessation at 6–12 months’ follow–up.

Our meta-analysis indicated that 6-month effectiveness estimates derived from this study were similar to effect estimates derived from full-scale RCTs and a network meta-analysis, as indicated by heterogeneity statistics (Q = 6.42, *P* = 0.170, I^2 ^= 37.7%, Tau2 = 0.0084).^32^^,^^33^Figure 4 indicates that on average varenicline was associated with higher abstinence rates compared with NRT [odds ratio 1.4 (95% confidence interval: 1.3 to 1.6] (Figure 4).

## Discussion

### Main findings

To date there are only three full-sized RCTs testing the relative efficacy of NRT and varenicline,^3–5^ two of which were open label,^3^^,^^4^ and all of which were limited to 6 to 12 months’ follow-up. These trials tell us little about the longer-term differences in abstinence rates caused by these medications.^8^ Second, there is little evidence about whether varenicline is effective in disadvantaged populations. This is the largest study of the effectiveness of varenicline versus NRT for enduring smoking cessation in primary care settings.^34^^,^^35^ We found that patients prescribed varenicline were more likely to quit smoking compared with patients prescribed NRT. This difference persisted over time, lasting up to 4 years, and the results were consistent across three different analysis methods. There was little evidence that varenicline’s effectiveness differed by level of deprivation; however, patients from more disadvantaged areas were less likely to be prescribed varenicline. 

### Strengths and limitations

Data from the CPRD are representative of the UK population^36^ and are typical of smokers from other developed nations;^21^^,^^22^^,^^31^ therefore these findings are likely to be generalizable. Misclassification of outcome and exposure is a major source of bias in observational studies.^13^ In this study, the exposure, smoking cessation medication, was defined using pre-existing and peer reviewed code lists.^21^ The outcome, smoking status, was well reported within the CPRD^11^ and was defined using expert reviewed definitions. Nevertheless, it is possible that some patients’ smoking status was inconsistently recorded. We defined smoking status using each patient’s latest smoking record within each follow-up period. In our primary analysis, we classified smokers with missing records as continuing smokers. Our findings were similar in a sensitivity analysis in which we imputed the outcome using multiple imputation. Most covariate data were complete, and we used multivariable multiple imputation to impute missing values for the exceptions (IMD and BMI).^29^^,^^30^

Residual confounding is a major limitation of observational studies.^24^^,^^37^ A particular strength of this study was the use of three different analytical methods to estimate the effectiveness of varenicline. The propensity score balanced the treatment groups’ observed baseline characteristics, and produced similar findings to the multivariable adjusted regression. Our instrumental variable analyses used naturally occurring variation in the GPs’ prescribing which, if its assumptions hold, is robust to unmeasured residual confounding of the exposure-outcome relationship, including confounding by indication. For example, our instrumental variable analysis would not suffer from bias if GPs were more likely to offer varenicline to patients they believed were more likely to quit, or were more supportive to these patients during their quit attempt or if a patient prescribed varenicline had a failed quit attempt using NRT. The instrumental variable analysis provides an alternative source of evidence about the effects of varenicline, using observational data.^24^ The instrumental variable results were less precise, but were consistent with the multivariable adjusted regression results, and suggested that varenicline was more effective for smoking cessation.^24^

This study used prescriptions issued in primary care; therefore, we do not have any information on medication adherence, and patients may have taken over-the-counter stop-smoking medications. Few patients faced a difference in out-of-pocket costs between varenicline and NRT prescriptions; therefore this is unlikely to have affected adherence. Furthermore, other studies have found that users of NRT continue taking the medication less than half the time it is prescribed.^38^ This means that our results are estimates of the effects of prescribing smoking cessation medications, and may underestimate the effects of actually taking these medications. Nonetheless, the estimates presented in this study reflect the effects of prescribing stop-smoking medications allowing for real-world patient treatment adherence. Finally, the diagnostic categories used to define covariates may not have captured all patients with applicable diagnoses. However, where possible we used validated code lists.^39^

### Comparison with other studies

A systematic review and network meta-analysis (i.e. a meta-analysis technique conducted where there a very few direct comparisons of treatments) has determined the efficacy of varenicline versus NRT for smoking cessation.^7^ The review found that at 6 to 12 months’ follow-up, participants allocated to varenicline were more likely to quit compared with those allocated to NRT.^7^ In this study, we meta-analysed the effect estimates derived from the network meta-analysis reported by Cochrane,^7^ estimates from full-scale RCTs^3–5^^,^^7^ and those derived from our study. The met- analysis indicated that our findings were comparable to those derived from gold-standard RCTs.^32^^,^^33^

Evidence from The Health Improvement Network indicated that smokers in more deprived groups were more likely to receive advice to quit from their GP.^11^ However, observational studies have shown that smokers from disadvantaged backgrounds are much less likely to quit.^10^^,^^12^ Our study found that those from the most deprived areas were less likely to be prescribed varenicline; but that there was little evidence of clinically meaningful differences in the effect of varenicline by SEP.

### Conclusion and clinical implications

Patients prescribed varenicline were more likely to quit smoking compared with those prescribed NRT up to 4 years after prescription, when treated in primary care. The results from this study provide new evidence that varenicline is not only efficacious (as indicated by RCTs) but is effective in real-world clinical practice. Taken together, this evidence may be used to update clinical guidelines on the use of varenicline for smoking cessation.

## Supplementary Data

Supplementary data are available at *IJE* online.

## Funding

The MRC Integrative Epidemiology Unit at the University of Bristol is supported by the Medical Research Council and the University of Bristol [MC_UU_12013/6, MC_UU_12013/9]. The research described in this paper was funded by the Medical Research Council [MR/N01006X/1] and the National Institute for Health Research (NIHR) Health Technology Assessment (HTA) programme [project number 14/49/94]. A.E.T., M.R.M. and G.T. are members of the UK Centre for Tobacco and Alcohol Studies, a UKCRC Public Health Research: Centre of Excellence. Funding from the British Heart Foundation, Cancer Research UK, Economic and Social Research Council, Medical Research Council and the National Institute for Health Research, under the auspices of the UK Clinical Research Collaboration, is gratefully acknowledged. K.H.T. was funded by a Clinical Lectureship award from the National Institute for Health Research from March 2014 to October 2016. R.M.M. is supported by a Cancer Research UK programme grant [C18281/A19169] (the Integrative Cancer Epidemiology Programme). T.J. is supported by the National Institute for Health Research (NIHR) Collaboration for Leadership in Applied Health Research and Care (CLAHRC) West at University Hospitals Bristol NHS Foundation Trust. The funders of this research had no role in the study’s design, conduct or reporting.

## Supplementary Material

Supplementary Figures and Tables

Supplementary Data

Supplementary Data
