# Supplementary material for: The effectiveness of varenicline versus nicotine replacement therapy on long-term smoking cessation in primary care: a prospective cohort study of electronic medical records
Source: Int J Epidemiol. 2017 Jun 26;46(6):1948–57. doi: 10.1093/ije/dyx109 (PMC5837420; doi:10.1093/ije/dyx109)
Supplement: Supplementary Figures and Tables [file ije-2017-02-0136-file002_dyx109.docx]

# Supplementary Material: Methods and Results

Table of Contents

[Supplementary Material: Methods and Results 1](#_Toc473624436)

[Methods 5](#_Toc473624437)

[Multivariable logistic regression model 5](#_Toc473624438)

[Propensity score matched logistic regression model 5](#_Toc473624439)

[Instrumental variable analysis 6](#_Toc473624440)

[Cox proportional hazards model 6](#_Toc473624441)

[Results 7](#_Toc473624442)

[Flow chart 8](#_Toc473624443)

[List of NRT products 9](#_Toc473624444)

[Baseline characteristics: Comparison of raw and imputed data 12](#_Toc473624445)

[Propensity score matching 13](#_Toc473624446)

[Summary of results 13](#_Toc473624447)

[% bias difference and covariate balance check 13](#_Toc473624448)

[Distribution plots: Kernel density estimation of groups’ propensity scores before and after propensity score matching 15](#_Toc473624449)

[Propensity score matched logistic regression model for the association between prescription of varenicline versus NRT and smoking cessation at all follow ups 17](#_Toc473624450)

[Instrumental variable analysis 18](#_Toc473624451)

[Summary of results 18](#_Toc473624452)

[Relative bias of linear regression and instrumental variable methods 19](#_Toc473624453)

[Quit rates by instrumental variable condition at follow-ups. 21](#_Toc473624454)

[Instrumental variable and conventional regression models 22](#_Toc473624455)

[Socioeconomic descriptive data as measured by the Index of Multiple Deprivation (IMD) 24](#_Toc473624456)

[Effectiveness of varenicline stratified by neighbourhood depreviation 25](#_Toc473624457)

[Sensitivity analysis: Missing outcome data 28](#_Toc473624458)

[Cox proportional hazards model: Test of proportional-hazards assumption 33](#_Toc473624459)

[References 35](#_Toc473624460)

List of Appendix Tables

[Supplementary Material Table 1. List of NRT products prescribed in study cohort 9](#_Toc473624461)

[Supplementary Material Table 2. Comparison of imputed and non-imputed data for BMI and IMD 12](#_Toc473624462)

[Supplementary Material Table 3. Propensity score matched models: Odds-ratios and 95% confidence intervals for the association between prescription of varenicline versus NRT and smoking cessation at 3, 6 and 9-months and 1, 2 and 4-years after exposure, N=141,218* 17](#_Toc473624463)

[Supplementary Material Table 4. Conventional and instrumental variable linear regression models: Risk difference per 100 patients treated and 95% confidence intervals for the association between varenicline versus NRT and smoking cessation at 3, 6 and 9-months and 1, 2 and 4-years after exposure, N=216,022 23](#_Toc473624464)

[Supplementary Material Table 5. Effectiveness of varenicline at 3, 6 and 9-months, and 1, 2 and 4-years after first prescription in the least deprived areas (IMD scores 1 to 10). Effect estimates and 95% confidence intervals presented for each analytic technique. 26](#_Toc473624465)

[Supplementary Material Table 6. Effectiveness of varenicline at 3, 6 and 9-months, and 1, 2 and 4-years after first prescription in the most deprived areas (IMD scores 11 to 20). Effect estimates and 95% confidence intervals presented for each analytic technique.‡ 27](#_Toc473624466)

[Supplementary Material Table 7. Number (N) and % of patients missing outcome data by treatment at all follow-ups 28](#_Toc473624467)

[Supplementary Material Table 8. Comparison of baseline characteristics between the whole sample and patients missing 2-year outcome data 30](#_Toc473624468)

[Supplementary Material Table 9. Multivariable logistic regression models: Comparison of estimates derived from the main analysis and the sensitivity analysis. Fully adjusted odds-ratios and 95% confidence intervals for the association between varenicline versus NRT and smoking cessation at 3, 6 and 9-months and 1, 2 and 4-years after exposure, N=220,136 32](#_Toc473624469)

[Supplementary Material Table 10. Cox proportional hazards model: Test of proportional-hazards assumption 34](#_Toc473624470)

List of Appendix Figures

[Supplementary Material Figure 1. Flow chart of the number (N) of patients and records assessed for eligibility and reasons for exclusion 8](#_Toc473624471)

[Supplementary Material Figure 2. % bias difference between treatment and control groups, before and after propensity score matching 14](#_Toc473624472)

[Supplementary Material Figure 3. Kernel density estimation of groups’ propensity scores before and after matching 16](#_Toc473624473)

[Supplementary Material Figure 4. Relative bias of linear regression and instrumental variable methods: Binary and continuous outcomes 20](#_Toc473624474)

[Supplementary Material Figure 5. Point prevalence quit rates by instrumental variable condition at 3, 6 and 9-months and 1, 2 and 4-years after exposure, N=216,022* 21](#_Toc473624475)

[Supplementary Material Figure 6. Number (N), % and 95% confidence interval of patients prescribed smoking cessation medication by level of deprivation. Missing IMD data were not imputed. Quartiles are displayed for presentation purposes only. 24](#_Toc473624476)

[Supplementary Material Figure 7. Multivariable logistic regression model, in which missing outcome data were imputed using multivariate multiple imputation: Fully adjusted odds-ratios and 95% confidence intervals for the association between prescription of varenicline versus NRT and smoking cessation at follow-ups. The difference in smoking cessation rates peaks at 6-months and declines over the following 3.5 years. 31](#_Toc473624477)

# Methods

## Multivariable logistic regression model

We conducted a multivariable logistic regression model to estimate the association between prescription of varenicline versus NRT and smoking cessation at 3, 6 and 9-months and 1, 2 and 4-years after exposure. Models were partially adjusted for age, sex, year of first prescription, and repeated with full adjustment for all baseline covariates.

## Propensity score matched **logistic regression model**

Propensity score matching was used to create a sample of patients balanced on baseline covariates (1-4). Each participant’s propensity score was their conditional probability of receiving varenicline versus NRT based upon their baseline characteristics (odds-ratio). Developing the propensity score model involved two steps: First using the psmatch2 command in Stata 14 (1) a logistic regression model was constructed including all baseline covariates to calculate each participant’s propensity score. Each patient prescribed varenicline was matched to another patient prescribed NRT with the closest propensity score on a ratio of 1:1 using a nearest neighbor greedy algorithm, with no replacements, and matching was restricted to the common support region (5-7). A logistic regression model was conducted in the matched sample with adjustment for propensity score to examine the association between prescription of smoking cessation medication and smoking cessation at all follow-up periods. Reporting of propensity score matching results followed reporting guidelines suggested by Thoemmes and Kim, as were model adequacy checks for balance and propensity score distributions (8).

*Model adequacy checks:* The propensity score model was checked to ensure that a balance of means and variances was achieved for covariates after matching using the psttest command (1, 8). The standardized % bias before and after matching was calculated to assess % bias reduction. The standardized % bias is the % difference of the sample means in the NRT and varenicline groups (whole or matched sample), as a % of the square root of the average of the sample variances in each group (1).

Matching was restricted to within the common support region, which can be examined diagrammatically; plots of Kernel density estimations of groups’ propensity scores were plotted by group, before and after matching (8). If overlap between the groups’ propensity score distributions is broad, this allows for causal estimates over the full range of propensity scores in the sample (9). However, a small common support region restricts estimation of a causal effect and can result in bias by changing the observed population (9).

## Instrumental variable analysis

GPs prescribing preferences for issuing smoking cessation medication prescriptions were used as the instrument (i.e. a variable which is related to the exposure, but has no common cause with the outcome, and has no direct effect on the outcome). We used the prescriptions that the GPs had issued to their previous patients as a proxy for their preferences. For example, if the instrument was based on one previous prescription, physicians who previously prescribed varenicline would be categorized as a varenicline prescriber (7, 10).

We conducted an instrumental variable analysis to estimate the effectiveness of varenicline versus NRT for smoking cessation. Prescribing preferences for the prescriptions that GPs issued to their patients were used as the instrument (7, 10, 11). The instrumental variable analysis was conducted using the ivreg2 command (12). Additive structural mean models estimated via the generalized method of moments were used to calculate risk differences in rates of cessation (11, 13, 14). Smoking status was analyzed at 3, 6 and 9-months and 1, 2 and 4-years after exposure. A conventional linear regression was conducted to compare with the instrumental variable regression and both approaches were conducted using the same sample.

*Model adequacy checks:* Using standard procedures we compared the relative bias of linear regression and instrumental variable methods, by comparing the association between exposure and baseline covariates, and the association between the instrument and covariates (15).

## **Cox proportional hazards model**

In our protocol we stated that we would test the use of a Cox proportional hazards model, however our data did not meet the assumptions for proportional hazards as indicated by the test of proportional hazards using Schoenfeld residuals.

# Results

## Flow chart

Supplementary Material Figure 1. Flow chart of the number (N) of patients and records assessed for eligibility and reasons for exclusion

## List of NRT products

Supplementary Material Table 1 displays a list of NRT products that were prescribed in the study’s cohort.

Supplementary Material Table 1. List of NRT products prescribed in study cohort

| **Product code** | **Product name** |
| --- | --- |
| 41372 | NiQuitin Clear 21mg patches (GlaxoSmithKline Consumer Healthcare) |
| 41356 | Nicorette Microtab 2mg sublingual tablets (McNeil Products Ltd) |
| 5320 | Nicorette 10mg Inhalator (McNeil Products Ltd) |
| 2876 | Nicorette Citrus 2mg medicated chewing gum (Pfizer Ltd) |
| 46592 | Nicorette 15mg Inhalator (McNeil Products Ltd) |
| 6448 | Nicotine 21mg/24hours transdermal patches |
| 39572 | Nicorette invisi 10mg/16hours patches (McNeil Products Ltd) |
| 40730 | NiQuitin Minis Mint 1.5mg lozenges (GlaxoSmithKline Consumer Healthcare) |
| 5479 | Nicotine 10mg/16hours transdermal patches |
| 1703 | Nicorette 15mg Transdermal patch (Pharmacia Ltd) |
| 6565 | NiQuitin Mint 2mg medicated chewing gum (GlaxoSmithKline Consumer Healthcare) |
| 5502 | Nicotine 15mg/16hours transdermal patches |
| 45504 | Nicotine 1mg/dose oromucosal spray sugar free |
| 41368 | NiQuitin 21mg patches (GlaxoSmithKline Consumer Healthcare) |
| 45429 | Nicorette QuickMist 1mg/dose mouthspray (McNeil Products Ltd) |
| 40617 | Nicotinell TTS 20 patches (Novartis Consumer Health UK Ltd) |
| 5944 | Nicotine 10mg inhalation cartridges with device |
| 9806 | Nicotine 2mg lozenges sugar free |
| 6323 | Nicotine 2mg medicated chewing gum sugar free |
| 3404 | Niquitin 21mg Transdermal patch (GlaxoSmithKline Consumer Healthcare) |
| 4717 | Niquitin 14mg Transdermal patch (GlaxoSmithKline Consumer Healthcare) |
| 3818 | Nicotinell tts 30 sq cm Transdermal patch (Novartis Consumer Health UK Ltd) |
| 41505 | NiQuitin Clear 14mg patches (GlaxoSmithKline Consumer Healthcare) |
| 9804 | Nicotine 7mg/24hours transdermal patches |
| 40865 | NiQuitin Minis Mint 4mg lozenges (GlaxoSmithKline Consumer Healthcare) |
| 9591 | Nicotine 14mg/24hours transdermal patches |
| 4166 | Nicorette Citrus 4mg medicated chewing gum (Pfizer Ltd) |
| 39046 | Nicorette invisi 25mg/16hours patches (McNeil Products Ltd) |
| 36457 | Nicopatch 21mg/24hours transdermal patches (Pierre Fabre Ltd) |
| 41376 | Nicorette 15mg patches (McNeil Products Ltd) |
| 5515 | Nicotine 1mg Lozenge |
| 5440 | Nicorette 10mg Transdermal patch (Pharmacia Ltd) |
| 4704 | Niquitin 7mg Transdermal patch (GlaxoSmithKline Consumer Healthcare) |
| 11718 | Nicotine 2mg sublingual tablets sugar free |
| 41802 | Nicorette 5mg patches (McNeil Products Ltd) |
| 46717 | Nicotine 15mg inhalation cartridges with device |
| 5946 | Nicotinell 2mg Medicated chewing-gum (Novartis Consumer Health UK Ltd) |
| 41765 | Nicotinell Mint 2mg medicated chewing gum (Novartis Consumer Health UK Ltd) |
| 5877 | Nicorette 2mg microtab (Pharmacia Ltd) |
| 7303 | Nicotinell tts 10 sq cm Transdermal patch (Novartis Consumer Health UK Ltd) |
| 37646 | Nicotine 1.5mg lozenges sugar free |
| 39123 | Nicotine 25mg/16hours transdermal patches |
| 39166 | Nicorette invisi 15mg/16hours patches (McNeil Products Ltd) |
| 13048 | Nicotinell 4mg Medicated chewing-gum (Novartis Consumer Health UK Ltd) |
| 5606 | Nicotinell tts 20 sq cm Transdermal patch (Novartis Consumer Health UK Ltd) |
| 44106 | NiQuitin Minis Cherry 1.5mg lozenges (GlaxoSmithKline Consumer Healthcare) |
| 41474 | Nicorette 10mg patches (McNeil Products Ltd) |
| 6018 | Nicorette 5mg Transdermal patch (Pharmacia Ltd) |
| 5659 | NiQuitin 4mg lozenges original menthol mint (GlaxoSmithKline Consumer Healthcare) |
| 36635 | Nicopatch 14mg/24hours transdermal patches (Pierre Fabre Ltd) |
| 5700 | NiQuitin 2mg lozenges original menthol mint (GlaxoSmithKline Consumer Healthcare) |
| 41493 | Nicorette Icy White 4mg medicated chewing gum (McNeil Products Ltd) |
| 41864 | Nicorette Freshfruit 2mg medicated chewing gum (McNeil Products Ltd) |
| 5758 | Nicotine 4mg medicated chewing gum sugar free |
| 41801 | Nicorette Freshmint 2mg medicated chewing gum (McNeil Products Ltd) |
| 49319 | Nicorette Cools 4mg lozenges (McNeil Products Ltd) |
| 41779 | Nicorette Icy White 2mg medicated chewing gum (McNeil Products Ltd) |
| 41496 | Nicorette 500micrograms/dose nasal spray (McNeil Products Ltd) |
| 49305 | Nicorette Cools 2mg lozenges (McNeil Products Ltd) |
| 6630 | NiQuitin Mint 2mg lozenges (GlaxoSmithKline Consumer Healthcare) |
| 41753 | Nicorette Original 4mg medicated chewing gum (McNeil Products Ltd) |
| 6642 | NiQuitin Mint 4mg medicated chewing gum (GlaxoSmithKline Consumer Healthcare) |
| 5784 | Nicotine 4mg lozenges sugar free |
| 42016 | Nicorette Mint 2mg medicated chewing gum (McNeil Products Ltd) |
| 8571 | Nicotine 500micrograms/dose nasal spray |
| 57829 | NiQuitin Strips Mint 2.5mg oral films (GlaxoSmithKline Consumer Healthcare) |
| 40620 | Nicotinell TTS 30 patches (Novartis Consumer Health UK Ltd) |
| 41808 | Nicotinell Fruit 4mg medicated chewing gum (Novartis Consumer Health UK Ltd) |
| 1248 | Nicorette 10mg/ml Nasal spray (Pharmacia Ltd) |
| 5457 | Nicotine 5mg/16hours transdermal patches |
| 41507 | NiQuitin Clear 7mg patches (GlaxoSmithKline Consumer Healthcare) |
| 41809 | Nicorette Mint 4mg medicated chewing gum (McNeil Products Ltd) |
| 41426 | NiQuitin 7mg patches (GlaxoSmithKline Consumer Healthcare) |
| 41377 | Nicorette Original 2mg medicated chewing gum (McNeil Products Ltd) |
| 45603 | Nicorette Freshmint 2mg lozenges (McNeil Products Ltd) |
| 41485 | NiQuitin 14mg patches (GlaxoSmithKline Consumer Healthcare) |
| 41425 | Nicorette Freshmint 4mg medicated chewing gum (McNeil Products Ltd) |
| 46588 | Nicotinell Icemint 2mg medicated chewing gum (Novartis Consumer Health UK Ltd) |
| 42011 | Nicotinell Classic 4mg medicated chewing gum (Novartis Consumer Health UK Ltd) |
| 6593 | NiQuitin Mint 4mg lozenges (GlaxoSmithKline Consumer Healthcare) |
| 40683 | Nicotinell TTS 10 patches (Novartis Consumer Health UK Ltd) |
| 38958 | Nicotinell 1mg lozenges (Novartis Consumer Health UK Ltd) |
| 6698 | Nicotinell 2mg lozenges (Novartis Consumer Health UK Ltd) |
| 46701 | Nicotinell Icemint 4mg medicated chewing gum (Novartis Consumer Health UK Ltd) |
| 41778 | Nicorette Fruitfusion 4mg medicated chewing gum (McNeil Products Ltd) |
| 58034 | Nicotine 2.5mg orodispersible films sugar free |
| 41909 | Nicotinell Mint 4mg medicated chewing gum (Novartis Consumer Health UK Ltd) |
| 41040 | Nicorette lemon 2mg microtab (McNeil Products Ltd) |
| 5531 | Nicotinell 1mg Lozenge (Novartis Consumer Health UK Ltd) |
| 42048 | Nicotine bitartrate 1mg lozenges sugar free |
| 36618 | Nicopatch 7mg/24hours transdermal patches (Pierre Fabre Ltd) |
| 39521 | NiQuitin Pre-Quit Mint 4mg lozenges (GlaxoSmithKline Consumer Healthcare) |
| 41931 | Nicotinell Fruit 2mg medicated chewing gum (Novartis Consumer Health UK Ltd) |
| 37716 | Nicopass 1.5mg Lozenge (Wockhardt UK Ltd) |
| 41881 | Nicotinell Classic 2mg medicated chewing gum (Novartis Consumer Health UK Ltd) |
| 42047 | Nicotinell Liquorice 4mg medicated chewing gum (Novartis Consumer Health UK Ltd) |
| 48620 | Boots NicAssist 10mg Inhalator (The Boots Company Plc) |
| 58410 | NiQuitin Minis Orange 1.5mg lozenges (GlaxoSmithKline Consumer Healthcare) |
| 54102 | NiQuitin Pre-Quit Clear 21mg patches (GlaxoSmithKline Consumer Healthcare) |
| 25510 | Nicotine 2mg mint flavour chewing-gum |
| 58034 | Nicotine 2.5mg orodispersible films sugar free |
| 54102 | NiQuitin Pre-Quit Clear 21mg patches (GlaxoSmithKline Consumer Healthcare) |
| 49088 | NiQuitin Clear 21mg patches (Waymade Healthcare Plc) |
| 25510 | Nicotine 2mg mint flavour chewing-gum |

## Baseline characteristics: Comparison of raw and imputed data

Supplementary Material Table 2 shows that there were few clinically meaningful differences between the imputed and non-imputed IMD and BMI data.

| Supplementary Material Table 2. Comparison of imputed and non-imputed data for BMI and IMD | | | | | | |
| --- | --- | --- | --- | --- | --- | --- |
|  | **BMI**  **Mean (standard deviation)** | | | **IMD**  **Median** | | |
|  | **Whole sample** | **NRT** | **Varenicline** | **Whole sample** | **NRT** | **Varenicline** |
| Non-imputed | 26.5 (5.6)  N=188,967 | 26.5 (5.7)  N=128,407 | 26.5 (5.4)  N=60,560 | 12  N=124,781 | 12  N=83,133 | 12  N=41,648 |
| Imputed | 26.4 (6.1)  N=220,136 | 26.4 (6.4)  N=149,526 | 26.5 (5.9)  N=70,610 | 12  N=220,136 | 12  N=149,526 | 12  N=70,610 |

## Propensity score matching

### Summary of results

70,609 patients prescribed varenicline (one patient was lost due to common support restrictions) were matched to 70,609 patients with similar propensity scores prescribed NRT. The propensity score model achieved a good balance between groups (Supplementary Material Figure 2 and Supplementary Material Figure 3). Models adjusted for propensity score indicated that varenicline was associated with increased odds of quitting smoking compared to NRT at all follow-ups and the 95% confidence intervals were precise (Supplementary Material Table 3), at 2-year follow-up the odds-ratio and 95% confidence interval were 1.26 (1.23 to 1.29), p<0.0001. The association attenuated slightly at 4-year follow-up, but the direction and size of the estimates were consistent with those derived from the multivariable adjusted logistic regression model.

### % bias difference and covariate balance check

Baseline covariates were included in the final propensity score model. Supplementary Material Figure 2 displays the % bias difference between treatment and control groups, before and after propensity score matching. I.e. the % difference of the sample means in the control and treatment groups, as a % of the square root of the average of the sample variances in each group (1). The propensity score analysis balanced the covariates.

Supplementary Material Figure 2. % bias difference between treatment and control groups, before and after propensity score matching

### Distribution plots: Kernel density estimation of groups’ propensity scores before and after propensity score matching

Kernel density estimation plots of exposure groups’ propensity scores before and after matching indicates that there was an adequate common support region to conduct the propensity score matching procedure (Supplementary Material Figure 3) and that the propensity score distributions overlapped after matching (Supplementary Material Figure 3).

Supplementary Material Figure 3. Kernel density estimation of groups’ propensity scores before and after matching

### Propensity score matched logistic regression model for the association between prescription of varenicline versus NRT and smoking cessation at all follow ups

Models adjusted for propensity score indicated that varenicline was associated with increased odds of quitting smoking compared to NRT at all follow-ups, and the 95% confidence intervals were precise (Supplementary Material Table 3). The association attenuated slightly overtime but the direction and precision of the association remained consistent.

| Supplementary Material Table 3. Propensity score matched models: Odds-ratios and 95% confidence intervals for the association between prescription of varenicline versus NRT and smoking cessation at 3, 6 and 9-months and 1, 2 and 4-years after exposure, N=141,218* | | | | | |
| --- | --- | --- | --- | --- | --- |
| **Odds-ratio (95% confidence interval) ‡** | | | | | |
| **3-months** | **6-months** | **9-months** | **1-year** | **2-years** | **4-years** |
| 1.42  (1.37 to 1.48) | 1.45  (1.40 to 1.51) | 1.40  (1.35 to 1.45) | 1.35  (1.30 to 1.39) | 1.27  (1.23 to 1.30) | 1.19  (1.16 to 1.22) |
| ‡Model adjusted for propensity score. *Missing BMI and IMD values were imputed using multiple imputation (16). | | | | | |

## Instrumental variable analysis

### Summary of results

In the instrumental variable analysis 216,022 patients were included. 4,114 patients were excluded as they were the first individuals to consult with each GP; for these individuals, we did not have data about the GP’s previous prescriptions to create the instrument. Supplementary Material Table 4 displays the risk difference per 100 patients and 95% confidence interval at each follow-up from the instrumental variable and regression models. The partial adjusted linear regression models indicated that patients prescribed varenicline were more likely to quit smoking compared to those prescribed NRT at all follow-ups (Supplementary Material Table 4 and Supplementary Material Figure 5); the risk difference per 100 patients and 95% confidence interval at 2-year follow-up were; 5.03 (4.57 to 5.50), p<0.0001. The direction and precision of this association remained consistent overtime, but the association was slightly attenuated at the 4-year follow-up. The instrumental variable results were consistent with the multivariable linear regression results at all follow-up periods (all Hausman-test p-values>0.05) (Supplementary Material Table 4).

The F-statistics were large (>12,000), indicating that our results were unlikely to suffer from weak instrument bias (Supplementary Material Table 4). We investigated the relative bias of the multivariable adjusted and instrumental variable regressions and found the instrumental variable analysis was at much less risk of confounding (Supplementary Material Figure 4).

### Relative bias of linear regression and instrumental variable methods

Supplementary Material Figure 4 shows the relative bias of linear regression and instrumental variable methods. Squares (□) indicate the bias component in the linear regression model, and triangles (△) indicate the bias component in the instrumental variable model. The instrumental variable bias terms were generally smaller than the linear regression bias terms. This is consistent with the instrumental variable analysis being less biased than the multivariable adjusted regression.

Supplementary Material Figure 4. Relative bias of linear regression and instrumental variable methods: Binary and continuous outcomes

### Quit rates by instrumental variable condition at follow-ups.

Supplementary Material Figure 5 shows that the proportion of quitters was higher at all follow-ups for patients in the instrumental variable condition for varenicline, compared to those in the NRT condition.

Supplementary Material Figure 5. Point prevalence quit rates by instrumental variable condition at 3, 6 and 9-months and 1, 2 and 4-years after exposure, N=216,022*

*4114 patients were excluded from the instrumental variable analysis as they were the first individuals to consult with each GP, thus for these individuals we do not have data about the GP previous prescribing behavior to enable generation of the instrument.

### Instrumental variable and conventional regression models

Supplementary Material Table 4 shows that patients prescribed varenicline were more likely to stop smoking compared to those prescribed NRT at all follow-ups.

| Supplementary Material Table 4. Conventional and instrumental variable linear regression models: Risk difference per 100 patients treated and 95% confidence intervals for the association between varenicline versus NRT and smoking cessation at 3, 6 and 9-months and 1, 2 and 4-years after exposure, N=216,022 | | | | | | |
| --- | --- | --- | --- | --- | --- | --- |
| **Model** | **3-months** | **6-months** | **9-months** | **1-year** | **2-years** | **4-years** |
|  | **Risk difference (95% confidence interval)** | | | | | |
| Linear regression model‡ | 5.09  (4.61 to 5.58) | 6.41  (5.91 to 6.91) | 6.15  (5.65 to 6.64) | 6.15  (5.65 to 6.64) | 5.03  (4.57 to 5.50) | 4.33  (3.86 to 4.80) |
| Instrumental variable linear regression model‡‡ | 4.13  (2.24 to 6.01) | 6.51  (4.52 to 8.500) | 6.42  (4.40 to 8.43) | 5.97  (3.94 to 8.00) | 4.76  (2.77 to 6.74) | 4.06  (2.09 to 6.03) |
| Partial F-statistic* | 12466.37 | 12466.37 | 12466.37 | 12466.37 | 12466.37 | 12466.37 |
| Hausman test | 0.46 | 0.52 | 0.86 | 0.93 | 0.12 | 0.11 |
|  | P=0.50 | P=0.47 | P=0.35 | P=0.33 | P=0.73 | P=0.74 |
| ‡Conventional linear regression model adjusted for age, sex and year of 1^st^ prescription. ‡‡ Instrumental variable linear regression model adjusted only for year of 1^st^ prescription. *This table presents Cragg-Donald Wald F statistics (i.e. the test of the association of the instrument and the prescription) and the Hausman test of endogenous the exposure. 4114 patients were excluded from the instrumental variable analysis as they were the first individuals to consult with each GP, thus for these individuals we do not have data about the GP previous prescribing behavior to enable generation of the instrument. | | | | | | |

## Socioeconomic descriptive data as measured by the Index of Multiple Deprivation (IMD)

Supplementary Material Figure 6 shows the number and proportion of patients prescribed smoking cessation medications by level of Index of Multiple Deprivation (IMD) score. Patients from the most deprived areas were less likely to be prescribed varenicline compared to those from the least deprived areas; age and sex adjusted odds-ratio were 0.91 (95% confidence interval: 0.90 to 0.92), p<0.0001.

Supplementary Material Figure 6. Number (N), % and 95% confidence interval of patients prescribed smoking cessation medication by level of deprivation. Missing IMD data were not imputed. Quartiles are displayed for presentation purposes only.

## Effectiveness of varenicline stratified by neighbourhood depreviation

Supplementary Material Table 5 and Supplementary Material Table 6 show that varenicline appeared to be slightly more effective in patients from the least deprived areas at 3-months to 1-year after first prescription, but was similarly effective in patients from the most and least deprived areas at 2 and 4-years follow-up. The propensity score matched analysis produced similar results to the multivariable adjusted analysis; however in the propensity score models the strength of the association in patients from least deprived areas dissipated at 2 and 4-years follow-up. The instrumental variable analysis provided little evidence of an association, but was under powered.

Supplementary Material Table 5. Effectiveness of varenicline at 3, 6 and 9-months, and 1, 2 and 4-years after first prescription in the least deprived areas (IMD scores 1 to 10). Effect estimates and 95% confidence intervals presented for each analytic technique.

| **Analysis technique** | **3-months** | **6-months** | **9-months** | **1-year** | **2-years** | **4-years** |
| --- | --- | --- | --- | --- | --- | --- |
|  | **Effect estimate (95% confidence interval)** | | | | | |
| Logistic regression model^1^ | 1.45 (1.37 to 1.54) | 1.50 (1.42 to 1.57) | 1.46 (1.39 to 1.54) | 1.38 (1.31 to 1.45) | 1.29 (1.23 to 1.35) | 1.19 (1.14 to 1.24) |
|  | p<0.0001 | p<0.0001 | p<0.0001 | p<0.0001 | p<0.0001 | p<0.0001 |
| Propensity score matched logistic regression model^2^ | 1.41 (1.33 to 1.50) | 1.43 (1.35 to 1.51) | 1.23 (1.17 to 1.30) | 1.18 (1.08 to 1.28) | 1.07 (0.99 to 1.16) | 1.00 (0.92 to 1.08) |
|  | p<0.0001 | p<0.0001 | p<0.0001 | p=0.0003 | p=0.1063 | p=0.9841 |
| Instrumental variable analysis^3^ | 1.49 (-2.05 to 5.04) | 3.84 (0.05 to 7.64) | 4.46 (0.64 to 8.28) | 4.13 (0.32 to 7.93) | 2.79 (-1.07 to 6.65) | 0.59 (-3.30 to 4.48) |
|  | p=0.4097 | p=0.0473 | p=0.0222 | p=0.0334 | p=0.1567 | p=0.7661 |
| 1 N= 52,534; data reported are partial adjusted odds-ratios, models were adjusted for age, sex and year of prescription. 2 N= 31,407; data reported are odds-ratios, models were adjusted for propensity score. 3 N= 51,436; data reported are risk difference per 100 patients treated; models were adjusted for year of prescription. ‡Missing IMD values were not imputed, and patients with missing IMD data were excluded from analyses to ensure comparability of results across samples. | | | | | | |

Supplementary Material Table 6. Effectiveness of varenicline at 3, 6 and 9-months, and 1, 2 and 4-years after first prescription in the most deprived areas (IMD scores 11 to 20). Effect estimates and 95% confidence intervals presented for each analytic technique.‡

| **Analysis technique** | **3-months** | **6-months** | **9-months** | **1-year** | **2-years** | **4-years** |
| --- | --- | --- | --- | --- | --- | --- |
|  | **Effect estimate (95% confidence interval)** | | | | | |
| Logistic regression model^1^ | 1.38 (1.31 to 1.46) | 1.43 (1.36 to 1.50) | 1.37 (1.31 to 1.44) | 1.33 (1.27 to 1.39) | 1.28 (1.23 to 1.34) | 1.22 (1.17 to 1.26) |
|  | p<0.0001 | p<0.0001 | p<0.0001 | p<0.0001 | p<0.0001 | p<0.0001 |
| Propensity score matched logistic regression model^2^ | 1.32 (1.23 to 1.41) | 1.35 (1.27 to 1.42) | 1.23 (1.17 to 1.29) | 1.45 (1.35 to 1.54) | 1.37 (1.29 to 1.46) | 1.32 (1.24 to 1.40) |
|  | p<0.0001 | p<0.0001 | p<0.0001 | p<0.0001 | p<0.0001 | p<0.0001 |
| Instrumental variable analysis^3^ | 0.66 (-2.44 to 3.75) | 3.09 (-0.11 to 6.30) | 2.07 (-1.20 to 5.33) | 2.15 (-1.21 to 5.52) | 2.85 (-0.40 to 6.11) | 2.54 (-0.64 to 5.72) |
|  | p=0.6785 | p=0.0584 | p=0.2144 | p=0.2098 | p=0.0856 | p=0.1171 |
| 1 N= 72,247; data reported are partial adjusted odds-ratios, models were adjusted for age, sex and year of prescription. 2 N= 40,243; data reported are odds-ratios, models were adjusted for propensity score. 3 N= 71,041; data reported are risk difference per 100 patients treated; models were adjusted for year of prescription. ‡Missing IMD values were not imputed, and patients with missing IMD data were excluded from analyses to ensure comparability of results across samples. | | | | | | |

## Sensitivity analysis: Missing outcome data

Table 6 shows the proportion of patients who were missing outcome data by exposure group at all follow-ups. The proportions of missing data were similar between exposure groups. The proportion of missing data decreased overtime.

Supplementary Material Table 7. Number (N) and % of patients missing outcome data by treatment at all follow-ups

|  | **3-months** | **6-months** | **9-months** | **1-year** | **2-years** | **4-years** |
| --- | --- | --- | --- | --- | --- | --- |
| NRT | 69.4% | 56.0% | 45.9% | 37.2% | 19.8% | 10.8% |
|  | N=103,743/ 149,526 | N=83,803/ 149,526 | N=68,665/ 149,526 | N=55,696/ 149,526 | N=29,608/ 149,526 | N=16,151/ 149,526 |
| Varenicline | 65.6% | 53.6% | 44.9% | 37.4% | 21.4% | 12.7% |
|  | 46,312/  70,610 | 37,819/  70,610 | 31,732/  70,610 | 26,400/  70,610 | 15,129/  70,610 | 8,984/  70,610 |

Supplementary Material Table 8 compares baseline characteristics between patients who were missing outcome data at 2-years to the whole cohort. Patients with missing outcome data at 2-years were on-average, younger, male, visited the GP fewer times per year, and had fewer comorbidities.

Supplementary Material Table 8. Comparison of baseline characteristics between the whole sample and patients missing 2-year outcome data

|  | **NRT** | | **Varenicline** | |
| --- | --- | --- | --- | --- |
| **Characteristic** | **Patients with missing outcome data**  **(N=29,608)** | **Whole sample (N= 149,526)** | **Patients with missing outcome data**  **(N= 15,129)** | **Whole sample (N= 70,610)** |
| Age at time of first prescription^1^ | 40.7 (14.2) | 46.4 (15.5) | 40.5 (11.9) | 44.5 (13.2) |
| Sex (female) | 43.9% (12,988) | 53.7% (80,348) | 39.6% (5,995) | 50.2% (35,466) |
| Index of multiple deprivation score (IMD)*^2^ | 12 | 12 | 12 | 12 |
| Mean number of GP visits 1-year prior to first prescription^1^ | 5.5 (5.7) | 7.9 (7.4) | 4.5 (4.6) | 6.3 (6.1) |
| BMI*^1^ | 26.4 (2.8) | 26.4 (6.4) | 26.5 (2.7) | 26.5 (5.9) |
| Year of first prescription^2^ | 2009 | 2009 | 2010 | 2010 |
| Days of history^1^ | 3,060.3 (1894.2) | 3158.7 (1892.1) | 3,210.6 (2011.3) | 3283.9 (1976.6) |
| Comorbidity ever (Charlson Index) (17, 18) | 21.8% (6,465) | 37.6% (56,274) | 19.7% (2,981) | 31.9% (22,523) |
| Alcohol misuse | 7.0% (2,076) | 8.3% (12,422) | 4.9% (742) | 6.0 (4,199) |
| Drug misuse ever | 3.2% (948) | 3.1% (4,595) | 1.94% (294) | 1.9% (1,357) |
| Bipolar ever | <1% (109) | 1% (1,464) | <1% (14) | <1% (160) |
| Depression ever | 28.4% (8,398) | 35.0% (52,233) | 23.5% (3,554) | 29.2% (20,615) |
| Neurotic disorder ever | 19.4% (5,737) | 24.7% (36,921) | 15.5% (2,340) | 20.1% (14,189) |
| Self-harm ever | 9.1% (2,700) | 10.6% (15,903) | 7.5% (1,137) | 8.7% (6,169) |
| Other mental disorder ever | 6.0% (1,780) | 6.9% (10,343) | 3.8% (574) | 4.0% (2,832) |
| Antidepressant prescription ever | 41.5% (12,282) | 50.1% (74,921) | 35.1% (5,304) | 43.1% (30,435) |
| Antipsychotic prescription ever | 13.7% (4,066) | 20.0% (29,873) | 10.2% (1,547) | 14.8% (10,459) |
| Hypnotics/anxiolytics prescription ever | 16.2% (4,784) | 21.1% (31,513) | 13.5% (2,036) | 17.6% (12,415) |
| Other psychotropic medication | <1% (117) | <1% (473) | <1% (32) | <1% (120) |
| *Missing data: BMI data was missing for 14.2% (N= 31,169); IMD data was missing for 43.3% (N= 95,355). Missing BMI and IMD values were imputed using multiple imputation (16). 1 Data presented are mean and standard deviation. 2 Data presented are median. | | | | |

Supplementary Material Figure 7 presents odds-ratios and 95% confidence intervals for association between prescription of varenicline versus NRT, and smoking cessation at all follow-ups; these data were from the sensitivity model in which missing outcome data were imputed, and these effect estimates are similar to the main analysis in which patients with missing outcome data were assumed to be smoking (Main paper Figure 2).

Supplementary Material Figure 7. Multivariable logistic regression model, in which missing outcome data were imputed using multivariate multiple imputation: Fully adjusted odds-ratios and 95% confidence intervals for the association between prescription of varenicline versus NRT and smoking cessation at follow-ups. The difference in smoking cessation rates peaks at 6-months and declines over the following 3.5 years.

Supplementary Material Table 9 shows that effect estimates derived from the main analysis in which patients with missing outcome data were assumed to be smoking were similar to estimates derived from models in which missing outcome data were imputed.

| Supplementary Material Table 9. Multivariable logistic regression models: Comparison of estimates derived from the main analysis and the sensitivity analysis. Fully adjusted odds-ratios and 95% confidence intervals for the association between varenicline versus NRT and smoking cessation at 3, 6 and 9-months and 1, 2 and 4-years after exposure, N=220,136 | | | | | | |
| --- | --- | --- | --- | --- | --- | --- |
|  | **3-months** | **6-months** | **9-months** | **1-year** | **2-years** | **4-years** |
|  | **Odds-ratios (95% confidence interval) ‡‡** | | | | | |
| Main analysis (missing outcome data=smoking) (19) | 1.42  (1.38 to 1.47) | 1.46  (1.42 to 1.50) | 1.40  (1.36 to 1.44) | 1.34  (1.31 to 1.38) | 1.26  (1.23 to 1.29) | 1.19  (1.16 to 1.21) |
| Sensitivity (missing outcome data=multiply imputed) | 1.37  (1.33 to 1.40) | 1.40  (1.36 to 1.44) | 1.34  (1.31 to 1.38) | 1.29  (1.26 to 1.33) | 1.23  (1.20 to 1.26) | 1.17  (1.14 to 1.19) |
| ‡‡ Data reported are odds-ratios and models were fully adjusted for all baseline covariates. Missing BMI and IMD values were imputed using multiple imputation (16). | | | | | | |

## Cox proportional hazards model: Test of proportional-hazards assumption

In our protocol we proposed to use a Cox proportional hazards model (20). Supplementary Material Table 10 presents results from the test of proportional-hazards assumption using Schoenfeld residuals. The global summary test shows that the model did not meet the Cox proportional hazards assumption; therefore, we did not use the Cox model in our main analysis.

Supplementary Material Table 10. Cox proportional hazards model: Test of proportional-hazards assumption

| **Variable** | **Rho** | **P-Value** |
| --- | --- | --- |
| Smoking cessation medication | 0.00160 | p<0.0001 |
| Days of history | 0.00263 | p<0.0001 |
| Index of multiple deprivation score (IMD) | -0.00685 | p<0.0001 |
| Mean number of GP visits in year prior to 1st prescription | -0.03636 | p<0.0001 |
| BMI | -0.01598 | -0.03636 |
| Age | -0.08533 | p<0.0001 |
| Sex (female=1) | -0.01444 | p<0.0001 |
| Year of first prescription (2006) | 0.00285 | p<0.0001 |
| Year of first prescription (2007) | 0.00295 | p<0.0001 |
| Year of first prescription (2008) | 0.00322 | p<0.0001 |
| Year of first prescription (2009) | 0.00339 | p<0.0001 |
| Year of first prescription (2010) | 0.00387 | p<0.0001 |
| Year of first prescription (2011) | 0.00388 | p<0.0001 |
| Year of first prescription (2012) | 0.00319 | p<0.0001 |
| Year of first prescription (2013) | 0.00294 | p<0.0001 |
| Year of first prescription (2014) | 0.00282 | p<0.0001 |
| Comorbidity ever (Charlson Index) | -0.06645 | p<0.0001 |
| Alcohol misuse ever | -0.00015 | 0.1830 |
| Drug misuse ever | 0.00384 | p<0.0001 |
| Bipolar ever | -0.00210 | p<0.0001 |
| Depression ever | -0.00184 | p<0.0001 |
| Neurotic disorder ever | -0.00311 | p<0.0001 |
| Self-harm ever | 0.00237 | p<0.0001 |
| Antidepressant prescription ever | 0.01230 | 0.5121 |
| Antipsychotics ever | 0.00008 | p<0.0001 |
| Hypnotic or anxiolytic medication prescription ever | 0.00894 | p<0.0001 |
| Prescription of rare psychotropic medication | 0.00837 | p<0.0001 |
| Rare mental disorder ever | -0.01038 | p<0.0001 |
| Global test (Degrees of freedom= 28) | | p<0.0001 |

# References

1. Leuven E, Sianesi B. PSMATCH2: STATA module to perform full Mahalanobis and propensity score matching, common support graphing, and covariate imbalance testing. Boston, MA, USA: Boston College Department of Economics; 2003.

2. Rosenbaum P, Rubin D. The central role of the propensity score in observational studies for causal effects. Biometrika. 1983;70(1):41-55.

3. Rosenbaum P, Rubin D. Reducing bias in observational studies using subclassification on the propensity score. Journal of the American Statistical Association. 1984;79(387):516-24.

4. Glynn RJ, Schneeweiss S, Stürmer T. Indications for propensity scores and review of their use in pharmacoepidemiology. Basic & clinical pharmacology & toxicology. 2006;98(3):253-9.

5. Taylor G, Girling A, McNeill A, Aveyard P. Does smoking cessation result in improved mental health? A comparison of regression modelling and propensity score matching. BMJ open. 2015;5(10):e008774.

6. Taylor G, Taylor A, Munafò MR, McNeill A, Aveyard P. Does smoking reduction worsen mental health? A comparison of two observational approaches. BMJ open. 2015;5(5):e007812.

7. Thomas KH, Martin RM, Davies NM, Metcalfe C, Windmeijer F, Gunnell D. Smoking cessation treatment and risk of depression, suicide, and self harm in the Clinical Practice Research Datalink: prospective cohort study. BMJ: British Medical Journal. 2013;347:f5704.

8. Thoemmes F, Kim E. A systematic review of propensity score methods in the social sciences. Multivariate Behavioral Research. 2011;46(1):90-118.

9. King G, Zeng L. The dangers of extreme counterfactuals. Political Analysis. 2005;14(2):131-59.

10. Davies NM, Gunnell D, Thomas KH, Metcalfe C, Windmeijer F, Martin RM. Physicians' prescribing preferences were a potential instrument for patients' actual prescriptions of antidepressants. Journal of clinical epidemiology. 2013;66(12):1386-96.

11. Clarke PS, Windmeijer F. Instrumental variable estimators for binary outcomes. Journal of the American Statistical Association. 2012;107(500):1638-52.

12. Baum C SM, Stillman S. IVREG2: Stata module for extended instrumental variables/2SLS and GMM estimation. Stata Software; 2002.

13. Hansen LP, Singleton KJ. Generalized instrumental variables estimation of nonlinear rational expectations models. Econometrica: Journal of the Econometric Society. 1982:1269-86.

14. Clarke PS, Windmeijer F. Identification of causal effects on binary outcomes using structural mean models. Biostatistics. 2010;11(4):756-70.

15. Jackson JW, Swanson SA. Toward a clearer portrayal of confounding bias in instrumental variable applications. Epidemiology (Cambridge, Mass). 2015;26(4):498.

16. Royston PW, I. Multiple Imputation by Chained Equations (MICE): Implementation in Stata. Journal of Statistical Software. 2011;Volume 45( Issue 4):1-20.

17. Khan NF, Perera R, Harper S, Rose PW. Adaptation and validation of the Charlson Index for Read/OXMIS coded databases. BMC family practice. 2010;11(1):1.

18. Charlson ME, Pompei P, Ales KL, MacKenzie CR. A new method of classifying prognostic comorbidity in longitudinal studies: development and validation. Journal of chronic diseases. 1987;40(5):373-83.

19. West R, Hajek P, Stead L, Stapleton J. Outcome criteria in smoking cessation trials: proposal for a common standard. Addiction. 2005;100(3):299-303.

20. Davies NM, Taylor G, Taylor AE, Thomas KH, Windmeijer F, Martin RM, et al. What are the effects of varenicline compared with nicotine replacement therapy on long-term smoking cessation and clinically important outcomes? Protocol for a prospective cohort study. BMJ Open. 2015;5(11):e009665
