# Supplementary material for: The effectiveness of varenicline versus nicotine replacement therapy on long-term smoking cessation in primary care: a prospective cohort study of electronic medical records
Source: Int J Epidemiol. 2017 Jun 26;46(6):1948–57. doi: 10.1093/ije/dyx109 (PMC5837420; doi:10.1093/ije/dyx109)
Supplement: Supplementary Data [file ije-2017-02-0136-file003_dyx109.docx]

Supplementary Material: Code lists

Table of Contents

[Exposure 3](#_Toc473622498)

[Varenicline 3](#_Toc473622499)

[Nicotine replacement therapy (NRT) 4](#_Toc473622500)

[Outcome 9](#_Toc473622501)

[Smoking status 9](#_Toc473622502)

[Covariates 12](#_Toc473622503)

[Alcohol misuse 12](#_Toc473622504)

[Anxiety, neurotic, stress-related and somatoform disorder (ICD-10 F40-48) 17](#_Toc473622505)

[Autism (ICD-10 Chapters F84.0-84.1, F84.4-84.9) 23](#_Toc473622506)

[Bipolar disorder diagnosis (ICD-10 Chapters F10-31 24](#_Toc473622507)

[Dementia diagnosis (ICD-10 Chapters F00-02) 28](#_Toc473622508)

[Depression diagnosis (ICD-10 Chapters F32-39) 31](#_Toc473622509)

[Drug misuse 37](#_Toc473622510)

[Eating disorder (ICD-10 F50) 44](#_Toc473622511)

[Learning disorder (ICD-10 F70-79, F81.0-81.9) 46](#_Toc473622512)

[Major chronic illness (Charlson Index) 51](#_Toc473622513)

[Other behavioural disorder (ICD-10 F90-98) 159](#_Toc473622514)

[Personality disorder (ICD-10 F60-61) 163](#_Toc473622515)

[Schizophrenia and related disorder (ICD-10 Chapters F20-F29) 166](#_Toc473622516)

[Self-harm 173](#_Toc473622517)

[Antidepressant medication prescription (BNF Chapter 4.3 Antidepressant drugs) 183](#_Toc473622518)

[Antipsychotic medication prescription (BNF Chapter 4.2 Drugs used in psychoses and related disorders) 203](#_Toc473622519)

[Central nervous system (CNS) stimulant prescription (BNF Chapter 4.4 CNS simulants and drugs used for attention deficit hyperactivity disorder) 224](#_Toc473622520)

[Dementia medication prescription (BNF Chapter 4.11 Dementia drugs) 228](#_Toc473622521)

[Hypnotics and anxiolytics prescription (BNF Chapters 4.1 Hypnotics and anxiolytics) 232](#_Toc473622522)

# Exposure

## Varenicline

Varenicline code list

| Product code | Product name |
| --- | --- |
| 27410 | Champix titration pack (Pfizer Ltd) |
| 27411 | Champix 1mg tablets (Pfizer Ltd) |
| 27412 | Varenicline 1mg tablets and Varenicline 500microgram tablets |
| 27414 | Varenicline 1mg tablets |
| 35035 | Champix 0.5mg tablets (Pfizer Ltd) |
| 35089 | Varenicline 500microgram tablets |
| 49204 | Champix titration pack (Mawdsley-Brooks & Company Ltd) |
| 49901 | Champix 1mg tablets (Waymade Healthcare Plc) |
| 50541 | Champix titration pack (Sigma Pharmaceuticals Plc) |

## Nicotine replacement therapy (NRT)

NRT code list

| Product code | Product name |
| --- | --- |
| 30086 | NICORETTE MICROTAB |
| 41923 | Nicotine 15mg/16 hours transdermal patches and Nicotine 2mg medicated chewing gum sugar free |
| 42400 | Nicorette combi 15mg transdermal patches + 2mg medicated gum (McNeil Products Ltd) |
| 57829 | NiQuitin Strips Mint 2.5mg oral films (GlaxoSmithKline Consumer Healthcare) |
| 58410 | NiQuitin Minis Orange 1.5mg lozenges (GlaxoSmithKline Consumer Healthcare) |
| 58034 | Nicotine 2.5mg orodispersible films sugar free |
| 8297 | NICOTINE TRANSDERMAL PATCH 20CM |
| 116 | NICOTINE TRANSDERMAL PATCH 30CM |
| 1248 | Nicorette 10mg/ml Nasal spray (Pharmacia Ltd) |
| 1703 | Nicorette 15mg Transdermal patch (Pharmacia Ltd) |
| 2876 | Nicorette Citrus 2mg medicated chewing gum (Pfizer Ltd) |
| 3404 | Niquitin 21mg Transdermal patch (GlaxoSmithKline Consumer Healthcare) |
| 3818 | Nicotinell tts 30 sq cm Transdermal patch (Novartis Consumer Health UK Ltd) |
| 4166 | Nicorette Citrus 4mg medicated chewing gum (Pfizer Ltd) |
| 4704 | Niquitin 7mg Transdermal patch (GlaxoSmithKline Consumer Healthcare) |
| 4717 | Niquitin 14mg Transdermal patch (GlaxoSmithKline Consumer Healthcare) |
| 5320 | Nicorette 10mg Inhalator (McNeil Products Ltd) |
| 5440 | Nicorette 10mg Transdermal patch (Pharmacia Ltd) |
| 5457 | Nicotine 5mg/16hours transdermal patches |
| 5479 | Nicotine 10mg/16hours transdermal patches |
| 5502 | Nicotine 15mg/16hours transdermal patches |
| 5515 | Nicotine 1mg Lozenge |
| 5531 | Nicotinell 1mg Lozenge (Novartis Consumer Health UK Ltd) |
| 5606 | Nicotinell tts 20 sq cm Transdermal patch (Novartis Consumer Health UK Ltd) |
| 5659 | NiQuitin 4mg lozenges original menthol mint (GlaxoSmithKline Consumer Healthcare) |
| 5700 | NiQuitin 2mg lozenges original menthol mint (GlaxoSmithKline Consumer Healthcare) |
| 5758 | Nicotine 4mg medicated chewing gum sugar free |
| 5784 | Nicotine 4mg lozenges sugar free |
| 5944 | Nicotine 10mg inhalation cartridges with device |
| 5946 | Nicotinell 2mg Medicated chewing-gum (Novartis Consumer Health UK Ltd) |
| 6018 | Nicorette 5mg Transdermal patch (Pharmacia Ltd) |
| 6323 | Nicotine 2mg medicated chewing gum sugar free |
| 6448 | Nicotine 21mg/24hours transdermal patches |
| 6565 | NiQuitin Mint 2mg medicated chewing gum (GlaxoSmithKline Consumer Healthcare) |
| 6593 | NiQuitin Mint 4mg lozenges (GlaxoSmithKline Consumer Healthcare) |
| 6630 | NiQuitin Mint 2mg lozenges (GlaxoSmithKline Consumer Healthcare) |
| 6642 | NiQuitin Mint 4mg medicated chewing gum (GlaxoSmithKline Consumer Healthcare) |
| 7303 | Nicotinell tts 10 sq cm Transdermal patch (Novartis Consumer Health UK Ltd) |
| 7644 | Nicabate 21mg Transdermal patch (Marion Merrell Dow Ltd) |
| 8571 | Nicotine 500micrograms/dose nasal spray |
| 9591 | Nicotine 14mg/24hours transdermal patches |
| 9804 | Nicotine 7mg/24hours transdermal patches |
| 9806 | Nicotine 2mg lozenges sugar free |
| 10527 | Nicabate 14mg Transdermal patch (Marion Merrell Dow Ltd) |
| 10623 | Nicabate 7mg Transdermal patch (Marion Merrell Dow Ltd) |
| 13048 | Nicotinell 4mg Medicated chewing-gum (Novartis Consumer Health UK Ltd) |
| 25510 | Nicotine 2mg mint flavour chewing-gum |
| 25516 | Nicotine 4mg mint flavour chewing-gum |
| 25523 | Nicorette 2mg mint flavour chewing-gum (Pharmacia Ltd) |
| 27311 | Niconil 22mg/24 hr Transdermal patch (Elan Pharma) |
| 29680 | Niconil 11mg/24 hr Transdermal patch (Elan Pharma) |
| 31939 | Nicorette 4mg mint flavour chewing-gum (Pharmacia Ltd) |
| 33392 | Nicotine 22mg/24 hr Transdermal patch |
| 36457 | Nicopatch 21mg/24hours transdermal patches (Pierre Fabre Ltd) |
| 36618 | Nicopatch 7mg/24hours transdermal patches (Pierre Fabre Ltd) |
| 36635 | Nicopatch 14mg/24hours transdermal patches (Pierre Fabre Ltd) |
| 37646 | Nicotine 1.5mg lozenges sugar free |
| 37716 | Nicopass 1.5mg Lozenge (Wockhardt UK Ltd) |
| 39046 | Nicorette invisi 25mg/16hours patches (McNeil Products Ltd) |
| 39123 | Nicotine 25mg/16hours transdermal patches |
| 39166 | Nicorette invisi 15mg/16hours patches (McNeil Products Ltd) |
| 39521 | NiQuitin Pre-Quit Mint 4mg lozenges (GlaxoSmithKline Consumer Healthcare) |
| 39572 | Nicorette invisi 10mg/16hours patches (McNeil Products Ltd) |
| 40617 | Nicotinell TTS 20 patches (Novartis Consumer Health UK Ltd) |
| 40620 | Nicotinell TTS 30 patches (Novartis Consumer Health UK Ltd) |
| 40683 | Nicotinell TTS 10 patches (Novartis Consumer Health UK Ltd) |
| 40730 | NiQuitin Minis Mint 1.5mg lozenges (GlaxoSmithKline Consumer Healthcare) |
| 40865 | NiQuitin Minis Mint 4mg lozenges (GlaxoSmithKline Consumer Healthcare) |
| 41040 | Nicorette lemon 2mg microtab (McNeil Products Ltd) |
| 41368 | NiQuitin 21mg patches (GlaxoSmithKline Consumer Healthcare) |
| 41372 | NiQuitin Clear 21mg patches (GlaxoSmithKline Consumer Healthcare) |
| 41376 | Nicorette 15mg patches (McNeil Products Ltd) |
| 41377 | Nicorette Original 2mg medicated chewing gum (McNeil Products Ltd) |
| 41425 | Nicorette Freshmint 4mg medicated chewing gum (McNeil Products Ltd) |
| 41426 | NiQuitin 7mg patches (GlaxoSmithKline Consumer Healthcare) |
| 41474 | Nicorette 10mg patches (McNeil Products Ltd) |
| 41485 | NiQuitin 14mg patches (GlaxoSmithKline Consumer Healthcare) |
| 41493 | Nicorette Icy White 4mg medicated chewing gum (McNeil Products Ltd) |
| 41496 | Nicorette 500micrograms/dose nasal spray (McNeil Products Ltd) |
| 41505 | NiQuitin Clear 14mg patches (GlaxoSmithKline Consumer Healthcare) |
| 41507 | NiQuitin Clear 7mg patches (GlaxoSmithKline Consumer Healthcare) |
| 41753 | Nicorette Original 4mg medicated chewing gum (McNeil Products Ltd) |
| 41765 | Nicotinell Mint 2mg medicated chewing gum (Novartis Consumer Health UK Ltd) |
| 41778 | Nicorette Freshfruit 4mg medicated chewing gum (McNeil Products Ltd) |
| 41779 | Nicorette Icy White 2mg medicated chewing gum (McNeil Products Ltd) |
| 41801 | Nicorette Freshmint 2mg medicated chewing gum (McNeil Products Ltd) |
| 41802 | Nicorette 5mg patches (McNeil Products Ltd) |
| 41808 | Nicotinell Fruit 4mg medicated chewing gum (Novartis Consumer Health UK Ltd) |
| 41809 | Nicorette Mint 4mg medicated chewing gum (McNeil Products Ltd) |
| 41860 | Nicotine bitartrate 2mg Sublingual tablet |
| 41864 | Nicorette Freshfruit 2mg medicated chewing gum (McNeil Products Ltd) |
| 41879 | Nicotinell Liquorice 2mg medicated chewing gum (Novartis Consumer Health UK Ltd) |
| 41881 | Nicotinell Classic 2mg medicated chewing gum (Novartis Consumer Health UK Ltd) |
| 41909 | Nicotinell Mint 4mg medicated chewing gum (Novartis Consumer Health UK Ltd) |
| 41931 | Nicotinell Fruit 2mg medicated chewing gum (Novartis Consumer Health UK Ltd) |
| 42011 | Nicotinell Classic 4mg medicated chewing gum (Novartis Consumer Health UK Ltd) |
| 42016 | Nicorette Mint 2mg medicated chewing gum (McNeil Products Ltd) |
| 42047 | Nicotinell Liquorice 4mg medicated chewing gum (Novartis Consumer Health UK Ltd) |
| 42221 | Nicotine 4mg lozenges sugar free (Teva UK Ltd) |
| 44106 | NiQuitin Minis Cherry 1.5mg lozenges (GlaxoSmithKline Consumer Healthcare) |
| 45429 | Nicorette QuickMist 1mg/dose mouthspray (McNeil Products Ltd) |
| 45504 | Nicotine 1mg/dose oromucosal spray sugar free |
| 46588 | Nicotinell Icemint 2mg medicated chewing gum (Novartis Consumer Health UK Ltd) |
| 46592 | Nicorette 15mg Inhalator (McNeil Products Ltd) |
| 46701 | Nicotinell Icemint 4mg medicated chewing gum (Novartis Consumer Health UK Ltd) |
| 46717 | Nicotine 15mg inhalation cartridges with device |
| 48620 | Boots NicAssist 10mg Inhalator (The Boots Company Plc) |
| 49088 | NiQuitin Clear 21mg patches (Waymade Healthcare Plc) |
| 49305 | Nicorette Cools 2mg lozenges (McNeil Products Ltd) |
| 49319 | Nicorette Cools 4mg lozenges (McNeil Products Ltd) |
| 49607 | Boots NicAssist 15mg patches (The Boots Company Plc) |
| 50487 | NiQuitin Clear 14mg patches (Waymade Healthcare Plc) |
| 54102 | NiQuitin Pre-Quit Clear 21mg patches (GlaxoSmithKline Consumer Healthcare) |
| 54574 | Boots NicAssist Minty Fresh 4mg medicated chewing gum (The Boots Company Plc) |
| 55590 | Nicotine 11mg/24 hr Transdermal patch |
| 56552 | Boots NicAssist Minty Fresh 2mg medicated chewing gum (The Boots Company Plc) |
| 57417 | Nicorette 5mg patches (Waymade Healthcare Plc) |
| 58675 | Boots NicAssist 5mg patches (The Boots Company Plc) |
| 60236 | Boots NicAssist 10mg patches (The Boots Company Plc) |
| 61777 | Nicotinell Support Icemint 4mg medicated chewing gum (Novartis Consumer Health UK Ltd) |
| 62246 | Nicotinell Support Icemint 2mg medicated chewing gum (Novartis Consumer Health UK Ltd) |
| 6698 | Nicotinell 2mg lozenges (Novartis Consumer Health UK Ltd) |
| 38958 | Nicotinell 1mg lozenges (Novartis Consumer Health UK Ltd) |
| 45603 | Nicorette Freshmint 2mg lozenges (McNeil Products Ltd) |
| 42048 | Nicotine bitartrate 1mg lozenges sugar free |
| 42286 | Nicotine bitartrate 2mg lozenges sugar free |
| 5877 | Nicorette 2mg microtab (Pharmacia Ltd) |
| 41356 | Nicorette Microtab 2mg sublingual tablets (McNeil Products Ltd) |
| 11718 | Nicotine 2mg sublingual tablets sugar free |

**Outcome**

## Smoking status

Smoking status code list

| Medical code | Smoking status |
| --- | --- |
| 93 | current |
| 1822 | current |
| 1823 | current |
| 1878 | current |
| 2111 | current |
| 3568 | current |
| 10558 | current |
| 10742 | current |
| 12240 | current |
| 12941 | current |
| 12942 | current |
| 12943 | current |
| 12944 | current |
| 12945 | current |
| 12947 | current |
| 12951 | current |
| 12952 | current |
| 12958 | current |
| 12962 | current |
| 12964 | current |
| 12966 | current |
| 18573 | current |
| 18926 | current |
| 24529 | current |
| 30423 | current |
| 30762 | current |
| 31114 | current |
| 32687 | current |
| 34126 | current |
| 35055 | current |
| 38112 | current |
| 41042 | current |
| 41979 | current |
| 43433 | current |
| 46321 | current |
| 47273 | current |
| 49418 | current |
| 59866 | current |
| 62686 | current |
| 63299 | current |
| 63666 | current |
| 66409 | current |
| 68658 | current |
| 70746 | current |
| 91513 | current |
| 95610 | current |
| 97643 | current |
| 98137 | current |
| 98154 | current |
| 98283 | current |
| 98284 | current |
| 98347 | current |
| 100099 | current |
| 101338 | current |
| 101764 | current |
| 102361 | current |
| 103400 | current |
| 103507 | current |
| 103760 | current |
| 104310 | current |
| 106359 | current |
| 107504 | current |
| 107792 | current |
| 108835 | current |
| 16717 | current |
| 104185 | current |
| 104230 | current |
| 106391 | current |
| 104086 | current |
| 105572 | current |
| 106384 | current |
| 106385 | current |

**Covariates**

## Alcohol misuse

Alcohol misuse code list

| Medical code | Read code | Read term |
| --- | --- | --- |
| 669 | E250000 | Nondependent alcohol abuse, unspecified |
| 1399 | E23..12 | Alcohol problem drinking |
| 2081 | E23..11 | Alcoholism |
| 2082 | E01y000 | Alcohol withdrawal syndrome |
| 2083 | 8BA8.00 | Alcohol detoxification |
| 2084 | E23..00 | Alcohol dependence syndrome |
| 2925 | F375.00 | Alcoholic polyneuropathy |
| 3216 | J611.00 | Acute alcoholic hepatitis |
| 4500 | E011000 | Korsakov's alcoholic psychosis |
| 4506 | J153.00 | Alcoholic gastritis |
| 4743 | J612.00 | Alcoholic cirrhosis of liver |
| 4915 | G555.00 | Alcoholic cardiomyopathy |
| 5611 | Eu10.00 | [X]Mental and behavioural disorders due to use of alcohol |
| 5740 | E230.00 | Acute alcoholic intoxication in alcoholism |
| 5758 | Eu10212 | [X]Chronic alcoholism |
| 6169 | E23z.00 | Alcohol dependence syndrome NOS |
| 6467 | Eu10511 | [X]Alcoholic hallucinosis |
| 7123 | ZV11300 | [V]Personal history of alcoholism |
| 7602 | J617000 | Chronic alcoholic hepatitis |
| 7746 | E250.00 | Nondependent alcohol abuse |
| 7885 | J613.00 | Alcoholic liver damage unspecified |
| 7943 | J617.00 | Alcoholic hepatitis |
| 8030 | ZV6D600 | [V]Alcohol abuse counselling and surveillance |
| 8363 | G852300 | Oesophageal varices in alcoholic cirrhosis of the liver |
| 8388 | ZV57A00 | [V]Alcohol rehabilitation |
| 8430 | 1462 | H/O: alcoholism |
| 9489 | 9NN2.00 | Under care of community alcohol team |
| 9508 | Eu10011 | [X]Acute alcoholic drunkenness |
| 9849 | 8H7p.00 | Referral to community alcohol team |
| 10691 | J610.00 | Alcoholic fatty liver |
| 11106 | E011100 | Korsakov's alcoholic psychosis with peripheral neuritis |
| 11670 | Eu10611 | [X]Korsakov's psychosis, alcohol induced |
| 11740 | 9k1..00 | Alcohol misuse - enhanced services administration |
| 12353 | Eu10500 | [X]Mental & behav dis due to use alcohol: psychotic disorder |
| 12442 | 66e..00 | Alcohol disorder monitoring |
| 12554 | 8HHe.00 | Referral to community drug and alcohol team |
| 12974 | E250200 | Nondependent alcohol abuse, episodic |
| 12976 | 1369 | Suspect alcohol abuse - denied |
| 12982 | 136K.00 | Alcohol intake above recommended sensible limits |
| 16225 | E010.00 | Alcohol withdrawal delirium |
| 16237 | E01..00 | Alcoholic psychoses |
| 16587 | ZV11311 | [V]Problems related to lifestyle alcohol use |
| 17259 | Eu10411 | [X]Delirium tremens, alcohol induced |
| 17330 | J613000 | Alcoholic hepatic failure |
| 17607 | Eu10514 | [X]Alcoholic psychosis NOS |
| 18156 | 13Y8.00 | Alcoholics anonymous |
| 19494 | 136S.00 | Hazardous alcohol use |
| 20514 | Eu10300 | [X]Mental and behav dis due to use alcohol: withdrawal state |
| 20762 | E011.00 | Alcohol amnestic syndrome |
| 21624 | E230200 | Episodic acute alcoholic intoxication in alcoholism |
| 21650 | 8H35.00 | Admitted to alcohol detoxification centre |
| 21713 | J612000 | Alcoholic fibrosis and sclerosis of liver |
| 21879 | Eu10100 | [X]Mental and behav dis due to use of alcohol: harmful use |
| 23610 | E250100 | Nondependent alcohol abuse, continuous |
| 23945 | PK80.00 | Fetal alcohol syndrome |
| 24064 | E231100 | Continuous chronic alcoholism |
| 24485 | E231300 | Chronic alcoholism in remission |
| 24984 | J671000 | Alcohol-induced chronic pancreatitis |
| 25110 | E013.00 | Alcohol withdrawal hallucinosis |
| 26106 | E231200 | Episodic chronic alcoholism |
| 26323 | Eu10711 | [X]Alcoholic dementia NOS |
| 27342 | E012.11 | Alcoholic dementia NOS |
| 27670 | L255300 | Maternal care for (suspected) damage to fetus from alcohol |
| 28150 | E250z00 | Nondependent alcohol abuse NOS |
| 28780 | Eu10211 | [X]Alcohol addiction |
| 29691 | 8G32.00 | Aversion therapy - alcoholism |
| 30162 | Eu10513 | [X]Alcoholic paranoia |
| 30404 | E015.00 | Alcoholic paranoia |
| 30460 | Z4B1.00 | Alcoholism counselling |
| 30604 | F25B.00 | Alcohol-induced epilepsy |
| 30695 | 136T.00 | Harmful alcohol use |
| 31443 | E231.00 | Chronic alcoholism |
| 31569 | E250300 | Nondependent alcohol abuse in remission |
| 31742 | F394100 | Alcoholic myopathy |
| 32927 | Eu10800 | [X]Alcohol withdrawal-induced seizure |
| 32964 | 66e0.00 | Alcohol abuse monitoring |
| 33635 | E231z00 | Chronic alcoholism NOS |
| 33670 | E01y.00 | Other alcoholic psychosis |
| 33839 | F144000 | Cerebellar ataxia due to alcoholism |
| 35330 | 9k11.00 | Alcohol consumption counselling |
| 36296 | E230z00 | Acute alcoholic intoxication in alcoholism NOS |
| 36748 | F11x011 | Alcoholic encephalopathy |
| 37691 | Eu10712 | [X]Chronic alcoholic brain syndrome |
| 37946 | E012000 | Chronic alcoholic brain syndrome |
| 38061 | 1B1c.00 | Alcohol induced hallucinations |
| 39327 | Eu10200 | [X]Mental and behav dis due to use alcohol: dependence syndr |
| 39799 | Eu10600 | [X]Mental and behav dis due to use alcohol: amnesic syndrome |
| 40530 | E230000 | Acute alcoholic intoxication, unspecified, in alcoholism |
| 41920 | E011z00 | Alcohol amnestic syndrome NOS |
| 41983 | Z191.00 | Alcohol detoxification |
| 43193 | E231000 | Unspecified chronic alcoholism |
| 44299 | Eu10000 | [X]Mental & behav dis due to use alcohol: acute intoxication |
| 45169 | Eu10y00 | [X]Men & behav dis due to use alcohol: oth men & behav dis |
| 46677 | Z191100 | Alcohol withdrawal regime |
| 47123 | 9k14.00 | Alcohol counselling by other agencies |
| 47555 | F11x000 | Cerebral degeneration due to alcoholism |
| 54505 | E012.00 | Other alcoholic dementia |
| 56410 | 7P22100 | Delivery of rehabilitation for alcohol addiction |
| 56947 | E230100 | Continuous acute alcoholic intoxication in alcoholism |
| 57714 | E230.11 | Alcohol dependence with acute alcoholic intoxication |
| 57939 | E014.00 | Pathological alcohol intoxication |
| 59574 | E230300 | Acute alcoholic intoxication in remission, in alcoholism |
| 61383 | Z191200 | Planned reduction of alcohol consumption |
| 62000 | Eu10700 | [X]Men & behav dis due alcoh: resid & late-onset psychot dis |
| 63529 | 9k12.00 | Alcohol misuse - enhanced service completed |
| 64101 | Eu10400 | [X]Men & behav dis due alcohl: withdrawl state with delirium |
| 64389 | Eu10z00 | [X]Ment & behav dis due use alcohol: unsp ment & behav dis |
| 65754 | C150500 | Alcohol-induced pseudo-Cushing's syndrome |
| 65932 | Eu10512 | [X]Alcoholic jealousy |
| 66019 | L254.11 | Suspect fetal damage from maternal alcohol |
| 66699 | PK83.00 | Fetus and newborn affected by maternal use of alcohol |
| 67651 | E01z.00 | Alcoholic psychosis NOS |
| 68111 | E01yz00 | Other alcoholic psychosis NOS |
| 72757 | Q007100 | Fetus/neonate affected by placental/breast transfer alcohol |
| 84218 | 13ZY.00 | Disqualified from driving due to excess alcohol |
| 94553 | 8HkG.00 | Referral to specialist alcohol treatment service |
| 94670 | 136W.00 | Alcohol misuse |
| 95181 | Z191211 | Alcohol reduction programme |
| 96053 | 9k1A.00 | Brief intervention for excessive alcohol consumptn completed |
| 96054 | 9k1B.00 | Extended intervention for excessive alcohol consumptn complt |
| 96993 | 8HkJ.00 | Referral to alcohol brief intervention service |
| 97261 | 8IAF.00 | Brief intervention for excessive alcohol consumptn declined |
| 97680 | 8IAJ.00 | Declined referral to specialist alcohol treatment service |
| 97916 | Q007111 | Fetal alcohol syndrome |
| 102247 | 8IAt.00 | Extended interven for excessive alcohol consumption declined |
| 104611 | J670800 | Alcohol-induced acute pancreatitis |

## Anxiety, neurotic, stress-related and somatoform disorder (ICD-10 F40-48)

Anxiety, neurotic, stress-related and somatoform disorder code list

| Medical code | Read code | Read term |
| --- | --- | --- |
| 3407 | 1466 | H/O: anxiety state |
| 11764 | 146G.00 | H/O: agoraphobia |
| 19000 | 225J.00 | O/E - panic attack |
| 15811 | 285..00 | Neurotic condition, insight present |
| 5274 | 286..00 | Poor insight into neurotic condition |
| 100992 | 38Du.00 | Improving Access to Psychological Therapies pro phobia scale |
| 101323 | 38Du000 | IAPT phobia scale - Soc sit due fear embarrass mak fool self |
| 102106 | 38Du100 | IAPT phobia scale - Cert situ fear panic attak distres symp |
| 102133 | 38Du200 | IAPT phobia scale - Cert situ becse fear particulr obj activ |
| 63521 | 8G52.00 | Antiphobic therapy |
| 9125 | 8G94.00 | Anxiety management training |
| 28925 | 8HHp.00 | Referral for guided self-help for anxiety |
| 29937 | E131.00 | Acute hysterical psychosis |
| 7332 | E132.00 | Reactive confusion |
| 22117 | E13y000 | Psychogenic stupor |
| 9686 | E2...00 | Neurotic, personality and other nonpsychotic disorders |
| 5249 | E20..00 | Neurotic disorders |
| 636 | E200.00 | Anxiety states |
| 6939 | E200000 | Anxiety state unspecified |
| 4069 | E200100 | Panic disorder |
| 462 | E200111 | Panic attack |
| 4659 | E200200 | Generalised anxiety disorder |
| 655 | E200300 | Anxiety with depression |
| 1758 | E200400 | Chronic anxiety |
| 4634 | E200500 | Recurrent anxiety |
| 4534 | E200z00 | Anxiety state NOS |
| 2188 | E201.00 | Hysteria |
| 41572 | E201000 | Hysteria unspecified |
| 3438 | E201100 | Hysterical blindness |
| 44739 | E201200 | Hysterical deafness |
| 23598 | E201300 | Hysterical tremor |
| 34696 | E201400 | Hysterical paralysis |
| 16484 | E201500 | Hysterical seizures |
| 33702 | E201511 | Fit - hysterical |
| 15431 | E201600 | Other conversion disorder |
| 46399 | E201611 | Astasia - abasia, hysterical |
| 4143 | E201612 | Globus hystericus |
| 4269 | E201700 | Hysterical amnesia |
| 4775 | E201800 | Hysterical fugue |
| 23490 | E201A00 | Dissociative reaction unspecified |
| 24525 | E201C00 | Phantom pregnancy |
| 23354 | E201z00 | Hysteria NOS |
| 4105 | E201z11 | Aphonia - hysterical |
| 24638 | E201z12 | Ataxia - hysterical |
| 40066 | E201z13 | Ganser's syndrome - hysterical |
| 1907 | E202.00 | Phobic disorders |
| 16638 | E202.11 | Social phobic disorders |
| 9944 | E202.12 | Phobic anxiety |
| 2300 | E202000 | Phobia unspecified |
| 3076 | E202100 | Agoraphobia with panic attacks |
| 12838 | E202200 | Agoraphobia without mention of panic attacks |
| 16199 | E202300 | Social phobia, fear of eating in public |
| 31957 | E202400 | Social phobia, fear of public speaking |
| 18603 | E202500 | Social phobia, fear of public washing |
| 28106 | E202600 | Acrophobia |
| 28938 | E202700 | Animal phobia |
| 1723 | E202800 | Claustrophobia |
| 31672 | E202900 | Fear of crowds |
| 4167 | E202A00 | Fear of flying |
| 1510 | E202B00 | Cancer phobia |
| 2366 | E202C00 | Dental phobia |
| 10390 | E202D00 | Fear of death |
| 6071 | E202E00 | Fear of pregnancy |
| 14729 | E202z00 | Phobic disorder NOS |
| 3208 | E203.00 | Obsessive-compulsive disorders |
| 47365 | E203.11 | Anancastic neurosis |
| 5678 | E203000 | Compulsive neurosis |
| 2030 | E203100 | Obsessional neurosis |
| 15566 | E203z00 | Obsessive-compulsive disorder NOS |
| 3361 | E205.00 | Neurasthenia - nervous debility |
| 5305 | E206.00 | Depersonalisation syndrome |
| 966 | E207.00 | Hypochondriasis |
| 42000 | E20y.00 | Other neurotic disorders |
| 15321 | E20y000 | Somatization disorder |
| 56941 | E20y011 | Briquet's disorder |
| 3685 | E20y100 | Writer's cramp neurosis |
| 39518 | E20y200 | Other occupational neurosis |
| 72171 | E20y300 | Psychasthenic neurosis |
| 43050 | E20yz00 | Other neurotic disorder NOS |
| 14780 | E20z.00 | Neurotic disorder NOS |
| 21253 | E227700 | Psychogenic dyspareunia |
| 4199 | E26..00 | Physiological malfunction arising from mental factors |
| 44212 | E260.00 | Psychogenic musculoskeletal symptoms |
| 48561 | E260000 | Psychogenic paralysis |
| 56800 | E260100 | Psychogenic torticollis |
| 15035 | E260z00 | Psychogenic musculoskeletal symptoms NOS |
| 38134 | E261.00 | Psychogenic respiratory symptoms |
| 32034 | E261000 | Psychogenic air hunger |
| 15483 | E261100 | Psychogenic cough |
| 47809 | E261200 | Psychogenic hiccough |
| 20053 | E261300 | Psychogenic hyperventilation |
| 23413 | E261400 | Psychogenic yawning |
| 41615 | E261500 | Psychogenic aphonia |
| 34664 | E261z00 | Psychogenic respiratory symptom NOS |
| 29448 | E262.00 | Psychogenic cardiovascular symptoms |
| 15292 | E262000 | Cardiac neurosis |
| 15284 | E262200 | Neurocirculatory asthenia |
| 30961 | E262300 | Psychogenic cardiovascular disorder |
| 15034 | E262z00 | Psychogenic cardiovascular symptom NOS |
| 29461 | E263.00 | Psychogenic skin symptoms |
| 15959 | E263000 | Psychogenic pruritus |
| 15224 | E263z00 | Psychogenic skin symptoms NOS |
| 31422 | E264.00 | Psychogenic gastrointestinal tract symptoms |
| 37695 | E264.11 | Globus abdominalis |
| 4963 | E264000 | Psychogenic aerophagy |
| 23774 | E264011 | Air swallowing - excessive |
| 2871 | E264200 | Cyclical vomiting - psychogenic |
| 15371 | E264300 | Psychogenic diarrhoea |
| 10158 | E264311 | Spurious diarrhoea |
| 3869 | E264400 | Psychogenic dyspepsia |
| 15939 | E264500 | Psychogenic constipation |
| 71437 | E264z00 | Psychogenic gastrointestinal tract symptom NOS |
| 68379 | E265.00 | Psychogenic genitourinary tract symptoms |
| 20109 | E265100 | Psychogenic vaginismus |
| 44547 | E265200 | Psychogenic dysmenorrhea |
| 55781 | E265300 | Psychogenic dysuria |
| 73547 | E265z00 | Psychogenic genitourinary tract symptom NOS |
| 89237 | E267.00 | Psychogenic symptom of special sense organ |
| 62400 | E26y.00 | Other psychogenic malfunction |
| 10001 | E26y000 | Bruxism (teeth grinding) |
| 96391 | E26yz00 | Other psychogenic malfunction NOS |
| 5067 | E26z.00 | Psychosomatic disorder NOS |
| 52481 | E27..00 | Psychogenic syndromes NEC |
| 65173 | E275300 | Psychogenic rumination |
| 26395 | E275700 | Psychogenic polydipsia |
| 45362 | E275711 | Compulsive water drinking |
| 40311 | E278.00 | Psychalgia |
| 53766 | E278000 | Psychogenic pain unspecified |
| 45205 | E278200 | Psychogenic backache |
| 54373 | E278z00 | Psychalgia NOS |
| 71355 | E27z.00 | Other and unspecified psychogenic syndrome NEC |
| 24076 | E27zz00 | Psychogenic syndromes NOS |
| 276 | E28..00 | Acute reaction to stress |
| 43550 | E28..11 | Combat fatigue |
| 11940 | E280.00 | Acute panic state due to acute stress reaction |
| 42737 | E281.00 | Acute fugue state due to acute stress reaction |
| 15551 | E282.00 | Acute stupor state due to acute stress reaction |
| 38640 | E283.00 | Other acute stress reactions |
| 24847 | E283100 | Acute posttrauma stress state |
| 29707 | E283z00 | Other acute stress reaction NOS |
| 23869 | E284.00 | Stress reaction causing mixed disturbance of emotion/conduct |
| 26138 | E28z.00 | Acute stress reaction NOS |
| 20802 | E28z.12 | Flying phobia |
| 2826 | E29..00 | Adjustment reaction |
| 2775 | E290000 | Grief reaction |

## Autism (ICD-10 Chapters F84.0-84.1, F84.4-84.9)

Prevalence of autism was rare in the CPRD (e.g. ≤1% prevalence) and therefore was included in the other mental disorders category.

Autism code list

| Med code | Read code | Read term |
| --- | --- | --- |
| 44327 | Eu84z00 | [X]Pervasive developmental disorder, unspecified |
| 7226 | Eu84.00 | [X]Pervasive developmental disorders |
| 47948 | Eu84y00 | [X]Other pervasive developmental disorders |
| 69016 | E140100 | Residual infantile autism |
| 24044 | Eu84100 | [X]Atypical autism |
| 26343 | 1J9..00 | Suspected autism |
| 1276 | E140.12 | Autism |
| 7302 | E140.13 | Childhood autism |
| 50337 | Eu84012 | [X]Infantile autism |
| 22098 | E140.00 | Infantile autism |
| 3637 | Eu84000 | [X]Childhood autism |
| 36662 | E140z00 | Infantile autism NOS |
| 63251 | E140000 | Active infantile autism |
| 42941 | Eu84z11 | [X]Autistic spectrum disorder |
| 9982 | Eu84011 | [X]Autistic disorder |
| 51375 | Eu84511 | [X]Autistic psychopathy |
| 34174 | Eu84112 | [X]Mental retardation with autistic features |
| 2950 | Eu84500 | [X]Asperger's syndrome |

## Bipolar disorder diagnosis (ICD-10 Chapters F10-31

Bipolar disorder code list

| Medical code | Read code | Read term |
| --- | --- | --- |
| 11548 | 146D.00 | H/O: manic depressive disorder |
| 85102 | 212V.00 | Bipolar affective disorder resolved |
| 8567 | E11..11 | Bipolar psychoses |
| 26161 | E11..13 | Manic psychoses |
| 37070 | E110.00 | Manic disorder, single episode |
| 18909 | E110.11 | Hypomanic psychoses |
| 20110 | E110000 | Single manic episode, unspecified |
| 14728 | E110100 | Single manic episode, mild |
| 24640 | E110200 | Single manic episode, moderate |
| 43093 | E110300 | Single manic episode, severe without mention of psychosis |
| 50218 | E110400 | Single manic episode, severe, with psychosis |
| 109485 | E110500 | Single manic episode in partial or unspecified remission |
| 70000 | E110600 | Single manic episode in full remission |
| 36611 | E110z00 | Manic disorder, single episode NOS |
| 26227 | E111.00 | Recurrent manic episodes |
| 19967 | E111000 | Recurrent manic episodes, unspecified |
| 46425 | E111100 | Recurrent manic episodes, mild |
| 27739 | E111200 | Recurrent manic episodes, moderate |
| 65811 | E111300 | Recurrent manic episodes, severe without mention psychosis |
| 32295 | E111400 | Recurrent manic episodes, severe, with psychosis |
| 58863 | E111500 | Recurrent manic episodes, partial or unspecified remission |
| 37178 | E111600 | Recurrent manic episodes, in full remission |
| 46415 | E111z00 | Recurrent manic episode NOS |
| 3702 | E114.00 | Bipolar affective disorder, currently manic |
| 17385 | E114.11 | Manic-depressive - now manic |
| 35738 | E114000 | Bipolar affective disorder, currently manic, unspecified |
| 36126 | E114100 | Bipolar affective disorder, currently manic, mild |
| 46434 | E114200 | Bipolar affective disorder, currently manic, moderate |
| 16347 | E114300 | Bipolar affect disord, currently manic, severe, no psychosis |
| 55829 | E114400 | Bipolar affect disord, currently manic,severe with psychosis |
| 59011 | E114500 | Bipolar affect disord,currently manic, part/unspec remission |
| 63784 | E114600 | Bipolar affective disorder, currently manic, full remission |
| 57605 | E114z00 | Bipolar affective disorder, currently manic, NOS |
| 4677 | E115.00 | Bipolar affective disorder, currently depressed |
| 12831 | E115.11 | Manic-depressive - now depressed |
| 15923 | E115000 | Bipolar affective disorder, currently depressed, unspecified |
| 35734 | E115100 | Bipolar affective disorder, currently depressed, mild |
| 27890 | E115200 | Bipolar affective disorder, currently depressed, moderate |
| 35607 | E115300 | Bipolar affect disord, now depressed, severe, no psychosis |
| 63701 | E115400 | Bipolar affect disord, now depressed, severe with psychosis |
| 72026 | E115500 | Bipolar affect disord, now depressed, part/unspec remission |
| 57465 | E115600 | Bipolar affective disorder, now depressed, in full remission |
| 37296 | E115z00 | Bipolar affective disorder, currently depressed, NOS |
| 31316 | E116.00 | Mixed bipolar affective disorder |
| 31535 | E116000 | Mixed bipolar affective disorder, unspecified |
| 24689 | E116100 | Mixed bipolar affective disorder, mild |
| 63150 | E116200 | Mixed bipolar affective disorder, moderate |
| 63284 | E116300 | Mixed bipolar affective disorder, severe, without psychosis |
| 54195 | E116400 | Mixed bipolar affective disorder, severe, with psychosis |
| 63651 | E116500 | Mixed bipolar affective disorder, partial/unspec remission |
| 55064 | E116600 | Mixed bipolar affective disorder, in full remission |
| 63583 | E116z00 | Mixed bipolar affective disorder, NOS |
| 14784 | E117.00 | Unspecified bipolar affective disorder |
| 49763 | E117000 | Unspecified bipolar affective disorder, unspecified |
| 63698 | E117100 | Unspecified bipolar affective disorder, mild |
| 68647 | E117200 | Unspecified bipolar affective disorder, moderate |
| 73423 | E117300 | Unspecified bipolar affective disorder, severe, no psychosis |
| 68326 | E117400 | Unspecified bipolar affective disorder,severe with psychosis |
| 70721 | E117500 | Unspecified bipolar affect disord, partial/unspec remission |
| 24230 | E117600 | Unspecified bipolar affective disorder, in full remission |
| 27986 | E117z00 | Unspecified bipolar affective disorder, NOS |
| 60178 | E11y.00 | Other and unspecified manic-depressive psychoses |
| 11596 | E11y000 | Unspecified manic-depressive psychoses |
| 70925 | E11y100 | Atypical manic disorder |
| 70399 | E11y300 | Other mixed manic-depressive psychoses |
| 33426 | E11yz00 | Other and unspecified manic-depressive psychoses NOS |
| 12173 | Eu30.00 | [X]Manic episode |
| 9521 | Eu30.11 | [X]Bipolar disorder, single manic episode |
| 2741 | Eu30000 | [X]Hypomania |
| 13024 | Eu30100 | [X]Mania without psychotic symptoms |
| 21065 | Eu30200 | [X]Mania with psychotic symptoms |
| 37102 | Eu30211 | [X]Mania with mood-congruent psychotic symptoms |
| 48632 | Eu30212 | [X]Mania with mood-incongruent psychotic symptoms |
| 32088 | Eu30y00 | [X]Other manic episodes |
| 44513 | Eu30z00 | [X]Manic episode, unspecified |
| 4678 | Eu30z11 | [X]Mania NOS |
| 6874 | Eu31.00 | [X]Bipolar affective disorder |
| 1531 | Eu31.11 | [X]Manic-depressive illness |
| 6710 | Eu31.12 | [X]Manic-depressive psychosis |
| 66153 | Eu31.13 | [X]Manic-depressive reaction |
| 16808 | Eu31000 | [X]Bipolar affective disorder, current episode hypomanic |
| 26299 | Eu31100 | [X]Bipolar affect disorder cur epi manic wout psychotic symp |
| 28277 | Eu31200 | [X]Bipolar affect disorder cur epi manic with psychotic symp |
| 16562 | Eu31300 | [X]Bipolar affect disorder cur epi mild or moderate depressn |
| 23713 | Eu31400 | [X]Bipol aff disord, curr epis sev depress, no psychot symp |
| 4732 | Eu31500 | [X]Bipolar affect dis cur epi severe depres with psyc symp |
| 44693 | Eu31600 | [X]Bipolar affective disorder, current episode mixed |
| 27584 | Eu31700 | [X]Bipolar affective disorder, currently in remission |
| 104065 | Eu31800 | [X]Bipolar affective disorder type I |
| 103915 | Eu31900 | [X]Bipolar affective disorder type II |
| 104051 | Eu31911 | [X]Bipolar II disorder |
| 53840 | Eu31y00 | [X]Other bipolar affective disorders |
| 73924 | Eu31y11 | [X]Bipolar II disorder |
| 51032 | Eu31y12 | [X]Recurrent manic episodes |
| 33751 | Eu31z00 | [X]Bipolar affective disorder, unspecified |
| 23963 | ZV11111 | [V]Personal history of manic-depressive psychosis |
| 22080 | ZV11112 | [V]Personal history of manic-depressive psychosis |

## Dementia diagnosis (ICD-10 Chapters F00-02)

Prevalence of dementia was rare in the CPRD (e.g. ≤1% prevalence) and therefore was included in the other mental disorders category.

Dementia code list

| Medical code | Read code | Read term |
| --- | --- | --- |
| 26270 | Eu02500 | [X]Lewy body dementia |
| 44674 | E002.00 | Senile dementia with depressive or paranoid features |
| 19393 | Eu01z00 | [X]Vascular dementia, unspecified |
| 4693 | Eu02z00 | [X] Unspecified dementia |
| 25704 | Eu00011 | [X]Presenile dementia,Alzheimer's type |
| 19477 | E004.00 | Arteriosclerotic dementia |
| 55313 | Eu01y00 | [X]Other vascular dementia |
| 30032 | E001200 | Presenile dementia with paranoia |
| 18386 | E002000 | Senile dementia with paranoia |
| 56912 | E004100 | Arteriosclerotic dementia with delirium |
| 33707 | E00..00 | Senile and presenile organic psychotic conditions |
| 4357 | Eu02z14 | [X] Senile dementia NOS |
| 12710 | 6AB..00 | Dementia annual review |
| 41089 | E002z00 | Senile dementia with depressive or paranoid features NOS |
| 9509 | Eu02300 | [X]Dementia in Parkinson's disease |
| 15165 | E001.00 | Presenile dementia |
| 49513 | E001100 | Presenile dementia with delirium |
| 42602 | E001000 | Uncomplicated presenile dementia |
| 7572 | F116.00 | Lewy body disease |
| 30706 | Eu00200 | [X]Dementia in Alzheimer's dis, atypical or mixed type |
| 1916 | E00..11 | Senile dementia |
| 55467 | E004200 | Arteriosclerotic dementia with paranoia |
| 9565 | Eu01.11 | [X]Arteriosclerotic dementia |
| 8934 | Eu01200 | [X]Subcortical vascular dementia |
| 31016 | Eu01300 | [X]Mixed cortical and subcortical vascular dementia |
| 43089 | E004000 | Uncomplicated arteriosclerotic dementia |
| 11175 | Eu01100 | [X]Multi-infarct dementia |
| 42279 | E004z00 | Arteriosclerotic dementia NOS |
| 29386 | Eu00z00 | [X]Dementia in Alzheimer's disease, unspecified |
| 38438 | E001z00 | Presenile dementia NOS |
| 8634 | E004.11 | Multi infarct dementia |
| 1917 | F110.00 | Alzheimer's disease |
| 61528 | Eu00013 | [X]Alzheimer's disease type 2 |
| 1350 | E00..12 | Senile/presenile dementia |
| 38678 | Eu00100 | [X]Dementia in Alzheimer's disease with late onset |
| 27677 | E001300 | Presenile dementia with depression |
| 46762 | Eu00111 | [X]Alzheimer's disease type 1 |
| 11379 | Eu00112 | [X]Senile dementia,Alzheimer's type |
| 21887 | E002100 | Senile dementia with depression |
| 2882 | E00z.00 | Senile or presenile psychoses NOS |
| 25386 | E041.00 | Dementia in conditions EC |
| 49263 | Eu00000 | [X]Dementia in Alzheimer's disease with early onset |
| 59122 | Fyu3000 | [X]Other Alzheimer's disease |
| 30641 | 9hD0.00 | Excepted from dementia quality indicators: Patient unsuitabl |
| 34944 | Eu02z13 | [X] Primary degenerative dementia NOS |
| 40805 | 9hD1.00 | Excepted from dementia quality indicators: Informed dissent |
| 37015 | E003.00 | Senile dementia with delirium |
| 6578 | Eu01.00 | [X]Vascular dementia |
| 7323 | E000.00 | Uncomplicated senile dementia |
| 55838 | Eu01111 | [X]Predominantly cortical dementia |
| 29512 | F112.00 | Senile degeneration of brain |
| 12621 | Eu02.00 | [X]Dementia in other diseases classified elsewhere |
| 7664 | Eu00.00 | [X]Dementia in Alzheimer's disease |
| 60059 | Eu00012 | [X]Primary degen dementia, Alzheimer's type, presenile onset |
| 15249 | E00y.00 | Other senile and presenile organic psychoses |
| 43346 | Eu00113 | [X]Primary degen dementia of Alzheimer's type, senile onset |
| 53446 | Eu04100 | [X]Delirium superimposed on dementia |
| 43292 | E004300 | Arteriosclerotic dementia with depression |
| 32057 | F110100 | Alzheimer's disease with late onset |
| 8195 | Eu00z11 | [X]Alzheimer's dementia unspec |
| 64267 | Eu02y00 | [X]Dementia in other specified diseases classif elsewhere |
| 46488 | Eu01000 | [X]Vascular dementia of acute onset |
| 27759 | Eu02z16 | [X] Senile dementia, depressed or paranoid type |
| 16797 | F110000 | Alzheimer's disease with early onset |
| 48501 | Eu02z11 | [X] Presenile dementia NOS |
| 104155 | 1JA2.00 | Suspected dementia |
| 109047 | 8BPa.00 | Antipsychotic drug therapy for dementia |
| 106311 | 8CMZ.00 | Dementia care plan |
| 103445 | 8Hla.00 | Referral to dementia care advisor |
| 106627 | 8T05.00 | Referral to dementia service |
| 44341 | 9hD..00 | Exception reporting: dementia quality indicators |
| 28402 | Eu02000 | [X]Dementia in Pick's disease |
| 54106 | Eu02100 | [X]Dementia in Creutzfeldt-Jakob disease |
| 37014 | Eu02200 | [X]Dementia in Huntington's disease |
| 41185 | Eu02400 | [X]Dementia in human immunodef virus [HIV] disease |
| 5931 | 1461 | H/O: dementia |

## Depression diagnosis (ICD-10 Chapters F32-39)

Depression code list

| Medical code | Read code | Read term |
| --- | --- | --- |
| 2716 | 1465 | H/O: depression |
| 9796 | 1B1U.00 | Symptoms of depression |
| 10438 | 1B1U.11 | Depressive symptoms |
| 100977 | 1JJ..00 | Suspected depression |
| 19439 | 212S.00 | Depression resolved |
| 2923 | 62T1.00 | Puerperal depression |
| 18702 | 6G00.00 | Postnatal depression counselling |
| 44848 | 8BK0.00 | Depression management programme |
| 30483 | 8CAa.00 | Patient given advice about management of depression |
| 32841 | 8HHq.00 | Referral for guided self-help for depression |
| 12399 | 9H90.00 | Depression annual review |
| 12122 | 9H91.00 | Depression medication review |
| 30405 | 9H92.00 | Depression interim review |
| 42931 | 9HA0.00 | On depression register |
| 44936 | 9HA1.00 | Removed from depression register |
| 51258 | 9Ov..00 | Depression monitoring administration |
| 71009 | 9Ov0.00 | Depression monitoring first letter |
| 72966 | 9Ov1.00 | Depression monitoring second letter |
| 91105 | 9Ov2.00 | Depression monitoring third letter |
| 88644 | 9Ov3.00 | Depression monitoring verbal invite |
| 85852 | 9Ov4.00 | Depression monitoring telephone invite |
| 48970 | 9hC..00 | Exception reporting: depression quality indicators |
| 28970 | 9hC0.00 | Excepted from depression quality indicators: Patient unsuita |
| 43239 | 9hC1.00 | Excepted from depression quality indicators: Informed dissen |
| 30583 | 9k4..00 | Depression - enhanced services administration |
| 65435 | 9k40.00 | Depression - enhanced service completed |
| 96995 | 9kQ..00 | On full dose long term treatment depression - enh serv admin |
| 14656 | E11..00 | Affective psychoses |
| 2560 | E11..12 | Depressive psychoses |
| 10610 | E112.00 | Single major depressive episode |
| 5879 | E112.11 | Agitated depression |
| 6546 | E112.12 | Endogenous depression first episode |
| 6950 | E112.13 | Endogenous depression first episode |
| 595 | E112.14 | Endogenous depression |
| 34390 | E112000 | Single major depressive episode, unspecified |
| 16506 | E112100 | Single major depressive episode, mild |
| 15155 | E112200 | Single major depressive episode, moderate |
| 15219 | E112300 | Single major depressive episode, severe, without psychosis |
| 32159 | E112400 | Single major depressive episode, severe, with psychosis |
| 43324 | E112500 | Single major depressive episode, partial or unspec remission |
| 57409 | E112600 | Single major depressive episode, in full remission |
| 7011 | E112z00 | Single major depressive episode NOS |
| 15099 | E113.00 | Recurrent major depressive episode |
| 6932 | E113.11 | Endogenous depression - recurrent |
| 35671 | E113000 | Recurrent major depressive episodes, unspecified |
| 29342 | E113100 | Recurrent major depressive episodes, mild |
| 14709 | E113200 | Recurrent major depressive episodes, moderate |
| 25697 | E113300 | Recurrent major depressive episodes, severe, no psychosis |
| 24171 | E113400 | Recurrent major depressive episodes, severe, with psychosis |
| 56273 | E113500 | Recurrent major depressive episodes,partial/unspec remission |
| 55384 | E113600 | Recurrent major depressive episodes, in full remission |
| 6482 | E113700 | Recurrent depression |
| 25563 | E113z00 | Recurrent major depressive episode NOS |
| 10825 | E118.00 | Seasonal affective disorder |
| 27491 | E11y200 | Atypical depressive disorder |
| 9183 | E11z200 | Masked depression |
| 8478 | E130.00 | Reactive depressive psychosis |
| 17770 | E130.11 | Psychotic reactive depression |
| 1055 | E135.00 | Agitated depression |
| 1131 | E204.00 | Neurotic depression reactive type |
| 2639 | E204.11 | Postnatal depression |
| 12707 | E211300 | Cyclothymic personality disorder |
| 1533 | E290.00 | Brief depressive reaction |
| 36246 | E290z00 | Brief depressive reaction NOS |
| 16632 | E291.00 | Prolonged depressive reaction |
| 324 | E2B..00 | Depressive disorder NEC |
| 4323 | E2B1.00 | Chronic depression |
| 5726 | Eu3..00 | [X]Mood - affective disorders |
| 4639 | Eu32.00 | [X]Depressive episode |
| 9055 | Eu32.11 | [X]Single episode of depressive reaction |
| 18510 | Eu32.12 | [X]Single episode of psychogenic depression |
| 7604 | Eu32.13 | [X]Single episode of reactive depression |
| 11717 | Eu32000 | [X]Mild depressive episode |
| 9211 | Eu32100 | [X]Moderate depressive episode |
| 9667 | Eu32200 | [X]Severe depressive episode without psychotic symptoms |
| 41989 | Eu32211 | [X]Single episode agitated depressn w'out psychotic symptoms |
| 22806 | Eu32212 | [X]Single episode major depression w'out psychotic symptoms |
| 59386 | Eu32213 | [X]Single episode vital depression w'out psychotic symptoms |
| 12099 | Eu32300 | [X]Severe depressive episode with psychotic symptoms |
| 24117 | Eu32311 | [X]Single episode of major depression and psychotic symptoms |
| 52678 | Eu32312 | [X]Single episode of psychogenic depressive psychosis |
| 24112 | Eu32313 | [X]Single episode of psychotic depression |
| 28863 | Eu32314 | [X]Single episode of reactive depressive psychosis |
| 10667 | Eu32400 | [X]Mild depression |
| 98346 | Eu32500 | [X]Major depression, mild |
| 98252 | Eu32600 | [X]Major depression, moderately severe |
| 98414 | Eu32700 | [X]Major depression, severe without psychotic symptoms |
| 98417 | Eu32800 | [X]Major depression, severe with psychotic symptoms |
| 101054 | Eu32900 | [X]Single major depr ep, severe with psych, psych in remiss |
| 101153 | Eu32A00 | [X]Recurr major depr ep, severe with psych, psych in remiss |
| 103677 | Eu32B00 | [X]Antenatal depression |
| 6854 | Eu32y00 | [X]Other depressive episodes |
| 10720 | Eu32y11 | [X]Atypical depression |
| 56609 | Eu32y12 | [X]Single episode of masked depression NOS |
| 2970 | Eu32z00 | [X]Depressive episode, unspecified |
| 543 | Eu32z11 | [X]Depression NOS |
| 3291 | Eu32z12 | [X]Depressive disorder NOS |
| 28248 | Eu32z13 | [X]Prolonged single episode of reactive depression |
| 5987 | Eu32z14 | [X] Reactive depression NOS |
| 3292 | Eu33.00 | [X]Recurrent depressive disorder |
| 8851 | Eu33.11 | [X]Recurrent episodes of depressive reaction |
| 19696 | Eu33.12 | [X]Recurrent episodes of psychogenic depression |
| 8902 | Eu33.13 | [X]Recurrent episodes of reactive depression |
| 28756 | Eu33.14 | [X]Seasonal depressive disorder |
| 8826 | Eu33.15 | [X]SAD - Seasonal affective disorder |
| 29784 | Eu33000 | [X]Recurrent depressive disorder, current episode mild |
| 29520 | Eu33100 | [X]Recurrent depressive disorder, current episode moderate |
| 33469 | Eu33200 | [X]Recurr depress disorder cur epi severe without psyc sympt |
| 11329 | Eu33211 | [X]Endogenous depression without psychotic symptoms |
| 11252 | Eu33212 | [X]Major depression, recurrent without psychotic symptoms |
| 29451 | Eu33213 | [X]Manic-depress psychosis,depressd,no psychotic symptoms |
| 73991 | Eu33214 | [X]Vital depression, recurrent without psychotic symptoms |
| 47009 | Eu33300 | [X]Recurrent depress disorder cur epi severe with psyc symp |
| 23731 | Eu33311 | [X]Endogenous depression with psychotic symptoms |
| 28677 | Eu33312 | [X]Manic-depress psychosis,depressed type+psychotic symptoms |
| 32941 | Eu33313 | [X]Recurr severe episodes/major depression+psychotic symptom |
| 31757 | Eu33314 | [X]Recurr severe episodes/psychogenic depressive psychosis |
| 16861 | Eu33315 | [X]Recurrent severe episodes of psychotic depression |
| 37764 | Eu33316 | [X]Recurrent severe episodes/reactive depressive psychosis |
| 22116 | Eu33400 | [X]Recurrent depressive disorder, currently in remission |
| 47731 | Eu33y00 | [X]Other recurrent depressive disorders |
| 44300 | Eu33z00 | [X]Recurrent depressive disorder, unspecified |
| 36616 | Eu33z11 | [X]Monopolar depression NOS |
| 42857 | Eu34.00 | [X]Persistent mood affective disorders |
| 21540 | Eu34000 | [X]Cyclothymia |
| 26839 | Eu34011 | [X]Affective personality disorder |
| 54848 | Eu34012 | [X]Cycloid personality |
| 23854 | Eu34013 | [X]Cyclothymic personality |
| 7953 | Eu34100 | [X]Dysthymia |
| 8584 | Eu34111 | [X]Depressive neurosis |
| 10290 | Eu34112 | [X]Depressive personality disorder |
| 7737 | Eu34113 | [X]Neurotic depression |
| 15220 | Eu34114 | [X]Persistant anxiety depression |
| 50243 | Eu34y00 | [X]Other persistent mood affective disorders |
| 39767 | Eu34z00 | [X]Persistent mood affective disorder, unspecified |
| 28008 | Eu3y.00 | [X]Other mood affective disorders |
| 50998 | Eu3y000 | [X]Other single mood affective disorders |
| 30688 | Eu3y011 | [X]Mixed affective episode |
| 29921 | Eu3y100 | [X]Other recurrent mood affective disorders |
| 19054 | Eu3y111 | [X]Recurrent brief depressive episodes |
| 100211 | Eu3y200 | [X]Premenstrual dysphoric disorder |
| 29579 | Eu3yy00 | [X]Other specified mood affective disorders |
| 37090 | Eu3z.00 | [X]Unspecified mood affective disorder |
| 31633 | Eu3z.11 | [X]Affective psychosis NOS |
| 13307 | Eu53011 | [X]Postnatal depression NOS |
| 4979 | Eu53012 | [X]Postpartum depression NOS |
| 4876 | ZV79000 | [V]Screening for depression |
| 13307 | Eu53011 | [X]Postnatal depression NOS |
| 4979 | Eu53012 | [X]Postpartum depression NOS |
| 4876 | ZV79000 | [V]Screening for depression |

## Drug misuse

Drug misuse code list

| Medical code | Read code | Read term |
| --- | --- | --- |
| 101481 | U200400 | [X]Intent self pois nonopioid analgesic in street/highway |
| 103991 | Eu11600 | [X]Mental and behav dis due to use opioids: amnesic syndrome |
| 106028 | U200600 | [X]Int self pois nonopioid analgesic indust/construct area |
| 108575 | SyuFB00 | [X]Poisoning by other opioids |
| 107415 | 46QM000 | Urine cannabinoid positive |
| 107782 | 46QM100 | Urine cannabinoid negative |
| 103241 | 9k51.11 | Shared care drug misuse treatment |
| 103725 | 1V37.00 | Drug injecting equipment hygiene |
| 103726 | 1V35.00 | Shares drug equipment |
| 103844 | 1V0B.00 | Sniffs drugs |
| 105999 | 1V08.00 | Smokes drugs in cigarette form |
| 106290 | 9NdN.00 | Declined consent for notification of drug misuse |
| 106705 | 1V3A.00 | Does not share drug injection equipment |
| 106802 | 9s...00 | Drug misuse clinic administration |
| 106999 | 1V2..00 | Frequency of drug misuse |
| 106903 | 1P62.00 | Abnormal craving for drugs |
| 107355 | 9N1yJ00 | Seen in drug misuse clinic |
| 107593 | 1V09.00 | Smokes drugs through a pipe |
| 102582 | Eu1A200 | [X]Mental behav disorders due use crack cocaine: depend synd |
| 102475 | Eu1A500 | [X]Mental behav disord due crack cocaine: psychotic disorder |
| 101738 | Eu1A300 | [X]Mental behav disord due crack cocaine: withdrawal state |
| 91577 | Eu1Az00 | [X]Ment behav dis due crack cocaine: unsp ment and behav dis |
| 107854 | 46QA100 | Urine cocaine positive |
| 108323 | 46QA000 | Urine cocaine negative |
| 100178 | 8Hq..00 | Admission to substance misuse detoxification centre |
| 100935 | 1V0E.00 | Health problem secondary to drug misuse |
| 3565 | 1J1..00 | Suspected drug abuse |
| 6107 | 1J10.00 | Suspected abuse soft drugs |
| 7234 | 1J11.00 | Suspected abuse hard drugs |
| 16256 | E02..00 | Drug psychoses |
| 3844 | E020.00 | Drug withdrawal syndrome |
| 45997 | E021.00 | Drug-induced paranoia or hallucinatory states |
| 12628 | E021000 | Drug-induced paranoid state |
| 20026 | E021100 | Drug-induced hallucinosis |
| 26481 | E021z00 | Drug-induced paranoia or hallucinatory state NOS |
| 15876 | E022.00 | Pathological drug intoxication |
| 51135 | E02y.00 | Other drug psychoses |
| 29783 | E02y000 | Drug-induced delirium |
| 62132 | E02y100 | Drug-induced dementia |
| 46244 | E02y300 | Drug-induced depressive state |
| 22103 | E02y400 | Drug-induced personality disorder |
| 28767 | E02yz00 | Other drug psychoses NOS |
| 26002 | E02z.00 | Drug psychosis NOS |
| 5105 | E24..00 | Drug dependence |
| 3519 | E24..11 | Drug addiction |
| 16243 | E240.00 | Opioid type drug dependence |
| 689 | E240.11 | Heroin dependence |
| 16374 | E240.12 | Methadone dependence |
| 22059 | E240.13 | Morphine dependence |
| 32804 | E240.14 | Opium dependence |
| 38034 | E240000 | Unspecified opioid dependence |
| 43075 | E240100 | Continuous opioid dependence |
| 20962 | E240200 | Episodic opioid dependence |
| 27960 | E240300 | Opioid dependence in remission |
| 24441 | E240z00 | Opioid drug dependence NOS |
| 11840 | E242.00 | Cocaine type drug dependence |
| 25808 | E242000 | Cocaine dependence, unspecified |
| 25748 | E242100 | Cocaine dependence, continuous |
| 39836 | E242200 | Cocaine dependence, episodic |
| 52765 | E242300 | Cocaine dependence in remission |
| 33942 | E242z00 | Cocaine drug dependence NOS |
| 8284 | E243.00 | Cannabis type drug dependence |
| 40720 | E243.11 | Hashish dependence |
| 23712 | E243.12 | Hemp dependence |
| 37316 | E243.13 | Marihuana dependence |
| 24616 | E243000 | Cannabis dependence, unspecified |
| 42923 | E243100 | Cannabis dependence, continuous |
| 52794 | E243200 | Cannabis dependence, episodic |
| 44991 | E243300 | Cannabis dependence in remission |
| 33462 | E243z00 | Cannabis drug dependence NOS |
| 22186 | E244.00 | Amphetamine or other psychostimulant dependence |
| 41476 | E244.11 | Psychostimulant dependence |
| 25670 | E244.12 | Stimulant dependence |
| 37472 | E244000 | Amphetamine or psychostimulant dependence, unspecified |
| 38360 | E244100 | Amphetamine or psychostimulant dependence, continuous |
| 49585 | E244200 | Amphetamine or psychostimulant dependence, episodic |
| 46800 | E244300 | Amphetamine or psychostimulant dependence in remission |
| 25646 | E244z00 | Amphetamine or psychostimulant dependence NOS |
| 32887 | E245.00 | Hallucinogen dependence |
| 21683 | E245.11 | LSD dependence |
| 72371 | E245.12 | Lysergic acid diethylamide dependence |
| 92353 | E245000 | Hallucinogen dependence, unspecified |
| 71086 | E245100 | Hallucinogen dependence, continuous |
| 73448 | E245200 | Hallucinogen dependence, episodic |
| 70578 | E245300 | Hallucinogen dependence in remission |
| 68150 | E245z00 | Hallucinogen dependence NOS |
| 5203 | E246.00 | Glue sniffing dependence |
| 38072 | E246000 | Glue sniffing dependence, unspecified |
| 97375 | E246100 | Glue sniffing dependence, continuous |
| 33774 | E246200 | Glue sniffing dependence, episodic |
| 70761 | E246300 | Glue sniffing dependence in remission |
| 59184 | E246z00 | Glue sniffing dependence NOS |
| 51197 | E247.00 | Other specified drug dependence |
| 87505 | E247.11 | Absinthe addiction |
| 59676 | E247000 | Other specified drug dependence, unspecified- ISN'T THIS NON SPEconfidence intervalFIC |
| 24998 | E247100 | Other specified drug dependence, continuous |
| 72564 | E247200 | Other specified drug dependence, episodic |
| 64269 | E247300 | Other specified drug dependence in remission |
| 40781 | E247z00 | Other specified drug dependence NOS |
| 26061 | E248.00 | Combined opioid with other drug dependence |
| 56194 | E248000 | Combined opioid with other drug dependence, unspecified |
| 64265 | E248100 | Combined opioid with other drug dependence, continuous |
| 64277 | E248200 | Combined opioid with other drug dependence, episodic |
| 52451 | E248300 | Combined opioid with other drug dependence in remission |
| 73737 | E248z00 | Combined opioid with other drug dependence NOS |
| 14809 | E249.00 | Combined drug dependence, excluding opioids |
| 91260 | E249000 | Combined drug dependence, excluding opioid, unspecified |
| 53678 | E249100 | Combined drug dependence, excluding opioid, continuous |
| 62717 | E249200 | Combined drug dependence, excluding opioid, episodic |
| 70900 | E249300 | Combined drug dependence, excluding opioid, in remission |
| 72342 | E249z00 | Combined drug dependence, excluding opioid, NOS |
| 21087 | E24A.00 | Ecstasy type drug dependence |
| 29446 | E24z.00 | Drug dependence NOS |
| 7747 | E25..00 | Nondependent abuse of drugs |
| 42140 | E252000 | Nondependent cannabis abuse, unspecified |
| 39983 | E252100 | Nondependent cannabis abuse, continuous |
| 25448 | E252200 | Nondependent cannabis abuse, episodic |
| 53071 | E252300 | Nondependent cannabis abuse in remission |
| 25526 | E252z00 | Nondependent cannabis abuse NOS |
| 5610 | E253.00 | Nondependent hallucinogen abuse |
| 60048 | E253.11 | Bad trips |
| 16071 | E253.12 | LSD reaction |
| 71060 | E253000 | Nondependent hallucinogen abuse, unspecified |
| 95955 | E253100 | Nondependent hallucinogen abuse, continuous |
| 95956 | E253200 | Nondependent hallucinogen abuse, episodic |
| 67462 | E253300 | Nondependent hallucinogen abuse in remission |
| 48131 | E253z00 | Nondependent hallucinogen abuse NOS |
| 26831 | E255.00 | Nondependent opioid abuse |
| 40536 | E255000 | Nondependent opioid abuse, unspecified |
| 58731 | E255100 | Nondependent opioid abuse, continuous |
| 64382 | E255200 | Nondependent opioid abuse, episodic |
| 95953 | E255300 | Nondependent opioid abuse in remission |
| 69508 | E255z00 | Nondependent opioid abuse NOS |
| 10860 | E256.00 | Nondependent cocaine abuse |
| 32931 | E256000 | Nondependent cocaine abuse, unspecified |
| 46896 | E256100 | Nondependent cocaine abuse, continuous |
| 43901 | E256200 | Nondependent cocaine abuse, episodic |
| 58934 | E256300 | Nondependent cocaine abuse in remission |
| 64338 | E256z00 | Nondependent cocaine abuse NOS |
| 22481 | E257.00 | Nondependent amphetamine or other psychostimulant abuse |
| 32751 | E257.11 | Psychostimulant abuse |
| 46732 | E257.12 | Stimulant abuse |
| 39058 | E257000 | Nondependent amphetamine/psychostimulant abuse, unspecified |
| 52841 | E257100 | Nondependent amphetamine/psychostimulant abuse, continuous |
| 25229 | E257200 | Nondependent amphetamine or psychostimulant abuse, episodic |
| 43176 | E257300 | Nondependent amphetamine/psychostimulant abuse in remission |
| 47836 | E257z00 | Nondependent amphetamine or psychostimulant abuse NOS |
| 53008 | E259.00 | Nondependent mixed drug abuse |
| 56337 | E259000 | Nondependent mixed drug abuse, unspecified |
| 62887 | E259100 | Nondependent mixed drug abuse, continuous |
| 52953 | E259200 | Nondependent mixed drug abuse, episodic |
| 52842 | E259300 | Nondependent mixed drug abuse in remission |
| 33838 | E259z00 | Nondependent mixed drug abuse NOS |
| 16161 | E25y.00 | Nondependent other drug abuse |
| 63076 | E25y000 | Nondependent other drug abuse, unspecified |
| 54800 | E25y100 | Nondependent other drug abuse, continuous |
| 64983 | E25y200 | Nondependent other drug abuse, episodic |
| 52846 | E25y300 | Nondependent other drug abuse in remission |
| 64316 | E25yz00 | Nondependent other drug abuse NOS |
| 1588 | E25z.00 | Misuse of drugs NOS |
| 47335 | Eu11.00 | [X]Mental and behavioural disorders due to use of opioids |
| 42456 | Eu11000 | [X]Mental & behav dis due to use opioids: acute intoxication |
| 34249 | Eu11200 | [X]Mental and behav dis due to use opioids: dependence syndr |
| 50964 | Eu11500 | [X]Mental & behav dis due to use opioids: psychotic disorder |
| 27652 | Eu11700 | [X]Men & beh dis due opioids: resid & late-onset psychot dis |
| 52739 | Eu11y00 | [X]Men & behav dis due to use opioids: oth men & behav dis |
| 91801 | Eu11z00 | [X]Ment & behav dis due use opioids: unsp ment & behav dis |
| 10655 | Eu12.00 | [X]Mental and behavioural disorders due to use cannabinoids |
| 37389 | Eu12000 | [X]Mental & behav dis due cannabinoids: acute intoxication |
| 50343 | Eu12100 | [X]Mental and behav dis due to use cannabinoids: harmful use |
| 56504 | Eu12200 | [X]Mental and behav dis due to cannabinoids: dependence synd |
| 65681 | Eu12300 | [X]Mental and behav dis due cannabinoids: withdrawal state |
| 38429 | Eu12500 | [X]Mental & behav dis due to cannabinoids: psychotic disordr |
| 97561 | Eu12600 | [X]Mental and behav dis due to use cannabinoids: amnesic syn |
| 57574 | Eu12700 | [X]Mnt/bh dis due cannabinds: resid & late-onset psychot dis |
| 64308 | Eu12y00 | [X]Men/behav dis due to use cannabinoids: oth men/behav disd |
| 64987 | Eu12z00 | [X]Ment/behav dis due use cannabinoids: unsp ment/behav disd |
| 32052 | Eu14.00 | [X]Mental and behavioural disorders due to use of cocaine |
| 49566 | Eu14000 | [X]Mental & behav dis due to use cocaine: acute intoxication |
| 47739 | Eu14100 | [X]Mental and behav dis due to use of cocaine: harmful use |
| 50302 | Eu14200 | [X]Mental and behav dis due to use cocaine: dependence syndr |
| 67535 | Eu14300 | [X]Mental and behav dis due to use cocaine: withdrawal state |
| 49565 | Eu14500 | [X]Mental & behav dis due to use cocaine: psychotic disorder |
| 102591 | Eu14700 | [X]Men & beh dis due cocaine: resid & late-onset psychot dis |
| 44742 | Eu15000 | [X]Mnt/beh dis due oth stim inc caffein: acute intoxication |

## Eating disorder (ICD-10 F50)

Prevalence of eating disorders was rare in the CPRD (e.g. ≤1% prevalence) and therefore was included in the other mental disorders category.

Eating disorder code list

| Medical code | Read code | Read Term |
| --- | --- | --- |
| 8027 | 1467 | H/O: anorexia nervosa |
| 11612 | 8HTN.00 | Referral to eating disorders clinic |
| 95883 | 9Nk9.00 | Seen in eating disorder clinic |
| 2135 | E271.00 | Anorexia nervosa |
| 7743 | E275.00 | Other and unspecified non-organic eating disorders |
| 44544 | E275000 | Unspecified non-organic eating disorder |
| 4377 | E275100 | Bulimia (non-organic overeating) |
| 11608 | E275111 | Compulsive eating disorder |
| 61236 | E275y00 | Other specified non-organic eating disorder |
| 32892 | E275z00 | Non-organic eating disorder NOS |
| 6159 | Eu50.00 | [X]Eating disorders |
| 30570 | Eu50000 | [X]Anorexia nervosa |
| 34929 | Eu50100 | [X]Atypical anorexia nervosa |
| 9581 | Eu50200 | [X]Bulimia nervosa |
| 6583 | Eu50211 | [X]Bulimia NOS |
| 33863 | Eu50300 | [X]Atypical bulimia nervosa |
| 39383 | Eu50400 | [X]Overeating associated with other psychological disturbncs |
| 17439 | Eu50411 | [X]Psychogenic overeating |
| 16622 | Eu50500 | [X]Vomiting associated with other psychological disturbances |
| 34995 | Eu50y00 | [X]Other eating disorders |
| 62150 | Eu50y11 | [X]Pica in adults |
| 36946 | Eu50z00 | [X]Eating disorder, unspecified |
| 605 | R036011 | [D]Bulimia NOS |
| 12201 | Z4B5.00 | Eating disorder counselling |
| 67510 | ZC2CD00 | Dietary advice for eating disorder |

## Learning disorder (ICD-10 F70-79, F81.0-81.9)

Prevalence of learning disorders was rare in the CPRD (e.g. ≤1% prevalence) and therefore was included in the other mental disorders category.

Learning disorder code list

| Medical code | Read code | Read term |
| --- | --- | --- |
| 102234 | 9HB7.11 | Did not attend learning disabilities annual health check |
| 106219 | 9mA..00 | Learning disability annual health check invitation |
| 43445 | 9HB2.00 | Learning disabilities health action plan reviewed |
| 43447 | 9HB0.00 | Learning disabilities health action plan declined |
| 33276 | Z7CD100 | Learning performance |
| 100648 | Eu81700 | [X]Profound learning disability |
| 56376 | Z7CD200 | Learning difficulties |
| 43436 | 9HB4.00 | Learning disabilities health action plan completed |
| 106276 | 9mA2200 | Learning disability annual health check invtation 3rd letter |
| 106274 | 9mA2100 | Learning disability annual health check invtation 2nd letter |
| 19445 | 9HB..00 | Learning disabilities administration status |
| 4672 | E2F2.00 | Other specific learning difficulty |
| 32952 | 9HB5.00 | Learning disabilities annual health assessment |
| 32511 | 9HB3.00 | Learning disabilities health assessment |
| 99774 | Eu81600 | [X]Mild learning disability |
| 47098 | Z7CD.00 | Learning observations |
| 32667 | 8Hg2.00 | Discharge from learning disability team |
| 9466 | ZV40000 | [V]Problems with learning |
| 96895 | 69DB.00 | Learning disability health examination |
| 93406 | 9N0y.00 | Seen in learning disabilities clinic |
| 22760 | 918e.00 | On learning disability register |
| 41391 | 9HB1.00 | Learning disabilities health action plan offered |
| 100729 | 9HB7.00 | Did not attend learning disabilities annual health assessmnt |
| 106248 | 9mA1.00 | Learning disability annual health check telephone invitation |
| 16855 | Eu81z12 | [X]Learning disorder NOS |
| 4477 | Eu81z11 | [X]Learning disability NOS |
| 106249 | 9mA0.00 | Learning disability annual health check verbal invitation |
| 19436 | ZS34.11 | Learning disability |
| 100730 | 9HB6.00 | Learning disabilities annual health assessment declined |
| 18815 | 8HHP.00 | Referral to learning disability team |
| 100980 | 9hL..00 | Exception reporting: learning disability quality indicators |
| 94684 | 8H4f.00 | Referral to learning disabilities psychiatrist |
| 107968 | Eu81800 | [X]Specific learning disability |
| 98293 | Eu81500 | [X]Severe learning disability |
| 106272 | 9mA2000 | Learning disability annual health check invtation 1st letter |
| 100965 | 9HB6.11 | Learning disabilities annual health check declined |
| 2052 | 13Z4E00 | Learning difficulties |
| 106116 | Z7CD211 | LD - Learning difficulties |
| 98342 | Eu81400 | [X]Moderate learning disability |
| 103187 | 8Ce6.00 | Preferred place of care - learning disability unit |
| 106247 | 9mA2.00 | Learning disability annual health check letter invitation |
| 44987 | Z7CBB00 | Below average intellect |
| 11866 | Z7CBE00 | Intellectual functioning disability |
| 40894 | ZL5B500 | Referral to psychiatrist for mental handicap |
| 8158 | ZV62311 | [V]Educational handicap |
| 97406 | ZLD2f00 | Discharge by psychiatrist for mental handicap |
| 32588 | ZL1B500 | Under care of psychiatrist for mental handicap |
| 56503 | ZV79.11 | [V]Screening for mental developmental handicap |
| 4246 | 6664 | Mental handicap problem |
| 27502 | 689Z.00 | Mental/dev.handicap screen NOS |
| 60656 | ZRH1.00 | Functional communicat assessm physically handicap children |
| 32924 | ZLE9400 | Discharge from mental handicap psychiatry service |
| 38954 | ZL9D500 | Seen by psychiatrist for mental handicap |
| 60473 | Eu71z00 | [X]Mod mental retardation without mention impairment behav |
| 56577 | E31..00 | Other specified mental retardation |
| 42589 | Eu7z.00 | [X]Unspecified mental retardation |
| 51268 | Eu73.00 | [X]Profound mental retardation |
| 46504 | Eu70000 | [X]Mld mental retard with statement no or min impairm behav |
| 1787 | E30..00 | Mild mental retardation, IQ in range 50-70 |
| 42886 | Eu7z000 | [X]Unsp mental retard with statement no or min impairm behav |
| 52602 | Eu84400 | [X]Overactive disorder assoc mental retard/stereotype movts |
| 70008 | Eu7y000 | [X]Oth mental retard with statement no or min impairm behav |
| 42520 | Eu7yy00 | [X]Other mental retardation, other impairments of behaviour |
| 54179 | E31z.00 | Other specified mental retardation NOS |
| 59407 | Eu71y00 | [X]Mod retard oth behav impair |
| 6123 | Eu71.00 | [X]Moderate mental retardation |
| 22645 | 1Ba2.00 | Thought retardation |
| 71196 | Eu7y.00 | [X]Other mental retardation |
| 66383 | Eu7zy00 | [X]Unspecified mental retardatn, other impairments of behav |
| 30362 | R034y11 | [D]Global retardation |
| 37867 | E3z..00 | Mental retardation NOS |
| 55406 | Eu81013 | [X]Specific reading retardation |
| 36143 | Eu72.00 | [X]Severe mental retardation |
| 50606 | Eu70z00 | [X]Mild mental retardation without mention impairment behav |
| 55560 | Eu72z00 | [X]Sev mental retardation without mention impairment behav |
| 60913 | Eu71000 | [X]Mod mental retard with statement no or min impairm behav |
| 63273 | Eu7yz00 | [X]Other mental retardation without mention impairment behav |
| 55848 | Eu72y00 | [X]Severe mental retardation, other impairments of behaviour |
| 50751 | Eu72100 | [X]Sev mental retard sig impairment behav req attent/treatmt |
| 302 | E310.00 | Moderate mental retardation, IQ in range 35-49 |
| 66827 | ZS32.12 | Specific reading retardation |
| 50947 | Eu72000 | [X]Sev mental retard with statement no or min impairm behav |
| 68900 | Eu81111 | [X]Specific spelling retardation without reading disorder |
| 1362 | E3...00 | Mental retardation |
| 34174 | Eu84112 | [X]Mental retardation with autistic features |
| 54881 | Eu71100 | [X]Mod mental retard sig impairment behav req attent/treatmt |
| 90276 | Eu73y00 | [X]Profound mental retardation, other impairments of behavr |
| 4825 | E311.00 | Severe mental retardation, IQ in range 20-34 |
| 57199 | E3y..00 | Other specified mental retardation |
| 56547 | Eu7y100 | [X]Oth mental retard sig impairment behav req attent/treatmt |
| 45133 | E312.00 | Profound mental retardation with IQ less than 20 |
| 28740 | Eu70.00 | [X]Mild mental retardation |
| 60062 | Eu73z00 | [X]Prfnd mental retardation without mention impairment behav |
| 28962 | Eu7..00 | [X]Mental retardation |
| 39412 | Eu70100 | [X]Mld mental retard sig impairment behav req attent/treatmt |
| 32820 | Eu7zz00 | [X]Unsp mental retardation without mention impairment behav |
| 39016 | Eu70y00 | [X]Mild mental retardation, other impairments of behaviour |
| 66783 | Eu7z100 | [X]Unsp mentl retard sig impairment behav req attent/treatmt |
| 98100 | Eu73100 | [X]Profound ment retard sig impairmnt behav req attent/treat |
| 34498 | 1JB0.00 | Suspected Downs syndrome |
| 1543 | PJ0..00 | Down's syndrome - trisomy 21 |
| 10759 | PJ0z.00 | Down's syndrome NOS |
| 101309 | PJ02.11 | Partial trisomy 21 in Down's syndrome |
| 10956 | PKy9300 | Prader - Willi syndrome |
| 27280 | PKy0.12 | Prader-Willi syndrome |
| 41461 | PKy0.11 | Prader-Willi Syndrome |
| 56371 | PJ53400 | Individual with autosomal fragile site |
| 104279 | 677C400 | Carrier of fragile X gene mutation |
| 32603 | PJyy400 | Fragile X syndrome |
| 105180 | F1y0.00 | Fragile X associated tremor ataxia syndrome |
| 10628 | PJyy200 | Fragile X chromosome |
| 4749 | PK61.00 | Sturge-Weber syndrome |
| 4479 | PK5..00 | Tuberous sclerosis |
| 98617 | PKyz711 | Angelman syndrome |
| 25306 | PKyz511 | Angelman syndrome |
| 97059 | PKyz700 | Angelman's syndrome |
| 16087 | PKy4.00 | William syndrome |
| 89727 | PKy0.13 | Noonan's syndrome |
| 10068 | PKy8000 | Noonan's syndrome |
| 21418 | PKy6011 | Cornelia de Lange syndrome |
| 31795 | PJ31.00 | Cri-du-chat syndrome |
| 39017 | PJ2z.00 | Edward's syndrome NOS |
| 107162 | PJ22.11 | Partial trisomy 18 in Edward's syndrome |
| 33642 | PJ2..00 | Edward's syndrome - trisomy 18 |
| 4318 | C301.00 | Phenylketonuria |
| 64361 | ZC2C600 | Dietary advice for phenylketonuria |
| 107237 | 1JT..00 | Suspected phenylketonuria |

## Major chronic illness (Charlson Index)

Major chronic illness code list

| Medical code | Read term | Read code |
| --- | --- | --- |
| 42602 | Uncomplicated presenile dementia | E001000 |
| 17359 | Chest infection - unspecified bronchitis | H30..11 |
| 37272 | Other specified leukaemia | B67..00 |
| 37493 | Other cerebrovascular disease NOS | G67z.00 |
| 49132 | Malignant neoplasm of medulla oblongata | B517100 |
| 91943 | Type I diabetes mellitus with polyneuropathy | C10EC11 |
| 101311 | Insulin dependent diabetes mellitus with polyneuropathy | C10EC12 |
| 5267 | Intrinsic asthma | H331.00 |
| 17874 | Mesothelioma of peritoneum | B181.00 |
| 83454 | EPULIS | 2104EP |
| 15907 | Malignant neoplasm gallbladder/extrahepatic bile ducts NOS | B16z.00 |
| 15979 | Unspecified gastrojejunal ulcer with perforation | J14y200 |
| 51921 | Malignant neoplasm of pubis | B306200 |
| 5137 | Leukaemic reticuloendotheliosis | B624.11 |
| 51138 | Sequelae/other unspecified cerebrovascular diseases | G68W.00 |
| 64668 | Insulin treated Type II diabetes mellitus | C10FJ11 |
| 106061 |  |  |
| 13279 | Other specified diabetes mellitus with renal complications | C104y00 |
| 24503 | Cardiac failure therapy | 8B29.00 |
| 95772 | Malignant neoplasm of upper buccal sulcus | B051000 |
| 43951 | Diabetic - cooperative patient | 66AK.00 |
| 7416 | Asthma disturbing sleep | 663N.00 |
| 13103 | O/E - left eye preproliferative diabetic retinopathy | 2BBS.00 |
| 42070 | Malignant neoplasm of lower-outer quadrant of female breast | B345.00 |
| 36633 | Hyperosmolar non-ketotic state in type 2 diabetes mellitus | C109K00 |
| 38438 | Presenile dementia NOS | E001z00 |
| 45222 | Malignant neoplasm of lower-inner quadrant of female breast | B343.00 |
| 31546 | Malignant neoplasm of central part of female breast | B341.00 |
| 78703 | LYMPHOMA, NON HODGKINS | 2029CH |
| 8443 | Brain stem stroke syndrome | G663.00 |
| 51326 | Other precerebral artery occlusion | G63y.00 |
| 95458 | Malignant neoplasm of nasal bone | B300300 |
| 94220 | Malignant neoplasm of adrenal medulla | B540100 |
| 4970 | [D]Gangrene | R054.00 |
| 7804 | Chronic glomerulonephritis | K02..00 |
| 67995 | Focal membranoproliferative glomerulonephritis | K032000 |
| 74880 | OVARIAN TUMOUR | 1830T |
| 78597 | BRONCHITIS PURULENT | 466 D |
| 79909 | MALIGNANT NEOPLASM OVARY | 1830A |
| 76858 | CARconfidence intervalNOMA OVARY | 1830C |
| 2462 | Hodgkin's disease | B61..00 |
| 68155 | Malignant neoplasm of fundus of corpus uteri | B430100 |
| 8181 | Traumatic subdural haemorrhage | S628.00 |
| 74025 | ASTHMA | 493 |
| 74285 | EMPHYSEMA PULMONARY | 492 |
| 74717 | CHRONIC BRONCHITIS | 491 |
| 74205 | BRONCHITIS | 490 |
| 108638 |  |  |
| 52236 | Malnutrition-related diabetes mellitus | C10A.00 |
| 64482 | Acute pyelonephritis without medullary necrosis | K101000 |
| 32945 | Heart failure care plan discussed with patient | 8CL3.00 |
| 70005 | Suture of ulcer of stomach NEC | 761J111 |
| 65490 | Secondary cancer of the vulva | B58y411 |
| 10946 | Malignant neoplasm of ascending colon | B136.00 |
| 45071 | Malignant neoplasm of connective and soft tissue of abdomen | B314.00 |
| 63925 | [X]Malignant neoplasm of meninges, unspecified | ByuA200 |
| 28769 | Diabetic on insulin and oral treatment | 66AV.00 |
| 5798 | Chronic asthmatic bronchitis | H312000 |
| 9088 | Malignant neoplasm of hepatic flexure of colon | B130.00 |
| 10864 | Malignant neoplasm of descending colon | B132.00 |
| 11104 | Perforated chronic gastric ulcer | J111211 |
| 3213 | Malignant neoplasm of corpus uteri, excluding isthmus | B430.00 |
| 82043 | ULCER MARGINAL | 5349MR |
| 84112 | SPASTIC HEMIPLEGIA | 344 SH |
| 60857 | Chronic nephritic syn diffuse crescentic glomerulonephritis | K0A3700 |
| 10809 | Chronic membranous glomerulonephritis | K021.00 |
| 61930 | [X]Renal failure | Kyu2.00 |
| 16967 | Malignant neoplasm of overlapping lesion of corpus uteri | B432.00 |
| 65064 | Chronic rapidly progressive glomerulonephritis | K023.00 |
| 57671 | Megakaryocytic leukaemia | B672.00 |
| 70380 | Malignant neoplasm of skin of axillary fold | B335000 |
| 17262 | Non-insulin-dependent diabetes mellitus with retinopathy | C109600 |
| 26652 | Malig neop nasal cavities, middle ear and accessory sinuses | B20..00 |
| 69204 | Multilobular portal cirrhosis | J615100 |
| 101805 | Malignant neoplasm of lacrimal sac | B507000 |
| 44196 | Hodgkin's granuloma | B611.00 |
| 38939 | Hodgkin's disease, lymphocytic-histiocytic predominance | B613.00 |
| 60059 | [X]Primary degen dementia, Alzheimer's type, presenile onset | Eu00012 |
| 25704 | [X]Presenile dementia,Alzheimer's type | Eu00011 |
| 47649 | Type 1 diabetes mellitus with ophthalmic complications | C10E100 |
| 54222 | Malignant neoplasm of connective and soft tissue of foot | B312400 |
| 88247 | MALIGNANT NEOPLASM ANUS | 1736AN |
| 10636 | Hepatorenal syndrome | J624.00 |
| 62674 | Type 2 diabetes mellitus with mononeuropathy | C10FA00 |
| 70528 | Human immunodeficiency virus with secondary infection | A788500 |
| 5002 | Diabetic polyneuropathy | F372.11 |
| 2342 | Diabetic neuropathy | F372.12 |
| 23480 | Malignant neoplasm of perianal skin | B335900 |
| 61194 | Malignant neoplasm of skin of lower limb or hip NOS | B337z00 |
| 15868 | Malignant neoplasm of skin of trunk, excluding scrotum, NOS | B335z00 |
| 95630 | True histiocytic lymphoma | B62x600 |
| 68332 | [X]2ndry malignant neoplasm/oth?? parts/nervous system | ByuC600 |
| 84564 | PLASMACYTOMA | 203 PL |
| 95992 | Type I diabetes mellitus without complication | C108A11 |
| 101836 | Human immunodeficiency virus with secondary cancers | A788600 |
| 106915 |  |  |
| 38331 | Other lymphoid leukaemia NOS | B64yz00 |
| 319 | Malignant neoplasm of larynx | B21..00 |
| 63216 | Obliterative bronchiolitis due to chemical fumes | H464100 |
| 3903 | Malignant neoplasm of bronchus or lung NOS | B22z.00 |
| 28639 | Follicular non-Hodgkin's small cleaved cell lymphoma | B627000 |
| 107916 |  |  |
| 77143 | SERO POSITIVE RHEUMATOID ARTHRITIS | L 151F |
| 79981 | SERO NEGATIVE RHEUMATOID ARTHRITIS | L 151E |
| 82911 | SQUAMOUS CELL CARconfidence intervalNOMA FRONTAL & ETHMOI | 1608CM |
| 54685 | Malignant melanoma of upper arm | B326100 |
| 46153 | Malignant neoplasm of parametrium | B443.00 |
| 47672 | Nephrotic syndrome in systemic lupus erythematosus | K01x400 |
| 42831 | Type 1 diabetes mellitus with neurological complications | C10E200 |
| 84019 | CARconfidence intervalNOMA EXTRAHEPATIC BILE DUCT | 1561C |
| 30779 | Heart failure annual review | 662W.00 |
| 31167 | Asthma night-time symptoms | 66YP.00 |
| 6813 | H/O: diabetes mellitus | 1434 |
| 52570 | Malignant neoplasm, overlapping lesion of penis | B487.00 |
| 64195 | Malig neop of endocrine gland or related structure NOS | B54z.00 |
| 711 | Diabetes mellitus | C10..00 |
| 80316 | CARconfidence intervalNOMA THROAT | 149 CT |
| 61071 | Type 2 diabetes mellitus with hypoglycaemic coma | C109D12 |
| 17841 | Malignant neoplasm of glans penis | B481.00 |
| 47204 | Letterer-Siwe disease NOS | B625z00 |
| 38343 | Secondary and unspec malig neop submental lymph nodes | B560700 |
| 21549 | Follicular non-Hodgkin's lymphoma | B627C00 |
| 95715 | Mucosa-associated lymphoma | B627900 |
| 50076 | HIV disease resulting in multiple infections | A789400 |
| 66270 | Malignant neoplasm of upper lip, external | B000000 |
| 10692 | Type 1 diabetes mellitus with ketoacidosis | C10EM00 |
| 69993 | Type 1 diabetes mellitus with gangrene | C10E600 |
| 88235 | MALIGNANT NEOPLASM PANCREAS | 1579A |
| 76045 | PANCREAS CARconfidence intervalNOMA | 1579C |
| 26054 | Type 2 diabetes mellitus with persistent proteinuria | C10FL00 |
| 94441 | Malignant neoplasm of lower lip, oral aspect | B003300 |
| 30202 | Intracerebral haemorrhage, intraventricular | G617.00 |
| 42153 | Malignant melanoma of other specified skin site | B32y.00 |
| 64918 | Secondary and unspec malig neop of superficial parotid LN | B560000 |
| 16500 | Secondary malignant neoplasm of other specified site NOS | B58z.00 |
| 70509 | Diffuse non-Hodgkin's centroblastic lymphoma | B627D00 |
| 1208 | Childhood asthma | H330.12 |
| 15248 | Hay fever with asthma | H330.13 |
| 7731 | Pollen asthma | H330.14 |
| 79537 | MALIGNANT NEOPLASM SINUS (ACCESSORY RESP | 1609A |
| 73744 | Malignant melanoma of ear and external auricular canal NOS | B322z00 |
| 98740 | Malignant neoplasm of upper lip, vermilion border NOS | B000z00 |
| 87113 | Malignant neoplasm-pluriglandular involvement,unspecified | B54X.00 |
| 107878 |  |  |
| 5842 | Secondary malignant neoplasm of other specified sites | B58..00 |
| 97547 | Malignant neoplasm of intrathoracic site NOS | B551200 |
| 47582 | Type 1 diabetes mellitus with renal complications | C10E000 |
| 40598 | [X]Malignant neoplasm of female genital organs | Byu7.00 |
| 100532 | Sezary's disease NOS | B622z00 |
| 40592 | [X]Malignant neoplasm of mesothelial and soft tissue | Byu5.00 |
| 73777 | Leukaemic reticuloendotheliosis NOS | B624z00 |
| 49145 | Secondary malignant neoplasm of penis | B58y700 |
| 18712 | Renal malignant neoplasm | B4A..11 |
| 68399 | Malignant neoplasm of lip unspecified, mucosa | B004200 |
| 107771 |  |  |
| 50777 | Malignant neoplasm,overlap lesion periph nerve & auton ns | B524600 |
| 13569 | Disseminated malignancy NOS | B590.00 |
| 73139 | Oesophageal varices without bleeding in diseases EC | G852100 |
| 70942 | Malignant neoplasm of hypothalamus | B510400 |
| 92955 | Acute vesicular emphysema | H32y000 |
| 53704 | Acute yellow atrophy | J600200 |
| 76518 | PAROTID GLAND ADENOMA | 2102AP |
| 3163 | Tracheobronchitis NOS | H300.00 |
| 63979 | Malignant neoplasm of frenulum linguae | B013100 |
| 35235 | Other acute renal failure | K04y.00 |
| 10792 | Stroke monitoring | 662M.00 |
| 104134 |  |  |
| 93353 | Sequoiosis (red-cedar asthma) | H35y600 |
| 46242 | Acute renal cortical necrosis | K041.00 |
| 73213 | Secondary malignant neoplasm of other urinary organs | B581.00 |
| 33843 | Secondary malignant neoplasm of brain and spinal cord | B583.00 |
| 74295 | BRONCHIECTASIS | 518 |
| 96235 | Type I diabetes mellitus maturity onset | C10E911 |
| 40053 | Generalised ischaemic cerebrovascular disease NOS | G671.00 |
| 105209 |  |  |
| 95949 | Mycosis fungoides of unspecified site | B621000 |
| 107236 |  |  |
| 61677 | Secondary and unspec malig neop inferior mesenteric LN | B562200 |
| 53910 | Malignant neoplasm of clitoris | B453.00 |
| 39317 | Diabetes mellitus, adult onset, neurological manifestation | C106100 |
| 43761 | Malignant neoplasm of labia majora | B451.00 |
| 39923 | Malignant neoplasm of middle lobe of lung | B223100 |
| 29643 | Acute inferoposterior infarction | G303.00 |
| 14898 | Lateral myocardial infarction NOS | G305.00 |
| 35875 | Monocytic leukaemia | B66..00 |
| 16639 | [V]Personal history of malignant neoplasm of breast | ZV10300 |
| 5198 | Secondary malignant neoplasm of brain | B583000 |
| 14671 | Acute gastric ulcer with perforation | J110200 |
| 108103 |  |  |
| 85471 | GLOMERULONEPHRITIS CHRONIC | 583 GC |
| 42940 | Lung disease with polymyositis | H57y100 |
| 95182 | Malignant neoplasm of talus | B308100 |
| 23672 | Gangrene of hand | G732400 |
| 108005 |  |  |
| 49067 | Rheumatoid arthritis of hip | N040B00 |
| 45491 | Diabetes mellitus with unspecified complication | C10z.00 |
| 38488 | Malignant neoplasm of ventral tongue surface NOS | B013z00 |
| 107258 |  |  |
| 82757 | CONGESTIVE HEART FAILURE COMPENSATED | 4270CC |
| 104963 |  |  |
| 7219 | Carcinoma of rectum | B141.11 |
| 5901 | Rectal carcinoma | B141.12 |
| 46255 | Malignant melanoma of lower limb and hip | B327.00 |
| 10098 | Other specified diabetes mellitus with other spec comps | C10yy00 |
| 99231 | Type I diabetes mellitus with mononeuropathy | C108B11 |
| 42429 | Malignant neoplasm overlapping lesion of skin | B33X.00 |
| 33580 | Nephritis and nephropathy unspecified | K03..00 |
| 43089 | Uncomplicated arteriosclerotic dementia | E004000 |
| 98616 | Type II diabetes mellitus with neurological complications | C10F211 |
| 27458 | Chronic monocytic leukaemia | B661.00 |
| 82660 | TRACHEA CARconfidence intervalNOMA | 1620C |
| 87621 | NEOPLASM MALIGNANT TRACHEA | 1620A |
| 77109 | LEUKAEMIA ACUTE | 2070 |
| 105095 |  |  |
| 41369 | Lymphosarcoma and reticulosarcoma | B60..00 |
| 79977 | ULCER GASTRIC PERFORATED | 5310 |
| 66457 | [V]Personal history of other specified malignant neoplasm | ZV10y00 |
| 66145 | Type I diabetes mellitus with ketoacidotic coma | C10EN11 |
| 72197 | Lymphosarcoma cell leukaemia | B67y000 |
| 53989 | Malig neop connective and soft tissue upper limb/shoulder | B311.00 |
| 84858 | METASTASIS BRONCHUS | 1973M |
| 26506 | Asthma severely restricts exercise | ######## |
| 28003 | Choriocarcinoma | B420.00 |
| 98408 | Malig neop of connective and soft tissue of thorax NOS | B313z00 |
| 15175 | Duodenal ulcer NOS | J12z.00 |
| 83255 | CARconfidence intervalNOMA TONSIL | 1460C |
| 40966 | Malignant neoplasm of sacral vertebra | B306300 |
| 23951 | HIV disease resulting in candidiasis | A789200 |
| 40671 | [X]Malignant neoplasm of male genital organs | Byu8.00 |
| 63482 | Unspecified gastrojejunal ulcer | J14y.00 |
| 88240 | MALIGNANT NEOPLASM CAECUM | 1530AC |
| 26134 | Malignant neoplasm of epiglottis, free border | B064000 |
| 44260 | Insulin dependent diabetes mellitus with diabetic cataract | C108F00 |
| 40023 | Diabetes mellitus, juvenile type, with hyperosmolar coma | C102000 |
| 75877 | CONGESTIVE HEART FAILURE | 4270 |
| 74131 | LVF (LEFT VENTRICULAR FAILURE) | 4271 |
| 86707 | MALIGNANT NEOPLASM LIVER PRIMARY | 1550AP |
| 35053 | Secondary malig neop of respiratory and digestive systems | B57..00 |
| 24397 | Malignant neoplasm of tonsillar fossa | B061.00 |
| 84622 | BRONCHITIS SUBACUTE | 466 BC |
| 27931 | Kaposi's sarcoma of skin | B33z000 |
| 100352 | Lymphosarcoma of lymph nodes of inguinal region and leg | B601500 |
| 90562 | MYELOFIBROSIS SECONDARY | 209 BF |
| 23082 | Gastrojejunal ulcer (GJU) | J14..00 |
| 58121 | Malignant neoplasm of anterior 2/3 of tongue unspecified | B014.00 |
| 65165 | [X]Other leukaemia of unspecified cell type | ByuD900 |
| 65745 | [X]Other subarachnoid haemorrhage | Gyu6100 |
| 11124 | Bleeding acute gastric ulcer | J110111 |
| 4137 | Secondary malignant neoplasm of lung | B570.00 |
| 16213 | Secondary malignant neoplasm of pleura | B572.00 |
| 241 | Acute myocardial infarction | G30..00 |
| 74798 | MELANOMA MALIGNANT | 1729 |
| 54134 | Malignant neoplasm of middle lobe, bronchus or lung NOS | B223z00 |
| 44443 | Insulin dependent diabetes mellitus with ulcer | C108500 |
| 68018 | [V]Personal history of malignant neoplasm of anus | ZV10011 |
| 64568 | [V]Personal history of malig neop of gastrointestinal tract | ZV10012 |
| 58177 | [V]Personal history of malignant neoplasm of liver | ZV10015 |
| 57727 | [V]Personal history of malignant neoplasm of large intestine | ZV10014 |
| 62785 | [V]Personal history of malignant neoplasm of rectum | ZV10017 |
| 51001 | [V]Personal history of malignant neoplasm of oesophagus | ZV10016 |
| 99931 | [V]Personal history of malignant neoplasm of tongue | ZV10019 |
| 49447 | [V]Personal history of malignant neoplasm of stomach | ZV10018 |
| 81562 | HEPATITIS CHRONIC AGGRESSIVE | 070 G |
| 95188 | CKD stage 3 without proteinuria | 1Z1C.11 |
| 95176 | CKD stage 3A without proteinuria | 1Z1E.11 |
| 35385 | Type 2 diabetes mellitus with neuropathic arthropathy | C10FH00 |
| 29876 | Hodgkin's, lymphocytic-histiocytic predominance NOS | B613z00 |
| 77740 | ULCER DUODENAL RECURRANCE | 5329BD |
| 30577 | Malignant neoplasm of skin of fore-arm | B336200 |
| 100002 | Malignant neoplasm of tonsillar fossa NOS | B062z00 |
| 28556 | Malignant melanoma of skin NOS | B32z.00 |
| 18505 | IDDM-Insulin dependent diabetes mellitus | C108.11 |
| 9029 | Chronic active hepatitis | J614100 |
| 106058 |  |  |
| 84368 | Secondary and unspec malig neop internal iliac lymph nodes | B565000 |
| 67211 | Malignant neoplasm of spinal meninges NOS | B523z00 |
| 105385 |  |  |
| 67029 | [X]Other lymphoid leukaemia | ByuD500 |
| 95180 | CKD stage 3B with proteinuria | 1Z1F.11 |
| 99312 | CKD stage 4 with proteinuria | 1Z1H.11 |
| 40401 | Non-insulin dependent diabetes mellitus with gangrene | C109500 |
| 104609 |  |  |
| 79182 | HEPATITIS CHRONIC ACTIVE | 5730CA |
| 47810 | Malignant neoplasm of unspecified site | B59..00 |
| 6863 | Cirrhosis and chronic liver disease | J61..00 |
| 94355 | Malignant neoplasm of flank NOS | B55y200 |
| 102252 | [X]HIV disease resulting in other specified conditions | AyuCC00 |
| 75850 | BASAL CELL PAPILLOMA | 1739BP |
| 83047 | RHEUMATISM NONARTICULAR | 7179FN |
| 49074 | Type 2 diabetes mellitus with ulcer | C10F400 |
| 57427 | Malignant lymphoma NOS of unspecified site | B62y000 |
| 92062 | MENINGIOMA BRAIN MALIGNANT | 1929MN |
| 44284 | Unspecified gastric ulcer NOS | J11yz00 |
| 54305 | Malignant melanoma of knee | B327200 |
| 63408 | Tube graft abdominal Aortic aneurysm (emergency) | 7A13411 |
| 19444 | [X]Malignant melanoma of skin, unspecified | Byu4100 |
| 21402 | Burkitt's lymphoma | B602.00 |
| 53515 | Malignant neoplasm skin of ear and external auricular canal | B332.00 |
| 48149 | Sequelae of intracerebral haemorrhage | G681.00 |
| 49360 | Malignant neoplasm of lower gum | B031.00 |
| 5867 | Exercise induced asthma | 173A.00 |
| 27819 | Obstructive chronic bronchitis | H312.00 |
| 38978 | Malignant neoplasm of liver and intrahepatic bile ducts NOS | B15z.00 |
| 25603 | Simple chronic bronchitis | H310.00 |
| 107032 |  |  |
| 55463 | Secondary and unspec malig neop post mediastinal lymph nodes | B561400 |
| 44335 | Acute duodenal ulcer without mention of complication | J120000 |
| 71450 | HIV disease resulting/unspcf infectious disease | A788X00 |
| 95629 | Malignant melanoma of perineum | B325500 |
| 53369 | Malignant melanoma of great toe | B327900 |
| 1323 | Diabetic retinopathy | F420.00 |
| 89480 | MENINGIOMA SPINAL CORD MALIGNANT | 1923 |
| 3462 | Duodenal erosion | J123.00 |
| 67451 | Malignant neoplasm/overlap lesion/bone??? cartilage | B30W.00 |
| 9853 | Chronic duodenal ulcer | J121.00 |
| 57260 | Malignant melanoma of ear and external auricular canal | B322.00 |
| 65625 | Malignant melanoma of scalp and neck | B324.00 |
| 69951 | Malignant neoplasm of roof of mouth | B055100 |
| 65164 | Malignant melanoma of upper limb and shoulder | B326.00 |
| 62080 | Malignant neoplasm of skin of external auditory meatus | B332100 |
| 30526 | [X]Mesothelioma, unspecified | Byu5100 |
| 25366 | Secondary and unspec malig neop ant mediastinal lymph nodes | B561300 |
| 73538 | Secondary and unspec malig neop axilla and upper limb LN NOS | B563z00 |
| 63718 | Endoscopic injection haemostasis of gastric ulcer | 761D600 |
| 74561 | EPITHELIOMA BASAL CELL | 1739B |
| 82131 | CARconfidence intervalNOMA SKIN | 1739C |
| 79908 | SKIN NEOPLASM MALIGNANT | 1739A |
| 31225 | Asthma causes daytime symptoms 1 to 2 times per month | 663t.00 |
| 23770 | Acquired immune deficiency syndrome | A788.00 |
| 24363 | Diabetic stabilisation | 8A13.00 |
| 106597 |  |  |
| 97198 | Acute renal failure due to urinary obstruction | K044.00 |
| 108656 |  |  |
| 41931 | Malignant neoplasm of cheek NOS | B550100 |
| 9618 | Secondary and unspecified malignant neoplasm of lymph nodes | B56..00 |
| 31794 | Unspecified B-cell non-Hodgkin's lymphoma | B627W00 |
| 51708 | HIV dis reslt/oth mal neopl/lymph,h'matopoetc? tissu | A789X00 |
| 99493 | Malignant neoplasm of upper lip, inner aspect | B002.00 |
| 104275 |  |  |
| 61523 | Other specified diabetes mellitus with neurological comps | C106y00 |
| 105083 |  |  |
| 7978 | Hypernephroma | B4A0000 |
| 106884 |  |  |
| 67506 | Hodgkin's nodular sclerosis of intrathoracic lymph nodes | B614200 |
| 50904 | Secondary and unspec malig neop infraclavicular lymph nodes | B563200 |
| 95508 | Chronic kidney disease stage 5 with proteinuria | 1Z1K.00 |
| 36161 | Malignant neoplasm of tongue, tip and lateral border | B012.00 |
| 65062 | Diabetes mellitus NOS with ketoacidotic coma | C103z00 |
| 107949 |  |  |
| 95253 | PAGET'S DISEASE SCROTUM | 1735CP |
| 42505 | Diabetes mellitus NOS with ketoacidosis | C101z00 |
| 63105 | Malignant lymphoma NOS of lymph node inguinal region and leg | B62y500 |
| 45267 | Malignant neoplasm of other and ill defined site NOS | B55z.00 |
| 15027 | Malignant lymphoma NOS | B62yz00 |
| 56202 | [X]Seropositive rheumatoid arthritis, unspecified | Nyu1G00 |
| 27483 | Malignant neoplasm of thymus | B240.00 |
| 105267 |  |  |
| 76765 | INTRINSIC ASTHMA | 493 HT |
| 41694 | Mushroom workers' lung | H355.00 |
| 37549 | Kaposi's sarcoma of palate | B05z000 |
| 89593 | Malignant neoplasm of intrahepatic biliary passages | B151200 |
| 42824 | Asthma daytime symptoms | 663q.00 |
| 4892 | Status asthmaticus NOS | H33z000 |
| 83557 | HEART FAILURE RIGHT-SIDED | 4270R |
| 74146 | CONGESTIVE CARDIAC FAILURE | 4270C |
| 83955 | CONGESTIVE HEART FAILURE DECOMPENSATED | 4270D |
| 6806 | Malignant neoplasm of small intestine and duodenum | B12..00 |
| 55588 | [X]Malignant neoplasm of female genital organ, unspecified | Byu7300 |
| 45781 | Precerebral arterial occlusion | G63..00 |
| 95177 | Chronic kidney disease stage 3B without proteinuria | 1Z1G.00 |
| 77971 | ADENOCARconfidence intervalNOMA SIGMOID COLON | 1533AD |
| 10698 | Malignant neoplasm of vaginal vault | B450100 |
| 12455 | Type I diabetes mellitus | C10E.11 |
| 89481 | MESOTHELIOMA ABDOMEN | 1950MA |
| 98392 | Maturity onset diabetes in youth type 1 | C10C.12 |
| 62840 | Malignant neoplasm of ventral surface of tongue | B013.00 |
| 13066 | Asthma - currently dormant | 663h.00 |
| 1407 | Insulin treated Type 2 diabetes mellitus | C10FJ00 |
| 76543 | LIVER confidence intervalRRHOSIS | 5719HP |
| 63357 | Diabetes mellitus, adult, peripheral circulatory disorder | C107100 |
| 57235 | Malignant neoplasm of endocervical canal | B410000 |
| 8355 | Asthma monitored | 9OJA.11 |
| 56715 | Malignant neoplasm of other site of female breast | B34y.00 |
| 21983 | Type 1 diabetes mellitus with renal complications | C108012 |
| 91674 | Mycosis fungoides of intra-abdominal lymph nodes | B621300 |
| 9707 | Seropositive errosive rheumatoid arthritis | N047.00 |
| 87935 | PAGET'S DISEASE BREAST | 174 PB |
| 48946 | Chronic gastric ulcer without mention of complication | J111000 |
| 16280 | Malignant neoplasm of neck NOS | B550400 |
| 53095 | Allergic alveolitis and pneumonitis NOS | H35zz00 |
| 80490 | NEOPLASM MALIGNANT GENITAL TRACT FEMALE | 1849A |
| 42274 | Acute gastrojejunal ulcer NOS | J140z00 |
| 48832 | Rheumatoid arthritis of wrist | N040700 |
| 47205 | Malignant overlapping lesion of tongue | B017.00 |
| 37096 | Malignant neoplasm of tongue, junctional zone | B015.00 |
| 108360 |  |  |
| 91586 | Malignant neoplasm of connective and soft tissue of finger | B311400 |
| 104532 |  |  |
| 5414 | Gangrene of toe | G732000 |
| 70716 | Immunoproliferative neoplasm | B62zz11 |
| 35399 | Diabetes mellitus with peripheral circulatory disorder | C107.00 |
| 94104 | Other specified operation on gastric ulcer | 761Jy00 |
| 81888 | CARconfidence intervalNOMA RECURRENT | 1991CR |
| 12582 | Malignant neoplasm of lower lobe of lung | B224100 |
| 95012 | Mycosis fungoides of lymph nodes of multiple sites | B621800 |
| 25181 | Asthma restricts exercise | 663e.00 |
| 79531 | CARconfidence intervalNOMA KIDNEY | 1890C |
| 85527 | NEOPLASM MALIGNANT KIDNEY | 1890A |
| 40014 | Malignant neoplasm of soft tissue of face | B310100 |
| 83549 | WILM'S TUMOUR | 1890W |
| 92110 | SARCOMA KIDNEY | 1890R |
| 67356 | Chronic gastric ulcer with obstruction | J111400 |
| 4870 | Histiocytosis X (acute, progressive) | B625.11 |
| 104391 |  |  |
| 65400 | Chronic diffuse glomerulonephritis | K02y300 |
| 54494 | Malignant neoplasm of nipple and areola of male breast | B350.00 |
| 107959 |  |  |
| 28163 | Malignant neoplasm of colon NOS | B13z.00 |
| 15165 | Presenile dementia | E001.00 |
| 844 | Rheumatoid arthritis | N040.00 |
| 48519 | Malignant neoplasm of junctional region of epiglottis | B065.00 |
| 64602 | Malignant neoplasm of undescended testis | B470.00 |
| 51795 | Malignant neoplasm of glomus jugulare | B545000 |
| 68641 | Malignant neoplasm of brain stem NOS | B517z00 |
| 95783 | Malignant neoplasm of specified site of pancreas NOS | B17yz00 |
| 66750 | Malignant neoplasm of heart, thymus and mediastinum NOS | B24z.00 |
| 41491 | Post-infective bronchiectasis | H341.00 |
| 5909 | Chronic wheezy bronchitis | H312011 |
| 13243 | Malignant neoplasm of trachea, bronchus and lung | B22..00 |
| 19162 | Malignant neoplasm of anterior wall of urinary bladder | B493.00 |
| 80926 | ADENOCARconfidence intervalNOMA BREAST | 174 DC |
| 90435 | ADENOCARconfidence intervalNOMA BREAST ULCERATION | 174 DL |
| 38907 | Other specified peripheral vascular disease | G73y.00 |
| 42416 | Malignant neoplasm of lower third of oesophagus | B105.00 |
| 98142 | Siewert type I adenocarcinoma | B107.00 |
| 82999 | LATENT DIABETES | 2500AH |
| 96628 | Acute gastrojejunal ulcer with haemorrhage | J140100 |
| 6842 | Impaired renal function | K060.11 |
| 96635 | Malignant neoplasm of ectopic pancreatic tissue | B17y000 |
| 87779 | DI GUGLIELMO'S DISEASE | 2072DG |
| 50152 | Malignant sacral teratoma | B306500 |
| 50789 | Malignant neoplasm of upper third of oesophagus | B103.00 |
| 73718 | Malig neop connective and soft tissue head, face, neck NOS | B310z00 |
| 80401 | GANGRENE FOOT | 4459FT |
| 31102 | Malignant neoplasm of urinary bladder NOS | B49z.00 |
| 102704 |  |  |
| 86184 | MACRONODULAR confidence intervalRRHOSIS | 5719MA |
| 18278 | Insulin treated Type 2 diabetes mellitus | C109J00 |
| 21358 | Rheumatoid arthritis of shoulder | N040200 |
| 16034 | Aortic aneurysm without mention of rupture NOS | G716.00 |
| 104386 |  |  |
| 7740 | Seminoma of undescended testis | B470200 |
| 13065 | Moderate asthma | 663V200 |
| 52041 | O/E - left eye stable treated prolif diabetic retinopathy | 2BBl.00 |
| 43857 | Lipoatrophic diabetes mellitus | C10M.00 |
| 61390 | Malignant neoplasm of adrenal cortex | B540000 |
| 11433 | O/E - right eye background diabetic retinopathy | 2BBP.00 |
| 10797 | Oesophageal varices NOS | G858.00 |
| 90670 | MALIGNANT NEOPLASM UTERUS CORPUS | 1820A |
| 50527 | Type II diabetes mellitus with polyneuropathy | C10FB11 |
| 69671 | Malignant neoplasm of posterior third of tongue | B010.11 |
| 77540 | SCLERODERMA | 7340A |
| 86494 | SCLERODERMA GENERALIZED | 7340D |
| 59115 | Burkitt's lymphoma of lymph nodes of head, face and neck | B602100 |
| 89753 | NEUROBLASTOMA DISSEMINATED | 1990NB |
| 99716 | Insulin dependent diabetes mellitus with hypoglycaemic coma | C10EE12 |
| 72723 | Malignant neoplasm of cornu of corpus uteri | B430000 |
| 42461 | Hodgkin's disease NOS | B61zz00 |
| 74538 | RHEUMATIC ARTHRITIS | 7149A |
| 97587 | CKD stage 4 without proteinuria | 1Z1J.11 |
| 97683 | CKD stage 5 without proteinuria | 1Z1L.11 |
| 19280 | Anterior cerebral artery syndrome | G661.00 |
| 9237 | Malignant neoplasm of larynx NOS | B21z.00 |
| 96802 | Malignant neoplasm of posterior wall of stomach NEC | B11y100 |
| 66646 | Malignant neoplasm, overlap lesion of resp & intrathor orgs | B26..00 |
| 4017 | Old myocardial infarction | G32..00 |
| 106519 |  |  |
| 44452 | Malignant neoplasm of vomer | B300C00 |
| 41515 | [X]Malignant neoplasm/central nervous system, unspecified | ByuA100 |
| 88905 | NEOPLASM MALIGNANT GENITAL TRACT MALE | 1879A |
| 51858 | Other allergic alveolitis | H35y.00 |
| 77965 | FARMERS' LUNG | 5161F |
| 107701 |  |  |
| 7805 | Malignant neoplasm of ovary | B440.00 |
| 22487 | Secondary diabetes mellitus | C10N.00 |
| 45077 | Malignant neoplasm of skin of back | B335700 |
| 33444 | Malignant neoplasm of hilus of lung | B221100 |
| 28807 | Subarachnoid haemorrhage following injury | S62..12 |
| 65357 | Malignant neoplasm of nasolacrimal duct | B507100 |
| 1555 | Bronchial asthma | H33..11 |
| 18245 | Malignant neoplasm of skin of lip | B330.00 |
| 30747 | Malignant neoplasm of skin of upper limb and shoulder | B336.00 |
| 39027 | [X]Malignant neoplasm of other specified sites | ByuC000 |
| 54234 | Malignant neoplasm of scalp and skin of neck | B334.00 |
| 9913 | Heart failure confirmed | 1O1..00 |
| 60499 | Insulin dependent diabetes mellitus with gangrene | C108600 |
| 85407 | GANGRENE DIABETIC | 250 GA |
| 37806 | Type 2 diabetes mellitus with peripheral angiopathy | C10FF00 |
| 758 | Type 2 diabetes mellitus | C10F.00 |
| 44884 | Malignant neoplasm of other urinary organs | B4Ay.00 |
| 36695 | Diabetes mellitus autosomal dominant type 2 | C10D.00 |
| 103946 |  |  |
| 24693 | Non-insulin dependent diabetes mellitus with arthropathy | C109G00 |
| 49403 | Malignant neoplasm of skin of chin | B333100 |
| 106131 |  |  |
| 3293 | Right hemiplegia | F223.00 |
| 80976 | BRILL- SYMMERS DISEASE | 2020BR |
| 33969 | Malnutrition-related diabetes mellitus with ketoacidosis | C10A100 |
| 42218 | Malignant neoplasm of other specified sites | B55y.00 |
| 33388 | Malignant neoplasm of adenoid | B071000 |
| 49525 | Kaposi's sarcoma, unspecified | B59zX00 |
| 7176 | Myeloid leukaemia | B65..00 |
| 348 | Ca female breast | B34..11 |
| 46008 | Malignant neoplasm skin other and unspec part of face NOS | B333z00 |
| 3837 | Diabetic maculopathy | F420400 |
| 43479 | Malignant neoplasm of jejunum | B121.00 |
| 27528 | Malignant neoplasm of ribs, sternum and clavicle | B303.00 |
| 45490 | Malignant neoplasm of corpus uteri NOS | B430z00 |
| 53081 | Unspecified gastric ulcer | J11y.00 |
| 34001 | Lung disease due to external agents NOS | H4z..00 |
| 63568 | Malignant neoplasm of peripheral nerves of head, face & neck | B524000 |
| 24048 | Malignant neoplasm of retrocaecal tissue | B180200 |
| 17460 | Diffuse non-Hodgkin's lymphoblastic (diffuse) lymphoma | B627700 |
| 10099 | Advanced diabetic maculopathy | F420300 |
| 59903 | Diabetic amyotrophy | C106.11 |
| 7795 | Diabetes mellitus with neuropathy | C106.12 |
| 16491 | Diabetes mellitus with polyneuropathy | C106.13 |
| 71881 | Chronic duodenal ulcer with haemorrhage and perforation | J121300 |
| 99096 | [X]Malignant neopl/overlapping les/resp???? organs | Byu2300 |
| 60092 | Malignant lymphoma NOS of spleen | B62y700 |
| 99644 | Nephrotic syndrome??????? glomerulonephritis | K012.00 |
| 40749 | [X]Malignant neoplasm of bone and articular cartilage | Byu3.00 |
| 35816 | [V]Personal history of malignant neoplasm of bladder | ZV10511 |
| 47683 | [V]Personal history of malignant neoplasm of kidney | ZV10512 |
| 28881 | [V]Personal history of malignant neoplasm of kidney | ZV10513 |
| 46301 | Type 1 diabetes mellitus with polyneuropathy | C10EC00 |
| 17887 | Malignant lymphoma otherwise specified | B62x.00 |
| 50505 | Malignant melanoma of shoulder | B326000 |
| 107638 |  |  |
| 67217 | Malignant neoplasm of trunk NOS | B55y100 |
| 65434 | Malignant neoplasms of lymphoid and histiocytic tissue NOS | B62z.00 |
| 27330 | Leukaemic reticuloendotheliosis | B624.00 |
| 15036 | Malignant mast cell tumours | B626.00 |
| 51756 | Type 2 diabetes mellitus with ketoacidotic coma | C10FP00 |
| 2491 | Coronary thrombosis | G30..12 |
| 39085 | Flaccid hemiplegia | F220.00 |
| 61716 | Malignant neoplasm of peripheral nerve,upp limb,incl should | B524100 |
| 44120 | Toxic liver disease with fibrosis and cirrhosis of liver | J635600 |
| 57184 | [X]Oth malignant neoplasm/skin of oth??? parts of face | Byu4200 |
| 58684 | Hodgkin's mixed cellularity of intrathoracic lymph nodes | B615200 |
| 28059 | Secondary and unspec malig neop of facial lymph nodes | B560600 |
| 27641 | HIV disease resulting in Pneumocystis carinii pneumonia | A789300 |
| 93778 | Malignant neoplasm of spleen NOS | B1z1z00 |
| 50296 | Malignant neoplasm of upper lip, lipstick area | B000100 |
| 77998 | ADENOCARconfidence intervalNOMA COLON | 1538AD |
| 89318 | MALIGNANT NEOPLASM LARGE BOWEL NONRECTAL | 1538AN |
| 69278 | Non-insulin depend diabetes mellitus with diabetic cataract | C109E00 |
| 87451 | MUconfidence intervalNOUS CYSTADENOCARconfidence intervalNOMA OVARY | 1830MC |
| 18683 | Type 1 diabetes mellitus with ulcer | C10E500 |
| 83384 | LYMPHADENOMA | 2029LD |
| 103353 |  |  |
| 54600 | Unstable insulin dependent diabetes mellitus | C10E412 |
| 22524 | Secondary malignant neoplasm of other specified site NOS | B58yz00 |
| 24675 | Malignant neoplasm of nasopharynx | B07..00 |
| 86433 | TUMOUR TESTIS SERTOLI CELL | 186 AN |
| 39554 | Malignant neoplasm of vallecula | B063.00 |
| 85267 | HYPEROSMOLAR DIABETIC STATE | 250 NH |
| 77043 | UNSTABLE DIABETIC | 250 NT |
| 10647 | Nephritis - chronic | K02..11 |
| 11875 | Nephropathy - chronic | K02..12 |
| 6578 | [X]Vascular dementia | Eu01.00 |
| 106970 |  |  |
| 1735 | Aortic aneurysm | G71..00 |
| 1735 | Aortic aneurysm | g71..00 |
| 89909 | Malignant neoplasm of lower lip, mucosa | B003200 |
| 18390 | Type 2 diabetes mellitus with persistent microalbuminuria | C10FM00 |
| 104291 |  |  |
| 37247 | Chronic obstructive pulmonary disease NOS | H3z..11 |
| 55246 | Malignant neoplasm of accessory sinus NOS | B20z.00 |
| 108886 |  |  |
| 58871 | Malignant histiocytosis NOS | B623z00 |
| 94005 | Hodgkin's disease, mixed cellularity NOS | B615z00 |
| 79961 | NEOPLASM MALIGNANT BRONCHUS | 1621AB |
| 73537 | Malig neop auditory tube, middle ear, mastoid air cells NOS | B201z00 |
| 96003 | Malignant neoplasm of junction of hard and soft palate | B055000 |
| 74406 | TRANSITIONAL CELL CARconfidence intervalNOMA BLADDER | 188 TC |
| 4555 | Malig neop of other and unspecified female genital organs | B45..00 |
| 26270 | [X]Lewy body dementia | Eu02500 |
| 80791 | MENINGEAL HAEMORRHAGE TRAUMATIC | 8520M |
| 90733 | HAEMORRHAGE SUBARACHNOID TRAUMATIC | 8520A |
| 90024 | HODGKIN'S GRANULOMA | 201 G |
| 12464 | Peripheral T-cell lymphoma | B62x200 |
| 23861 | Malignant neoplasm of chest wall NOS | B551100 |
| 10418 | Type 1 diabetes mellitus with nephropathy | C10ED00 |
| 18616 | Secondary malignant neoplasm of other specified sites | B58y.00 |
| 60335 | Secondary malignant neoplasm of vulva | B58y400 |
| 81835 | ASTHMA EPISODIC | 493 EP |
| 82405 | ULCER PEPTIC PERFORATED | 5330 |
| 97499 | Siewert type II adenocarcinoma | B118.00 |
| 65777 | Thrombocytic leukaemia | B672.11 |
| 69132 | Secondary and unspec malig neop external iliac lymph nodes | B562400 |
| 73988 | Malignant neoplasm of peripheral nerve of pelvis | B524500 |
| 44627 | Secondary and unspec malig neop anterior cervical LN | B560800 |
| 58604 | Type II diabetes mellitus with retinopathy | C109611 |
| 42762 | Type 2 diabetes mellitus with retinopathy | C109612 |
| 54636 | Malignant neoplasm of ethmoid sinus | B203.00 |
| 100232 | Malig neop of other site of heart, thymus and mediastinum | B24y.00 |
| 9505 | Secondary malignant neoplasm of skin of breast | B582600 |
| 62126 | Malignant neoplasm of thalamus | B510500 |
| 74516 | HEART FAILURE | 7824FH |
| 79580 | MYOCARDIAL FAILURE | 7824FM |
| 70787 | Atrophic (senile) emphysema | H32y100 |
| 49263 | [X]Dementia in Alzheimer's disease with early onset | Eu00000 |
| 64357 | Diabetes mellitus NOS with unspecified complication | C10zz00 |
| 74348 | ASTHMA EXERconfidence intervalSE INDUCED | 493 EB |
| 28241 | Malignant neoplasm of ureteric orifice | B496.00 |
| 104981 |  |  |
| 95505 | Malignant neoplasm of cervical stump | B41y000 |
| 45427 | Pituitary snuff-takers' disease | H35y500 |
| 38304 | Closed traumatic subarachnoid haemorrhage | S620.00 |
| 88177 | MEMBRANOPROLIFERATIVE GLOMERULONEPHRITIS | 583 MP |
| 86101 | MESANGIOCAPILLARY GLOMERULONEPHRITIS | 583 MA |
| 80833 | NEPHROPATHY MEMBRANOUS | 583 MN |
| 63723 | Lymphosarcoma NOS | B601z00 |
| 69767 | [X]HIV disease resulting in other non-Hodgkin's lymphoma | AyuC600 |
| 51410 | Asbestosis NOS | H41z.00 |
| 95351 | Type II diabetes mellitus with mononeuropathy | C10FA11 |
| 31210 | Hepatoblastoma of liver | B150100 |
| 37315 | Diabetic mononeuropathy | F3y0.00 |
| 50298 | Malignant neoplasm of orbital bone | B300500 |
| 67236 | Malignant neoplasm of hippocampus | B512000 |
| 23446 | Silica pneumoconiosis NOS | H42z.00 |
| 9696 | Subarachnoid haemorrhage from posterior communicating artery | G604.00 |
| 71403 | Acute gastric ulcer with haemorrhage and perforation | J110300 |
| 104139 |  |  |
| 104639 |  |  |
| 105797 |  |  |
| 60134 | Secondary malignant neoplasm of ureter | B581000 |
| 37590 | Malignant neoplasm of hard palate | B052.00 |
| 76545 | confidence intervalRRHOSIS ALCOHOLIC | 5710CA |
| 95539 | Maternally inherited diabetes mellitus | C10FS00 |
| 61692 | Malignant neoplasm of lip unspecified, inner aspect | B004.00 |
| 61741 | Malignant neoplasm of humerus | B304200 |
| 24456 | Malig neop auditory tube, middle ear and mastoid air cells | B201.00 |
| 89981 | MALIGNANT NEOPLASM EAR INNER | 1601A |
| 51057 | Gangrene of thumb | G732300 |
| 87198 | TESTIS ADENOCARconfidence intervalNOMA INFANCY | 186 BL |
| 30632 | Other specified leukaemia NOS | B67z.00 |
| 46613 | Malignant neoplasm of specified parts of peritoneum | B18y.00 |
| 50960 | Pre-existing diabetes mellitus, insulin-dependent | L180500 |
| 80827 | CARconfidence intervalNOMA GALLBLADDER | 1560C |
| 12139 | Acute anterolateral infarction | G300.00 |
| 80394 | MALIGNANT NEOPLASM VOCAL CORDS | 1610A |
| 39570 | Asthma causes night symptoms 1 to 2 times per month | 663r.00 |
| 51767 | Pure sensory lacunar syndrome | G666.00 |
| 108719 |  |  |
| 48073 | Malignant neoplasm of basal ganglia | B510000 |
| 86367 | PAROTID MALIGNANT NEOPLASM | 1420A |
| 102112 | Type I diabetes mellitus with gangrene | C10E611 |
| 109051 |  |  |
| 75550 | ULCER PREPYLORIC | 5319PP |
| 83017 | ULCER PEPTIC STOMACH | 5319PT |
| 80699 | DEMENTIA AGGRESSIVE | 299 G |
| 48537 | Malignant neoplasm of other specified sites of pancreas | B17y.00 |
| 89618 | MALIGNANT NEOPLASM THIGH | 1959DB |
| 108866 |  |  |
| 34012 | Malignant neoplasm of hypopharynx | B08..00 |
| 19226 | Malignant neoplasm of parietal lobe | B513.00 |
| 52323 | Multiple gastric ulcers | J11z.12 |
| 96090 | Chronic gastrojejunal ulcer | J141.00 |
| 102151 | Malignant neoplasm of overlapping lesion of tonsil | B060200 |
| 56268 | Type II diabetes mellitus with hypoglycaemic coma | C109D11 |
| 7146 | Extrinsic (atopic) asthma | H330.00 |
| 26319 | Oesophageal varices in cirrhosis of the liver | G852200 |
| 27651 | Secondary carcinoma of other specified sites | B58..11 |
| 35357 | Malignant neoplasm of rectum, rectosigmoid junction and anus | B14..00 |
| 30322 | [V]Personal history of malignant neoplasm of urinary organ | ZV10500 |
| 7602 | Chronic alcoholic hepatitis | J617000 |
| 64670 | Lymphosarcoma of intra-abdominal lymph nodes | B601300 |
| 91895 | Malignant neoplasm of glossoepiglottic fold | B064100 |
| 83679 | HYPERTENSION CONGESTIVE HEART FAILURE | 402 C |
| 64446 | Insulin dependent diab mell with peripheral angiopathy | C108G00 |
| 7046 | Malignant neoplasm of body of uterus | B43..00 |
| 76231 | RHEUMATOID ARTHRITIS INCREASED ACTIVITY | 7123CR |
| 1220 | Malignant neoplasm of colon | B13..00 |
| 38931 | Malignant neoplasm of genitourinary organ OS | B4y..00 |
| 38862 | Malignant neoplasm of trigone of urinary bladder | B490.00 |
| 42539 | Acute erythraemia and erythroleukaemia | B670.00 |
| 58836 | Malig neop of connective and soft tissue of pelvis NOS | B315z00 |
| 88688 | HEMIPLEGIA WITH HYPERTENSION | 4380HP |
| 3366 | Severe asthma | 663V300 |
| 504 | Transient cerebral ischaemia | G65..00 |
| 49194 | Berylliosis | H432.00 |
| 62556 | Malignant neoplasm of thymus, heart and mediastinum | B24..00 |
| 77516 | SALIVARY GLAND MALIGNANT NEOPLASM | 1429B |
| 86359 | CARconfidence intervalNOMA SALIVARY GLAND | 1429C |
| 59036 | Malignant neoplasm of bones of skull and face | B300.00 |
| 54631 | Malignant neoplasm of pelvic bones, sacrum and coccyx | B306.00 |
| 80514 | SECONDARY NEOPLASTIC DEPOSITS LIVER | 1977 |
| 80561 | SECONDARY NEOPLASTIC DEPOSITS LUNG | 1970 |
| 60052 | Malignant neoplasm of specified site NOS | B55yz00 |
| 80364 | THYMOMA MALIGNANT | 1942AM |
| 37553 | Malignant neoplasm of lip, unspecified | B007.00 |
| 15709 | Malignant neoplasm of digestive organs and peritoneum | B1...00 |
| 93384 | Unspec malig neop lymphoid/histiocytic of intrathoracic node | B62z200 |
| 56954 | Malignant neoplasm of skin of knee | B337200 |
| 6125 | Diabetic annual review | 66AS.00 |
| 107807 |  |  |
| 93951 | Hodgkin's, lymphocytic-histiocytic pred inguinal and leg | B613500 |
| 105862 |  |  |
| 95016 | Malignant neoplasm of Waldeyer's ring | B0z1.00 |
| 43940 | Malignant neoplasm of isthmus of uterine body | B431.00 |
| 15489 | Chronic hepatitis NOS | J614z00 |
| 53599 | Malignant neoplasm of frontal bone | B300100 |
| 65721 | Mast cell leukaemia | B673.00 |
| 19928 | Peptic ulcer NOS | J13z.00 |
| 58630 | Biliary cirrhosis NOS | J616z00 |
| 26448 | Malignant neoplasm of faucial tonsil | B060000 |
| 82609 | CHRONIC NEPHRITIS | 582 N |
| 37112 | Malignant neoplasm of histiocytic tissue | B6...11 |
| 23492 | Chronic bullous emphysema NOS | H320z00 |
| 54253 | [X]Secondary malignant neoplasm of other specified sites | ByuC700 |
| 105488 |  |  |
| 46017 | Other acute myocardial infarction NOS | G30yz00 |
| 70842 | Follicular non-Hodg mixed sml cleavd & lge cell lymphoma | B627100 |
| 94390 | Malignant neoplasm of roof of nasopharynx | B070.00 |
| 26232 | Tube graft of Abdominal aortic aneurysm | 7A14411 |
| 66422 | Malignant neoplasm, overlapping lesion of nasopharynx | B074.00 |
| 96971 | Malig neop other site nasal cavity, middle ear and sinuses | B20y.00 |
| 54352 | Malignant neoplasm of skin of hand | B336300 |
| 91860 | SARCOMA TESTIS | 186 RC |
| 97832 | Secondary cancer of the cervix | B58y211 |
| 23578 | Chronic persistent hepatitis | J614000 |
| 76317 | POLYMYALGIA RHEUMATICA | 7179PR |
| 61118 | Simple chronic bronchitis NOS | H310z00 |
| 68027 | [X]Malignant neoplasm/other and unspecified cranial nerves | ByuA000 |
| 103034 |  |  |
| 24694 | Insulin dependent diabetes mellitus with mononeuropathy | C108B00 |
| 90124 | Malignant neoplasm of posterior wall of oropharynx | B067.00 |
| 60188 | Giant bullous emphysema | H320200 |
| 34268 | Type 2 diabetes mellitus with neurological complications | C10F200 |
| 96226 | [X]Malignant neoplasm/overlap lesion/other?defined sites | ByuC100 |
| 74766 | HAEMORRHAGE INTRACEREBRAL | 4319CE |
| 89348 | LEUKAEMIA MONOCYTIC | 2069 |
| 88914 | MONOCYTIC LEUKAEMIA CHRONIC | 2061 |
| 84925 | PANCOAST TUMOUR | 1621D |
| 80957 | NEOPLASM MALIGNANT LUNG | 1621A |
| 74492 | LUNG CARconfidence intervalNOMA | 1621C |
| 66043 | Other chronic bronchitis | H31y.00 |
| 57958 | Unspecified gastric ulcer with haemorrhage | J11y100 |
| 61693 | [X]Other myeloid leukaemia | ByuD600 |
| 34912 | Non-insulin dependent diabetes mellitus with ulcer | C109400 |
| 43392 | Malignant neoplasm of penis, part unspecified | B483.00 |
| 16416 | Chronic leukaemia NOS | B681.00 |
| 105250 |  |  |
| 45307 | Carcinoma of respiratory tract and intrathoracic organs | B2...11 |
| 15821 | Stress ulcer NOS | J13..11 |
| 31608 | Malignant neoplasm of other site of uterine body | B43y.00 |
| 6701 | Secondary and unspec malig neop intrapelvic lymph nodes | B565.00 |
| 67711 | Acute peptic ulcer unspecified | J130y00 |
| 89989 | SCLERODERMA ACROSCLEROTIC | 7340BC |
| 42579 | Malignant lymphoma NOS of intra-abdominal lymph nodes | B62y300 |
| 92757 | ACROSCLEROSIS | 7340BA |
| 43614 | Malignant neoplasm/bones??? cartilage/limb,unspfd | B30X.00 |
| 94397 | Unspec gastric ulcer; unspec haemorrhage and/or perforation | J11yy00 |
| 39878 | Malignant melanoma of popliteal fossa area | B327300 |
| 101778 | Malignant neoplasm of broad ligament | B442.00 |
| 100786 | Chronic eosinophilic leukaemia | B651000 |
| 79364 | MALIGNANT NEOPLASM OESOPHAGUS | 150 A |
| 68197 | Malignant neoplasm of skin of popliteal fossa area | B337300 |
| 91620 | MALIGNANT NEOPLASM NOSE INTERNAL | 1600A |
| 95219 | MYELOBLASTOSIS WITH REFRACTORY ANAEMIA | 2050RA |
| 107163 |  |  |
| 105613 |  |  |
| 37620 | Chronic peptic ulcer with perforation | J131200 |
| 43390 | Malignant neoplasm of small intestine NOS | B12z.00 |
| 76788 | CARconfidence intervalNOMA BLADDER | 188 C |
| 83735 | MALIGNANT NEOPLASM BLADDER | 188 A |
| 47767 | Malignant neoplasm of scrotum | B486.00 |
| 95671 | [X]Malignant neoplasm of peritoneum, unspecified | Byu5700 |
| 65123 | Leukaemic reticuloend of intra-abdominal lymph nodes | B624300 |
| 101715 | Hodgkin's disease, lymphocytic depletion of spleen | B616700 |
| 58692 | Secondary and unspec malig neop paratracheal lymph nodes | B561500 |
| 79361 | LEUKAEMIA MYELOBLASTIC ACUTE | 2050MB |
| 79888 | LEUKAEMIA MYELOMONOCYTIC ACUTE | 2050MM |
| 52141 | [V]Personal history of malignant neoplasm of ovary | ZV10414 |
| 46779 | [V]Personal history of malignant neoplasm of uterine body | ZV10417 |
| 83352 | OBSTRUCTIVE LUNG DISEASE | 5199CL |
| 48808 | [V]Personal history of malignant neoplasm of testis | ZV10416 |
| 91784 | HAEMANGIOENDOTHELIAL SARCOMA LIVER | 1550BH |
| 90982 | SARCOMA LIVER PRIMARY | 1550BP |
| 53629 | Malignant melanoma of buttock | B325200 |
| 91154 | MALIGNANT NEOPLASM SKELETAL | 1709AK |
| 28665 | Malignant neoplasm of nasopharynx NOS | B07z.00 |
| 92925 | Y graft of abdominal Aortic aneurysm (emergency) | 7A11211 |
| 14658 | Acute myocardial infarction NOS | G30z.00 |
| 99001 | Malignant neoplasm of upper lip, frenulum | B002100 |
| 80236 | STATUS ASTHMATICUS | 493 D |
| 98537 | Malignant neoplasm of tympanic cavity | B201100 |
| 67034 | [X]Mesothelioma of other sites | Byu5000 |
| 92055 | MYELOSCLEROSIS | 209 CL |
| 74848 | ULCER DUODENUM PERFORATED | 5320 |
| 65483 | Hodgkin's nodular sclerosis of lymph nodes of axilla and arm | B614400 |
| 74197 | RECTUM CARconfidence intervalNOMA | 1541C |
| 80334 | MALIGNANT NEOPLASM RECTUM | 1541A |
| 105302 |  |  |
| 95480 | Malignant neoplasm of lower lip, lipstick area | B001100 |
| 47954 | Type 2 diabetes mellitus without complication | C10F900 |
| 74457 | CEREBROVASCULAR DISEASE | 4389 |
| 87389 | CEREBROVASCULAR DISEASE WITH HYPERTENSIO | 4380 |
| 13252 | Malignant neoplasm of genitourinary organ | B4...00 |
| 64913 | Gastrocolic ulcer | J14..12 |
| 1295 | Stomal ulcer | J14..15 |
| 74381 | BRONCHIAL ASTHMA | 493 BR |
| 88423 | ASTHMA FREQUENCY ON EXERconfidence intervalSE ONLY | 493 BI |
| 84754 | ASTHMA FREQUENCY REGULARLY | 493 BG |
| 37461 | Adult T-cell leukaemia | B64y200 |
| 12490 | Malignant neoplasm of nose NOS | B550200 |
| 47862 | Malignant neoplasm of thyroid cartilage | B213300 |
| 16993 | H/O: aortic aneurysm | 14AE.00 |
| 38144 | Asthma limits walking up hills or stairs | 663w.00 |
| 24884 | Asthma causes daytime symptoms 1 to 2 times per week | 663u.00 |
| 88801 | SARCOMA COLON | 1538B |
| 81591 | MALIGNANT NEOPLASM LARGE INTESTINE | 1538A |
| 79861 | DISSECTION AORTA | 4410N |
| 91457 | [X]Malignant neoplasm/connective soft tissue,unspecified | Byu5900 |
| 55630 | Malignant neoplasm of other specified site of nasopharynx | B07y.00 |
| 46409 | Secondary and unspec malig neop pectoral lymph nodes | B563300 |
| 51957 | Type I diabetes mellitus with ulcer | C108511 |
| 93716 | Secondary and unspec malig neop intrathoracic LN NOS | B561z00 |
| 54632 | Malignant melanoma of eyelid including canthus | B321.00 |
| 31364 | Malignant neoplasm of cheek mucosa | B050.00 |
| 9600 | Mesothelioma of pleura | B232.00 |
| 37724 | Malignant neoplasm of retromolar area | B056.00 |
| 38617 | Other specified diabetes mellitus with ketoacidosis | C101y00 |
| 34089 | Malignant lymphoma NOS of lymph nodes of axilla and arm | B62y400 |
| 37516 | Malignant neoplasm of uvula | B054.00 |
| 63653 | Heilmeyer - Schoner disease | B671.11 |
| 232 | Asthma attack | H33z100 |
| 63555 | Polyneuropathy in disease NOS | F374z00 |
| 63915 | Secondary and unspec malig neop inguinal and lower limb LN | B564.00 |
| 48237 | Malignant neoplasm of prepylorus of stomach | B111000 |
| 66675 | Malnutrition-related diabetes mellitus with coma | C10A000 |
| 18231 | Phaeochromocytoma | B540.11 |
| 10919 | Sero negative polyarthritis | N04y111 |
| 56652 | Maple bark strippers' lung | H356.00 |
| 21590 | Secondary malignant neoplasm of prostate | B58y500 |
| 65025 | Diabetes mellitus NOS with peripheral circulatory disorder | C107z00 |
| 73417 | Acute duodenal ulcer with obstruction | J120400 |
| 34283 | Diabetes mellitus NOS with ophthalmic manifestation | C105z00 |
| 88717 | EXconfidence intervalSION BRONCHIECTASIS | K3441B |
| 16126 | Primary carcinoma of liver | B150000 |
| 72714 | Mycosis fungoides of lymph nodes of inguinal region and leg | B621500 |
| 8935 | Acute inferolateral infarction | G302.00 |
| 95408 | Chronic kidney disease stage 3A with proteinuria | 1Z1D.00 |
| 107195 |  |  |
| 95178 | Chronic kidney disease stage 3B with proteinuria | 1Z1F.00 |
| 94415 | Malignant histiocytosis of lymph nodes head, face and neck | B623100 |
| 95122 | Chronic kidney disease stage 4 with proteinuria | 1Z1H.00 |
| 102949 |  |  |
| 48828 | Secondary malignant neoplasm of skin of hip and leg | B582500 |
| 34648 | Renal dwarfism | K080100 |
| 45803 | [V]Personal history of malignant neoplasm of tongue | ZV10y16 |
| 76394 | DUODENAL ULCER BLEEDING | 5339DB |
| 8918 | Malignant neoplasm of liver and intrahepatic bile ducts | B15..00 |
| 22290 | Malignant neoplasm of connective and soft tissue of thorax | B313.00 |
| 67460 | Acute nephritis with lesions of necrotising glomerulitis | K001.00 |
| 87407 | KAHLER'S DISEASE | 203 KA |
| 64106 | Malignant neoplasm of specified parts of peritoneum NOS | B18yz00 |
| 42426 | Malignant neoplasm of frontal lobe | B511.00 |
| 105375 |  |  |
| 19477 | Arteriosclerotic dementia | E004.00 |
| 73510 | Malignant neoplasm of supraclavicular fossa NOS | B550500 |
| 108316 |  |  |
| 68376 | Florid cirrhosis | J612.11 |
| 15223 | Malignant neoplasm of ureter | B4A2.00 |
| 100914 | Rheumatoid arthritis of acromioclavicular joint | N040400 |
| 44740 | Sequelae of subarachnoid haemorrhage | G680.00 |
| 30165 | Malignant neoplasm of mesorectum | B18y200 |
| 18912 | Subdural haemorrhage NOS | G623.00 |
| 46850 | Type I diabetes mellitus - poor control | C108811 |
| 45914 | Type 1 diabetes mellitus - poor control | C108812 |
| 66306 | Heart failure as a complication of care | SP11111 |
| 94350 | Nephritis unsp????? glomerulonephritis lesion NOS | K032z00 |
| 57471 | Malig neop of connective and soft tissue trunk unspecified | B316.00 |
| 98723 | Type II diabetes mellitus with hypoglycaemic coma | C10FD11 |
| 36147 | Secondary malignant neoplasm of liver | B153.00 |
| 50290 | Kaposi's sarcoma of lymph nodes | B6z0.00 |
| 14777 | Extrinsic asthma without status asthmaticus | H330000 |
| 794 | Emphysema | H32..00 |
| 83090 | SENILE DETERIORATION | 794 D |
| 71585 | Precerebral artery occlusion NOS | G63z.00 |
| 92467 | SARCOMA GRANULOCYTIC | 2022GR |
| 104699 |  |  |
| 47840 | Malignant neoplasm of aortic body | B545100 |
| 39413 | Malignant neoplasm of pelvic peritoneum | B18y500 |
| 87471 | ERYTHRAEMIA ACUTE | 2072AE |
| 61513 | Mucopurulent chronic bronchitis NOS | H311z00 |
| 52212 | [X]Diabetes mellitus | Cyu2.00 |
| 56355 | Malignant neoplasm of lateral wall of oropharynx | B066.00 |
| 3063 | Paraplegia | F241.00 |
| 104152 |  |  |
| 55467 | Arteriosclerotic dementia with paranoia | E004200 |
| 81203 | PRECOMA DIABETIC | 250 HP |
| 77995 | HYPOGLYCAEMIC COMA DIABETIC | 250 HC |
| 65159 | Malignant neoplasm of perinephric tissue | B180100 |
| 7830 | Lymph node metastases | B56..11 |
| 70637 | Malignant melanoma of lip | B320.00 |
| 24021 | Peptic ulcer of oesophagus | J102000 |
| 17872 | Acute anteroseptal infarction | G301100 |
| 52138 | Chronic duodenal ulcer unspecified | J121y00 |
| 91240 | Malignant neoplasm of pons | B517300 |
| 99012 | Hodgkin's disease NOS of lymph nodes inguinal region and leg | B61z500 |
| 72262 | [V]Personal history of malig neop other intrathoracic organ | ZV10200 |
| 74491 | CARconfidence intervalNOMA CAECUM | 1530CC |
| 99160 | CKD stage 5 with proteinuria | 1Z1K.11 |
| 89182 | CARconfidence intervalNOMA DIGESTIVE ORGAN | 159 CD |
| 77928 | TUMOUR MIXED PAROTID | 2102MP |
| 16241 | Malignant neoplasm of tonsil | B060.00 |
| 100776 | Rheumatoid arthritis of sacro-iliac joint | N040C00 |
| 73961 | [X]Other specified peripheral vascular diseases | Gyu7400 |
| 38736 | Malignant neoplasm of other and unspecified site OS | B5y..00 |
| 102163 | Insulin dependent diabetes mellitus with nephropathy | C10ED12 |
| 35771 | [V]Personal history of malignant neoplasm of thyroid | ZV10y15 |
| 95058 | Reticulosarcoma of spleen | B600700 |
| 78456 | CARDIAC ASTHMA | 4271B |
| 82617 | HEART FAILURE LEFT-SIDED | 4271H |
| 107963 |  |  |
| 11370 | Asthma confirmed | 1O2..00 |
| 50402 | Malignant neoplasm of fibula | B307100 |
| 86602 | MALIGNANT NEOPLASM SIGMOID | 1533A |
| 83772 | METASTASIS GENERAL | 1990M |
| 78695 | NEOPLASTIC DISEASE DISSEMINATED | 1990C |
| 100633 | CKD stage 3B without proteinuria | 1Z1G.11 |
| 97996 | Malignant neoplasm of other site of uterine adnexa | B44y.00 |
| 92508 | MALIGNANT NEOPLASM INTESTINE | 1539AT |
| 15182 | Malignant neoplasm of connective and soft tissue, site NOS | B31z.00 |
| 60526 | Malignant neoplasm of skin of upper limb or shoulder NOS | B336z00 |
| 79714 | ASTHMA AND BRONCHITIS | 493 BD |
| 27897 | Malignant neoplasm of anus unspecified | B143.00 |
| 43400 | Malignant neoplasm of gum | B03..00 |
| 45793 | Malignant neoplasm of myometrium of corpus uteri | B430300 |
| 1749 | Hemiplegia | F22..00 |
| 36133 | O/E - paraplegia | 2835 |
| 81113 | ASTHMA EXERconfidence intervalSE INCLUDED | 493 EA |
| 83427 | SYNDROME HEPATORENAL | 573 HR |
| 87202 | MALIGNANT NEOPLASM BONE | 1709A |
| 83469 | EWING'S TUMOUR | 1709C |
| 82368 | CHONDROSARCOMA | 1709B |
| 10283 | Malignant neoplasm of tongue | B01..00 |
| 82255 | SARCOMA OSTEOGENIC | 1709D |
| 63598 | [X]Malignant neoplasms/independent (primary) multiple sites | ByuE.00 |
| 65312 | Malignant neoplasm of anterior wall of stomach NEC | B11y000 |
| 2773 | Nephritis, nephrosis and nephrotic syndrome | K0...00 |
| 80808 | MALIGNANT NEOPLASM GALLBLADDER | 1560A |
| 95572 | Chronic kidney disease stage 1 without proteinuria | 1Z18.00 |
| 46963 | Insulin-dependent diabetes mellitus with renal complications | C108000 |
| 22918 | Duodenal ulcer disease | J122.00 |
| 1754 | Chronic hepatitis | J614.00 |
| 10755 | Non proliferative diabetic retinopathy | F420600 |
| 185 | Acute exacerbation of asthma | H333.00 |
| 82282 | CONGESTIVE CARDIOMYOPATHY | 425 CC |
| 43450 | Immunoproliferative neoplasm or myeloma NOS | B63z.00 |
| 46458 | Malignant neoplasm of skin of perineum | B335600 |
| 107804 |  |  |
| 27438 | Cardiac portal cirrhosis | J615700 |
| 13564 | Cerebellar haemorrhage | G613.00 |
| 95338 | Hodgkin's, lymphocytic-histiocytic pred intrapelvic nodes | B613600 |
| 100144 | Malignant neoplasm of lip, oral aspect | B004300 |
| 95792 | Lymphoid and histiocytic malignancy NOS | B62zz00 |
| 32556 | Diabetes with gangrene | C107.12 |
| 32403 | Diabetes mellitus with gangrene | C107.11 |
| 107773 |  |  |
| 58706 | [X]Other forms of systemic lupus erythematosus | Nyu4300 |
| 57481 | [X]Secondary malignant neoplasm/oth? respiratory organs | ByuC300 |
| 6509 | Insulin dependent diabetes mellitus with retinopathy | C108700 |
| 59253 | Type 2 diabetes mellitus with arthropathy | C10FG00 |
| 6865 | Stomach ulcer operations | 761J.11 |
| 15644 | Malignant neoplasm of urethra | B4A3.00 |
| 64848 | Malignant neoplasm of ulna | B304400 |
| 91900 | Hodgkin's disease NOS of lymph nodes of axilla and arm | B61z400 |
| 67763 | Malignant neoplasm of costo-vertebral joint | B303400 |
| 61289 | Secondary and unspec malig neop deep inguinal lymph nodes | B564100 |
| 93927 | Polymyositis ossificans | N231400 |
| 98909 | Hodgkin's granuloma of lymph nodes of head, face and neck | B611100 |
| 35288 | Type 1 diabetes mellitus - poor control | C10E800 |
| 62437 | Malignant reticulosis | B62x400 |
| 43292 | Arteriosclerotic dementia with depression | E004300 |
| 43122 | Malignant neoplasm of skin of shoulder | B336000 |
| 52029 | [X]Malignant neoplasm without specification of site | ByuC800 |
| 84570 | CARconfidence intervalNOMA BREAST INDURATED | 174 confidence interval |
| 47684 | Detergent asthma | H47y000 |
| 99621 | Malignant neoplasm of cranial nerves | B520.00 |
| 104325 |  |  |
| 31188 | Malignant neoplasm of lower lobe, bronchus or lung | B224.00 |
| 45408 | Malignant neoplasm of anterior portion of floor of mouth | B040.00 |
| 68161 | Malignant neoplasm of seminal vesicle | B48y000 |
| 30700 | Malignant neoplasm of oesophagus NOS | B10z.00 |
| 53551 | Diffuse non-Hodgkin's immunoblastic (diffuse) lymphoma | B627600 |
| 17056 | Myeloproliferative disease | B6y0.11 |
| 39798 | Diffuse non-Hodgkin's lymphoma, unspecified | B627X00 |
| 88521 | MALIGNANT GOITRE | 193 AG |
| 40595 | [X]Malignant neoplasm of bronchus or lung, unspecified | Byu2000 |
| 65117 | HIV disease resulting in lymphoid interstitial pneumonitis | A789900 |
| 70126 | Malignant neoplasm of optic nerve | B520100 |
| 68105 | Type 1 diabetes mellitus with mononeuropathy | C10EB00 |
| 9030 | Malignant neoplasm of other and ill-defined sites | B55..00 |
| 71609 | Unspec malig neop lymphoid/histiocytic nodes inguinal/leg | B62z500 |
| 68787 | Malignant neoplasm of back NOS | B55y000 |
| 87162 | ERYTHROLEUKAEMIA | 2072EL |
| 44525 | Obstructive chronic bronchitis NOS | H312z00 |
| 76992 | CHRONIC RENAL FAILURE | 5932EC |
| 47252 | Malignant melanoma of other and unspecified parts of face | B323.00 |
| 37182 | Multiple myeloma and immunoproliferative neoplasms | B63..00 |
| 22441 | Malignant neoplasm of subglottis | B212.00 |
| 38689 | Malignant melanoma of trunk (excluding scrotum) | B325.00 |
| 107896 |  |  |
| 56121 | [X]Malignant neoplasm of skin, unspecified | Byu4300 |
| 37648 | Insulin treated non-insulin dependent diabetes mellitus | C109J11 |
| 18264 | Insulin treated Type II diabetes mellitus | C109J12 |
| 39433 | Secondary and unspec malig neop submandibular lymph nodes | B560500 |
| 101114 | Diffuse non-Hodgkin's large cell lymphoma | B627A00 |
| 51406 | Chronic duodenal ulcer NOS | J121z00 |
| 59004 | Malignant neoplasm of lateral wall of nasopharynx | B072.00 |
| 54202 | Malignant neoplasm of other site of male breast | B35z.00 |
| 80125 | METASTASIS CEREBRAL | 1983M |
| 104484 |  |  |
| 20092 | Malignant neoplasm of floor of mouth | B04..00 |
| 104324 |  |  |
| 104620 |  |  |
| 53591 | Malignant neoplasm of other specified part of oesophagus | B10y.00 |
| 18323 | Intrinsic asthma with asthma attack | H331111 |
| 65122 | Leukaemic reticuloendotheliosis of unspecified sites | B624000 |
| 15626 | Chronic catarrhal bronchitis | H310000 |
| 90918 | NODULAR RENAL BLASTOMA | 1890NR |
| 91843 | Malignant neoplasm of lower lip, frenulum | B003100 |
| 18425 | Type 2 diabetes mellitus with polyneuropathy | C10FB00 |
| 30645 | Malignant neoplasm of skin of cheek, external | B333000 |
| 55881 | Malignant melanoma of scalp | B324000 |
| 108424 |  |  |
| 86577 | NEUROFIBROMATOSIS MALIGNANT | 1924NF |
| 20284 | Intracranial haemorrhage NOS | G62z.00 |
| 6015 | [X]Other and unspecified cirrhosis of liver | Jyu7100 |
| 32240 | Lymphoma stage III | 4M22.00 |
| 90568 | LEUKAEMIA BLAST CELL | 2070BL |
| 12590 | Weak heart | G58z.11 |
| 17278 | Cardiac failure NOS | G58z.12 |
| 95405 | Chronic kidney disease stage 5 without proteinuria | 1Z1L.00 |
| 30511 | Malig neop of other endocrine glands and related structures | B54..00 |
| 1223 | Cardiac failure | G58..11 |
| 76493 | PROSTATE CARconfidence intervalNOMA | 185 C |
| 80370 | MALIGNANT NEOPLASM PROSTATE | 185 A |
| 103690 |  |  |
| 95406 | Chronic kidney disease stage 4 without proteinuria | 1Z1J.00 |
| 57719 | Malignant neoplasm of squamocolumnar junction of cervix | B41y100 |
| 68410 | Primary angiosarcoma of liver | B150200 |
| 2747 | Malignant neoplasm of cervix uteri | B41..00 |
| 74373 | DEMENTIA | 299 B |
| 44089 | Malignant neoplasm of brain stem | B517.00 |
| 70221 | [X]Other specified rheumatoid arthritis | Nyu1200 |
| 3704 | Acute subendocardial infarction | G307.00 |
| 63479 | MacLeod's unilateral emphysema | H32y200 |
| 38678 | [X]Dementia in Alzheimer's disease with late onset | Eu00100 |
| 7017 | Evacuation of intracerebral haematoma NEC | 7004300 |
| 78 | Asthma | H33..00 |
| 102946 |  |  |
| 1599 | Malignant neoplasm of kidney parenchyma | B4A0.00 |
| 105203 |  |  |
| 52511 | Malignant neoplasm of cerebral ventricles | B515.00 |
| 72174 | Malignant neoplasm of paraurethral glands | B4A4.00 |
| 86323 | MALIGNANT NEOPLASM LEG | 1959DA |
| 40068 | Presenile gangrene | G731100 |
| 34145 | Secondary malignant neoplasm of testis | B58y600 |
| 44399 | Primary malignant neoplasm of liver NOS | B150z00 |
| 61500 | Acute myelomonocytic leukaemia | B690.00 |
| 48192 | Type II diabetes mellitus with diabetic cataract | C109E11 |
| 55454 | Portal cirrhosis unspecified | J615y00 |
| 1517 | Intermittent claudication | G73z000 |
| 108775 |  |  |
| 12870 | Malignant neoplasm of main bronchus | B221.00 |
| 12987 | Late-onset asthma | H33z200 |
| 74498 | DIETARY CONTROL DIABETES | 250 DC |
| 55595 | Malignant neoplasm of sphenoid bone | B300700 |
| 28366 | Unspec duodenal ulcer; unspec haemorrhage and/or perforation | J12yy00 |
| 27617 | Malignant neoplasm of overlapping lesion of vulva | B45y000 |
| 54822 | Budgerigar-fanciers' lung | H352000 |
| 47257 | Portal cirrhosis | J615.11 |
| 94995 | Nodular lymphoma of lymph nodes of inguinal region and leg | B620500 |
| 76417 | DIABETIC DIARRHOEA | 250 DR |
| 57621 | Insulin dependent diabetes mellitus with nephropathy | C108D00 |
| 72522 | Malignant neoplasm of great vessels | B313200 |
| 15148 | Malignant neoplasm of testis | B47..00 |
| 22146 | Secondary malignant neoplasm of bladder | B581100 |
| 53504 | Malig neopl, overlap lesion brain & other part of CNS | B52W.00 |
| 103354 |  |  |
| 3530 | Peripheral vascular disease NOS | G73z.00 |
| 39899 | Malignant neoplasm of craniopharyngeal duct | B542100 |
| 42279 | Arteriosclerotic dementia NOS | E004z00 |
| 25980 | Impaired renal function disorder NOS | K08z.00 |
| 39949 | Gangrene of finger | G732200 |
| 46977 | Allergic alveolitis and pneumonitis NOS | H35z.00 |
| 99413 | Other and unspecified leukaemia NOS | B67yz00 |
| 29638 | Renal osteodystrophy | K080.00 |
| 16230 | Diabetes mellitus with neurological manifestation | C106.00 |
| 70316 | Type 2 diabetes mellitus with ophthalmic complications | C109112 |
| 59725 | Type II diabetes mellitus with ophthalmic complications | C109111 |
| 318 | Malignant neoplasm of glottis | B210.00 |
| 64345 | Malignant neoplasm of connective and soft tissue, upper arm | B311100 |
| 64556 | Chronic gastric ulcer unspecified | J111y00 |
| 58094 | Malignant neoplasm, overlapping lesion of cervix uteri | B412.00 |
| 38475 | Malignant neoplasm of other site of female breast NOS | B34yz00 |
| 15507 | Secondary and unspec malig neop lymph nodes NOS | B56z.00 |
| 9375 | Spastic paraplegia | F241100 |
| 66872 | Type I diabetes mellitus with nephropathy | C108D11 |
| 39336 | Myelosclerosis with myeloid metaplasia | B6y1.00 |
| 99670 | Unspecified peptic ulcer with obstruction | J13y400 |
| 19475 | Malignant neoplasm of descended testis | B471.00 |
| 85591 | LUPUS ERYTHEMATOSUS SYSTEMIC | 7341AA |
| 57278 | Type II diabetes mellitus with renal complications | C10F011 |
| 66089 | Other myeloid leukaemia NOS | B65yz00 |
| 83632 | CARconfidence intervalNOMA SCROTUM | 1735C |
| 106063 |  |  |
| 61399 | Malignant neoplasm of cerebral cortex | B510100 |
| 104453 |  |  |
| 64571 | Type II diabetes mellitus with nephropathy | C109C11 |
| 24836 | Type 2 diabetes mellitus with nephropathy | C109C12 |
| 63762 | Diabetes mellitus, adult onset, unspecified complication | C10z100 |
| 89350 | HEPATOBLASTOMA | 1550HB |
| 20292 | Malignant neoplasm of major salivary glands | B02..00 |
| 86996 | Malignant neoplasm of connective tissue of orbit | B501000 |
| 55552 | Other allergic alveolitis NOS | H35yz00 |
| 33344 | Myeloid leukaemia NOS | B65z.00 |
| 9565 | [X]Arteriosclerotic dementia | Eu01.11 |
| 8303 | Asbestosis | H41..00 |
| 104147 |  |  |
| 73439 | Malignant neoplasm of anterior epiglottis NOS | B064z00 |
| 44356 | Malig neop other/ill-defined sites resp/intrathoracic organs | B2z..00 |
| 7940 | [X]Non-Hodgkin's lymphoma NOS | ByuDF11 |
| 91010 | ERYTHRAEMIA CHRONIC | 208 CE |
| 8350 | Flare of rheumatoid arthritis | N040T00 |
| 100584 | Malignant neoplasm of cardiac orifice of stomach | B110000 |
| 69146 | Malignant neoplasm of bones of skull and face NOS | B300z00 |
| 99913 | Malignant neoplasm of globus pallidus | B510300 |
| 62380 | Lymphosarcoma of intrathoracic lymph nodes | B601200 |
| 61510 | Malignant neoplasm of palatoglossal arch | B062200 |
| 6960 | CVA - cerebrovascular accid due to intracerebral haemorrhage | G61..11 |
| 18604 | Stroke due to intracerebral haemorrhage | G61..12 |
| 65616 | Insulin dependent diabetes mellitus with arthropathy | C108H00 |
| 77811 | PARAPLEGIA | 344 G |
| 74113 | PARALYSIS HEMIPLEGIA | 344 B |
| 105773 |  |  |
| 105296 |  |  |
| 62305 | Malignant neoplasm of skin of buttock | B335800 |
| 92036 | [X]Occlusion and stenosis of other cerebral arteries | Gyu6600 |
| 90117 | OSTEODYSTROPHY URAEMIC | 5930AR |
| 50225 | Type II diabetes mellitus with renal complications | C109011 |
| 6872 | Aortic aneurysm NOS | G71z.00 |
| 60346 | Unspecified gastrojejunal ulcer with haemorrhage | J14y100 |
| 15424 | Secondary biliary cirrhosis | J616100 |
| 73992 | Malignant neoplasm of cornea | B504.00 |
| 15991 | Malignant neoplasm of choroid | B506.00 |
| 98813 | Malig neop eyeball excl conjunctiva, cornea, retina, choroid | B500.00 |
| 89748 | LUPUS ERYTHEMATOSUS ACUTE | 7341AD |
| 89647 | ACUTE SYSTEMIC LUPUS ERYTHEMATOSUS | 7341AC |
| 5521 | Acute peptic ulcer with perforation | J130200 |
| 107975 |  |  |
| 99386 | Malignant neoplasm posterior margin nasal septum and choanae | B073200 |
| 107166 |  |  |
| 64270 | Malignant neoplasm of skin of ankle | B337500 |
| 40962 | Non-insulin dependent d m with neuropathic arthropathy | C109H00 |
| 46578 | Panlobular emphysema | H321.00 |
| 50609 | Pre-existing diabetes mellitus, non-insulin-dependent | L180600 |
| 92543 | HISTIOCYTIC MEDULLARY RETICULOSIS | 209 HM |
| 70390 | Chronic peptic ulcer unspecified | J131y00 |
| 53594 | Malignant neoplasm of ethmoid bone | B300000 |
| 18689 | Middle cerebral artery syndrome | G660.00 |
| 4643 | Peptic ulcer symptoms | 1956 |
| 100906 | Malignant neoplasm of lip, unspecified, external | B00z000 |
| 100918 | Malignant neoplasm of anterior wall of nasopharynx NOS | B073z00 |
| 42707 | Malignant neoplasm of skin of upper arm | B336100 |
| 41958 | Malignant neoplasm of lower eyelid | B331200 |
| 79581 | NEOPLASM MALIGNANT GASTROINTESTINAL TRAC | 159 A |
| 82927 | GASTROINTESTINAL CARconfidence intervalNOMA | 159 C |
| 91492 | EPITHELIOMA CHORION TESTIS | 186 CH |
| 39084 | Malignant neoplasm of laryngopharynx | B0z2.00 |
| 16297 | Malignant neoplasm of pharynx unspecified | B0z0.00 |
| 53397 | Hodgkin's disease NOS | B61z.00 |
| 43311 | [V]Personal history of malignant neoplasm of larynx | ZV10212 |
| 61655 | [V]Personal history of malignant neoplasm - accessory sinus | ZV10211 |
| 55842 | Non-insulin-dependent diabetes mellitus with neuro comps | C109200 |
| 86691 | OLIGODENDROGLIOMA | 1929DG |
| 31053 | [D]Widespread diabetic foot gangrene | R054300 |
| 30294 | Type 1 diabetes mellitus with persistent microalbuminuria | C10EL00 |
| 17475 | Malignant neoplasm of maxilla | B300A00 |
| 56490 | Malignant neoplasm of nervous system NOS | B52z.00 |
| 50299 | Malignant neoplasm of zygomatic bone | B300900 |
| 53480 | Recurrent hepatitis | J614300 |
| 22158 | Malignant plasma cell neoplasm, extramedullary plasmacytoma | B630000 |
| 80735 | MYELOMA MULTIPLE | 203 |
| 75425 | HODGKIN'S DISEASE | 201 |
| 76395 | POLYCYTHAEMIA | 208 |
| 80877 | MYELOFIBROSIS | 209 |
| 101662 | Secondary and unspec malig neop circumflex iliac LN | B565200 |
| 68039 | Hodgkin's sarcoma of lymph nodes of axilla and upper limb | B612400 |
| 88246 | MALIGNANT NEOPLASM PHARYNX | 149 AT |
| 8933 | Left hemiplegia | F222.00 |
| 36294 | Acquired human immunodeficiency virus infection syndrome NOS | A788z00 |
| 73338 | Unspecified gastric ulcer without mention of complication | J11y000 |
| 53940 | [X]Other chronic renal failure | Kyu2100 |
| 66319 | Malignant neoplasm of skin of groin | B335500 |
| 89762 | [X]Other monocytic leukaemia | ByuD700 |
| 74306 | CARconfidence intervalNOMA BREAST | 174 C |
| 79706 | NEOPLASM MALIGNANT BREAST | 174 A |
| 102593 |  |  |
| 45700 | Neoplasms otherwise specified | By...00 |
| 73614 | Malignant neoplasm of lip unspecified, buccal aspect | B004000 |
| 89545 | MALIGNANT NEOPLASM EYE | 190 A |
| 40963 | Portal fibrosis without cirrhosis | J61y300 |
| 91360 | MALIGNANT NEOPLASM FINGER | 1959CF |
| 91331 | MALIGNANT NEOPLASM SHOULDER | 1959CB |
| 92818 | MALIGNANT NEOPLASM ARM | 1959CA |
| 44318 | Oth and unspecif peripheral & cutaneous T-cell lymphomas | B62xX00 |
| 29771 | Gastric ulcer NOS | J11z.00 |
| 6853 | Claudication | G73z011 |
| 54691 | Malignant neoplasm of lumbar vertebra | B302200 |
| 15157 | Chronic bronchitis NOS | H31z.00 |
| 27540 | Malignant neoplasm of renal calyces | B4A1000 |
| 73296 | [X]Malignant neoplasm/bones??? cartilage/limb,unspfd | Byu3100 |
| 50681 | Malignant neoplasm of prepuce (foreskin) | B480.00 |
| 100615 | Mast cell malignancy of lymph nodes inguinal region and leg | B626500 |
| 48743 | Malignant neoplasm of body of penis | B482.00 |
| 16800 | Ruptured thoracic aortic aneurysm | G711.11 |
| 100423 | Hodgkin's paragranuloma of lymph nodes of head, face, neck | B610100 |
| 33333 | Other malignant neoplasm of lymphoid and histiocytic tissue | B62..00 |
| 54793 | Subacute leukaemia NOS | B682.00 |
| 4072 | Acute leukaemia NOS | B680.00 |
| 76529 | CARconfidence intervalNOMA THYROID | 193 C |
| 83375 | THYROID MALIGNANT NEOPLASM | 193 A |
| 72725 | Malignant lymphoma NOS of intrathoracic lymph nodes | B62y200 |
| 2744 | Malignant neoplasm of uterus, part unspecified | B40..00 |
| 37872 | Malignant melanoma of lower leg | B327400 |
| 63375 | [X]Unspecified B-cell non-Hodgkin's lymphoma | ByuDE00 |
| 80802 | ADENOCARconfidence intervalNOMA UTERUS | 1830AU |
| 79549 | ADENOCARconfidence intervalNOMA OVARY | 1830AD |
| 47668 | Malignant neoplasm of tunica vaginalis | B48y100 |
| 106924 |  |  |
| 100721 | Malignant neoplasm of upper lip, inner aspect NOS | B002z00 |
| 75814 | LEUKAEMIA | 2079 |
| 40810 | Malignant neoplasm of body of pancreas | B171.00 |
| 81290 | RHABDOMYOSARCOMA URINARY BLADDER | 188 RH |
| 49701 | Malignant neoplasm of vertebral column NOS | B302z00 |
| 29178 | Hodgkin's disease, nodular sclerosis | B614.00 |
| 105388 |  |  |
| 35180 | [X]Malignant neoplasm of digestive organs | Byu1.00 |
| 58859 | Asymptomatic human immunodeficiency virus infection | A788100 |
| 39863 | [V]Personal history of malignant neoplasm of nose | ZV10214 |
| 34259 | Malignant melanoma of groin | B325300 |
| 64327 | Malignant melanoma of lower limb or hip NOS | B327z00 |
| 64116 | Secondary and unspec malig neop intrathoracic lymph nodes | B561.00 |
| 70456 | Unspecified peptic ulcer with haemorrhage | J13y100 |
| 27340 | Di Guglielmo's disease | B670.11 |
| 86046 | Malignant neoplasm of peripheral nerve of abdomen | B524400 |
| 24235 | Malig neopl peripheral nerves and autonomic nervous system | B524.00 |
| 50695 | Diffuse non-Hodgkin mixed sml & lge cell (diffuse) lymphoma | B627500 |
| 78040 | HYPERNEPHROMA | 1890CH |
| 98500 | Malignant neoplasm of upper lip, mucosa | B002200 |
| 40561 | [V]Personal history of Hodgkin's disease | ZV10711 |
| 65466 | Kaposi's sarcoma of multiple organs | B592X00 |
| 55096 | Secondary malignant neoplasm of skin NOS | B582z00 |
| 69676 | Type 1 diabetes mellitus without complication | C10EA00 |
| 57568 | Chronic pyelonephritis with medullary necrosis | K100100 |
| 42012 | Malignant neoplasm of posterior wall of urinary bladder | B494.00 |
| 105472 |  |  |
| 54267 | Malignant neoplasm of unspecified site NOS | B59z.00 |
| 62909 | Secondary malignant neoplasm of rectum | B575100 |
| 74380 | ADENOCARconfidence intervalNOMA STOMACH | 1519DA |
| 32246 | [V]Personal history of malignant neoplasm of bronchus | ZV10111 |
| 104475 |  |  |
| 33254 | Diabetes mellitus with ophthalmic manifestation | C105.00 |
| 89114 | BYSSINOSIS | 5192BY |
| 15690 | Diabetes mellitus with ketoacidotic coma | C103.00 |
| 107791 |  |  |
| 3357 | Carcinoma of digestive organs and peritoneum | B1...11 |
| 104973 |  |  |
| 31586 | Prolymphocytic leukaemia | B64y100 |
| 51818 | Malignant neoplasm of jaw NOS | B550300 |
| 95146 | Chronic kidney disease stage 2 with proteinuria | 1Z19.00 |
| 52313 | Chronic peptic ulcer with obstruction | J131400 |
| 44309 | Chronic gastric ulcer NOS | J111z00 |
| 74699 | ASTHMA EXACERBATION | 493 KB |
| 105751 |  |  |
| 89587 | Other specified viral hepatitis with hepatic coma NOS | A704z00 |
| 64267 | [X]Dementia in other specified diseases classif elsewhere | Eu02y00 |
| 106069 |  |  |
| 91896 | [X]Mal neoplasm/connective? tissue of trunk,unspecified | Byu5800 |
| 37540 | Secondary and unspec malig neop axillary lymph nodes | B563000 |
| 32174 | Malignant neoplasm of maxillary sinus | B202.00 |
| 102417 | [M]Superficial basal cell carcinoma | BB3C.00 |
| 46488 | [X]Vascular dementia of acute onset | Eu01000 |
| 41389 | Diabetes mellitus, adult onset, ophthalmic manifestation | C105100 |
| 12323 | Malignant neoplasm of lymphatic and haemopoietic tissue | B6...00 |
| 85392 | OSTEODYSTROPHY RENAL | 5930R |
| 74539 | ULCER PEPTIC DUODENUM | 5329PT |
| 88425 | OSTEODYSTROPHY AZOTAEMIC | 5930A |
| 92726 | STERNUM MALIGNANT TUMOUR | 1703A |
| 44996 | Malignant neoplasm of dome of urinary bladder | B491.00 |
| 12586 | Chronic kidney disease stage 2 | 1Z11.00 |
| 12479 | Chronic kidney disease stage 4 | 1Z13.00 |
| 65124 | Malignant neoplasm of interlobular bile ducts | B151000 |
| 96717 | Open traumatic subarachnoid haemorrhage | S621.00 |
| 95202 | ALEUKAEMIC LEUKAEMIA | 2079AL |
| 41271 | [V] Personal history of gastric ulcer | ZV12C00 |
| 22841 | Macronodular cirrhosis of liver | J615z11 |
| 1638 | Cirrhosis of liver NOS | J615z13 |
| 4250 | Leukaemia NOS | B68z.00 |
| 49758 | Malignant neoplasm of other sites lip, oral cavity, pharynx | B0zy.00 |
| 79777 | MALIGNANT NEOPLASM LIVER SECONDARY | 1977A |
| 63054 | Hodgkin's disease, nodular sclerosis NOS | B614z00 |
| 73616 | Secondary malignant neoplasm of cervix uteri | B58y200 |
| 50579 | Malignant neoplasm, overlapping lesion of larynx | B214.00 |
| 1204 | Heart attack | G30..14 |
| 86293 | MONOCYTIC LEUKAEMIA ACUTE | 2060 |
| 62146 | Non-insulin-dependent diabetes mellitus with multiple comps | C109300 |
| 41910 | Subarachnoid haemorrhage from basilar artery | G605.00 |
| 96379 | Mycosis fungoides of lymph nodes of axilla and upper limb | B621400 |
| 19492 | Coal workers' pneumoconiosis | H40..00 |
| 44805 | Malig neop of connective and soft tissue thigh and upper leg | B312100 |
| 90567 | TUMOUR TESTIS INTERSTITIAL CELL | 186 NT |
| 65704 | Type 2 diabetes mellitus with ulcer | C109412 |
| 55075 | Type II diabetes mellitus with ulcer | C109411 |
| 40292 | Malignant neoplasm of soft palate | B053.00 |
| 68114 | Phosphate-losing tubular disorders | K080000 |
| 89344 | NEOPLASM MALIGNANT URINARY TRACT | 1899T |
| 82877 | URETHRAL MALIGNANT NEOPLASM | 1899A |
| 92695 | Balfour excision of gastric ulcer | 7612111 |
| 7664 | [X]Dementia in Alzheimer's disease | Eu00.00 |
| 77821 | TONGUE CARconfidence intervalNOMA | 1419C |
| 779 | Malignant neoplasm of urinary bladder | B49..00 |
| 89206 | Massive silicotic fibrosis | H423.00 |
| 41716 | Insulin dependent diabetes mellitus with polyneuropathy | C108C00 |
| 670 | Peptic ulcer - (PU) site unspecified | J13..00 |
| 51209 | Malignant melanoma of chest wall | B325800 |
| 84651 | HYPERTENSIVE HEMIPLEGIA | 4360HP |
| 69124 | IDDM with peripheral circulatory disorder | C107300 |
| 93218 | Malignant neoplasm of gum NOS | B03z.00 |
| 104323 |  |  |
| 13099 | O/E - right eye preproliferative diabetic retinopathy | 2BBR.00 |
| 61829 | Type 1 diabetes mellitus with neurological complications | C108212 |
| 54184 | Malignant neoplasm of renal pelvis NOS | B4A1z00 |
| 49146 | Type I diabetes mellitus with neurological complications | C108211 |
| 5943 | Other peripheral vascular disease | G73..00 |
| 38639 | Bronchitis and pneumonitis due to chemical fumes | H460.00 |
| 107299 |  |  |
| 49463 | Malignant neoplasm of tarsus of eyelid | B310400 |
| 43475 | Malig neop of connective and soft tissue head, face and neck | B310.00 |
| 67949 | Malignant neoplasm of other male genital organ | B48y.00 |
| 66088 | Malig neop of connective and soft tissue of hip and leg | B312.00 |
| 4442 | Asthma unspecified | H33z.00 |
| 46792 | Malignant neoplasm of temporal lobe | B512.00 |
| 94174 | Other and unspecified leukaemia | B67y.00 |
| 15711 | Malignant neoplasm cerebrum (excluding lobes and ventricles) | B510.00 |
| 25796 | Mixed asthma | H332.00 |
| 34637 | Renal osteodystrophy NOS | K080z00 |
| 59738 | Rheumatoid arthritis of elbow | N040500 |
| 78166 | NEOPLASM MALIGNANT LYMPHOMA METASTATIC | 2022MT |
| 80498 | MALIGNANT LYMPHOMA | 2022ML |
| 31447 | Pigeon-fanciers' lung | H352100 |
| 74799 | STROKE | 4369B |
| 29519 | Systemic lupus erythematosus with organ or sys involv | N000300 |
| 36949 | Malignant neoplasm of other site of urinary bladder | B49y.00 |
| 107241 |  |  |
| 50898 | Malignant neoplasm of omentum | B18y300 |
| 37618 | Malignant neoplasm of axilla NOS | B551000 |
| 46789 | Malignant neoplasm of choroid plexus | B515000 |
| 98642 | Multiple and bilateral precerebral arterial occlusion | G633.00 |
| 96622 | Unspecified peptic ulcer with haemorrhage and perforation | J13y300 |
| 71274 | Occlusion??? of multiple and bilat cerebral arteries | G677400 |
| 11430 | Thoracoabdominal aortic aneurysm, ruptured | G715000 |
| 109002 |  |  |
| 80124 | SECONDARY NEOPLASTIC DEPOSITS LYMPH GLAN | 1969M |
| 54120 | Secondary malignant neoplasm of other part of nervous system | B584.00 |
| 105475 |  |  |
| 17326 | Subarachnoid haemorrh from intracranial artery, unspecif | G60X.00 |
| 30477 | High risk proliferative diabetic retinopathy | F420700 |
| 77661 | RENAL DISEASE | 5932A |
| 39088 | Malignant neoplasm of occipital lobe | B514.00 |
| 71150 | Unspecified duodenal ulcer without mention of complication | J12y000 |
| 12106 | [V]Personal history of malignant neoplasm | ZV10.00 |
| 34388 | Malignant neoplasm of pancreas NOS | B17z.00 |
| 67914 | Malignant neoplasm of skin of great toe | B337900 |
| 57988 | Malignant neoplasm of carpal bone - scaphoid | B305000 |
| 21715 | [X]Mesothelioma of lung | Byu5011 |
| 107881 |  |  |
| 19512 | Glycogenosis with hepatic cirrhosis | C310400 |
| 1038 | Insulin dependent diabetes mellitus | C100011 |
| 76716 | BRONCHITIS ALLERGIC | 493 A |
| 76831 | HEART ATTACK | 429 AH |
| 99719 | Insulin-dependent diabetes without complication | C10EA12 |
| 86669 | LEUKAEMIA MYELOCYTIC CHRONIC | 2051MC |
| 71810 | Malignant neoplasm of scapula and long bones of upper arm | B304.00 |
| 74790 | DIABETIC NEPHROPATHY | 250 N |
| 54083 | Letterer-Siwe disease of lymph nodes of multiple sites | B625800 |
| 75049 | ASTHMA ACUTE | 493 AA |
| 74093 | ASTHMA ATTACK | 493 AB |
| 84479 | BRONCHITIS ALLERGIC CHRONIC | 493 AC |
| 74627 | ASTHMA SEVERITY MILD | 493 AI |
| 84312 | ASTHMA SEVERITY MODERATE | 493 AJ |
| 66775 | Secondary and unspec malignant neoplasm mastoid lymph nodes | B560100 |
| 94383 | Secondary diabetes mellitus without complication | C10N000 |
| 36716 | Malignant neoplasm of floor of mouth NOS | B04z.00 |
| 64817 | Malignant neoplasm of lacrimal gland | B502.00 |
| 71904 | Unspecified duodenal ulcer with obstruction | J12y400 |
| 71031 | Reticulosarcoma of lymph nodes of head, face and neck | B600100 |
| 62814 | [V]Personal history of malig neop of gastrointestinal tract | ZV10000 |
| 76878 | CARconfidence intervalNOMA BOWEL | 1539C |
| 41941 | Rheumatoid arthritis of PIP joint of finger | N040900 |
| 21989 | Nephrotic syn,diffuse mesangiocapillary glomerulonephritis | K019.00 |
| 59152 | Malignant neoplasm of connective and soft tissue of perineum | B315200 |
| 93537 | Malignant neoplasm of midbrain | B517200 |
| 41686 | [X]Other specified diabetes mellitus | Cyu2000 |
| 65241 | Malignant neoplasm, overlapping lesion of brain | B51y200 |
| 37468 | Chronic erythraemia | B671.00 |
| 56718 | Malignant neoplasm of eyeball NOS | B500z00 |
| 105894 |  |  |
| 8335 | Asthma attack NOS | H33z111 |
| 82613 | TERATOMA | 186 TA |
| 36325 | Teratoma of undescended testis | B470300 |
| 94996 | Lung disease with systemic sclerosis | H572.00 |
| 46042 | Lambda light chain myeloma | B630300 |
| 105741 |  |  |
| 33833 | Malignant neoplasm of mandible | B301.00 |
| 19201 | Right sided intracerebral haemorrhage, unspecified | G61X100 |
| 9954 | Rheumatoid lung | H570.00 |
| 27855 | Malignant neoplasm of rectosigmoid junction | B140.00 |
| 2475 | Diabetic nephropathy | C104.11 |
| 46905 | Malignant neoplasm of coccygeal body | B545200 |
| 22135 | O/E - hemiplegia | 2833 |
| 18882 | Malignant neoplasm of overlapping lesion of lip | B006.00 |
| 101198 | Clark melanoma level 1 | 4M70.00 |
| 89892 | MALIGNANT NEOPLASM VAGINA | 1840A |
| 44324 | Acute gastric ulcer NOS | J110z00 |
| 100205 | Acute-on-chronic renal failure | K0E..00 |
| 38938 | Malignant neoplasm of pelvis, sacrum or coccyx NOS | B306z00 |
| 2890 | Malignant neoplasm of endometrium of corpus uteri | B430200 |
| 88578 | LYMPHOMA BURKITT'S | 2022BK |
| 25386 | Dementia in conditions EC | E041.00 |
| 51237 | Malignant neoplasm of rib, sternum and clavicle NOS | B303z00 |
| 101735 | Insulin-dependent diabetes mellitus with neurological comps | C10E212 |
| 71142 | Hodgkin's, lymphocytic-histiocytic predominance unspec site | B613000 |
| 37940 | Malignant neoplasm of pharyngeal recess | B072000 |
| 87524 | HEAVY-CHAIN DISEASE | 203 HC |
| 17912 | Malignant neoplasm, overlapping lesion of floor of mouth | B042.00 |
| 49276 | Insulin-dependent diabetes mellitus with ophthalmic comps | C108100 |
| 18314 | Malignant neoplasm of bone and articular cartilage | B30..00 |
| 50804 | Other impaired renal function disorder NOS | K08yz00 |
| 8634 | Multi infarct dementia | E004.11 |
| 95429 | Malignant neoplasm of posterior wall of nasopharynx | B071.00 |
| 44139 | Malignant neoplasm of anterior wall of nasopharynx | B073.00 |
| 9759 | Leaking abdominal aortic aneurysm | G718.00 |
| 48809 | Malignant neoplasm of male breast NOS | B35zz00 |
| 59755 | Hodgkin's disease NOS of intrathoracic lymph nodes | B61z200 |
| 44676 | Fatty portal cirrhosis | J615400 |
| 3514 | Hereditary spastic paraplegia | F141.00 |
| 40682 | Type 1 diabetes mellitus maturity onset | C10E900 |
| 26501 | Asthma never causes daytime symptoms | 663s.00 |
| 106911 |  |  |
| 2492 | Malignant neoplasm of skin NOS | B33z.00 |
| 102205 | Malignant neoplasm of lateral wall of nasopharynx NOS | B072z00 |
| 85817 | NOSE EXTERNAL MALIGNANT NEOPLASM | 1723A |
| 90512 | LEUKAEMIA EOSINOPHILIC | 2059E |
| 26454 | Malignant neoplasm/overlapping lesion/feml genital organs | B45X.00 |
| 87731 | MALIGNANT NEOPLASM THROAT | 149 A |
| 10079 | Right heart failure | G580.12 |
| 63995 | Malignant neoplasm of Meckel's diverticulum | B123.00 |
| 66163 | [X]2ndry?? malignant neoplasm lymph nodes/multi regions | ByuC200 |
| 6791 | Insulin dependent diabetes mellitus - poor control | C108800 |
| 75354 | ULCER STOMACH | 5319TM |
| 46917 | Type 2 diabetes mellitus with hypoglycaemic coma | C10FD00 |
| 62891 | Human immunodeficiency virus with other clinical findings | A788y00 |
| 66447 | Malignant neoplasm of skin of scapular region | B335A00 |
| 95323 | Malignant neoplasm of ectopic site of male breast | B35z000 |
| 27975 | Cerebral infarction due to embolism of cerebral arteries | G641000 |
| 72803 | Secondary and unspec malig neop intrapelvic LN NOS | B565z00 |
| 103178 |  |  |
| 58949 | Malignant neoplasm of phalanges of foot | B308D00 |
| 350 | Renal failure unspecified | K06..00 |
| 14798 | Emphysematous bronchitis | H312100 |
| 37916 | Malignant neoplasm of other specified mouth parts | B05y.00 |
| 56860 | Segmental bullous emphysema | H320000 |
| 47366 | Secondary and unspec malig neop sacral lymph nodes | B565300 |
| 41278 | Malignant melanoma of external surface of cheek | B323000 |
| 3458 | Occasional asthma | 663V000 |
| 31423 | Pneumoconiosis NOS | H45..00 |
| 67323 | Malignant neoplasm of oropharynx, other specified sites | B06y.00 |
| 34692 | Other leukaemia of unspecified cell type | B68y.00 |
| 5129 | Portal hypertension | J623.00 |
| 68611 | Secondary and unspec malig neop deep cervical LN | B560900 |
| 21232 | Allergic asthma NEC | H33zz12 |
| 18207 | Allergic bronchitis NEC | H33zz13 |
| 4606 | Exercise induced asthma | H33zz11 |
| 66444 | [X]Malignant neoplasm/overlap lesion/heart,mediastinm?? | Byu2100 |
| 8625 | Chronic lymphoid leukaemia | B641.00 |
| 76494 | METASTASIS | 1989M |
| 64971 | Malignant neoplasm of olfactory bulb | B520000 |
| 60692 | Subarachnoid haemorrhage from vertebral artery | G606.00 |
| 15205 | Nonarticular rheumatism NOS | N2z..00 |
| 66965 | Type 2 diabetes mellitus with neuropathic arthropathy | C109H12 |
| 29939 | Ruptured berry aneurysm | G600.00 |
| 109342 |  |  |
| 23389 | Malignant neoplasm of nasal cavities | B200.00 |
| 41011 | Malig neop of bone, connective tissue, skin and breast NOS | B3z..00 |
| 74500 | NOCTURNAL ASTHMA | 493 NA |
| 24989 | Oesophageal varices with bleeding | G850.00 |
| 39590 | Malignant neoplasm, overlapping lesion of accessory sinuses | B206.00 |
| 15684 | Malignant neoplasm of frontal sinus | B204.00 |
| 86943 | ADENOCARconfidence intervalNOMA CEREBELLAR METASTATIC | 1983AM |
| 54744 | Cerebral degeneration due to cerebrovascular disease | F11x200 |
| 94789 | Chronic kidney disease stage 1 with proteinuria | 1Z17.00 |
| 105069 |  |  |
| 92703 | Secondary and unspec malig neop deep parotid lymph nodes | B560400 |
| 74595 | INTERMITTENT CLAUDICATION | 4439A |
| 92211 | MEGAKARYOCYTIC LEUKAEMIA | 2079MK |
| 107052 |  |  |
| 66368 | HIV disease resulting in cytomegaloviral disease | A789100 |
| 79593 | MALIGNANCY | 1991M |
| 75114 | CARconfidence intervalNOMA | 1991C |
| 77787 | NEOPLASM MALIGNANT | 1991A |
| 43139 | Diabetes mellitus, adult onset, with hyperosmolar coma | C102100 |
| 63430 | Malignant neoplasm of endocardium | B241000 |
| 80109 | HEART FAILURE ACUTE | 7824AC |
| 43200 | Malignant neoplasm of oropharynx NOS | B06z.00 |
| 3541 | Malignant neoplasm of penis and other male genital organs | B48..00 |
| 16717 | Smokers' cough | H310100 |
| 67504 | Malignant neoplasm of lower lip, buccal aspect | B003000 |
| 12640 | Type 2 diabetes mellitus with nephropathy | C10FC00 |
| 105507 |  |  |
| 10368 | Gastric neoplasm | B11..11 |
| 49814 | Malignant melanoma of axilla | B325000 |
| 52327 | Chloroma | B653000 |
| 103796 |  |  |
| 10314 | Overlapping lesion of other and unspecified parts of mouth | B057.00 |
| 90201 | T-zone lymphoma | B62x000 |
| 70819 | Malignant neoplasm of palate unspecified | B055.00 |
| 86644 | BRONCHITIS OBSTRUCTIVE | 491 BT |
| 23707 | Acute congestive heart failure | G580000 |
| 28559 | Malignant neoplasm of palate NOS | B055z00 |
| 102734 |  |  |
| 67703 | Hodgkin's disease, lymphocytic depletion | B616.00 |
| 67806 | Malignant melanoma of face NOS | B323z00 |
| 18230 | Type 1 diabetes mellitus with neuropathic arthropathy | C108J12 |
| 60208 | Type I diabetes mellitus with neuropathic arthropathy | C108J11 |
| 93715 | [X]Other seropositive rheumatoid arthritis | Nyu1100 |
| 884 | Left ventricular failure | G581.00 |
| 37016 | Malignant neoplasm of sebaceous gland | B33..14 |
| 40443 | Malignant neoplasm of sweat gland | B33..15 |
| 5034 | Epithelioma | B33..12 |
| 78995 | MALIGNANT ASconfidence intervalTES | 1976A |
| 33438 | Chronic duodenal ulcer without mention of complication | J121000 |
| 87408 | PERITONITIS MALIGNANT | 1976M |
| 23580 | Subarachnoid haemorrhage NOS | G60z.00 |
| 60312 | Malignant neoplasm other gallbladder/extrahepatic bile duct | B16y.00 |
| 44779 | Type 2 diabetes mellitus with diabetic cataract | C109E12 |
| 34742 | Malignant neoplasm of pleura NOS | B23z.00 |
| 11009 | Malig neop oth/ill-defined sites digestive tract/peritoneum | B1z..00 |
| 92068 | Nodular lymphoma of intra-abdominal lymph nodes | B620300 |
| 107126 |  |  |
| 98626 | Secondary and unspec malig neop supratrochlear lymph nodes | B563100 |
| 75914 | ADENOCARconfidence intervalNOMA | 1991AD |
| 41049 | Type 1 diabetes mellitus with retinopathy | C108712 |
| 38161 | Type I diabetes mellitus with retinopathy | C108711 |
| 51255 | Malignant neoplasm of digestive tract and peritoneum NOS | B1zz.00 |
| 103902 |  |  |
| 8363 | Oesophageal varices in alcoholic cirrhosis of the liver | G852300 |
| 23688 | Operations on gastric ulcer | 761J.00 |
| 50974 | Malignant neoplasm rectum,rectosigmoid junction and anus NOS | B14z.00 |
| 43548 | Malignant neoplasm of postcricoid region | B080.00 |
| 106613 |  |  |
| 88252 | MALIGNANT NEOPLASM BILE/BILIARY TRACT | 1569A |
| 54899 | Type II diabetes mellitus with peripheral angiopathy | C109F11 |
| 60699 | Type 2 diabetes mellitus with peripheral angiopathy | C109F12 |
| 56939 | Hypokalaemic nephropathy | K08y000 |
| 67797 | Secondary and unspec malig neop superfic tracheobronchial LN | B561600 |
| 72702 | Insulin dependent diabetes mellitus - poor control | C10E812 |
| 105337 |  |  |
| 38146 | Asthma disturbs sleep weekly | 663N100 |
| 50858 | Acute panmyelosis | B674.00 |
| 80238 | LARYNX CARconfidence intervalNOMA | 1619C |
| 88264 | NEOPLASM MALIGNANT LARYNX | 1619A |
| 54186 | Malignant neoplasm of diaphragm | B313100 |
| 33499 | Pure motor lacunar syndrome | G665.00 |
| 20159 | Secondary and unspec malig neop lymph nodes multiple sites | B56y.00 |
| 30655 | Oesophageal varices without bleeding | G851.00 |
| 92014 | MALIGNANT NEOPLASM EYELID | 1731AL |
| 8550 | Malignant neoplasm of pituitary gland | B542000 |
| 88362 | Malignant neoplasm of other specified hypopharyngeal site | B08y.00 |
| 27664 | Acute promyelocytic leukaemia | B65y100 |
| 4865 | Oesophageal cancer | B10z.11 |
| 92329 | Malignant neoplasm of other male genital organ NOS | B48yz00 |
| 22752 | Occupational asthma | 173c.00 |
| 27563 | Thoracic aortic aneurysm which has ruptured | G711.00 |
| 53669 | Unspecified duodenal ulcer | J12y.00 |
| 9444 | [V]Personal history of malignant neoplasm of genital organ | ZV10400 |
| 12389 | Malignant neoplasm of renal pelvis | B4A1.00 |
| 54133 | Malignant neoplasm of cerebrum NOS | B510z00 |
| 52316 | Malignant neoplasm of pelvis | B553.00 |
| 50222 | Malignant neoplasm of connective and soft tissue of shoulder | B311000 |
| 26393 | Malignant neoplasm of liver unspecified | B152.00 |
| 26861 | Asthma sometimes restricts exercise | 6.63E+02 |
| 79014 | DEMENTIA ARTERIOSCLEROTIC | 2930 |
| 46175 | Flaccid paraplegia | F241000 |
| 86744 | HAEMORRHAGE INTRACRANIAL | 4319CR |
| 84283 | LEUKAEMIA HAIRY CELL TYPE | 2062 |
| 34878 | Malignant neoplasm of medial cuneiform | B308300 |
| 108389 |  |  |
| 65233 | Malig neop connective and soft tissue other specified site | B31y.00 |
| 88746 | CARconfidence intervalNOMA MOUTH CHEEK INTERNAL | 1450C |
| 107973 |  |  |
| 68480 | Malignant neoplasm of nipple of male breast | B350000 |
| 105784 |  |  |
| 109103 |  |  |
| 18676 | Pathological fracture due to metastatic bone disease | B585000 |
| 94427 | Malignant neoplasm of fifth metacarpal bone | B305C00 |
| 58196 | Intrinsic asthma with status asthmaticus | H331100 |
| 88659 | MESOBLASTIC NEPHROMA | 1890MB |
| 80152 | POLYCYTHAEMIA RUBRA VERA | 208 RV |
| 63657 | Malignant neoplasm of conjunctiva | B503.00 |
| 12555 | Generalised ischaemic cerebrovascular disease NOS | G671z00 |
| 34451 | Malignant neoplasm of connective and other soft tissue | B31..00 |
| 51115 | Malignant neoplasm of spinal cord | B522.00 |
| 18354 | Malignant neoplasm of other specified skin sites | B33y.00 |
| 86558 | EPENDYMOMA | 1929EP |
| 4024 | Heart failure NOS | G58z.00 |
| 49289 | [V]Personal history of malig neop of trachea/bronchus/lung | ZV10100 |
| 2062 | Heart failure | G58..00 |
| 22894 | Malignant neoplasm of cardio-oesophageal junction of stomach | B110100 |
| 55601 | Myopathy due to scleroderma | F396600 |
| 72445 | Malignant neoplasm of cystic duct | B161000 |
| 107440 |  |  |
| 59534 | H/O: Peripheral vascular disease procedure | 14NB.00 |
| 71238 | Lymphosarcoma of lymph nodes of head, face and neck | B601100 |
| 93842 | Malignant neoplasm of palatopharyngeal arch | B062300 |
| 53884 | Malignant neoplasm tonsil NOS | B060z00 |
| 3230 | Cervical carcinoma (uterus) | B41..11 |
| 92898 | LEUKAEMIC ADENIA | 2049AD |
| 67339 | [M] Malignant mastocytosis | BBp2.00 |
| 103883 |  |  |
| 59223 | Malignant neoplasm of ischium | B306100 |
| 44615 | Secondary malignant neoplasm of ovary | B586.00 |
| 65106 | Malignant neoplasm of uterine adnexa NOS | B44z.00 |
| 4944 | Multiple myeloma | B630.00 |
| 43346 | [X]Primary degen dementia of Alzheimer's type, senile onset | Eu00113 |
| 11379 | [X]Senile dementia,Alzheimer's type | Eu00112 |
| 90572 | [X]Occlusion and stenosis of other precerebral arteries | Gyu6500 |
| 68066 | Other chronic bronchitis NOS | H31yz00 |
| 63104 | Malignant neoplasm of orbit NOS | B501z00 |
| 107886 |  |  |
| 5141 | Congestive cardiomyopathy | G554000 |
| 54747 | Malignant neoplasm of parietal bone | B300600 |
| 6333 | Prepyloric ulcer | J11..11 |
| 3101 | Pyloric ulcer | J11..12 |
| 5638 | Primary biliary cirrhosis | J616000 |
| 59831 | Malignant neoplasm of nipple or areola of female breast NOS | B340z00 |
| 22893 | Malignant neoplasm of oropharynx | B06..00 |
| 82815 | POLYMYOSITIS | 7161 |
| 104505 |  |  |
| 45304 | Acute peptic ulcer with haemorrhage and perforation | J130300 |
| 105020 |  |  |
| 899 | Hepatitis unspecified | J633.00 |
| 33682 | Malignant neoplasm of skin of lower leg | B337400 |
| 64515 | [X]Diffuse non-Hodgkin's lymphoma, unspecified | ByuDC00 |
| 16725 | Cirrhosis - non alcoholic | J615.00 |
| 21698 | Malignant neoplasm of main bronchus NOS | B221z00 |
| 82325 | CYLINDROMA | 1991NC |
| 64567 | Other immunoproliferative neoplasms | B63y.00 |
| 53634 | [D]Gangrene of toe in diabetic | R054200 |
| 69761 | Malignant neoplasm of lip, vermilion border NOS | B00zz00 |
| 31564 | Lung disease with systemic lupus erythematosus | H57y400 |
| 55550 | Malignant neoplasm of upper eyelid | B331100 |
| 29283 | Malignant neoplasm of other site of respiratory tract | B2zy.00 |
| 18618 | Malignant neoplasm of skin of abdominal wall | B335300 |
| 94894 | Bauxite fibrosis of lung | H431.00 |
| 74883 | LYMPHOCYTIC LEUKAEMIA | 2049 |
| 50429 | Non-insulin-dependent diabetes mellitus with ophthalm comps | C109100 |
| 66384 | Malignant neoplasm of lower lip, external | B001000 |
| 35364 | Secondary malignant neoplasm of retroperitoneum | B576000 |
| 30323 | Type 1 diabetes mellitus with persistent proteinuria | C10EK00 |
| 35904 | Neoplasms NOS | Bz...00 |
| 62854 | [X]Human immunodeficiency virus disease | AyuC.00 |
| 1755 | Chronic aggressive hepatitis | J614200 |
| 37750 | [D]Gangrene NOS | R054z00 |
| 86638 | CHLOROMA | 2022CL |
| 10995 | Malignant neoplasm of other and unspecified sites | B5...00 |
| 49292 | [X]Malignant neoplsm/ill-defin sites within digestive system | Byu1300 |
| 57047 | Malignant neoplasm of carotid body | B544.00 |
| 18777 | Type 2 diabetes mellitus with renal complications | C10F000 |
| 67107 | Malignant neoplasm of parietal pleura | B230.00 |
| 77614 | NEPHRITIS | 583 A |
| 10659 | Diabetic cataract | F464000 |
| 65782 | Malignant neoplasm of skin of toe | B337800 |
| 56893 | Chron neph syn difus mesangial prolifrtiv glomerulonephritis | K0A3300 |
| 53945 | [X]Other acute renal failure | Kyu2000 |
| 719 | H/O: asthma | 14B4.00 |
| 43415 | [X]Other Hodgkin's disease | ByuD000 |
| 67748 | Malignant neoplasm of skin of umbilicus | B335400 |
| 6115 | Myeloproliferative disorder | B6y0.00 |
| 59365 | Non-insulin dependent diabetes mellitus with nephropathy | C109C00 |
| 12736 | Type 2 diabetes mellitus with gangrene | C10F500 |
| 8206 | Pigmentary cirrhosis of liver | C350012 |
| 79726 | ADENOCARconfidence intervalNOMA PROSTATE | 185 CA |
| 81669 | DIABETIC GLOMERULOSCLEROSIS | 250 LG |
| 59097 | Malignant neoplasm of lower uterine segment | B431000 |
| 67709 | Malt workers' lung | H354.00 |
| 102116 | Clark melanoma level 4 | 4M73.00 |
| 78210 | RETINOBLASTOMA | 190 B |
| 81291 | RHABDOMYOSARCOMA | 1719RH |
| 91840 | RHABDOMYOSARCOMA BUTTOCK | 1719RB |
| 79058 | CARconfidence intervalNOMA BRONCHUS | 1621CB |
| 92490 | ERYTHROBLASTOMA | 208 BL |
| 60249 | Unspecified peptic ulcer NOS | J13yz00 |
| 58601 | Malignant neoplasm of skin of thigh | B337100 |
| 8649 | [X]Non-Hodgkin's lymphoma, unspecified type | ByuDF00 |
| 88884 | SARCOMA URINARY BLADDER | 188 RC |
| 50668 | Diffuse non-Hodgkin's small cell (diffuse) lymphoma | B627300 |
| 71946 | Malignant neoplasm of mastoid air cells | B201300 |
| 39531 | Malig neo, overlapping lesion of heart, mediastinum & pleura | B25..00 |
| 29284 | [V]Personal history of malignant neoplasm of lung | ZV10112 |
| 93468 | Type 1 diabetes mellitus with peripheral angiopathy | C10EG00 |
| 74944 | EXACERBATION OF ASTHMA | 493 KA |
| 5637 | Malignant neoplasm of thyroid gland | B53..00 |
| 105072 |  |  |
| 5051 | Intracerebral haemorrhage | G61..00 |
| 69392 | Secondary and unspec malig neop inferior tracheobronchial LN | B561700 |
| 103047 |  |  |
| 74564 | RHEUMATISM | 718 |
| 6155 | Stroke due to cerebral arterial occlusion | G64..13 |
| 69766 | HIV infection with persistent generalised lymphadenopathy | A788200 |
| 101978 | [M]Grade 3 (Stage pTa) papillary urothelial/transit cell ca | BB4D.00 |
| 101700 | Hereditary nonpolyposis colon cancer | B139.00 |
| 28311 | Malignant neoplasm of cervix uteri NOS | B41z.00 |
| 105957 |  |  |
| 9835 | O/E - diabetic maculopathy present both eyes | 2BBL.00 |
| 108182 |  |  |
| 35325 | [X]Malignant neoplasm of respiratory and intrathoracic orga | Byu2.00 |
| 18608 | Malig neop of bone, connective tissue, skin and breast | B3...00 |
| 90610 | Malignant neoplasm of upper lip, oral aspect | B002300 |
| 1916 | Senile dementia | E00..11 |
| 63997 | Malignant melanoma of thumb | B326500 |
| 101606 | Subacute monocytic leukaemia | B662.00 |
| 28727 | Secondary malignant neoplasm of colon | B575000 |
| 55946 | Secondary malignant neoplasm of duodenum | B574000 |
| 21327 | Malignant neoplasm of skin of temple | B333500 |
| 42023 | Malignant neoplasm of urachus | B497.00 |
| 73697 | Unspecified gastric ulcer with obstruction | J11y400 |
| 41571 | Malignant neoplasm of bladder neck | B495.00 |
| 47816 | Type II diabetes mellitus with neuropathic arthropathy | C109H11 |
| 37959 | Fetid chronic bronchitis | H311100 |
| 16915 | Malignant neoplasm of intrahepatic bile ducts | B151.00 |
| 22050 | Chronic myelomonocytic leukaemia | B691.00 |
| 70747 | Secondary and unspec malig neop of inguinal and leg LN NOS | B564z00 |
| 20170 | Pancoast's syndrome | B222.11 |
| 1056 | Malignant neoplasm of other and unspecified site NOS | B5z..00 |
| 16075 | Malignant neoplasm of bone and articular cartilage NOS | B30z.00 |
| 104480 |  |  |
| 105100 |  |  |
| 90899 | REPAIR PERFORATED PEPTIC ULCER | K458 PT |
| 72464 | Malignant neoplasm of metacarpal bones | B305.12 |
| 33450 | Emphysema NOS | H32z.00 |
| 31016 | [X]Mixed cortical and subcortical vascular dementia | Eu01300 |
| 69748 | Diabetes mellitus, juvenile type, ophthalmic manifestation | C105000 |
| 50285 | Malignant neoplasm of endocervix NOS | B410z00 |
| 104934 |  |  |
| 105709 |  |  |
| 12006 | Mycosis fungoides | B621.00 |
| 27520 | Chronic myeloid leukaemia NOS | B651z00 |
| 79322 | BRONCHITIS RECURRENT | 491 R |
| 106349 |  |  |
| 67905 | Type II diabetes mellitus with neurological complications | C109211 |
| 45919 | Type 2 diabetes mellitus with neurological complications | C109212 |
| 102620 |  |  |
| 3243 | Chronic bronchitis | H31..00 |
| 47286 | Malignant neoplasm of thorax | B551.00 |
| 22187 | Hepatocellular carcinoma | B150300 |
| 70736 | Secondary malignant neoplasm of vagina | B58y300 |
| 31399 | Malignant neoplasm of lower limb NOS | B555.00 |
| 104954 |  |  |
| 18324 | Acute duodenal ulcer with perforation | J120200 |
| 10949 | Malignant neoplasm of ampulla of Vater | B162.00 |
| 48820 | Malignant neoplasm of endocervix | B410.00 |
| 55313 | [X]Other vascular dementia | Eu01y00 |
| 33807 | Diabetes mellitus, adult with gangrene | C107200 |
| 85275 | MOLE (SKIN) MALIGNANT | 1729L |
| 83674 | MALIGNANT MELANOMA SKIN | 1729B |
| 72713 | Secondary and unspec malig neop superficial mesenteric LN | B562100 |
| 18654 | Chronic gastric ulcer | J111.00 |
| 15097 | Chronic glomerulonephritis NOS | K02z.00 |
| 54965 | Malig neop connective and soft tissue of popliteal space | B312200 |
| 44529 | Secondary malignant neoplasm of large intestine and rectum | B575.00 |
| 11833 | Hypersensitivity pneumonitis NOS | H35z100 |
| 7191 | Asthma limiting activities | 663P.00 |
| 95259 | MALIGNANT NEOPLASM THORAX/THORAconfidence intervalC WALL | 1959BA |
| 91612 | MALIGNANT NEOPLASM CHEST WALL | 1959BB |
| 91407 | MALIGNANT NEOPLASM GROIN | 1959BD |
| 47801 | Malignant neoplasm, overlapping lesion of bladder | B49y000 |
| 30310 | Nephrogenic diabetes insipidus | K081.00 |
| 74334 | AORTIC ANEURYSM | 4419 |
| 5005 | Pleural plaque disease due to asbestosis | H410.00 |
| 101753 | Malignant neoplasm of other sites of gum | B03y.00 |
| 54679 | Secondary malignant neoplasm of unknown site | B594.00 |
| 29472 | Giant cell arteritis with polymyalgia rheumatica | N200.00 |
| 20007 | Disseminated lupus erythematosus | N000000 |
| 17085 | Muscular rheumatism | N240200 |
| 46159 | Malignant neoplasm of cloacogenic zone | B142000 |
| 83014 | DIABETIC CATARACT | 250 CT |
| 63001 | Anti-platelet induced gastric ulcer | J112.00 |
| 29160 | Malignant neoplasm of connective and soft tissue of axilla | B313000 |
| 91712 | SARCOMA DUODENUM | 1520B |
| 88103 | MALIGNANT NEOPLASM DUODENUM | 1520A |
| 43816 | Rheumatoid carditis | G5yA.00 |
| 85400 | LEUKAEMIA GRANULOCYTIC CHRONIC | 2051GR |
| 62584 | Secondary malignant neoplasm of other respiratory organs | B573.00 |
| 26165 | Malignant neoplasm of supraglottis | B211.00 |
| 52736 | Secondary and unspec malig neop intra-abdominal lymph nodes | B562.00 |
| 31369 | Acute renal medullary necrosis | K042.00 |
| 94965 | Chronic kidney disease stage 3A | 1Z15.00 |
| 86462 | NEPHROBLASTOMA | 1890BL |
| 72204 | [V]Personal history other lymphatic/haematopoietic neoplasm | ZV10700 |
| 51239 | Rheumatoid arthritis of ankle | N040F00 |
| 31700 | Malignant neoplasm of upper lobe bronchus | B222000 |
| 69104 | Malignant neoplasm of carpal bone - lunate | B305100 |
| 95571 | CKD stage 3A with proteinuria | 1Z1D.11 |
| 84359 | HEPATOMA | 1550B |
| 23461 | Pneumoconiosis due to inorganic dust NOS | H43z.00 |
| 28109 | Inflammatory abdominal aortic aneurysm | G714100 |
| 58791 | Lung disease with diseases EC | H57y.00 |
| 86718 | LINITIS PLASTICA | 1519CL |
| 30235 | Siderosis | H434.00 |
| 49655 | Type II diabetes mellitus with retinopathy | C10F611 |
| 4325 | Other specified peripheral vascular disease NOS | G73yz00 |
| 34409 | Malignant neoplasm of base of tongue dorsal surface | B010000 |
| 97979 | CKD stage 2 with proteinuria | 1Z19.11 |
| 67884 | Malignant neoplasm of areola of male breast | B350100 |
| 102177 | Acute gastrojejunal ulcer with perforation | J140200 |
| 41144 | Secondary malignant neoplasm of skin of trunk | B582300 |
| 68055 | Malignant neoplasm of long bones of leg | B307.00 |
| 73530 | Malignant neoplasm of hand bones | B305.00 |
| 45667 | Malignant neoplasm of orbit | B501.00 |
| 27759 | [X] Senile dementia, depressed or paranoid type | Eu02z16 |
| 4357 | [X] Senile dementia NOS | Eu02z14 |
| 93922 | Diabetes mellitus, juvenile type, with renal manifestation | C104000 |
| 3670 | Scleroderma | N001.00 |
| 66488 | Malig neop of connective and soft tissue of abdominal wall | B314000 |
| 81147 | MALIGNANT NEOPLASM SPINE | 1702P |
| 106528 |  |  |
| 12499 | [X]Malignant neoplasm of breast | Byu6.00 |
| 92380 | Burkitt's lymphoma of lymph nodes of inguinal region and leg | B602500 |
| 65215 | Malignant neoplasm of sphenoidal sinus | B205.00 |
| 12229 | Acute ST segment elevation myocardial infarction | G30X000 |
| 7484 | Mesothelioma | B226.00 |
| 15788 | Transient cerebral ischaemia NOS | G65zz00 |
| 10062 | Cerebrovascular disease NOS | G6z..00 |
| 21329 | Plasmacytoma NOS | B630200 |
| 77042 | RHEUMATISM MUSCULAR | 7179G |
| 45867 | Renal medullary necrosis unspecified | K035.00 |
| 71784 | Rheumatoid arthritis of other tarsal joint | N040J00 |
| 28069 | Malignant neoplasm of retina | B505.00 |
| 77466 | METASTASIS LUNG | 1970M |
| 100253 | Xanthomatous portal cirrhosis | J615C00 |
| 31724 | Rheumatoid lung | N04y000 |
| 86743 | MALIGNANT TERATOMA | 186 TM |
| 71584 | Malignant neoplasm of lacrimal duct | B507.00 |
| 27416 | Lymphosarcoma | B601.00 |
| 45262 | [X]Malignant neoplasm of male genital organ, unspecified | Byu8200 |
| 94975 | Malignant neoplasm of pericardium | B241300 |
| 87789 | LYMPHOBLASTIC LYMPHOSARCOMA | 2001BL |
| 61344 | Type I diabetes mellitus with renal complications | C108011 |
| 80524 | METASTASIS LIVER | 1977M |
| 42566 | Malignant neoplasm of lower lobe, bronchus or lung NOS | B224z00 |
| 97746 | Hodgkin's disease NOS of lymph nodes of multiple sites | B61z800 |
| 100210 | Gastrointestinal stromal tumour | B905400 |
| 95121 | Chronic kidney disease stage 2 without proteinuria | 1Z1A.00 |
| 44169 | Malignant neoplasm of upper lobe, bronchus or lung NOS | B222z00 |
| 88742 | OLIGODENDROBLASTOMA | 1929D |
| 46939 | Malignant neoplasm of cervical vertebra | B302000 |
| 68330 | Hodgkin's, lymphocytic-histiocytic pred of head, face, neck | B613100 |
| 102244 | [M]Grade 2 (Stage pTa) papillary urothelial/transit cell ca | BB4C.00 |
| 79265 | SQUAMOUS CELL CARconfidence intervalNOMA SKIN | 1739CM |
| 19354 | Other transient cerebral ischaemia | G65y.00 |
| 37165 | Malignant neoplasm of scalp | B334000 |
| 52283 | Insulin-dependent diabetes mellitus with neurological comps | C108200 |
| 104128 |  |  |
| 76073 | SYSTEMIC LUPUS ERYTHEMATOSUS | 6954 |
| 105151 |  |  |
| 83711 | LYMPHOMA LEUKAEMIC | 2022LK |
| 102708 |  |  |
| 95049 | Hodgkin's lymphocytic depletion of unspecified site | B616000 |
| 59778 | Hodgkin's disease NOS of lymph nodes of head, face and neck | B61z100 |
| 62630 | Malignant neoplasm of long bones of leg NOS | B307z00 |
| 47377 | Other specified diabetes mellitus with ophthalmic complicatn | C105y00 |
| 104743 |  |  |
| 26855 | Unstable insulin dependent diabetes mellitus | C108400 |
| 89982 | MALIGNANT NEOPLASM AUDITORY CANAL | 1732AC |
| 65880 | Malig neop of scapula and long bones of upper arm NOS | B304z00 |
| 35535 | Malignant neoplasm of pancreatic duct | B173.00 |
| 22023 | Diabetic - poor control NOS | 66AJz00 |
| 62182 | Malignant neoplasm of vestibule of nose | B200300 |
| 71763 | [X]Other forms of systemic sclerosis | Nyu4500 |
| 81889 | ADENOLYMPHOMA | 2029AL |
| 90583 | ANTHRACOSILICOSIS | 5151 |
| 74630 | ASBESTOSIS | 5152 |
| 97875 | Malignant neoplasm, overlapping lesion of pancreas | B175.00 |
| 97091 | [X]2ndry malignant neoplasm/bladder??? urinary organs | ByuC500 |
| 44982 | Type 2 diabetes mellitus with diabetic cataract | C10FE00 |
| 84227 | REPAIR PERFORATED GASTRIC ULCER | K4271 |
| 6707 | Extrinsic asthma with asthma attack | H330111 |
| 40437 | Malignant neoplasm of other specified site of eye | B50y.00 |
| 4513 | Non-insulin-dependent diabetes mellitus | C109.00 |
| 46114 | Malig neop other/ill-defined sites lip, oral cavity, pharynx | B0z..00 |
| 70026 | Secondary malig neop of small intestine or duodenum NOS | B574z00 |
| 108667 |  |  |
| 64710 | Resection of gastric ulcer by cautery | 7612500 |
| 65458 | Malig neop of other and unspecified parts of nervous system | B52..00 |
| 24490 | Diabetes mellitus, juvenile type, no mention of complication | C100000 |
| 100769 | [X]Unspecified human immunodeficiency virus [HIV] disease | AyuCD00 |
| 108682 |  |  |
| 71204 | Malignant neoplasm of cartilage of nose | B200000 |
| 47321 | Type 2 diabetes mellitus with ophthalmic complications | C10F100 |
| 23361 | Late effects of cerebrovascular disease | G68..00 |
| 71136 | Malignant melanoma of chin | B323100 |
| 1682 | Diabetes mellitus with ketoacidosis | C101.00 |
| 8771 | Malignant neoplasm of head of pancreas | B170.00 |
| 69497 | Malignant histiocytosis of unspecified site | B623000 |
| 49605 | Hodgkin's disease, mixed cellularity | B615.00 |
| 96585 | Overlapping malignant melanoma of skin | B32y000 |
| 512 | Chronic renal failure | K05..00 |
| 85480 | CARconfidence intervalNOMA MOUTH FLOOR | 144 C |
| 74441 | PVD (PERIPHERAL VASCULAR DISEASE) | 4439GD |
| 3540 | C/O bronchial catarrh | 1761 |
| 61643 | Malignant neoplasm of intrahepatic bile ducts NOS | B151z00 |
| 84305 | CARconfidence intervalNOMA ANUS | 1736CN |
| 30646 | Malignant neoplasm lymphatic or haematopoietic tissue OS | B6y..00 |
| 43715 | Malignant melanoma of umbilicus | B325600 |
| 20113 | Subcutaneous emphysema | SK07.00 |
| 63896 | Secondary malignant neoplasm of skin of shoulder and arm | B582400 |
| 61246 | Malignant melanoma of heel | B327600 |
| 94407 | Hodgkin's mixed cellularity of lymph nodes head, face, neck | B615100 |
| 65253 | Secondary and unspec malignant neoplasm occipital lymph node | B560300 |
| 41530 | Malignant neoplasm of other sites of tongue | B01y.00 |
| 105038 |  |  |
| 37006 | HIV disease resulting in mycobacterial infection | A789000 |
| 104328 |  |  |
| 45922 | Malignant neoplasm, overlapping lesion of eye and adnexa | B508.00 |
| 86966 | CARconfidence intervalNOMA URETHRA | 1899C |
| 78030 | PRIMARY BILIARY confidence intervalRRHOSIS (LIVER) | 5719PB |
| 77891 | PORTAL HYPERTENSION | 5719PH |
| 33271 | Malignant neoplasm of pinna NEC | B332200 |
| 2418 | Cerebrovascular disease | G6...00 |
| 22905 | Interstitial emphysema | H581.00 |
| 91992 | LEUKAEMIA PLASMA CELL | 2079PC |
| 12585 | Chronic kidney disease stage 5 | 1Z14.00 |
| 19389 | Malig neop of bone, connective tissue, skin and breast OS | B3y..00 |
| 51238 | Rheumatoid arthritis of 1st MTP joint | N040K00 |
| 11035 | Primary malignant neoplasm of unknown site | B593.00 |
| 88243 | MALIGNANT NEOPLASM VULVA | 1841A |
| 82106 | CARconfidence intervalNOMA VULVA | 1841C |
| 105392 |  |  |
| 19144 | [X]Melanoma and other malignant neoplasms of skin | Byu4.00 |
| 32768 | Malignant melanoma of breast | B325100 |
| 87195 | TONGUE TUMOUR MALIGNANT | 1419A |
| 49400 | Malignant neoplasm of endometrium | B430211 |
| 67396 | Secondary malig neop of retroperitoneum and peritoneum | B576.00 |
| 99015 | Other monocytic leukaemia | B66y.00 |
| 39629 | Granulocytic sarcoma | B653100 |
| 5931 | H/O: dementia | 1461 |
| 8281 | HIV disease resulting in wasting syndrome | A789A00 |
| 22573 | Diabetes mellitus NOS with neurological manifestation | C106z00 |
| 29013 | Chronic kidney disease stage 1 | 1Z10.00 |
| 50972 | Diabetes mellitus NOS with no mention of complication | C100z00 |
| 4413 | Acute myeloid leukaemia | B650.00 |
| 82396 | CARconfidence intervalNOMA VOCAL CORD | 1619CV |
| 12566 | Chronic kidney disease stage 3 | 1Z12.00 |
| 32671 | Chronic congestive heart failure | G580100 |
| 8195 | [X]Alzheimer's dementia unspec | Eu00z11 |
| 74590 | PIGEON FANconfidence intervalER'S LUNG | 114 PF |
| 63475 | Subacute myeloid leukaemia | B652.00 |
| 104788 |  |  |
| 29735 | Osteoma | B30..12 |
| 5062 | Chondroma | B30..11 |
| 36371 | Malignant neoplasm of overlapping lesion of bronchus & lung | B225.00 |
| 38918 | Secondary malignant neoplasm of spinal cord | B583100 |
| 45986 | Malignant neoplasm of lateral portion of floor of mouth | B041.00 |
| 42856 | Malignant neoplasm of nasal cavities NOS | B200z00 |
| 45139 | Malignant melanoma of external surface of nose | B323400 |
| 21837 | Hypertensive heart&renal dis wth (congestive) heart failure | G232.00 |
| 88261 | MALIGNANT NEOPLASM LIVER | 1550A |
| 79876 | CARconfidence intervalNOMA LIVER | 1550C |
| 39430 | Malignant neoplasm of lip, oral cavity and pharynx NOS | B0zz.00 |
| 34135 | H/O: CVA/stroke | 14A7.00 |
| 43111 | Malignant neoplasm of laryngeal cartilage | B213.00 |
| 9204 | Peripheral gangrene | G732.00 |
| 105317 |  |  |
| 34075 | Malig neop of respiratory tract and intrathoracic organs | B2...00 |
| 55374 | Malignant neoplasm of epiglottis NOS | B215.00 |
| 29462 | Malignant neoplasm of kidney or urinary organs NOS | B4Az.00 |
| 64283 | Other specified diabetes mellitus with unspecified comps | C10zy00 |
| 76438 | WHEEZING BRONCHIAL | 7832AB |
| 104412 |  |  |
| 66327 | Nodular lymphoma of unspecified site | B620000 |
| 352 | Duodenal ulcer - (DU) | J12..00 |
| 27603 | Rheumatoid arthritis and other inflammatory polyarthropathy | N04..00 |
| 92602 | SYMMETRICAL GANGRENE EXTREMITIES | 4430G |
| 27964 | Acute heart failure | G582.00 |
| 398 | Congestive heart failure | G580.00 |
| 73760 | Malignant neoplasm of scalp or skin of neck NOS | B334z00 |
| 70696 | Malignant neoplasm of other major salivary glands | B02y.00 |
| 74458 | LATE ONSET ASTHMA | L4930LO |
| 19519 | Asthma treatment compliance unsatisfactory | 663p.00 |
| 13572 | Ruptured abdominal aortic aneurysm | G713.11 |
| 68390 | Type 1 diabetes mellitus with ulcer | C108512 |
| 60805 | Talc pneumoconiosis | H420.00 |
| 87374 | POSTCRICOID CARconfidence intervalNOMA | 1480C |
| 94873 | [M]Squamous cell carcinoma of skin NOS | BB2A.13 |
| 41017 | Aspirin induced asthma | 1780 |
| 55659 | Malig neop other site rectum, rectosigmoid junction and anus | B14y.00 |
| 11175 | [X]Multi-infarct dementia | Eu01100 |
| 2755 | Cancers | B....11 |
| 31268 | Malignant neoplasm of middle lobe, bronchus or lung | B223.00 |
| 11680 | O/E - gangrene | 2I16.00 |
| 30815 | Asthma causing night waking | 663N000 |
| 61122 | Diabetes mellitus induced by non-steroid drugs | C10H.00 |
| 65701 | Nodular lymphoma NOS | B620z00 |
| 105335 |  |  |
| 63224 | Malignant neoplasm of penis and other male genital organ NOS | B48z.00 |
| 2760 | Peripheral vascular disease NOS | G73zz00 |
| 20677 | Closure of perforated gastric ulcer | 761J000 |
| 10851 | Cerebral tumour - malignant | B51..11 |
| 55758 | Bronchitis and pneumonitis due to chemical fumes NOS | H460z00 |
| 58903 | Malignant neoplasm of head, neck and face NOS | B550z00 |
| 71897 | Chronic gastric ulcer with haemorrhage and perforation | J111300 |
| 44267 | Malignant histiocytosis | B623.00 |
| 30542 | Malig neop of connective and soft tissue of lower leg | B312300 |
| 27790 | Chronic lymphatic leukaemia | B641.11 |
| 24458 | Type II diabetes mellitus - poor control | C109711 |
| 45913 | Type 2 diabetes mellitus - poor control | C109712 |
| 85744 | ADENOCARconfidence intervalNOMA ASCENDING COLON | 1530AD |
| 7654 | Secondary malignant neoplasm of bone and bone marrow | B585.00 |
| 46548 | Malignant neoplasm of pharyngeal tonsil | B071100 |
| 4554 | Malignant neoplasm of vulva unspecified | B454.00 |
| 20166 | Malignant neoplasm of female genital organ NOS | B45z.00 |
| 33914 | Operation on gastric ulcer NOS | 761Jz00 |
| 17858 | Type 1 diabetes mellitus | C108.12 |
| 24423 | Type I diabetes mellitus | C108.13 |
| 81833 | MICRONODULAR confidence intervalRRHOSIS | 5710MC |
| 97980 | CKD stage 1 with proteinuria | 1Z17.11 |
| 57442 | Malignant neoplasm of skin of lower limb and hip | B337.00 |
| 106969 |  |  |
| 55838 | [X]Predominantly cortical dementia | Eu01111 |
| 47556 | Malignant neoplasm of temporal lobe NOS | B512z00 |
| 50813 | Type II diabetes mellitus with mononeuropathy | C109A11 |
| 86812 | Malignant neoplasm of phalanges of hand | B305D00 |
| 62401 | Polyneuropathy in rheumatoid arthritis | F371200 |
| 19437 | Osteosarcoma | B30z000 |
| 59388 | Malignant neoplasm of mesocaecum | B18y100 |
| 65737 | Unspecified duodenal ulcer NOS | J12yz00 |
| 29325 | Intrinsic asthma without status asthmaticus | H331000 |
| 90659 | Malignant neoplasm of other specified endocrine gland | B54y.00 |
| 102201 | Type II diabetes mellitus with nephropathy | C10FC11 |
| 70824 | Malignant neoplasm of adrenal gland NOS | B540z00 |
| 59718 | Malig neop pituitary gland or craniopharyngeal duct NOS | B542z00 |
| 9476 | Teratoma of descended testis | B471100 |
| 90007 | MALIGNANT NEOPLASM NERVOUS SYSTEM | 1929A |
| 40823 | Brittle asthma | H334.00 |
| 90208 | ULCER GASTROJEJUNAL PERFORATED | 5340GJ |
| 16521 | Dissecting aortic aneurysm | G710.00 |
| 1867 | Abdominal aortic aneurysm without mention of rupture | G714.00 |
| 1641 | Oesophageal varices | G85..11 |
| 55431 | Pre-existing diabetes mellitus, unspecified | L180X00 |
| 71625 | Lymphosarcoma of unspecified site | B601000 |
| 51926 | Malignant neoplasm of faucial pillar | B062000 |
| 65599 | Malignant neoplasm of acoustic nerve | B520200 |
| 28148 | Malignant neoplasm of adrenal gland | B540.00 |
| 105740 |  |  |
| 44609 | Malignant neoplasm of ilium | B306000 |
| 83280 | MALIGNANT NEOPLASM NECK | 1959AG |
| 1800 | Malignant neoplasm of rectum | B141.00 |
| 64686 | Malignant neoplasm of areola of female breast | B340100 |
| 4743 | Alcoholic cirrhosis of liver | J612.00 |
| 4632 | Other malignant neoplasm of skin | B33..00 |
| 88465 | GASTROINTESTINAL MALIGNANCY | 159 |
| 4741 | Closure of perforated duodenal ulcer | 7627000 |
| 80615 | MYELOID LEUKAEMIA ACUTE | 2050 |
| 84360 | MYELOID LEUKAEMIA CHRONIC | 2051 |
| 73901 | [X]Cerebrovascular diseases | Gyu6.00 |
| 95343 | Type I diabetes mellitus with retinopathy | C10E711 |
| 75673 | ULCER PEPTIC | 5339 |
| 56709 | Malignant neoplasm of other sites of floor of mouth | B04y.00 |
| 35759 | Other specified nonarticular rheumatism | N2y..00 |
| 39187 | Plasma cell leukaemia | B631.00 |
| 94272 | Malig neoplasm of connective and soft tissues of lumb spine | B314100 |
| 68661 | Acute peptic ulcer without mention of complication | J130000 |
| 61555 | Malignant neoplasm of retroperitoneum NOS | B180z00 |
| 2195 | Bronchiectasis | H34..00 |
| 105889 |  |  |
| 45260 | [X]Malignant neoplasm of urinary organ, unspecified | Byu9000 |
| 70587 | Malignant neoplasm of skin of foot | B337700 |
| 88195 | ULCER STOMACH PERFORATED | 5310TM |
| 34944 | [X] Primary degenerative dementia NOS | Eu02z13 |
| 31701 | Chronic granulocytic leukaemia | B651.11 |
| 45089 | Chronic tracheobronchitis | H31y100 |
| 87642 | MALIGNANT NEOPLASM EPIGLOTTIS | 1618A |
| 108721 |  |  |
| 84081 | MEDULLOBLASTOMA BRAIN | 191 MB |
| 93478 | Malignant neoplasm, overlapping lesion of colon | B138.00 |
| 92804 | MALIGNANT NEOPLASM NIPPLE | 174 AN |
| 102547 |  |  |
| 103259 |  |  |
| 865 | Malignant melanoma of skin | B32..00 |
| 63920 | Ruptured suprarenal aortic aneurysm | G713000 |
| 71304 | Burkitt's lymphoma NOS | B602z00 |
| 83301 | MESOTHELIOMA PERITONEUM | 1589MP |
| 36731 | Malignant neoplasm of canthus | B331000 |
| 1062 | Malignant neoplasm of oesophagus | B10..00 |
| 106003 |  |  |
| 30543 | Malignant neoplasm of skin of breast | B335200 |
| 103532 |  |  |
| 37306 | [V]Personal history of malignant neoplasm of prostate | ZV10415 |
| 23936 | [V]Personal history of malignant neoplasm of cervix uteri | ZV10411 |
| 52303 | Non-insulin-dependent diabetes mellitus with renal comps | C109000 |
| 80980 | UTERUS BODY CARconfidence intervalNOMA | 1820C |
| 80892 | MALIGNANT NEOPLASM CHEEK EXTERNAL | 1733AC |
| 36495 | Carcinoma common bile duct | B161211 |
| 27391 | Secondary malignant neoplasm of peritoneum | B576100 |
| 93246 | MALIGNANT NEOPLASM SCALP | 1734AC |
| 20685 | Malignant neoplasm of axillary tail of female breast | B346.00 |
| 19412 | Subarachnoid haemorrhage from middle cerebral artery | G602.00 |
| 63198 | Rheumatoid arthritis of DIP joint of finger | N040A00 |
| 44440 | Insulin dependent diabetes mellitus with hypoglycaemic coma | C108E00 |
| 58061 | Malignant neoplasm of labia minora | B452.00 |
| 15103 | Secondary malignant neoplasm of liver | B577.00 |
| 90968 | MALIGNANT NEOPLASM GUM | 1439A |
| 96782 | Malignant neoplasm of lower lip, inner aspect NOS | B003z00 |
| 51551 | Secondary malignant neoplasm of mediastinum | B571.00 |
| 30576 | Malignant neoplasm of skin of forehead | B333300 |
| 57168 | Chron nephritic syndrom difuse membranous glomerulonephritis | K0A3200 |
| 68133 | Malignant melanoma of forehead | B323300 |
| 61662 | Hodgkin's disease NOS, unspecified site | B61z000 |
| 29979 | Non-insulin-dependent diabetes mellitus without complication | C109900 |
| 45467 | Non-insulin dependent diabetes mellitus with polyneuropathy | C109B00 |
| 83663 | TRANSIENT CEREBRAL ISCHAEMIA WITH HYPERT | 4350 |
| 83050 | CARconfidence intervalNOMA URINARY TRACT | 188 CT |
| 40997 | Chronic peptic ulcer | J131.00 |
| 35186 | [X]Malignant neoplasm of ill-defined, secondary and unspeci | ByuC.00 |
| 43151 | [X]Malignant neoplasm/bone??? cartilage, unspecified | Byu3300 |
| 94251 | Malignant neoplasm of lip, unspecified, lipstick area | B00z100 |
| 1469 | Stroke and cerebrovascular accident unspecified | G66..00 |
| 101530 | Hodgkin's disease, lymphocytic depletion NOS | B616z00 |
| 50696 | Malignant lymphoma NOS of lymph nodes of head, face and neck | B62y100 |
| 72774 | Subacute lymphoid leukaemia | B642.00 |
| 53636 | Human immunodeficiency virus with neurological disease | A788400 |
| 4251 | Acute lymphoid leukaemia | B640.00 |
| 98840 | Hodgkin's paragranuloma of intra-abdominal lymph nodes | B610300 |
| 79667 | MALIGNANT NEOPLASM CEREBRAL | 191 A |
| 3286 | Proliferative diabetic retinopathy | F420100 |
| 13071 | Diabetic - good control | 66AI.00 |
| 45154 | Malignant neoplasm of cerebellum | B516.00 |
| 70988 | Malignant neoplasm of skin of hip | B337000 |
| 103829 |  |  |
| 18632 | Malignant neoplasm of appendix | B135.00 |
| 17220 | Emergency repair of aortic aneurysm | 7A13.11 |
| 63695 | Malignant neoplasm of peripheral nerve of thorax | B524300 |
| 49262 | Follicular non-Hodgkin's large cell lymphoma | B627200 |
| 54613 | Malignant neoplasm of tympanic antrum | B201200 |
| 87277 | MALIGNANT NEOPLASM HEPATOCELLULAR | 1550HC |
| 94278 | Malignant neoplasm of gastro-oesophageal junction | B110111 |
| 102145 | Malignant melanoma of external auditory meatus | B322100 |
| 49554 | Type 1 diabetes mellitus with diabetic cataract | C10EF00 |
| 85456 | MYELOPROLIFERATING DISORDER | 2059MP |
| 76525 | MYELOPROLIFERATIVE DISEASE CHRONIC | 2059MR |
| 79362 | MYELOBLASTIC LEUKAEMIA | 2059MB |
| 86634 | MYELOCYTIC LEUKAEMIA | 2059MC |
| 12621 | [X]Dementia in other diseases classified elsewhere | Eu02.00 |
| 62475 | Malignant melanoma of hand | B326300 |
| 99240 | Reticulosarcoma NOS | B600z00 |
| 101668 | [X]Malignant neoplasm/peripheral nerves of trunk,unspecified | Byu5400 |
| 101668 | [X]Malignant neoplasm/peripheral nerves of trunk,unspecified | Byu5400 |
| 43435 | Malignant neoplasm of other site of cervix NOS | B41yz00 |
| 37919 | Secondary and unspec malig neop internal mammary lymph nodes | B561000 |
| 64336 | [X]Other specified types of non-Hodgkin's lymphoma | ByuD300 |
| 70869 | Human immunodeficiency virus with constitutional disease | A788300 |
| 70104 | Malignant neoplasm of cerebral meninges NOS | B521z00 |
| 65463 | High risk non proliferative diabetic retinopathy | F420800 |
| 43552 | Kahler's disease | B630.11 |
| 15211 | Myelomatosis | B630.12 |
| 92118 | MALIGNANT NEOPLASM MEDIASTINUM | 1631A |
| 99511 | Secondary malignant neoplasm of ileum | B574200 |
| 25602 | Malignant melanoma of finger | B326400 |
| 56345 | Secondary malignant neoplasm of other digestive organ | B57y.00 |
| 1786 | Subarachnoid haemorrhage | G60..00 |
| 67518 | [X]Other types of follicular non-Hodgkin's lymphoma | ByuD100 |
| 55303 | Hodgkin's nodular sclerosis of head, face and neck | B614100 |
| 1262 | Gastric ulcer - (GU) | J11..00 |
| 37160 | Congenital paraplegia | F230000 |
| 94776 | Malignant neoplasm, overlapping lesion of digestive system | B1z2.00 |
| 17559 | Malignant neoplasm of intestinal tract, part unspecified | B1z0.00 |
| 105275 |  |  |
| 24375 | Dermatofibrosarcoma protuberans | B339.00 |
| 39897 | Malignant neoplasm of pyriform sinus | B081.00 |
| 4388 | Malignant neoplasm of parotid gland | B020.00 |
| 90987 | SARCOMA STOMACH | 1519B |
| 75564 | STOMACH CARconfidence intervalNOMA | 1519C |
| 88250 | MALIGNANT NEOPLASM STOMACH | 1519A |
| 91429 | STOMACH LEIOMYBLASTOMA | 1519L |
| 16455 | Non-alcoholic cirrhosis NOS | J615z00 |
| 59288 | Other specified diabetes mellitus with coma | C103y00 |
| 15504 | Malignant lymphoma NOS of lymph nodes of multiple sites | B62y800 |
| 16202 | Malignant neoplasm of skin of nose (external) | B333400 |
| 35937 | Rheumatism or fibrositis NOS | N240z00 |
| 45766 | [X]Malignant neoplasm of intestinal tract, part unspecified | Byu1200 |
| 52594 | Malignant neoplasm of genitourinary organ NOS | B4z..00 |
| 50497 | Unspecified peptic ulcer | J13y.00 |
| 12735 | Gangrene of foot | G732100 |
| 107397 |  |  |
| 40159 | Purulent chronic bronchitis | H311000 |
| 33617 | Malignant neoplasm of body of uterus NOS | B43z.00 |
| 104895 |  |  |
| 82631 | CARconfidence intervalNOMA TESTIS | 186 C |
| 81919 | MONOARTICULAR RHEUMATISM | 715 MR |
| 77349 | SEMINOMA | 186 B |
| 82281 | CHORIONEPITHELIOMA | 181 C |
| 58545 | Traumatic subarachnoid haemorrhage | S627.00 |
| 45306 | Malignant melanoma of neck | B324100 |
| 98704 | Insulin dependent diabetes mellitus with ulcer | C10E512 |
| 75335 | CANCER | 1991MC |
| 106194 |  |  |
| 80443 | MESOTHELIOMA | 1991MT |
| 80908 | MALIGNANCY RECURRENT | 1991MR |
| 45824 | Secondary malignant neoplasm of tongue | B58y900 |
| 75716 | LEFT VENTRICULAR FAILURE ACUTE | 4271A |
| 8934 | [X]Subcortical vascular dementia | Eu01200 |
| 67446 | Malignant neoplasm of lower lip, vermilion border | B001.00 |
| 44931 | Secondary and unspec malig neop intra-abdominal LN NOS | B562z00 |
| 67129 | Secondary unspec malig neop lymph nodes head/face/neck NOS | B560z00 |
| 68824 | Malignant neoplasm, overlapping lesion male genital orgs | B48y200 |
| 8842 | Diabetic on insulin | 66A5.00 |
| 100296 | Secondary malignant neoplasm of skin of face | B582100 |
| 52537 | Malignant neoplasm of hepatic duct | B161100 |
| 90724 | ADAMANTINOMA | 2104AD |
| 107681 |  |  |
| 36240 | Bird-fancier's lung NOS | H352z00 |
| 68612 | [V]Personal history of unspecified malignant neoplasm | ZV10z00 |
| 12833 | Right sided CVA | G668.00 |
| 61494 | Chronic membranoproliferative glomerulonephritis | K022.00 |
| 75840 | THROMBOSIS CORONARY | 4109TC |
| 16760 | Secondary malignant neoplasm of breast | B58y000 |
| 8693 | Carcinoma of other and unspecified sites | B5...11 |
| 56803 | NIDDM with peripheral circulatory disorder | C107400 |
| 28276 | Acute myelofibrosis | B675.00 |
| 26442 | Cannabinosis | H441.00 |
| 70448 | Diabetes mellitus, juvenile ??? circulatory disorder | C107000 |
| 41691 | Secondary and unspec malig neop coeliac lymph nodes | B562000 |
| 85253 | RHEUMATISM HANDS ACUTE | 718 BH |
| 40837 | Type 1 diabetes mellitus with ketoacidotic coma | C10EN00 |
| 24370 | Malignant neoplasm of anal canal | B142.00 |
| 62104 | Malignant neoplasm of temporal bone | B300800 |
| 64165 | Acute gastric ulcer without mention of complication | J110000 |
| 109215 |  |  |
| 84082 | LYMPHOSARCOMA | 2001 |
| 81539 | SARCOMA RETICULUM-CELL | 2000 |
| 93206 | Suberosis ( cork-handlers' lung ) | H353.00 |
| 62200 | Bagassosis | H351.00 |
| 60107 | Unstable type I diabetes mellitus | C108411 |
| 63371 | Diabetes mellitus, adult, other specified manifestation | C10y100 |
| 15976 | Malignant neoplasm of abdomen | B552.00 |
| 63331 | Malignant neoplasm of spermatic cord | B485.00 |
| 78239 | CHRONIC ASTHMA | 493 JC |
| 99414 | Rheumatoid arthritis of lesser MTP joint | N040L00 |
| 57675 | Libman-Sacks disease | N000100 |
| 107958 |  |  |
| 90425 | MENINGEAL HAEMORRHAGE | 4309M |
| 45499 | Kimmelstiel - Wilson disease | K01x111 |
| 19141 | Malignant neoplasm of ovary and other uterine adnexa | B44..00 |
| 34803 | Other acute myocardial infarction | G30y.00 |
| 38510 | Malignant neoplasm of testis NOS | B47z.00 |
| 81364 | ASTHMA OCCASIONAL | 493 AD |
| 59823 | Malignant neoplasm pituitary gland and craniopharyngeal duct | B542.00 |
| 67193 | Nephritis unsp membranoprolif glomerulonephritis lesion | K032y00 |
| 32362 | Malignant neoplasm of fundus of stomach | B113.00 |
| 21620 | Malignant neoplasm of pylorus of stomach | B111.00 |
| 51690 | Malignant neoplasm, overlapping lesion of stomach | B117.00 |
| 91525 | ASTHMA HIGH RISK | 493 HR |
| 101885 | Mesothelioma of pericardium | B241400 |
| 657 | Unspecified duodenal ulcer with perforation | J12y200 |
| 9470 | Malignant neoplasm of female breast NOS | B34z.00 |
| 106090 |  |  |
| 34152 | Diabetic peripheral angiopathy | G73y000 |
| 11312 | Extrinsic allergic alveolitis | H35..00 |
| 9902 | Carcinoma of bone, connective tissue, skin and breast | B3...11 |
| 12539 | Sarcoma of bone and connective tissue | B3...12 |
| 36693 | [V]Personal history of leukaemia | ZV10600 |
| 1350 | Senile/presenile dementia | E00..12 |
| 73619 | Rheumatoid arthritis of subtalar joint | N040G00 |
| 25886 | Malignant neoplasm of upper lobe of lung | B222100 |
| 79397 | CHRONIC BRONCHITIS WITH EMPHYSEMA | 491 E |
| 45521 | Juxtarenal aortic aneurysm | G714000 |
| 48517 | Malignant neoplasm of soft tissue of neck | B310200 |
| 106654 |  |  |
| 22163 | Carcinoma of caecum | B134.11 |
| 88070 | ULCER PEPTIC DUODENUM PERFORATED | 5320PT |
| 86250 | MALIGNANT NEOPLASM LYMPH GLAND/NODE | 1969A |
| 89375 | HAMARTOMA FOETAL RENAL | 1890HM |
| 57315 | Intracerebral haemorrhage, multiple localized | G618.00 |
| 96630 | [X]Intracerebral haemorrhage in hemisphere, unspecified | Gyu6F00 |
| 70374 | Reticulosarcoma of intra-abdominal lymph nodes | B600300 |
| 91342 | MALIGNANT NEOPLASM BOWEL | 1539A |
| 8386 | Malignant neoplasm of stomach | B11..00 |
| 15221 | Malignant neoplasm of trachea | B220.00 |
| 99040 | Paraplegia - congenital | F230.11 |
| 24374 | Carcinoma of lip, oral cavity and pharynx | B0...11 |
| 87154 | MEGAKARYOCYTIC LEUKAEMIA ACUTE | 2070MG |
| 42719 | Systemic lupus erythematosus NOS | N000z00 |
| 34117 | Other cerebrovascular disease OS | G67y.00 |
| 108724 |  |  |
| 36401 | Secondary malignant neoplasm of adrenal gland | B587.00 |
| 97894 | Type I diabetes mellitus with exudative maculopathy | C10EP11 |
| 65372 | Malignant neoplasm of other specified site of stomach NOS | B11yz00 |
| 59170 | Malignant neoplasm of corpus callosum | B51y000 |
| 16874 | Carcinoma of genitourinary organ | B4...11 |
| 48231 | Malignant neoplasm of other specified sites of colon | B13y.00 |
| 49054 | Malignant neoplasm of scapula | B304000 |
| 64721 | Chronic emphysema due to chemical fumes | H464000 |
| 84416 | EMBRYONAL CARconfidence intervalNOMA TESTIS | 186 EM |
| 69927 | Malignant neoplasm of first metatarsal bone | B308800 |
| 96456 | Endemic polyarthritis | N060.11 |
| 4222 | Lymphatic leukaemia | B64..11 |
| 100006 | Burkitt's lymphoma of intrathoracic lymph nodes | B602200 |
| 21330 | Malignant neoplasm of retroperitoneum | B180.00 |
| 99896 | Malignant neoplasm of other specified site small intestine | B12y.00 |
| 88140 | MALIGNANT NEOPLASM MAXILLA | 1700AM |
| 63615 | Other chronic glomerulonephritis NOS | K02yz00 |
| 19028 | Solitary myeloma | B630100 |
| 82663 | CARconfidence intervalNOMA URETER | 1892C |
| 84667 | SCROTAL GANGRENE | 6075GM |
| 8166 | Malignant neoplasm of pancreas | B17..00 |
| 11920 | Systemic lupus erythematosus with pericarditis | N000400 |
| 36942 | Drug-induced systemic lupus erythematosus | N000200 |
| 55019 | Malignant neoplasm of other specified site of stomach | B11y.00 |
| 2378 | Diabetic - poor control | 66AJ.00 |
| 99185 | Malignant neoplasm of glossopalatine fold | B062100 |
| 42569 | Malignant neoplasm of respiratory tract NOS | B2zz.00 |
| 23399 | Malignant neoplasm of upper-outer quadrant of female breast | B344.00 |
| 100733 | Malignant neoplasm of other part of brain NOS | B51yz00 |
| 29826 | Malignant neoplasm of upper-inner quadrant of female breast | B342.00 |
| 75246 | SPASTIC PARAPLEGIA | 343 PR |
| 26853 | Malignant neoplasm of nipple and areola of female breast | B340.00 |
| 13577 | Other cerebrovascular disease | G67..00 |
| 107884 |  |  |
| 9013 | Unstable diabetes | 66AJ.11 |
| 19974 | Acute monocytic leukaemia | B660.00 |
| 14792 | Malignant neoplasm of other and unspecified parts of mouth | B05..00 |
| 67324 | Malig neop of connective and soft tissue of inguinal region | B315100 |
| 88144 | Malignant neoplasm of other specified part of nervous system | B52y.00 |
| 3665 | Late onset asthma | H331.11 |
| 3450 | Diffuse nodular cirrhosis | J615300 |
| 93298 | MALIGNANT NEOPLASM EAR EXTERNAL | 1959AE |
| 91376 | MALIGNANT NEOPLASM HEAD | 1959AA |
| 92245 | Hodgkin's, lymphocytic-histiocytic pred intrathoracic nodes | B613200 |
| 104790 |  |  |
| 53822 | Acute duodenal ulcer unspecified | J120y00 |
| 43619 | Malignant neoplasm of skin of neck | B334100 |
| 84533 | RHEUMATOID ARTHRITIS SPINE | 7124A |
| 77463 | HEMIPLEGIA RIGHT | 344 BR |
| 94935 | Lymphoma stage II | 4M21.00 |
| 30402 | Malignant neoplasm of buccal mucosa | B050.11 |
| 95378 | Secondary and unspec malig neop diaphragmatic lymph nodes | B561200 |
| 14800 | Malignant neoplasm of stomach NOS | B11z.00 |
| 37842 | Malignant neoplasm of rib | B303000 |
| 37810 | Malignant neoplasm of trachea NOS | B220z00 |
| 18619 | Malignant neoplasm of splenic flexure of colon | B137.00 |
| 51965 | Malignant neoplasm of connective and soft tissue of pelvis | B315.00 |
| 6935 | Malignant neoplasm of transverse colon | B131.00 |
| 2815 | Malignant neoplasm of sigmoid colon | B133.00 |
| 37365 | Byssinosis | H440.00 |
| 69663 | Chronic peptic ulcer NOS | J131z00 |
| 32024 | Malignant neoplasm of upper gum | B030.00 |
| 81279 | MASTOCYTOSIS SYSTEMIC | 2070MC |
| 88111 | MALIGNANT GANGLIONEUROMA | 1925GN |
| 82769 | MASTOCYTOMA | 2070MA |
| 5702 | Peripheral ischaemic vascular disease | G73..11 |
| 25245 | Malignant neoplasm of skin of finger | B336400 |
| 88022 | [X]Secondary malignant neoplasm/oth?? digestive organs | ByuC400 |
| 84955 | SUBARACHNOID HAEMORRHAGE WITH HYPERTENSI | 4300 |
| 74318 | SUBARACHNOID HAEMORRHAGE | 4309 |
| 98911 | Malignant neoplasm of nasal conchae | B200100 |
| 89065 | MALIGNANT FIBROBLASTOMA | 1719FB |
| 51166 | Y graft abdominal Aortic aneurysm | 7A11311 |
| 83827 | ADENOCARconfidence intervalNOMA KIDNEY | 1890AD |
| 59286 | Malignant neoplasm of overlapping lesion of urinary organs | B4Ay000 |
| 45760 | Malignant melanoma of trunk, excluding scrotum, NOS | B325z00 |
| 91037 | Malignant neoplasm of other specified site of oropharynx NOS | B06yz00 |
| 47094 | Malignant melanoma of eyebrow | B323200 |
| 72224 | Fibrosarcoma of spleen | B1z1100 |
| 86997 | [X]Malignant neoplasm/ill-defined sites within resp system | Byu2400 |
| 96280 | Clark melanoma level 3 | 4M72.00 |
| 8154 | Malignant ascites | B576200 |
| 55098 | Malignant neoplasm of head NOS | B550000 |
| 14803 | Diabetes mellitus, adult onset, no mention of complication | C100100 |
| 79648 | CHOLANGIO CARconfidence intervalNOMA | 1551C |
| 59041 | Malignant neoplasm of ciliary body | B500000 |
| 90076 | MALIGNANT LYMPHOMA STOMACH | 1519BL |
| 79292 | SECONDARY NEOPLASTIC DEPOSITS BONE | 1985 |
| 80921 | SECONDARY NEOPLASTIC DEPOSITS BRAIN | 1983 |
| 84139 | SKIN SECONDARY DEPOSITS | 1982 |
| 12019 | Seropositive rheumatoid arthritis, unspecified | N04X.00 |
| 80826 | SECONDARY NEOPLASTIC DEPOSITS | 1989 |
| 57248 | Malignant neoplasm aryepiglottic fold, hypopharyngeal aspect | B082.00 |
| 80917 | HEMIPLEGIA LEFT | 344 BL |
| 91960 | HEMIPLEGIA FLACconfidence intervalD | 344 BF |
| 31573 | Malignant neoplasm of pleura | B23..00 |
| 43921 | Unstable type 1 diabetes mellitus | C10E400 |
| 18496 | Type 2 diabetes mellitus with retinopathy | C10F600 |
| 71262 | Malignant lymphoma NOS of intrapelvic lymph nodes | B62y600 |
| 33474 | Rheumatism and fibrositis unspecified | N240.00 |
| 73536 | Malignant melanoma of hip | B327000 |
| 36583 | Bleeding chronic gastric ulcer | J111111 |
| 107216 |  |  |
| 92720 | Malignant neoplasm of posterior mediastinum | B243.00 |
| 98596 | [X]Other types of diffuse non-Hodgkin's lymphoma | ByuD200 |
| 106483 |  |  |
| 65793 | Malig neop of upper respiratory tract, part unspecified | B2z0.00 |
| 44617 | HIV disease resulting in Burkitt's lymphoma | A789600 |
| 97672 | Secondary malig neop of retroperitoneum or peritoneum NOS | B576z00 |
| 89078 | NEPHRITIS HEREDITARY | 7598A |
| 21482 | Diabetes mellitus with hyperosmolar coma | C102.00 |
| 53126 | Chronic peptic ulcer with haemorrhage | J131100 |
| 4693 | [X] Unspecified dementia | Eu02z00 |
| 108037 |  |  |
| 97758 | Chronic glomerulonephritis diseases EC | K02y000 |
| 38961 | Malignant neoplasm of other sites of bronchus or lung | B22y.00 |
| 90683 | MALIGNANT NEOPLASM ADENOID | 147 A |
| 74331 | RENAL FAILURE | 5932E |
| 91345 | MALIGNANT NEOPLASM MANDIBLE | 1701A |
| 32955 | Malignant neoplasm of other site of cervix | B41y.00 |
| 31324 | Mast cell malignancy of lymph nodes of multiple sites | B626800 |
| 28417 | Systemic sclerosis | N001.12 |
| 68277 | Acrosclerosis | N001.11 |
| 39420 | Myasthenic syndrome due to diabetic amyotrophy | F381300 |
| 80569 | HEPATITIS CHRONIC | 5719CH |
| 76546 | confidence intervalRRHOSIS | 5719CL |
| 32627 | Type 2 diabetes mellitus with ketoacidosis | C10FN00 |
| 60403 | Malignant neoplasm of costal cartilage | B303300 |
| 50372 | H/O: Myocardial infarction in last year | 14AH.00 |
| 41490 | Malignant melanoma of foot | B327700 |
| 77161 | ULCER DIABETIC | 250 G |
| 77949 | HYPOGLYCAEMIA IN DIABETES MELLITUS | 250 E |
| 87626 | CHARCOT'S DIABETIC ARTHROPATHY | 250 M |
| 98104 | Malignant neoplasm of other specified pleura | B23y.00 |
| 108964 |  |  |
| 79950 | GANGRENE | 4459N |
| 105966 |  |  |
| 74417 | RHEUMATOID ARTHRITIS | 7123 |
| 81274 | SYNDROME FELTY'S | 7121 |
| 24040 | Acute gastric ulcer | J110.00 |
| 50728 | Renal infantilism | K080200 |
| 56838 | Caplan's syndrome | N04y011 |
| 28853 | Fibrosing alveolitis associated with rheumatoid arthritis | N04y012 |
| 97530 | Malignant neoplasm of lower buccal sulcus | B051100 |
| 104608 |  |  |
| 53797 | Acute duodenal ulcer NOS | J120z00 |
| 51261 | Insulin dependent diabetes mellitus | C10E.12 |
| 25535 | Primary malignant neoplasm of liver | B150.00 |
| 84275 | ECZEMA WITH ASTHMA | 691 TM |
| 64111 | Unspecified peptic ulcer with perforation | J13y200 |
| 88051 | PRURITUS DIABETIC | 250 PR |
| 63460 | Malignant neoplasm of arytenoid cartilage | B213000 |
| 35039 | Malignant neoplasm, overlapping lesion of biliary tract | B163.00 |
| 23433 | Malignant neoplasm of extrahepatic bile ducts | B161.00 |
| 40787 | Thoracoabdominal aortic aneurysm, without mention of rupture | G716000 |
| 40788 | Other emphysema | H32y.00 |
| 148 | Bronchitis unspecified | H30..00 |
| 47214 | Rigid oesophagoscopic injection sclerotherapy oesoph varices | 760F300 |
| 19260 | Posterior cerebral artery syndrome | G662.00 |
| 27884 | Decompensated cardiac failure | G580200 |
| 62124 | Secondary and unspec malig neop bronchopulmonary lymph nodes | B561800 |
| 38986 | Diabetes mellitus with no mention of complication | C100.00 |
| 43642 | Malignant neoplasm of dorsal surface of tongue | B011.00 |
| 5199 | Cerebral metastasis | B583200 |
| 3480 | Bronchitis NOS | H30z.00 |
| 88831 | ODONTOMA | 2104DN |
| 58958 | Malignant melanoma of temple | B323500 |
| 105166 |  |  |
| 49725 | Other lymphoid leukaemia | B64y.00 |
| 102611 |  |  |
| 27449 | Malignant neoplasm of upper limb NOS | B554.00 |
| 50297 | Malignant neoplasm of exocervix | B411.00 |
| 35107 | Diabetes mellitis with nephropathy NOS | C104z00 |
| 72345 | Diabetes mellitus NOS with hyperosmolar coma | C102z00 |
| 45264 | Nodular lymphoma of lymph nodes of head, face and neck | B620100 |
| 102117 | [X]HIV disease resulting in multiple infections | AyuC300 |
| 57756 | [X]Malignant neoplasm/other specified female genital organs | Byu7100 |
| 86789 | LARGE BOWEL CARconfidence intervalNOMA NONRECTAL | 1538CN |
| 63017 | Type I diabetes mellitus maturity onset | C108911 |
| 97446 | Type 1 diabetes mellitus maturity onset | C108912 |
| 17322 | Cerebellar stroke syndrome | G664.00 |
| 69313 | Subacute yellow atrophy | J601200 |
| 7780 | Left sided CVA | G667.00 |
| 27715 | Malignant neoplasm of anterior mediastinum | B242.00 |
| 82930 | CEREBRAL NEOPLASM | 191 BC |
| 25582 | Acute renal failure NOS | K04z.00 |
| 81575 | TERATOMA OVARY | 1830TT |
| 5255 | Acute left ventricular failure | G581000 |
| 66062 | Renal rickets | K080300 |
| 5884 | NIDDM - Non-insulin dependent diabetes mellitus | C109.11 |
| 17859 | Type 2 diabetes mellitus | C109.12 |
| 42193 | Malignant neoplasm of lesser curve of stomach unspecified | B115.00 |
| 1952 | Secondary malignant neoplasm of kidney | B580.00 |
| 19945 | Secondary malignant neoplasm of skin | B582.00 |
| 50475 | Malignant neoplasm of major salivary gland NOS | B02z.00 |
| 23511 | Hepatic coma | J622.00 |
| 80026 | CARconfidence intervalNOMA ANAL CANAL | 1542C |
| 47315 | Type II diabetes mellitus - poor control | C10F711 |
| 25191 | Leukaemia of unspecified cell type | B68..00 |
| 58082 | Nodular lymphoma of lymph nodes of multiple sites | B620800 |
| 55101 | Malignant neoplasm of pelvis NOS | B553z00 |
| 105636 | Angioimmunoblastic T-cell lymphoma |  |
| 90684 | MALIGNANT NEOPLASM FACE | 1733AF |
| 46728 | Malignant neoplasm of anterior epiglottis | B064.00 |
| 82772 | SECONDARY BILIARY confidence intervalRRHOSIS (LIVER) | 5719CB |
| 64014 | Closure of gastric ulcer NEC | 761J100 |
| 100964 | Type II diabetes mellitus with ophthalmic complications | C10F111 |
| 93004 | SARCOMA SPERMATIC CORD | 1878 |
| 47782 | Chronic pulmonary fibrosis due to chemical fumes | H464200 |
| 53336 | Chronic gastric ulcer with perforation | J111200 |
| 65376 | Pneumoconiosis due to other inorganic dust | H43..00 |
| 77187 | CARconfidence intervalNOMA UTERUS | 1829C |
| 80774 | MALIGNANT NEOPLASM UTERUS | 1829A |
| 94793 | Chronic kidney disease stage 3 with proteinuria | 1Z1B.00 |
| 43463 | Malignant melanoma of back | B325700 |
| 57482 | Malignant neoplasm of connective and soft tissue of fore-arm | B311200 |
| 16956 | Cerebral palsy, not congenital or infantile, acute | G669.00 |
| 98071 | Insulin-dependent diabetes mellitus with ophthalmic comps | C10E112 |
| 99311 | Type I diabetes mellitus with ophthalmic complications | C10E111 |
| 33343 | Diabetes mellitus with other specified manifestation | C10y.00 |
| 60035 | Malignant neoplasm of cartilage of ear | B310300 |
| 88549 | MALIGNANT NEOPLASM PENIS | 1870A |
| 74638 | NEUROPATHY DIABETIC | 250 F |
| 74528 | SUGAR DIABETES | 250 A |
| 77996 | COMA DIABETIC | 250 H |
| 79582 | SALIVARY ADENOMA | 2102A |
| 1408 | Polymyalgia rheumatica | N20..00 |
| 93577 | Stannosis | H435.00 |
| 6916 | Seronegative rheumatoid arthritis | N040P00 |
| 89916 | Malignant neoplasm of presacral region | B553100 |
| 7871 | Systemic lupus erythematosus | N000.00 |
| 105739 | Acute renal failure |  |
| 45782 | Extrinsic asthma NOS | H330z00 |
| 99887 | Other specified reticulosarcoma or lymphosarcoma | B60y.00 |
| 69821 | Malignant neoplasm of the pouch of Douglas | B18y600 |
| 31393 | Carcinoma gallbladder | B160.11 |
| 33395 | Secondary and unspec malig neop superficial cervical LN | B560200 |
| 60242 | Reticulosarcoma of unspecified site | B600000 |
| 106860 | Renal failure-associated hyperphosphataemia |  |
| 95220 | CARconfidence intervalNOMA ISLETS OF LANGERHANS | 1579CL |
| 47328 | O/E - right eye stable treated prolif diabetic retinopathy | 2BBk.00 |
| 5928 | Operations on duodenal ulcer | 7627 |
| 15511 | Polymyositis | N004.00 |
| 87285 | MYOSARCOMA | 1719M |
| 81582 | HAEMANGIOBLASTOMA | 1719H |
| 79761 | FIBROSARCOMA | 1719F |
| 2587 | Lung cancer | B22z.11 |
| 61064 | Malignant neoplasm of mediastinum, part unspecified | B24X.00 |
| 42299 | Rheumatoid arthritis of MCP joint | N040800 |
| 103245 | Lymphosarcoma |  |
| 79174 | ASTROCYTOMA | 1929AC |
| 41676 | Renal cortical necrosis unspecified | K034.00 |
| 22205 | Lupus nephritis | K01x411 |
| 102400 | Asthma causes night time symptoms 1 to 2 times per week | 66Yq.00 |
| 70928 | Malignant neoplasm of sublingual gland | B022.00 |
| 12465 | Membranoproliferative nephritis unspecified | K032.00 |
| 88326 | CARconfidence intervalNOMA AMPULLA VATER | 1562C |
| 23380 | Malignant neoplasm of nipple of female breast | B340000 |
| 5871 | H/O: stroke | 14A7.12 |
| 73556 | Malignant neoplasm of hand bones NOS | B305z00 |
| 1481 | Reticulosarcoma | B600.00 |
| 18625 | Bleeding chronic duodenal ulcer | J121111 |
| 45184 | Acute gastrojejunal ulcer | J140.00 |
| 14712 | Malignant neoplasm of lip | B00..00 |
| 35113 | [X]Malignant neoplasm of urinary tract | Byu9.00 |
| 43785 | Non-insulin dependent diabetes mellitus with hypoglyca coma | C109D00 |
| 15403 | Gastrojejunal ulcer NOS | J14z.00 |
| 40814 | Malignant neoplasm of tibia | B307200 |
| 17182 | Follicular lymphoma NOS | B627C11 |
| 29353 | Hand rheumatism | N240700 |
| 62209 | Type I diabetes mellitus with ketoacidosis | C10EM11 |
| 55953 | Malignant neoplasm of occipital bone | B300400 |
| 53877 | Chronic hepatitis unspecified | J614y00 |
| 17767 | Abdominal aortic aneurysm which has ruptured | G713.00 |
| 44141 | Progressive systemic sclerosis | N001000 |
| 84569 | PAGET'S DISEASE NIPPLE | 174 PN |
| 60796 | Type II diabetes mellitus with persistent proteinuria | C10FL11 |
| 88267 | MALIGNANT NEOPLASM ABDOMEN | 1950A |
| 44637 | Acute peptic ulcer with haemorrhage | J130100 |
| 104025 | Malignant neoplasm of skin of heel |  |
| 31060 | Intracerebral haemorrhage in hemisphere, unspecified | G61X.00 |
| 1895 | Transient cerebral ischaemia NOS | G65z.00 |
| 9507 | Acute non-Q wave infarction | G307000 |
| 28451 | Malignant neoplasm of hypopharynx NOS | B08z.00 |
| 54893 | Compensatory emphysema | H582.00 |
| 782 | Neoplasms | B....00 |
| 20440 | Myelomonocytic leukaemia | B69..00 |
| 58973 | [X]Malignant neoplasm of lip, oral cavity and pharynx | Byu0.00 |
| 87459 | RENAL MEDULLARY NECROSIS | 5932MN |
| 46912 | H/O: Heart failure in last year | 14AM.00 |
| 13101 | O/E - left eye proliferative diabetic retinopathy | 2BBV.00 |
| 101988 | Malignant neoplasm of palatine tonsil | B060100 |
| 90078 | LYMPHOSARCOMA SMALL INTESTINE | 1529BL |
| 55066 | Malignant neoplasm of tonsillar pillar | B062.00 |
| 51634 | [D]Gangrene, spreading cutaneous | R054000 |
| 47633 | [X]Malig neopl, overlap lesion brain & other part of CNS | ByuA300 |
| 13559 | Malig neop of kidney and other unspecified urinary organs | B4A..00 |
| 88242 | MALIGNANT NEOPLASM MOUTH | 1459A |
| 82503 | MOUTH CARconfidence intervalNOMA | 1459C |
| 103066 | Pigmented basal cell carcinoma |  |
| 103440 | Basal cell carcinoma |  |
| 41520 | Malignant neoplasm of brain NOS | B51z.00 |
| 39870 | Malignant neoplasm of tail of pancreas | B172.00 |
| 35795 | Malignant neoplasm of Islets of Langerhans | B174.00 |
| 37969 | Malignant neoplasm of skin of chest, excluding breast | B335100 |
| 8403 | Non-insulin dependant diabetes mellitus - poor control | C109700 |
| 102395 | Asthma causes symptoms most nights | 66Yr.00 |
| 66908 | Malignant neoplasm of coccygeal vertebra | B306400 |
| 11991 | Primary vulval cancer | B454.11 |
| 1736 | Aortic aneurysm repair | 7A14.11 |
| 35999 | Secondary malignant neoplasm of skin of neck | B582200 |
| 103900 | Mast cell malignancy |  |
| 10980 | Centrilobular emphysema | H322.00 |
| 47632 | HIV disease result/haematological???? abnorms,NEC | A788U00 |
| 1647 | Insulin dependent diabetes mellitus | C108.00 |
| 43490 | [X]Other specified carcinomas of liver | Byu1100 |
| 50048 | Acute peptic ulcer NOS | J130z00 |
| 55670 | Malignant neoplasm of skin of eyebrow | B333200 |
| 106897 | Heart failure |  |
| 49491 | Malignant neoplasm of sternum | B303100 |
| 76578 | INTRA-ABDOMINAL CANCER | 1950 |
| 56007 | Subarachnoid haemorrhage from carotid siphon and bifurcation | G601.00 |
| 94279 | Hodgkin's disease NOS of spleen | B61z700 |
| 72320 | Non-insulin dependent diabetes mellitus with mononeuropathy | C109A00 |
| 109197 | Type II diabetes |  |
| 95636 | Latent autoimmune diabetes mellitus in adult | C10ER00 |
| 57737 | Lymphoepithelioid lymphoma | B62x100 |
| 108715 | Histiocytic leukaemia |  |
| 63300 | [X]Malignant neoplasm/overlap lesion/bone??? cartilage | Byu3200 |
| 38005 | Mycosis fungoides NOS | B621z00 |
| 54856 | Diabetes mellitus, adult onset, with ketoacidosis | C101100 |
| 43431 | Malignant neoplasm of base of tongue | B010.00 |
| 78618 | OESOPHAGUS CARconfidence intervalNOMA | 150 C |
| 2986 | Preproliferative diabetic retinopathy | F420200 |
| 101095 | [M]Grade 1 (Stage pTa) papillary urothelial/transit cell ca | BB4B.00 |
| 89258 | Malignant neoplasm of peripheral nerve of low limb, incl hip | B524200 |
| 59061 | Malignant melanoma of auricle (ear) | B322000 |
| 17345 | AAA - Abdominal aortic aneurysm without mention of rupture | G714.11 |
| 39070 | Type 1 diabetes mellitus with hypoglycaemic coma | C10EE00 |
| 45755 | Malignant melanoma of fore-arm | B326200 |
| 97978 | CKD stage 2 without proteinuria | 1Z1A.11 |
| 17391 | Malignant neoplasm of carina of bronchus | B221000 |
| 64897 | [X]Malignant neoplasms/independent(primary)multiple sites | ByuE000 |
| 93342 | Monocytic leukaemia NOS | B66z.00 |
| 93665 | [X]Kaposi's sarcoma, unspecified | Byu5300 |
| 106197 | Basophilic leukaemia |  |
| 48102 | Other sequelae of chronic liver disease | J62y.00 |
| 36200 | Secondary malig neop of large intestine or rectum NOS | B575z00 |
| 63690 | Type 2 diabetes mellitus with gastroparesis | C10FR00 |
| 91928 | FRANKLIN'S DISEASE | 203 FR |
| 42567 | Diabetes mellitus, juvenile type, with ketoacidotic coma | C103000 |
| 27370 | Malignant neoplasm skin of other and unspecified parts face | B333.00 |
| 780 | Malignant neoplasm of prostate | B46..00 |
| 11626 | Diabetic retinopathy NOS | F420z00 |
| 4850 | Nephritis and nephropathy unspecified | K03..11 |
| 24248 | Mixed simple and mucopurulent chronic bronchitis | H313.00 |
| 49214 | Secondary and unspec malig neop lymph nodes head/face/neck | B560.00 |
| 11150 | Mucopurulent chronic bronchitis | H311.00 |
| 78853 | LYMPHATIC LEUKAEMIA CHRONIC | 2041 |
| 74652 | LYMPHATIC LEUKAEMIA ACUTE | 2040 |
| 28314 | Left sided intracerebral haemorrhage, unspecified | G61X000 |
| 51352 | Malignant neoplasms of independent (primary) multiple sites | B592.00 |
| 47669 | [V]Personal history of malignant neoplasm of skin | ZV10y14 |
| 46282 | [V]Personal history of malignant neoplasm of bone | ZV10y11 |
| 48085 | [V]Personal history of malignant neoplasm of brain | ZV10y12 |
| 97474 | Unstable type 1 diabetes mellitus | C108412 |
| 6712 | End stage renal failure | K050.00 |
| 99257 | Malignant melanoma of scalp and neck NOS | B324z00 |
| 74496 | ULCER DUODENUM | 5329 |
| 55292 | Malignant melanoma of upper limb or shoulder NOS | B326z00 |
| 105383 | Chronic kidney disease |  |
| 105073 | Synocial sarcoma, spindle cell type |  |
| 108816 | Nephrotic syndrome in diseases |  |
| 57225 | Hodgkin's disease, nodular sclerosis of unspecified site | B614000 |
| 108922 | Wilms tumour |  |
| 18027 | Acute duodenal ulcer | J120.00 |
| 42331 | Subarachnoid haemorrhage from anterior communicating artery | G603.00 |
| 104862 | Cutaneou T-cell lyphoma |  |
| 79104 | SARCOMA | 1719B |
| 101608 | Malignant neoplasm of ureteropelvic junction | B4A1100 |
| 83845 | ACQUIRED IMMUNE DEFIconfidence intervalENCY SYNDROME | L7990A |
| 36461 | Unspecified gastric ulcer with perforation | J11y200 |
| 45284 | SYSTEMIC LUPUS ERYTHEMATOSUS WITH RENAL | 2A42.00 |
| 65489 | Hodgkin's paragranuloma | B610.00 |
| 16105 | Malignant neoplasm of gallbladder | B160.00 |
| 10726 | Chronic myeloid leukaemia | B651.00 |
| 70724 | Myeloid sarcoma | B653.00 |
| 102594 | Diffuse large B-cell lymphoma |  |
| 59362 | Malignant neoplasm of labia majora NOS | B451z00 |
| 46460 | Silica and silicate pneumoconiosis | H42..00 |
| 62582 | Oesophageal varices in diseases EC NOS | G852z00 |
| 44303 | Human immunodef virus resulting in other disease | A789.00 |
| 74896 | Malignant neoplasm of extrahepatic bile ducts NOS | B161z00 |
| 75635 | CERVIX UTERI CARconfidence intervalNOMA | 180 C |
| 79414 | MALIGNANT NEOPLASM UTERUS CERVIX | 180 A |
| 93762 | Malignant neoplasm of placenta | B42..00 |
| 54956 | Malignant neoplasm of eye NOS | B50z.00 |
| 74310 | MYELOMA | 203 N |
| 33871 | Malignant neoplasm of ileum | B122.00 |
| 92028 | SOLITARY MYELOMA | 203 A |
| 82654 | MYELOMATOSIS | 203 T |
| 88454 | LEUKAEMIA PROMYELOCYTIC ACUTE | 2050PM |
| 20160 | Malignant neoplasm of eye | B50..00 |
| 105085 | T/NK-cell lymphoma |  |
| 5179 | Nodular lymphoma (Brill - Symmers disease) | B620.00 |
| 75952 | CARconfidence intervalNOMA NOSE EXTERNAL | 1723C |
| 80159 | MYELOID LEUKAEMIA | 2059M |
| 17545 | Type I diabetes mellitus with diabetic cataract | C108F11 |
| 64516 | Malignant neoplasm of parietal peritoneum | B18y400 |
| 18209 | Type 2 diabetes mellitus with renal complications | C109012 |
| 43930 | Secondary malignant neoplasm of skin of head | B582000 |
| 44073 | Chronic duodenal ulcer with obstruction | J121400 |
| 47899 | Malignant neoplasm of greater vestibular (Bartholin's) gland | B451000 |
| 96756 | Oesophageal varices with bleeding in diseases EC | G852000 |
| 7982 | Malignant neoplasm of common bile duct | B161200 |
| 30706 | [X]Dementia in Alzheimer's dis, atypical or mixed type | Eu00200 |
| 3018 | Mild asthma | 663V100 |
| 102142 | Malignant neoplasm of anterior 2/3 of tongue ventral surface | B013000 |
| 55090 | Secondary malignant neoplasm of uterus | B58y100 |
| 11424 | Compensated cardiac failure | G580300 |
| 80221 | PHARYNX CARconfidence intervalNOMA | 149 C |
| 26503 | Asthma causes daytime symptoms most days | 663v.00 |
| 39478 | Wood asthma | H35y700 |
| 99631 | Chronic pyelonephritis without medullary necrosis | K100000 |
| 42729 | Type I diabetes mellitus with hypoglycaemic coma | C108E11 |
| 70766 | Type 1 diabetes mellitus with hypoglycaemic coma | C108E12 |
| 37328 | Malignant neoplasm of vagina | B450.00 |
| 4403 | Liver metastases | B577.11 |
| 79198 | MALIGNANT NEOPLASM URETER | 1892A |
| 63912 | Lung disease with diseases EC NOS | H57yz00 |
| 2906 | Congestive cardiac failure | G580.11 |
| 57191 | [X]Malignant neoplasm/other specified male genital organs | Byu8000 |
| 78015 | PRESENILE DEMENTIA | 2901A |
| 9622 | Malignant neoplasm of cauda equina | B525.00 |
| 102783 | Chronic neutrophilic leukaemia |  |
| 18658 | Secondary and unspec malig neop common iliac lymph nodes | B562300 |
| 47409 | Type II diabetes mellitus with polyneuropathy | C109B11 |
| 89508 | MALIGNANT LYMPHANGIOMA | 1719LM |
| 106360 | Erectile dysfunction due to diabetes mellitus |  |
| 91319 | MALIGNANT NEOPLASM ETHMOID (SINUS) | 1608A |
| 51786 | Malignant neoplasm of submandibular gland | B021.00 |
| 78034 | BIRD FANconfidence intervalER'S LUNG | L5161B |
| 43781 | Malignant neoplasm of dorsum of tongue NOS | B011z00 |
| 30054 | Acute gastric ulcer with haemorrhage | J110100 |
| 35014 | Sezary's disease | B622.00 |
| 104857 | Acute renal failure due to ACE inhibitor |  |
| 6116 | CVA - Cerebrovascular accident unspecified | G66..13 |
| 6253 | Stroke unspecified | G66..12 |
| 1298 | CVA unspecified | G66..11 |
| 88061 | ULCER GASTROJEJUNAL | 5349GJ |
| 17689 | Silent myocardial infarction | G30..17 |
| 1677 | MI - acute myocardial infarction | G30..15 |
| 30421 | Cardiac rupture following myocardial infarction (MI) | G30..13 |
| 49714 | Malignant neoplasm of spinal meninges | B523.00 |
| 14889 | Maturity onset diabetes | C100111 |
| 83975 | ULCER WITH GANGRENE | 4459CR |
| 18739 | Cryptogenic cirrhosis of liver | J615z12 |
| 106137 | Reticulosarcomas |  |
| 72212 | Malignant neoplasm of calcaneum | B308200 |
| 8828 | H/O: nephritis | 14D1.00 |
| 95421 | Malignant neoplasm of other specified female genital organ | B45y.00 |
| 100770 | Insulin dependent diabetes mellitus with diabetic cataract | C10EF12 |
| 95123 | Chronic kidney disease stage 3 without proteinuria | 1Z1C.00 |
| 107112 | Rheumatoid arthritis of IP joint of toe |  |
| 51311 | Other specified cerebrovascular disease | G6y..00 |
| 95175 | Chronic kidney disease stage 3A without proteinuria | 1Z1E.00 |
| 62828 | Secondary malignant neoplasm of other urinary organ NOS | B581z00 |
| 9984 | Carcinoma of lip | B00..11 |
| 15058 | H/O: heart failure | 14A6.00 |
| 24852 | Malignant neoplasm of lingual tonsil | B016.00 |
| 70821 | Diabetes mellitus NOS with other specified manifestation | C10yz00 |
| 56912 | Arteriosclerotic dementia with delirium | E004100 |
| 68236 | Malignant neoplasm of head, neck and face | B550.00 |
| 88461 | PLEURA NEOPLASM MALIGNANT PRIMARY | 1630A |
| 11129 | O/E - left eye background diabetic retinopathy | 2BBQ.00 |
| 93436 | Unspecified duodenal ulcer with haemorrhage and perforation | J12y300 |
| 105719 | Occusion of brachial artery |  |
| 50863 | Rheumatoid arthritis of knee | N040D00 |
| 46150 | Type 2 diabetes mellitus with gangrene | C109512 |
| 62107 | Type II diabetes mellitus with gangrene | C109511 |
| 96429 | Malignant neoplasm of undescended testis NOS | B470z00 |
| 46436 | Rheumatoid lung disease | N042100 |
| 77055 | TRACHEOBRONCHITIS | 490 T |
| 54493 | Malignant neoplasm of xiphoid process | B303500 |
| 12213 | Patient on maximal tolerated therapy for diabetes | 8BL2.00 |
| 11628 | Cancer of bowel | B1z0.11 |
| 23552 | Felty's syndrome | N041.00 |
| 71139 | Malignant neoplasm of other parts of brain | B51y.00 |
| 7323 | Uncomplicated senile dementia | E000.00 |
| 7323 | Uncomplicated senile dementia | e000.00 |
| 48730 | Acute duodenal ulcer with haemorrhage and perforation | J120300 |
| 31209 | Myopathy due to rheumatoid arthritis | F396400 |
| 88899 | RHEUMATISM NONARTICULAR SHOULDER | 7179GB |
| 80200 | RHEUMATISM MUSCULAR ARM | 7179GA |
| 15304 | Ruptured aortic aneurysm NOS | G715.00 |
| 20364 | Recurrent bronchiectasis | H340.00 |
| 35963 | Malignant neoplasm of lateral wall of urinary bladder | B492.00 |
| 32679 | Bronchiectasis NOS | H34z.00 |
| 26504 | Asthma never restricts exercise | 663f.00 |
| 64036 | Hodgkin's sarcoma | B612.00 |
| 10358 | Malignant neoplasm of upper lobe, bronchus or lung | B222.00 |
| 92612 | MALIGNANT NEOPLASM SPINAL CORD | 1922A |
| 105944 | Mised cell rhabdomyosarcoma |  |
| 41523 | Malignant neoplasm of middle lobe bronchus | B223000 |
| 75123 | CORONARY INFARCTION | 4109NC |
| 36342 | Mesangioproliferative glomerulonephritis NEC | K032y13 |
| 49875 | Malignant neoplasm of meninges, unspecified | B52X.00 |
| 97577 | Burkitt's lymphoma of intra-abdominal lymph nodes | B602300 |
| 19423 | Malignant neoplasm of male breast | B35..00 |
| 106020 | Occulsion of artery of lower limb |  |
| 105841 | Nodula sclerosis classical Hodgkin lymphoma |  |
| 61695 | Malignant neoplasm of cervical oesophagus | B100.00 |
| 70658 | Rheumatoid arthritis of talonavicular joint | N040H00 |
| 79973 | HEPATIC COMA | 573 B |
| 63470 | Malignant neoplasm of abdominal oesophagus | B102.00 |
| 54171 | Malignant neoplasm of middle third of oesophagus | B104.00 |
| 22536 | Chronic pulmonary fibrosis following radiation | H4y1000 |
| 45073 | Intrinsic asthma NOS | H331z00 |
| 64557 | Malignant neoplasm of cerebral peduncle | B517000 |
| 58962 | Malignant immunoproliferative small intestinal disease | B62x500 |
| 85530 | LINGUAL/TONGUE CANCER | 1419AA |
| 26108 | Steroid induced diabetes mellitus without complication | C10B000 |
| 77589 | RHEUMATISM HANDS | 718 AH |
| 50292 | [X]Malignant neoplasm of mediastinum, part unspecified | Byu2500 |
| 89329 | [X]Other specified leukaemias | ByuD800 |
| 53810 | [X]Other intracerebral haemorrhage | Gyu6200 |
| 70463 | Malignant neoplasm of connective and soft tissue of buttock | B315000 |
| 85537 | DIABETIC Aconfidence intervalDOSIS | 250 JA |
| 77511 | KETOSIS DIABETIC | 250 JL |
| 74809 | KETOAconfidence intervalDOSIS DIABETIC | 250 JK |
| 9491 | Anal carcinoma | B142.11 |
| 91998 | MALIGNANT NEOPLASM SMALL INTESTINE | 1529A |
| 85355 | CARconfidence intervalNOMA SMALL INTESTINE | 1529C |
| 84787 | SARCOMA SMALL INTESTINE | 1529B |
| 73532 | Hodgkin's, lymphocytic-histiocytic pred intra-abdominal node | B613300 |
| 95179 | Chronic kidney disease stage 3B | 1Z16.00 |
| 106867 | Non-follicular lymphoma |  |
| 80831 | AIDS | 799MD |
| 66092 | Unspecified gastrojejunal ulcer NOS | J14yz00 |
| 35285 | [X]Malignant neoplasm of eye, brain and other parts of cent | ByuA.00 |
| 76213 | SENILE DEMENTIA | 2900 |
| 106569 | Malignant neoplasm of crystalline lens |  |
| 63625 | Hodgkin's lymphocytic depletion lymph nodes axilla and arm | B616400 |
| 32856 | Acute peptic ulcer | J130.00 |
| 18678 | Malignant neoplasm of lower lobe bronchus | B224000 |
| 60053 | [X]Additional neoplasm classification terms | Byu..00 |
| 49148 | Malignant neoplasm, overlapping lesion of breast | B347.00 |
| 100083 | Neuroblastoma | B546.00 |
| 41362 | Malignant neoplasm of thoracic oesophagus | B101.00 |
| 59375 | Secondary malignant neoplasm of brain or spinal cord NOS | B583z00 |
| 233 | Severe asthma attack | H33z011 |
| 62399 | Malig neop skin of ear and external auricular canal NOS | B332z00 |
| 49828 | Malignant neoplasm of fallopian tube | B441.00 |
| 79947 | LUPUS ERYTHEMATOSUS DISSEMINATED | 7341 |
| 9494 | Biliary cirrhosis | J616.00 |
| 70729 | Malignant neoplasm of isthmus of uterine body NOS | B431z00 |
| 83884 | LIPOSARCOMA | 1992L |
| 63830 | Stenosis of precerebral arteries | G63..12 |
| 2961 | Seminoma of testis | B47z.11 |
| 15989 | Teratoma of testis | B47z.12 |
| 64406 | Malignant neoplasm of skin of thumb | B336500 |
| 59381 | Malignant neoplasm of iris | B500100 |
| 80819 | GANGRENE TOE | 4459TE |
| 41881 | Mesangiocapillary glomerulonephritis NEC | K032y14 |
| 73026 | Chronic neph syn difus mesangiocapillary glomerulonephritis | K0A3500 |
| 26034 | Other malignant neoplasm NOS | B591.00 |
| 21786 | Seminoma of descended testis | B471000 |
| 88172 | WEAK HEART | 7824BW |
| 43087 | Malignant neoplasm of eyelid including canthus | B331.00 |
| 102688 | Other Malignant immunoproliferative disease |  |
| 57446 | Malignant neoplasm of skin of trunk, excluding scrotum | B335.00 |
| 40740 | [X]Malignant neoplasms of lymphoid, haematopoietic and rela | ByuD.00 |
| 94597 | [V]Personal history of lymphoid leukaemia | ZV10611 |
| 4218 | Malignant neoplasm of parathyroid gland | B541.00 |
| 66058 | [X]Other emphysema | Hyu3000 |
| 51697 | Secondary pancreatic diabetes mellitus | C10G.00 |
| 1549 | Type 1 diabetes mellitus | C10E.00 |
| 104619 | Chronic Kidney disease |  |
| 32372 | Malignant neoplasm of thoracic vertebra | B302100 |
| 19321 | Malignant neoplasm of connective and soft tissue of hand | B311300 |
| 91064 | SECONDARY NEOPLASTIC DEPOSITS MULTI SITE | 1968M |
| 96751 | [X]HIV disease result/haematological???? abnorms,NEC | AyuCB00 |
| 3811 | Malignant neoplasm of caecum | B134.00 |
| 102947 | Ischaemic nephropathy |  |
| 78607 | BRONCHITIS ACUTE ON CHRONIC | 491 AC |
| 101707 | Malignant neoplasm of lower lip, vermilion border NOS | B001z00 |
| 6170 | Carcinomatosis | B590.11 |
| 62761 | Malignant neoplasm of septum of nose | B200200 |
| 25627 | Type 2 diabetes mellitus - poor control | C10F700 |
| 29317 | Recurrent duodenal ulcer | J124.00 |
| 64462 | Malignant neoplasm of posterior pharynx | B083.00 |
| 95145 | CKD stage 3 with proteinuria | 1Z1B.11 |
| 51873 | Malignant melanoma of thigh | B327100 |
| 56925 | [X]Malignant melanoma of other???? parts of face | Byu4000 |
| 19415 | Malignant neoplasm of lip, oral cavity and pharynx | B0...00 |
| 91267 | FOLLICULAR LYMPHOBLASTOMA | 2020FB |
| 88304 | LYMPHOID FOLLICULAR RETICULOSIS | 2020FR |
| 27853 | HIV disease resulting in Kaposi's sarcoma | A789500 |
| 16410 | Other emphysema NOS | H32yz00 |
| 55239 | Type 1 diabetes mellitus with gastroparesis | C10EQ00 |
| 80810 | MALIGNANT NEOPLASM TESTIS | 186 A |
| 26261 | [V]Personal history of peptic ulcer | ZV12711 |
| 77829 | TERATOMA TESTIS | 186 T |
| 18219 | Type II diabetes mellitus | C109.13 |
| 38914 | Lymphoid leukaemia NOS | B64z.00 |
| 65180 | Diffuse non-Hodgkin's lymphoma undifferentiated (diffuse) | B627800 |
| 18613 | Malignant neoplasm of duodenum | B120.00 |
| 64427 | Unspec malig neop lymphoid/histiocytic lymph node head/neck | B62z100 |
| 16704 | Malignant neoplasm of vertebral column | B302.00 |
| 60162 | [X]Malignant neoplasm overlapping lesion of skin | Byu5A00 |
| 66166 | Malignant neoplasm, overlapping lesion of small intestine | B124.00 |
| 26306 | Chronic bullous emphysema | H320.00 |
| 18001 | Acute duodenal ulcer with haemorrhage | J120100 |
| 89657 | Malignant mast cell tumour NOS | B626z00 |
| 71147 | Malignant neoplasm of lower lip, inner aspect | B003.00 |
| 19372 | Lymphoid leukaemia | B64..00 |
| 96869 | Malignant neoplasm of posterior wall of nasopharynx NOS | B071z00 |
| 96783 | Malignant neoplasm of commissure of lip | B005.00 |
| 12335 | Malignant lymphoma NOS | B62y.00 |
| 53200 | Diabetes mellitus, juvenile type, with ketoacidosis | C101000 |
| 89791 | SECONDARY NEOPLASTIC DEPOSITS PLEURA | 1972B |
| 83051 | CARconfidence intervalNOMA RENAL PELVIS | 1891C |
| 36899 | Malignant melanoma of toe | B327800 |
| 79175 | GLIOMA | 1929GL |
| 100139 | History of myocardial infarction | 14AT.00 |
| 66083 | Secondary malig neop of respiratory or digestive system NOS | B57z.00 |
| 34926 | Letterer-Siwe disease | B625.00 |
| 3604 | Non - Hodgkin's lymphoma | B627.00 |
| 64680 | Secondary malignant neoplasm of small intestine and duodenum | B574.00 |
| 65460 | Malignant neoplasm of spleen NEC | B1z1.00 |
| 103995 | Malignant neoplasm of aortic body |  |
| 56918 | Malignant neoplasm other spec digestive tract and peritoneum | B1zy.00 |
| 91718 | PITUITARY MALIGNANT NEOPLASM | 1943A |
| 18143 | Type II diabetes mellitus with arthropathy | C109G11 |
| 20122 | Spastic hemiplegia | F221.00 |
| 29386 | [X]Dementia in Alzheimer's disease, unspecified | Eu00z00 |
| 9118 | Colonic cancer | B13z.11 |
| 56513 | Malignant neoplasm of femur | B307000 |
| 5627 | Hay fever with asthma | H330011 |
| 33997 | Malignant neoplasm of skin of auricle (ear) | B332000 |
| 83963 | ULCER PEPTIC STOMACH PERFORATED | 5310PT |
| 57729 | [M]Lymphangiosarcoma | BBU1.00 |
| 67700 | Monoblastic leukaemia | B66..12 |
| 81601 | NEUROBLASTOMA | 1925NB |
| 42714 | Malignant melanoma of ankle | B327500 |
| 3535 | Intracerebral haemorrhage NOS | G61z.00 |
| 28919 | Malignant neoplasm of cerebral meninges | B521.00 |
| 7059 | Admit diabetic emergency | 8H2J.00 |
| 74832 | COLON CARconfidence intervalNOMA | 1538C |
| 107643 | T-cell prolymphocytic leukaemia |  |
| 23532 | Thoracic aortic aneurysm without mention of rupture | G712.00 |
| 37805 | Malignant neoplasm of cricoid cartilage | B213100 |
| 91357 | MALIGNANT NEOPLASM LIP | 1409A |
| 76824 | LIP CARconfidence intervalNOMA | 1409C |
| 46529 | Attends asthma monitoring | 9OJ1.00 |
| 73962 | Malignant neoplasm of upper lip, vermilion border | B000.00 |
| 44424 | Oesophageal varices in diseases EC | G852.00 |
| 103708 | Round cell liposarcoma |  |
| 95644 | Malignant neoplasm of heart | B241.00 |
| 61149 | Hodgkin's nodular sclerosis of intra-abdominal lymph nodes | B614300 |
| 52190 | Secondary and unspec malig neop pulmonary lymph nodes | B561900 |
| 108007 | Type 1 diabetes |  |
| 60772 | Malignant neoplasm of vagina NOS | B450z00 |
| 104913 | Malig neop connective soft tissue upper limb |  |
| 64810 | Malignant neoplasm of thorax NOS | B551z00 |
| 95545 | Maltoma | B627911 |
| 7229 | Asthma prophylactic medication used | 663W.00 |
| 87583 | MALIGNANT CHOLESTEATOMA | 1601AC |
| 26813 | Malignant neoplasm of larynx, other specified site | B21y.00 |
| 97332 | Malignant neoplasm of laryngeal cartilage NOS | B213z00 |
| 102158 | Letterer-Siwe disease of intrathoracic lymph nodes | B625200 |
| 50199 | Secondary and unspec malig neop axilla and upper limb LN | B563.00 |
| 96094 | Siewert type III adenocarcinoma | B119.00 |
| 74027 | DIABETES MELLITUS INSULIN DEPENDANT | 250 AD |
| 90669 | ABSCESS DIABETIC | 250 AB |
| 74167 | DIABETES | 250 AN |
| 74767 | MATURITY ONSET DIABETES MELLITUS INSULIN | 250 AK |
| 81641 | DIABETIC AMYOTROPHY | 250 AT |
| 44108 | Malignant neoplasm of retroperitoneum and peritoneum | B18..00 |
| 32898 | Admit heart failure emergency | 8H2S.00 |
| 41577 | H/O: cerebrovascular disease | 1477 |
| 24342 | Acute gastric ulcer unspecified | J110y00 |
| 107017 | Chronis lymphocytic |  |
| 50289 | Malignant neoplasm of heart NOS | B241z00 |
| 64497 | [X]Malignant neoplasm of uterine adnexa, unspecified | Byu7000 |
| 19140 | Hodgkin's nodular sclerosis of lymph nodes of multiple sites | B614800 |
| 10562 | Acute non-ST segment elevation myocardial infarction | G307100 |
| 84221 | HAEMORRHAGE INTRACEREBRAL WITH HYPERTENS | 4310 |
| 53528 | Secondary malignant neoplasm of urethra | B581200 |
| 91567 | NEOPLASM MALIGNANT SUBCUTANEOUS TISSUE | 1719AC |
| 102740 | Type 1 diabetes |  |
| 40557 | Malignant neoplasm of tongue NOS | B01z.00 |
| 95057 | Malignant neoplasm of ectopic site of female breast | B34y000 |
| 92371 | Malignant neoplasm of radius | B304300 |
| 74462 | SQUAMOUS CELL CARconfidence intervalNOMA | 1739CS |
| 108267 | Occlusion of anterior tibial artery |  |
| 86274 | PLEURA MESOTHELIOMA | 1639MA |
| 66639 | Malignant neoplasm of clavicle | B303200 |
| 59520 | Malignant neoplasm of malar bone | B300200 |
| 65642 | Malignant histiocytosis of intra-abdominal lymph nodes | B623300 |
| 55015 | Malignant neoplasm of mouth NOS | B05z.00 |
| 58088 | Malignant neoplasm of intrahepatic gall duct | B151400 |
| 41020 | Absent from work or school due to asthma | 66YC.00 |
| 18617 | Malignant neoplasm of brain | B51..00 |
| 27345 | Bird-fancier's lung | H352.00 |
| 506 | Non-insulin dependent diabetes mellitus | C100112 |
| 15588 | Farmers' lung | H350.00 |
| 65605 | Malignant neoplasm of myocardium | B241200 |
| 72127 | Malignant neoplasm of epididymis | B484.00 |
| 79856 | GASTRIC CANCER | 1519AA |
| 55434 | Malignant neoplasm of greater curve of stomach unspecified | B116.00 |
| 78930 | ASTHMA ALLERGIC GRASS | 493 GR |
| 84251 | ASTHMA POLLEN INITIATED | 493 GS |
| 77748 | FOLLICULAR LYMPHOMA | 2020FL |
| 43572 | Malignant neoplasm of body of stomach | B114.00 |
| 16408 | Healed myocardial infarction | G32..11 |
| 17464 | Personal history of myocardial infarction | G32..12 |
| 7058 | Emergency admission, asthma | 8H2P.00 |
| 53103 | Malignant neoplasm of endocervical gland | B410100 |
| 19393 | [X]Vascular dementia, unspecified | Eu01z00 |
| 91035 | Malignant neoplasm of fixed part of tongue NOS | B010z00 |
| 63582 | Chronic gastric ulcer with haemorrhage | J111100 |
| 99572 | Malignant neoplasm of connective and soft tissue of toe | B312500 |
| 1986 | Cancer of ovary | B440.11 |
| 1029 | Rheumatism unspecified | N240000 |
| 67575 | HIV disease resulting in unspecified malignant neoplasm | A788W00 |
| 63365 | Rheumatoid arthritis of distal radio-ulnar joint | N040600 |
| 42460 | Malignant neoplasm of pineal gland | B543.00 |
| 49301 | Malignant neoplasm lymphatic or haematopoietic tissue NOS | B6z..00 |
| 19318 | Malignant neoplasm of pyloric antrum of stomach | B112.00 |
| 50035 | Malignant neoplasm of aortic body and other paraganglia | B545.00 |
| 63988 | Malignant neoplasm of connective and soft tissue of thumb | B311500 |
| 32022 | Malignant neoplasm of cardia of stomach | B110.00 |
| 99430 | Chronic peptic ulcer without mention of complication | J131000 |
| 92668 | SCLERODERMA DIFFUSE | 7340C |
| 41215 | Malignant neoplasm of pyloric canal of stomach | B111100 |
| 2340 | Diabetic amyotrophy | F381311 |
| 59092 | Malignant neoplasm of pylorus of stomach NOS | B111z00 |
| 59382 | Malignant neoplasm of soft tissue of head | B310000 |
| 22884 | Type II diabetes mellitus | C10F.11 |
| 92700 | MALIGNANT NEOPLASM TONSIL | 1460A |
| 108072 | Myopathy due to disseminated lupus erythematosus | F396100 |
| 16298 | Malignant neoplasm of retroperitoneum and peritoneum NOS | B18z.00 |
| 59991 | Maturity onset diabetes in youth type 2 | C10D.11 |
| 4669 | Chronic focal glomerulonephritis | K02y200 |
| 80422 | PLEURAL EFFUSION MALIGNANT SECONDARY | 1972A |
| 101907 | Overlapping malign lesion of retroperitoneum and peritoneum | B182.00 |
| 49869 | Type 2 diabetes mellitus with arthropathy | C109G12 |
| 54278 | Secondary and unspec malig neop superficial inguinal LN | B564000 |
| 5387 | Other specified anterior myocardial infarction | G301.00 |
| 104720 | Sarcomatoid mesothelioma | BBP3.11 |
| 57854 | Malignant neoplasm of inguinal region NOS | B553000 |
| 106134 | Meningeal sarcomatosis | BBdB.00 |
| 67497 | Malignant neoplasm, overlapping lesion of oesophagus | B106.00 |
| 24301 | Secondary carcinoma of respiratory and/or digestive systems | B57..12 |
| 6471 | Metastases of respiratory and/or digestive systems | B57..11 |
| 90290 | Malignant neoplasm of mesentery | B18y700 |
| 97849 | Insulin dependent diabetes maturity onset | C10E912 |
| 97863 | Hodgkin's disease, mixed cellularity of unspecified site | B615000 |
| 67082 | Unspecified peptic ulcer without mention of complication | J13y000 |
| 37859 | Malignant neoplasm of cardia of stomach NOS | B110z00 |
| 82527 | MYCOSIS FUNGOIDES | 2021 |
| 80636 | LYMPHOMA GIANT FOLLICULAR | 2020 |
| 76436 | LYMPHOMA | 2022 |
| 60247 | Malig neop of connective and soft tissue of abdomen NOS | B314z00 |
| 3968 | Malignant neoplasm of female breast | B34..00 |
| 99951 | Reticulosarcoma or lymphosarcoma NOS | B60z.00 |
| 90546 | Malig neop connective and soft tissue hip and leg NOS | B312z00 |
| 101086 | Malignant neoplasm of cranial nerves NOS | B520z00 |
| 83607 | LEUKAEMIA CHRONIC | 2071 |
| 8492 | Hemiplegia NOS | F22z.00 |
| 106040 | Occlusion of radial artery | G783000 |
| 54103 | Malignant neoplasm gallbladder and extrahepatic bile ducts | B16..00 |
| 106511 | Subarachnoid h’ge inj + open intracran wnd + concussion unspec | S621z00 |

## Other behavioural disorder (ICD-10 F90-98)

Prevalence of other behavioural disorders was rare in the CPRD (e.g. ≤1% prevalence) and therefore was included in the other mental disorders category.

Other behavioural disorder code list

| Medical code | Read code | Read term |
| --- | --- | --- |
| 35871 | Eu91000 | [X]Conduct disorder confined to the family context |
| 33505 | Eu90100 | [X]Hyperkinetic conduct disorder |
| 6126 | Eu91200 | [X]Socialized conduct disorder |
| 33906 | Eu91111 | [X]Conduct disorder, solitary aggressive type |
| 41842 | E2C2z00 | Socialised conduct disorder NOS |
| 46452 | E2C1.00 | Nonaggressive unsocial conduct disorder |
| 35005 | Eu91100 | [X]Unsocialized conduct disorder |
| 43359 | E2C2.00 | Socialised conduct disorder |
| 2040 | E2C0.00 | Aggressive unsocial conduct disorder |
| 20182 | E2C1z00 | Nonaggressive unsocial conduct disorder NOS |
| 24352 | E2C0z00 | Aggressive unsocial conduct disorder NOS |
| 32845 | Eu92000 | [X]Depressive conduct disorder |
| 72887 | Eu91z12 | [X]Childhood conduct disorder NOS |
| 3787 | Eu91.00 | [X]Conduct disorders |
| 95346 | Eu92y11 | [X]Conduct disorder associated with emotional disorder |
| 103242 | Eu92y12 | [X]Conduct disorder associated with neurotic disorder |
| 34977 | Eu91z00 | [X]Conduct disorder, unspecified |
| 49615 | Eu91y00 | [X]Other conduct disorders |
| 45799 | Eu90111 | [X]Hyperkinetic disorder associated with conduct disorder |
| 45263 | E2E2.00 | Hyperkinetic conduct disorder |
| 6511 | Eu92z00 | [X]Mixed disorder of conduct and emotions, unspecified |
| 4919 | Eu92.00 | [X]Mixed disorders of conduct and emotions |
| 7305 | Eu92y00 | [X]Other mixed disorders of conduct and emotions |
| 44577 | Eu93.00 | [X]Emotional disorders with onset specific to childhood |
| 64400 | Eu93y00 | [X]Other childhood emotional disorders |
| 24351 | Eu93100 | [X]Phobic anxiety disorder of childhood |
| 29907 | Eu93200 | [X]Social anxiety disorder of childhood |
| 18032 | Eu93000 | [X]Separation anxiety disorder of childhood |
| 37757 | Eu93300 | [X]Sibling rivalry disorder |
| 61430 | Eu93y12 | [X]Childhood overanxious disorder |
| 50238 | Eu93z00 | [X]Childhood emotional disorder, unspecified |
| 45406 | Eu94z00 | [X]Childhood disorder of social functioning, unspecified |
| 39480 | Eu94y00 | [X]Other childhood disorders of social functioning |
| 36541 | E2D2211 | Mutism of childhood or adolescence |
| 99609 | ZS7C700 | Post-traumatic mutism |
| 7716 | E29y300 | Elective mutism due to an adjustment reaction |
| 36500 | Eu94000 | [X]Elective mutism |
| 31865 | Eu94011 | [X]Selective mutism |
| 56465 | E2D2200 | Childhood and adolescent disturbance with elective mutism |
| 34142 | Eu94100 | [X]Reactive attachment disorder of childhood |
| 33976 | Eu94200 | [X]Disinhibited attachment disorder of childhood |
| 27571 | E272200 | Chronic motor tic disorder |
| 43751 | Eu95000 | [X]Transient tic disorder |
| 15164 | E272000 | Tic disorder unspecified |
| 42827 | Eu95100 | [X]Chronic motor or vocal tic disorder |
| 16726 | Eu95200 | [X]Comb vocal multiple motor tic disorder - de la Tourette |
| 66949 | Eu95y00 | [X]Other tic disorders |
| 21429 | Eu95.00 | [X]Tic disorders |
| 44322 | Eu95z00 | [X]Tic disorder, unspecified |
| 16093 | E276.00 | Non-organic enuresis |
| 55760 | E276100 | Non-organic secondary enuresis |
| 28525 | Z9EA111 | Provision of enuresis alarm |
| 35708 | E276z00 | Non-organic enuresis NOS |
| 11096 | 8O6..00 | Enuresis support |
| 11400 | 8D73.12 | Enuresis alarm |
| 4375 | R083000 | [D]Enuresis NOS |
| 2213 | 1A22000 | Nocturnal enuresis |
| 4058 | Eu9y000 | [X]Nonorganic enuresis |
| 10423 | 1A22100 | Daytime enuresis |
| 100168 | 9No6.00 | Seen in enuresis clinic |
| 55818 | E276000 | Non-organic primary enuresis |
| 98187 | 8HTt.00 | Referral to enuresis clinic |
| 659 | 1A22.00 | Enuresis |
| 11903 | Z9MO.00 | Enuresis support |
| 29557 | 19E2.00 | Soiling - encopresis |
| 70301 | E277000 | Non-organic continuous encopresis |
| 7874 | 19E2.11 | Encopresis symptom |
| 6120 | Eu9y100 | [X]Nonorganic encopresis |
| 2134 | R076000 | [D]Encopresis NOS |
| 3788 | E277.00 | Non-organic encopresis |
| 69256 | E277z00 | Non-organic encopresis NOS |
| 21270 | Eu9y200 | [X]Feeding disorder of infancy and childhood |
| 52580 | Eu9y300 | [X]Pica of infancy and childhood |
| 19740 | Eu9y400 | [X]Stereotyped movement disorders |
| 2393 | E270.00 | Stammering or stuttering |
| 104021 | ZRkv.00 | Stuttering intervention programme |
| 25667 | Eu9y500 | [X]Stuttering [stammering] |
| 5227 | E270.12 | Stuttering |
| 93983 | Eu9y600 | [X]Cluttering |
| 71766 | ZS12.11 | Cluttering |
| 103196 | ZS12.00 | Cluttering - speech |
|  |  |  |

## Personality disorder (ICD-10 F60-61)

Prevalence of personality disorders was rare in the CPRD (e.g. ≤1% prevalence) and therefore was included in the other mental disorders category.

Personality disorder code list

| Medical code | Read code | Read term |
| --- | --- | --- |
| 2076 | E21..00 | Personality disorders |
| 28227 | E21..11 | Neurotic personality disorder |
| 5652 | E210.00 | Paranoid personality disorder |
| 12228 | E211100 | Hypomanic personality disorder |
| 10455 | E211200 | Depressive personality disorder |
| 3369 | E212.00 | Schizoid personality disorder |
| 67130 | E212000 | Unspecified schizoid personality disorder |
| 14747 | E212z00 | Schizoid personality disorder NOS |
| 23597 | E213.00 | Explosive personality disorder |
| 6339 | E213.11 | Aggressive personality |
| 20881 | E214.00 | Compulsive personality disorders |
| 40057 | E214.11 | Anancastic personality |
| 30395 | E214000 | Anankastic personality |
| 1293 | E214100 | Obsessional personality |
| 34456 | E214z00 | Compulsive personality disorder NOS |
| 27481 | E215.00 | Histrionic personality disorders |
| 4759 | E215.11 | Hysterical personality disorders |
| 44242 | E215000 | Unspecified histrionic personality disorder |
| 3709 | E215200 | Emotionally unstable personality |
| 67590 | E215300 | Psychoinfantile personality |
| 60522 | E215z00 | Histrionic personality disorder NOS |
| 4515 | E216.00 | Inadequate personality disorder |
| 48796 | E216.11 | Asthenic personality |
| 21665 | E216.12 | Dependent personality |
| 19931 | E216.13 | Labile personality |
| 23977 | E217.00 | Antisocial or sociopathic personality disorder |
| 68042 | E217.11 | Amoral personality |
| 25146 | E21y.00 | Other personality disorders |
| 37289 | E21y000 | Narcissistic personality disorder |
| 35642 | E21y100 | Avoidant personality disorder |
| 18565 | E21y200 | Borderline personality disorder |
| 35763 | E21y300 | Passive-aggressive personality disorder |
| 27803 | E21y400 | Eccentric personality disorder |
| 1364 | E21y500 | Immature personality disorder |
| 70899 | E21y600 | Masochistic personality disorder |
| 21077 | E21y700 | Psychoneurotic personality disorder |
| 2729 | E21y711 | Neurotic personality |
| 15960 | E21yz00 | Other personality disorder NOS |
| 20033 | E21yz11 | Manipulative personality |
| 15098 | E21z.00 | Personality disorder NOS |
| 792 | E21z.11 | Psychopathic personality |
| 21005 | Eu6..00 | [X]Disorders of adult personality and behaviour |
| 50188 | Eu60.00 | [X]Specific personality disorders |
| 21338 | Eu60000 | [X]Paranoid personality disorder |
| 69000 | Eu60013 | [X]Querulant personality disorder |
| 48687 | Eu60014 | [X]Sensitive paranoid personality disorder |
| 38371 | Eu60100 | [X]Schizoid personality disorder |
| 31632 | Eu60200 | [X]Dissocial personality disorder |
| 105029 | Eu60211 | [X]Amoral personality disorder |
| 32869 | Eu60212 | [X]Antisocial personality disorder |
| 56502 | Eu60213 | [X]Asocial personality disorder |
| 21671 | Eu60214 | [X]Psychopathic personality disorder |
| 45188 | Eu60215 | [X]Sociopathic personality disorder |
| 7745 | Eu60300 | [X]Emotionally unstable personality disorder |
| 20839 | Eu60311 | [X]Aggressive personality disorder |
| 31789 | Eu60312 | [X]Borderline personality disorder |
| 58693 | Eu60313 | [X]Explosive personality disorder |
| 43690 | Eu60400 | [X]Histrionic personality disorder |
| 27945 | Eu60411 | [X]Hysterical personality disorder |
| 69185 | Eu60412 | [X]Psychoinfantile personality disorder |
| 52465 | Eu60500 | [X]Anankastic personality disorder |
| 38100 | Eu60511 | [X]Compulsive personality disorder |
| 22259 | Eu60512 | [X]Obsessional personality disorder |
| 17420 | Eu60513 | [X]Obsessive-compulsive personality disorder |
| 8424 | Eu60600 | [X]Anxious [avoidant] personality disorder |
| 31819 | Eu60700 | [X]Dependent personality disorder |
| 39535 | Eu60711 | [X]Asthenic personality disorder |
| 38031 | Eu60712 | [X]Inadequate personality disorder |
| 33741 | Eu60713 | [X]Passive personality disorder |
| 59008 | Eu60714 | [X]Self defeating personality disorder |
| 40104 | Eu60800 | [X]Addictive personality |
| 49600 | Eu60y00 | [X]Other specific personality disorders |
| 55969 | Eu60y11 | [X]Eccentric personality disorder |
| 71431 | Eu60y12 | [X]Haltlose type personality disorder |
| 49779 | Eu60y13 | [X]Immature personality disorder |
| 53335 | Eu60y14 | [X]Narcissistic personality disorder |
| 50348 | Eu60y16 | [X]Psychoneurotic personality disorder |
| 42496 | Eu60z00 | [X]Personality disorder, unspecified |
| 575674 | Eu60z11 | [X]Character neurosis NOS |
| 49721 | Eu60z12 | [X]Pathological personality NOS |
| 30603 | Eu61.00 | [X]Mixed and other personality disorders |
| 64838 | Eu6y.00 | [X]Other disorders of adult personality and behaviour |
| 34896 | Eu6yy00 | [X]Other specified disorders of adult personality/behaviour |
| 39777 | Eu6z.00 | [X]Unspecified disorder of adult personality and behaviour |

## Schizophrenia and related disorder (ICD-10 Chapters F20-F29)

Prevalence of schizophrenia and related disorders was rare in the CPRD (e.g. ≤1% prevalence) and therefore was included in the other mental disorders category.

Schizophrenia and related disorder code list

| Medical code | Read code | Read term |
| --- | --- | --- |
| 6325 | 1464 | H/O: schizophrenia |
| 12777 | 146H.00 | H/O: psychosis |
| 19345 | 212T.00 | Psychosis, schizophrenia + bipolar affective disord resolved |
| 88275 | 212W.00 | Schizophrenia resolved |
| 85972 | 212X.00 | Psychosis resolved |
| 4390 | 285..11 | Psychotic condition, insight present |
| 22644 | 286..11 | Poor insight into psychotic condition |
| 26178 | 8HHs.00 | Referral to psychosis early intervention service |
| 15958 | E1...00 | Non-organic psychoses |
| 854 | E10..00 | Schizophrenic disorders |
| 32222 | E100.00 | Simple schizophrenia |
| 73295 | E100.11 | Schizophrenia simplex |
| 15733 | E100000 | Unspecified schizophrenia |
| 23616 | E100100 | Subchronic schizophrenia |
| 3984 | E100200 | Chronic schizophrenic |
| 57666 | E100300 | Acute exacerbation of subchronic schizophrenia |
| 44498 | E100400 | Acute exacerbation of chronic schizophrenia |
| 58687 | E100500 | Schizophrenia in remission |
| 53625 | E100z00 | Simple schizophrenia NOS |
| 30619 | E101.00 | Hebephrenic schizophrenia |
| 66506 | E101000 | Unspecified hebephrenic schizophrenia |
| 97919 | E101400 | Acute exacerbation of chronic hebephrenic schizophrenia |
| 67768 | E101500 | Hebephrenic schizophrenia in remission |
| 48054 | E101z00 | Hebephrenic schizophrenia NOS |
| 25546 | E102.00 | Catatonic schizophrenia |
| 58716 | E102000 | Unspecified catatonic schizophrenia |
| 99199 | E102100 | Subchronic catatonic schizophrenia |
| 107222 | E102400 | Acute exacerbation of chronic catatonic schizophrenia |
| 102427 | E102500 | Catatonic schizophrenia in remission |
| 63867 | E102z00 | Catatonic schizophrenia NOS |
| 1494 | E103.00 | Paranoid schizophrenia |
| 33383 | E103000 | Unspecified paranoid schizophrenia |
| 104760 | E103100 | Subchronic paranoid schizophrenia |
| 31362 | E103200 | Chronic paranoid schizophrenia |
| 51322 | E103300 | Acute exacerbation of subchronic paranoid schizophrenia |
| 53032 | E103400 | Acute exacerbation of chronic paranoid schizophrenia |
| 36172 | E103500 | Paranoid schizophrenia in remission |
| 9281 | E103z00 | Paranoid schizophrenia NOS |
| 576 | E104.00 | Acute schizophrenic episode |
| 93167 | E104.11 | Oneirophrenia |
| 66410 | E105.00 | Latent schizophrenia |
| 102311 | E105000 | Unspecified latent schizophrenia |
| 94299 | E105200 | Chronic latent schizophrenia |
| 96883 | E105500 | Latent schizophrenia in remission |
| 102446 | E105z00 | Latent schizophrenia NOS |
| 38063 | E106.00 | Residual schizophrenia |
| 2117 | E107.00 | Schizo-affective schizophrenia |
| 99000 | E107.11 | Cyclic schizophrenia |
| 58862 | E107000 | Unspecified schizo-affective schizophrenia |
| 61098 | E107100 | Subchronic schizo-affective schizophrenia |
| 43800 | E107200 | Chronic schizo-affective schizophrenia |
| 58866 | E107300 | Acute exacerbation subchronic schizo-affective schizophrenia |
| 63478 | E107400 | Acute exacerbation of chronic schizo-affective schizophrenia |
| 56438 | E107500 | Schizo-affective schizophrenia in remission |
| 10575 | E107z00 | Schizo-affective schizophrenia NOS |
| 39062 | E10y.00 | Other schizophrenia |
| 92994 | E10y.11 | Cenesthopathic schizophrenia |
| 33338 | E10y000 | Atypical schizophrenia |
| 99070 | E10y100 | Coenesthopathic schizophrenia |
| 49761 | E10yz00 | Other schizophrenia NOS |
| 8407 | E10z.00 | Schizophrenia NOS |
| 4261 | E12..00 | Paranoid states |
| 14743 | E120.00 | Simple paranoid state |
| 3890 | E121.00 | Chronic paranoid psychosis |
| 14971 | E122.00 | Paraphrenia |
| 62680 | E123.00 | Shared paranoid disorder |
| 50868 | E123.11 | Folie a deux |
| 31589 | E12y.00 | Other paranoid states |
| 66766 | E12y000 | Paranoia querulans |
| 31455 | E12yz00 | Other paranoid states NOS |
| 12771 | E12z.00 | Paranoid psychosis NOS |
| 31984 | E13..00 | Other nonorganic psychoses |
| 20228 | E13..11 | Reactive psychoses |
| 15053 | E133.00 | Acute paranoid reaction |
| 68058 | E133.11 | Bouffee delirante |
| 24345 | E134.00 | Psychogenic paranoid psychosis |
| 16333 | E13y.00 | Other reactive psychoses |
| 23538 | E13y100 | Brief reactive psychosis |
| 26119 | E13yz00 | Other reactive psychoses NOS |
| 14965 | E13z.00 | Nonorganic psychosis NOS |
| 3636 | E13z.11 | Psychotic episode NOS |
| 43225 | E14..00 | Psychoses with origin in childhood |
| 56143 | E141.00 | Disintegrative psychosis |
| 16537 | E1y..00 | Other specified non-organic psychoses |
| 22188 | E1z..00 | Non-organic psychosis NOS |
| 61969 | E212200 | Schizotypal personality |
| 17281 | Eu2..00 | [X]Schizophrenia, schizotypal and delusional disorders |
| 34236 | Eu20.00 | [X]Schizophrenia |
| 16764 | Eu20000 | [X]Paranoid schizophrenia |
| 50060 | Eu20011 | [X]Paraphrenic schizophrenia |
| 43405 | Eu20100 | [X]Hebephrenic schizophrenia |
| 53985 | Eu20111 | [X]Disorganised schizophrenia |
| 61501 | Eu20200 | [X]Catatonic schizophrenia |
| 20572 | Eu20211 | [X]Catatonic stupor |
| 64533 | Eu20212 | [X]Schizophrenic catalepsy |
| 35877 | Eu20213 | [X]Schizophrenic catatonia |
| 31493 | Eu20214 | [X]Schizophrenic flexibilatis cerea |
| 60013 | Eu20300 | [X]Undifferentiated schizophrenia |
| 91547 | Eu20311 | [X]Atypical schizophrenia |
| 20785 | Eu20400 | [X]Post-schizophrenic depression |
| 64264 | Eu20500 | [X]Residual schizophrenia |
| 24107 | Eu20511 | [X]Chronic undifferentiated schizophrenia |
| 35848 | Eu20600 | [X]Simple schizophrenia |
| 49420 | Eu20y00 | [X]Other schizophrenia |
| 94001 | Eu20y12 | [X]Schizophreniform disord NOS |
| 18053 | Eu20y13 | [X]Schizophrenifrm psychos NOS |
| 34966 | Eu20z00 | [X]Schizophrenia, unspecified |
| 39316 | Eu21.00 | [X]Schizotypal disorder |
| 91511 | Eu21.11 | [X]Latent schizophrenic reaction |
| 54387 | Eu21.12 | [X]Borderline schizophrenia |
| 64993 | Eu21.13 | [X]Latent schizophrenia |
| 62449 | Eu21.14 | [X]Prepsychotic schizophrenia |
| 40386 | Eu21.15 | [X]Prodromal schizophrenia |
| 49852 | Eu21.16 | [X]Pseudoneurotic schizophrenia |
| 71250 | Eu21.17 | [X]Pseudopsychopathic schizophrenia |
| 26859 | Eu21.18 | [X]Schizotypal personality disorder |
| 28562 | Eu22.00 | [X]Persistent delusional disorders |
| 34389 | Eu22000 | [X]Delusional disorder |
| 2113 | Eu22011 | [X]Paranoid psychosis |
| 11172 | Eu22012 | [X]Paranoid state |
| 47947 | Eu22013 | [X]Paraphrenia - late |
| 65127 | Eu22014 | [X]Sensitiver Beziehungswahn |
| 4843 | Eu22015 | [X]Paranoia |
| 62405 | Eu22100 | [X]Delusional misidentification syndrome |
| 55221 | Eu22111 | [X]Capgras syndrome |
| 98821 | Eu22200 | [X]Cotard syndrome |
| 101720 | Eu22300 | [X]Paranoid state in remission |
| 66077 | Eu22y00 | [X]Other persistent delusional disorders |
| 40981 | Eu22y11 | [X]Delusional dysmorphophobia |
| 50248 | Eu22y12 | [X]Involutional paranoid state |
| 55236 | Eu22y13 | [X]Paranoia querulans |
| 49223 | Eu22z00 | [X]Persistent delusional disorder, unspecified |
| 25019 | Eu23.00 | [X]Acute and transient psychotic disorders |
| 36720 | Eu23000 | [X]Acute polymorphic psychot disord without symp of schizoph |
| 50023 | Eu23011 | [X]Bouffee delirante |
| 21455 | Eu23012 | [X]Cycloid psychosis |
| 21595 | Eu23100 | [X]Acute polymorphic psychot disord with symp of schizophren |
| 26143 | Eu23112 | [X]Cycloid psychosis with symptoms of schizophrenia |
| 11778 | Eu23200 | [X]Acute schizophrenia-like psychotic disorder |
| 59096 | Eu23211 | [X]Brief schizophreniform disorder |
| 70884 | Eu23212 | [X]Brief schizophrenifrm psych |
| 94604 | Eu23214 | [X]Schizophrenic reaction |
| 44307 | Eu23300 | [X]Other acute predominantly delusional psychotic disorders |
| 27770 | Eu23312 | [X]Psychogenic paranoid psychosis |
| 44503 | Eu23y00 | [X]Other acute and transient psychotic disorders |
| 34168 | Eu23z00 | [X]Acute and transient psychotic disorder, unspecified |
| 31707 | Eu23z11 | [X]Brief reactive psychosis NOS |
| 29651 | Eu23z12 | [X]Reactive psychosis |
| 51302 | Eu24.00 | [X]Induced delusional disorder |
| 105606 | Eu24.11 | [X]Folie a deux |
| 47230 | Eu24.12 | [X]Induced paranoid disorder |
| 11973 | Eu24.13 | [X]Induced psychotic disorder |
| 9422 | Eu25.00 | [X]Schizoaffective disorders |
| 33847 | Eu25000 | [X]Schizoaffective disorder, manic type |
| 16905 | Eu25011 | [X]Schizoaffective psychosis, manic type |
| 51903 | Eu25012 | [X]Schizophreniform psychosis, manic type |
| 11055 | Eu25100 | [X]Schizoaffective disorder, depressive type |
| 35274 | Eu25111 | [X]Schizoaffective psychosis, depressive type |
| 41022 | Eu25112 | [X]Schizophreniform psychosis, depressive type |
| 33693 | Eu25200 | [X]Schizoaffective disorder, mixed type |
| 104763 | Eu25211 | [X]Cyclic schizophrenia |
| 37580 | Eu25212 | [X]Mixed schizophrenic and affective psychosis |
| 58532 | Eu25y00 | [X]Other schizoaffective disorders |
| 37681 | Eu25z00 | [X]Schizoaffective disorder, unspecified |
| 33410 | Eu25z11 | [X]Schizoaffective psychosis NOS |
| 30985 | Eu2y.00 | [X]Other nonorganic psychotic disorders |
| 31738 | Eu2y.11 | [X]Chronic hallucinatory psychosis |
| 11244 | Eu2z.00 | [X]Unspecified nonorganic psychosis |
| 694 | Eu2z.11 | [X]Psychosis NOS |
| 22104 | ZV11000 | [V]Personal history of schizophrenia |

## Self-harm

As defined by (ICD-10 (international classification of diseases, 10th revision) codes: X60-X84 (intentional self-harm) and Y10-Y34 (event of undetermined intent) excluding Y33.9 where verdict was still pending(1))

Self-harm code list

| Medical code | Read code | Read term |
| --- | --- | --- |
| 30370 | ZX1H100 | Self-strangulation |
| 28694 | ZX18.00 | Hanging self |
| 42086 | U20yz00 | [X]Intent self poison unspecif chemical unspecif place |
| 44508 | U204000 | [X]Int self poison/exposure to psychotropic drug at home |
| 69969 | TK10.00 | Suicide + selfinflicted poisoning by gas via pipeline |
| 22281 | ZX11.00 | Biting self |
| 93837 | U2C1.00 | [X]Int self harm jump/lying befr mov obje occ resid instit'n |
| 52458 | TK06.00 | Suicide + selfinflicted poisoning by agricultural chemical |
| 36255 | TK6..00 | Suicide and selfinflicted injury by cutting and stabbing |
| 36255 | TK6..00 | Suicide and selfinflicted injury by cutting and stabbing |
| 66063 | TKxy.00 | Suicide and selfinflicted injury by other specified means |
| 98594 | TKx3.00 | Suicide and selfinflicted injury by extremes of cold |
| 64744 | TKx5.00 | Suicide and selfinflicted injury by crashing motor vehicle |
| 96430 | TKx7.00 | Suicide and selfinflicted injury caustic subst, excl poison |
| 13557 | TK30.00 | Suicide and selfinflicted injury by hanging |
| 22199 | TK04.00 | Suicide + selfinflicted poisoning by other drugs/medicines |
| 48324 | U202.16 | [X]Overdose - benzodiazepine |
| 52931 | U202.15 | [X]Overdose - nitrazepam |
| 51381 | U202.13 | [X]Overdose - temazepam |
| 45748 | U202.12 | [X]Overdose - diazepam |
| 55395 | U202.11 | [X]Overdose - sleeping tabs |
| 45166 | U2D0.00 | [X]Intent self harm by crash of motor vehicl occurrn at home |
| 50482 | ZX1J.00 | Self-electrocution |
| 56681 | ZX1H.00 | Self-asphyxiation |
| 54091 | U210.00 | [X]Intent self harm by hanging strangulat/suffocat occ home |
| 70414 | U20A400 | [X]Int self poison org solvent,halogen hydrocarb,in highway |
| 65955 | U201.00 | [X]Intent self poison/exposure to antiepileptic |
| 96224 | U2zy.00 | [X]Intent self harm by unspecif means occ oth specif place |
| 101481 | U200400 | [X]Intent self pois nonopioid analgesic in street/highway |
| 90704 | ZX1L300 | Self-mutilation of penis |
| 70391 | U205y00 | [X]Int self poison narcotic drug other spec place |
| 61546 | U20y200 | [X]Int self poison unspecif chemical school/pub admin area |
| 100372 | U4Bz.00 | [X]Fall jump/push frm high plce undt intnt occ unspecif plce |
| 100372 | U4Bz.00 | [X]Fall jump/push frm high plce undt intnt occ unspecif plce |
| 10057 | ZX1..13 | Deliberate self-harm |
| 47623 | ZX1..12 | SIB - Self-injurious behaviour |
| 72747 | U270.00 | [X]Intention self harm by smoke fire/flames occurrn at home |
| 94442 | TK01400 | Suicide and self inflicted injury by Phenobarbitone |
| 64227 | ZX1K.11 | Setting fire to self |
| 69145 | ZX1K.12 | Setting self alight |
| 3406 | TK...17 | Para-suicide |
| 41241 | TKz..00 | Suicide and selfinflicted injury NOS |
| 18379 | U200.11 | [X]Overdose - paracetamol |
| 29861 | U200.13 | [X]Overdose - aspirin |
| 44886 | U200.12 | [X]Overdose - ibuprofen |
| 34703 | U204.12 | [X]Overdose - amitriptyline |
| 60404 | U221.00 | [X]Intent self harm by drowning/submersn occ resid instit'n |
| 38760 | U2y..00 | [X]Intentional self harm by other specified means |
| 15177 | TK60.00 | Suicide and selfinflicted injury by cutting |
| 15177 | TK60.00 | Suicide and selfinflicted injury by cutting |
| 38749 | U20B200 | [X]Int self poison other gas/vapour school/pub admin area |
| 68793 | U207.00 | [X]Intent self poison/exposure to oth autonomic drug |
| 2557 | TK05.00 | Suicide + selfinflicted poisoning by drug or medicine NOS |
| 28080 | TK07.00 | Suicide + selfinflicted poisoning by corrosive/caustic subst |
| 52778 | U29z.00 | [X]Intentional self harm by sharp object occ unspecif place |
| 54929 | ZX1M.00 | Shooting self |
| 27522 | TK03.00 | Suicide + selfinflicted poisoning tranquilliser/psychotropic |
| 72734 | U2B0.00 | [X]Intent self harm by jumping from high place occ at home |
| 56075 | U2B4.00 | [X]Intent self harm by jump from high place occ street/h'way |
| 104834 | TK2y.00 | Suicide + selfinflicted poisoning by other gases and vapours |
| 49135 | TK1y.00 | Suicide and selfinflicted poisoning by other utility gas |
| 94412 | TK1z.00 | Suicide + selfinflicted poisoning by domestic gases NOS |
| 51328 | TK21.00 | Suicide and selfinflicted poisoning by other carbon monoxide |
| 16485 | TK01.00 | Suicide + selfinflicted poisoning by barbiturates |
| 73666 | U26..00 | [X]Intentional self harm by explosive material |
| 30360 | U21..00 | [X]Intent self harm by hanging strangulation / suffocation |
| 25854 | ZX19.00 | Hitting self |
| 51309 | U20B.00 | [X]Intent self poison/exposure to other gas/vapour |
| 57479 | ZX1L200 | Self-mutilation of genitalia |
| 68788 | U205z00 | [X]Intent self poison narcotic drug unspecif place |
| 31854 | TK4..00 | Suicide and selfinflicted injury by drowning |
| 58901 | ZX1B200 | Jumping from bridge |
| 21029 | TK...00 | Suicide and selfinflicted injury |
| 41384 | ZX1B.00 | Jumping from height |
| 59405 | TKx0000 | Suicide + selfinflicted injury-jumping before moving object |
| 68790 | U202z00 | [X]Intent self poison sedative hypnotic unspecif place |
| 73603 | U28z.00 | [X]Intent self harm by steam hot vapour/obj occ unspec place |
| 62382 | ZX1K.00 | Self-incineration |
| 18983 | ZX1I.00 | Self-scalding |
| 54695 | U20By00 | [X]Int self poison other gas/vapour other spec place |
| 66915 | TK5..00 | Suicide and selfinflicted injury by firearms and explosives |
| 51292 | U202.00 | [X]Intent self poison/exposure to sedative hypnotic |
| 73776 | U207000 | [X]Int self poison/exposure to oth autonomic drug at home |
| 89371 | ZX1Q.11 | Jumping under train |
| 72792 | U206400 | [X]Intent self pois hallucinogen in street/highway |
| 66117 | U201z00 | [X]Intent self poison antiepileptic unspecif place |
| 54950 | U44..00 | [X]Rifle shotgun+larger firearm discharge undetermin intent |
| 45796 | U72..00 | [X]Sequel intentn self-harm assault+event of undeterm intent |
| 96729 | U208400 | [X]Intent self pois oth/unsp drug/medic in street/highway |
| 44530 | U202000 | [X]Int self poison/exposure to sedative hypnotic at home |
| 90857 | U21y.00 | [X]Intent self harm by hangng strangul/suffoct oth spec plce |
| 69342 | U2yz.00 | [X]Intent self harm by oth specif means occ unspecif place |
| 71661 | ZX...11 | Self-damage |
| 3423 | TK...12 | Injury - self-inflicted |
| 102479 | U206000 | [X]Int self poison/exposure to hallucinogen at home |
| 63099 | U2C4.00 | [X]Int self harm jump/lying befr mov obje occ street/highway |
| 28531 | U45..00 | [X]Other+unspecified firearm discharge undetermined intent |
| 35123 | ZX1L100 | Self-mutilation of hands |
| 5616 | TK3y.00 | Suicide + selfinflicted inj oth mean hang/strangle/suffocate |
| 95790 | U2B6.00 | [X]Int self harm by jump from high place indust/constr area |
| 28680 | ZX1LD00 | [X]Self mutilation |
| 46911 | ZRLfC12 | HoNOS item 2 - non-accidental self injury |
| 69343 | U202y00 | [X]Int self poison sedative hypnotic other spec place |
| 24463 | U20B.11 | [X]Self carbon monoxide poisoning |
| 49552 | U202.17 | [X]Overdose - barbiturate |
| 94725 | U202.18 | [X]Overdose - amobarbital |
| 42937 | TK71.00 | Suicide+selfinflicted injury-jump from oth manmade structure |
| 27713 | U20..00 | [X]Intentional self poisoning/exposure to noxious substances |
| 3246 | TK...15 | Attempted suicide |
| 10644 | TK...13 | Poisoning - self-inflicted |
| 6595 | TK...11 | Cause of overdose - deliberate |
| 68806 | U20C.00 | [X]Intent self poison/exposure to pesticide |
| 42097 | U2C..00 | [X]Intent self harm by jumping / lying before moving object |
| 37874 | ZX12.00 | Burning self |
| 38008 | U27z.00 | [X]Intent self harm by smoke fire/flames occ unspecif place |
| 102454 | U4B6.00 | [X]Fall jump/push frm high plce undt intn indust/constr area |
| 25859 | ZX1G.00 | Scratches self |
| 25859 | ZX1G.00 | Scratches self |
| 64331 | ZX1E.00 | Pinching self |
| 24086 | U20A.11 | [X]Self poisoning from glue solvent |
| 94377 | U2Bz.00 | [X]Int self harm by jump from high place occ unspecif place |
| 67400 | U280.00 | [X]Intent self harm by steam hot vapour/hot obj occ at home |
| 36197 | U4B..00 | [X]Falling jumping/pushed from high place undeterm intent |
| 63100 | U274.00 | [X]Intent self harm by smoke fire/flame occ street/highway |
| 51192 | ZX1S.00 | Throwing self onto floor |
| 99427 | ZX1Q.00 | Throwing self in front of train |
| 106180 | TKx0z00 | Suicide + selfinflicted inj-jump/lie before moving obj NOS |
| 101056 | TK5z.00 | Suicide and selfinflicted injury by firearms/explosives NOS |
| 99566 | TK01100 | Suicide and self inflicted injury by Barbitone |
| 96740 | U20C000 | [X]Int self poison/exposure to pesticide at home |
| 64364 | U20Bz00 | [X]Intent self poison other gas/vapour unspecif place |
| 36863 | ZX13.11 | Cuts self |
| 96730 | U200100 | [X]Intent self poison nonopioid analgesic at res institut |
| 42103 | U2z0.00 | [X]Intentional self harm by unspecif means occurrn at home |
| 10464 | ZX...00 | Self-harm |
| 65309 | TK54.00 | Suicide and selfinflicted injury by other firearm |
| 56137 | TK52.00 | Suicide and selfinflicted injury by hunting rifle |
| 94644 | U20Cy00 | [X]Int self poison pesticide other spec place |
| 90440 | U294.00 | [X]Intention self harm by sharp object occ street/highway |
| 44965 | ZX1B100 | Jumping from building |
| 56138 | U242.00 | [X]Int slf hrm rifl s'gun/lrg frarm dis sch/ins/pub adm area |
| 34156 | U2z..00 | [X]Intentional self harm by unspecified means |
| 63074 | U2A..00 | [X]Intentional self harm by blunt object |
| 68102 | U208z00 | [X]Intent self poison oth/unsp drug/medic unspecif place |
| 47283 | ZX11.11 | Bites self |
| 60684 | U2A1.00 | [X]Intent self harm by blunt object occ resident instit'n |
| 28115 | TK51.00 | Suicide and selfinflicted injury by shotgun |
| 97943 | U20Az00 | [X]Int self pois org solv,halogen hydrocarb, unspec place |
| 66634 | U207z00 | [X]Intent self poison oth autonomic drug unspecif place |
| 58594 | U20A000 | [X]Intent self pois organ solvent,halogen hydrocarb, home |
| 70946 | TKx0.00 | Suicide + selfinflicted injury-jump/lie before moving object |
| 52712 | U205000 | [X]Int self poison/exposure to narcotic drug at home |
| 72559 | ZX19100 | Punching self |
| 697 | U20..11 | [X]Deliberate drug overdose / other poisoning |
| 99011 | U204100 | [X]Intent self poison psychotropic drug at res institut |
| 65448 | TK6z.00 | Suicide and selfinflicted injury by cutting and stabbing NOS |
| 65448 | TK6z.00 | Suicide and selfinflicted injury by cutting and stabbing NOS |
| 23080 | TK3..00 | Suicide + selfinflicted injury by hang/strangulate/suffocate |
| 24461 | U27..00 | [X]Intentional self harm by smoke, fire and flames |
| 58039 | ZX1C.00 | Nipping self |
| 100635 | U2By.00 | [X]Int self harm by jump from high place occ oth specif plce |
| 100635 | U2By.00 | [X]Int self harm by jump from high place occ oth specif plce |
| 69263 | U720.00 | [X]Sequelae of intentional self-harm |
| 46154 | ZX15.00 | Drowning self |
| 53004 | U20B000 | [X]Int self poison/exposure to other gas/vapour at home |
| 9604 | TK60111 | Slashed wrists self inflicted |
| 67409 | U2y0.00 | [X]Intentionl self harm by oth specif means occurrn at home |
| 61569 | TK70.00 | Suicide+selfinflicted injury-jump from residential premises |
| 73628 | TK2z.00 | Suicide + selfinflicted poisoning by gases and vapours NOS |
| 42418 | U20A.00 | [X]Intentional self poison organ solvent,halogen hydrocarb |
| 41400 | U22..00 | [X]Intentional self harm by drowning and submersion |
| 48345 | ZX1N.00 | Stabbing self |
| 64200 | ZX1L600 | Self-mutilation of ears |
| 48934 | U200000 | [X]Int self poison/exposure to nonopioid analgesic at home |
| 46747 | U2B..00 | [X]Intentional self harm by jumping from a high place |
| 94637 | U22y.00 | [X]Intent self harm by drown/submersn occ oth specif place |
| 66118 | U204z00 | [X]Intent self poison psychotropic drug unspecif place |
| 713 | SLHz.00 | Drug and medicament poisoning NOS |
| 96728 | U200500 | [X]Intent self pois nonopioid analgesic trade/service area |
| 35879 | U208.00 | [X]Int self poison/exposure to other/unspec drug/medicament |
| 96714 | U202400 | [X]Intent self pois sedative hypnotic in street/highway |
| 12333 | ZX13.00 | Cutting self |
| 73825 | U2Cy.00 | [X]Int self harm jump/lying bef mov obje occ oth specif plce |
| 30292 | TK0..00 | Suicide + selfinflicted poisoning by solid/liquid substances |
| 42471 | U21z.00 | [X]Intent self harm by hangng strangul/suffoct unspecif plce |
| 96753 | U208y00 | [X]Int self poison oth/unsp drug/medic other spec place |
| 45709 | U25..00 | [X]Intent self harm by other/unspecified firearm discharge |
| 51362 | U20y000 | [X]Int self poison/exposure to unspecif chemical at home |
| 61177 | U2zz.00 | [X]Intent self harm by unspecif means occ at unspecif place |
| 97334 | U2z2.00 | [X]Intent self harm by unspec mean occ sch/ins/pub adm area |
| 64410 | U211.00 | [X]Intent self harm by hangng strangult/suffoct resid instit |
| 17046 | U2...00 | [X]Intentional self-harm |
| 60767 | TK3z.00 | Suicide + selfinflicted inj by hang/strangle/suffocate NOS |
| 50601 | ZX19200 | Slapping self |
| 42464 | TKy..00 | Late effects of selfinflicted injury |
| 21027 | TK...14 | Suicide and self harm |
| 71375 | TK11.00 | Suicide + selfinflicted poisoning by liquified petrol gas |
| 16907 | U30..11 | [X]Deliberate drug poisoning |
| 32267 | ZX1L.00 | Self-mutilation |
| 47501 | TKx..00 | Suicide and selfinflicted injury by other means |
| 70405 | TK1..00 | Suicide + selfinflicted poisoning by gases in domestic use |
| 67586 | U241.00 | [X]Int self harm rifl s'gun/lrg frarm disch occ resid instit |
| 33596 | TK02.00 | Suicide + selfinflicted poisoning by oth sedatives/hypnotics |
| 89429 | U20C.11 | [X]Self poisoning with weedkiller |
| 67956 | U20C.12 | [X]Self poisoning with paraquat |
| 71843 | TKxz.00 | Suicide and selfinflicted injury by other means NOS |
| 36084 | TKx2.00 | Suicide and selfinflicted injury by scald |
| 71159 | TKx4.00 | Suicide and selfinflicted injury by electrocution |
| 101906 | TKx6.00 | Suicide and selfinflicted injury by crashing of aircraft |
| 51685 | TK31.00 | Suicide + selfinflicted injury by suffocation by plastic bag |
| 52881 | U291.00 | [X]Intent self harm by sharp object occ resident instit'n |
| 68091 | U2A0.00 | [X]Intentional self harm by blunt object occurrence at home |
| 46456 | TKx1.00 | Suicide and selfinflicted injury by burns or fire |
| 92308 | ZX1R.00 | Throwing self in front of vehicle |
| 46280 | U204.11 | [X]Overdose - antidepressant |
| 60559 | U204.13 | [X]Overdose - SSRI |
| 89578 | ZX1H200 | Self-suffocation |
| 27470 | TK61.00 | Suicide and selfinflicted injury by stabbing |
| 27470 | TK61.00 | Suicide and selfinflicted injury by stabbing |
| 101971 | U2D6.00 | [X]Intent self harm crash motor vehic occ indust/constr area |
| 35868 | U2D4.00 | [X]Intent self harm by crash motor vehicl occ street/highway |
| 11122 | TK60100 | Self inflicted lacerations to wrist |
| 95794 | U250.00 | [X]Intent self harm oth/unspecif firearm disch occ at home |
| 66109 | TK01000 | Suicide and self inflicted injury by Amylobarbitone |
| 96687 | U204y00 | [X]Int self poison psychotropic drug other spec place |
| 56380 | U2D..00 | [X]Intentional self harm by crashing of motor vehicle |
| 20650 | U200z00 | [X]Intent self poison nonopioid analgesic unspecif place |
| 30074 | U290.00 | [X]Intentional self harm by sharp object occurrence at home |
| 58605 | TK7z.00 | Suicide+selfinflicted injury-jump from high place NOS |
| 53444 | U2A3.00 | [X]Intent self harm by blunt object occ sports/athlet area |
| 23753 | TK7..00 | Suicide and selfinflicted injury by jumping from high place |
| 95712 | ZX1B300 | Jumping from cliff |
| 61113 | TK72.00 | Suicide+selfinflicted injury-jump from natural sites |
| 26465 | U29..00 | [X]Intentional self harm by sharp object |
| 66621 | TK0z.00 | Suicide + selfinflicted poisoning by solid/liquid subst NOS |
| 87882 | U2y1.00 | [X]Intent self harm by oth specif means occ resid instit'n |
| 40284 | U41..00 | [X]Hanging strangulation + suffocation undetermined intent |
| 35247 | ZX1..00 | Self-injurious behaviour |
| 5242 | U2E..00 | [X]Self mutilation |
| 171 | SL...15 | Overdose of drug |
| 11708 | SL...14 | Overdose of biological substance |
| 97794 | U22z.00 | [X]Intent self harm by drown/submersn occ unspecified place |
| 21211 | U200.00 | [X]Intent self poison/exposure to nonopioid analgesic |
| 94662 | U206.00 | [X]Intent self poison/exposure to hallucinogen |
| 36398 | U204.00 | [X]Intent self poison/exposure to psychotropic drug |
| 73585 | U29y.00 | [X]Intention self harm by sharp object occ oth specif place |
| 99775 | 14K1.00 | Intentional overdose of prescription only medication |
| 96651 | U200y00 | [X]Int self poison nonopioid analgesic other spec place |
| 14853 | TK00.00 | Suicide + selfinflicted poisoning by analgesic/antipyretic |
| 10717 | U2...11 | [X]Self inflicted injury |
| 3985 | U2...13 | [X]Suicide |
| 20625 | U2...12 | [X]Injury - self-inflicted |
| 8229 | U2...15 | [X]Para-suicide |
| 17378 | U2...14 | [X]Attempted suicide |
| 53204 | U201000 | [X]Int self poison/exposure to antiepileptic at home |
| 61618 | TK2..00 | Suicide + selfinflicted poisoning by other gases and vapours |
| 56378 | U28..00 | [X]Intentional self harm by steam hot vapours / hot objects |
| 57079 | U20y.00 | [X]Intent self poison/exposure to unspecif chemical |
| 48871 | TK20.00 | Suicide + selfinflicted poisoning by motor veh exhaust gas |
| 51224 | U24..00 | [X]Intent self harm by rifle shotgun/larger firearm disch |

## Antidepressant medication prescription (BNF Chapter 4.3 Antidepressant drugs)

Antidepressant medication presciption code list

| Product code | Product name |
| --- | --- |
| 37801 | Pregabalin 225mg capsules |
| 6949 | Pregabalin 200mg capsules |
| 60543 | Pregabalin 75mg/5ml oral solution |
| 6631 | Pregabalin 150mg capsules |
| 55972 | Pregabalin 150mg/5ml oral solution |
| 819 | Pregabalin 75mg capsules |
| 790 | Pregabalin 25mg capsules |
| 6999 | Pregabalin 100mg capsules |
| 6936 | Pregabalin 50mg capsules |
| 7005 | Pregabalin 300mg capsules |
| 52547 | Lyrica 50mg capsules (Waymade Healthcare Plc) |
| 6584 | Lyrica 75mg capsules (Pfizer Ltd) |
| 16542 | Lyrica 25mg capsules (Pfizer Ltd) |
| 7209 | Lyrica 50mg capsules (Pfizer Ltd) |
| 38293 | Lyrica 225mg capsules (Pfizer Ltd) |
| 51227 | Pregabalin 20mg/ml oral solution sugar free |
| 16509 | Lyrica 150mg capsules (Pfizer Ltd) |
| 48253 | Lyrica 150mg capsules (Lexon (UK) Ltd) |
| 10189 | Lyrica 300mg capsules (Pfizer Ltd) |
| 51924 | Lyrica 20mg/ml oral solution (Pfizer Ltd) |
| 7208 | Lyrica 100mg capsules (Pfizer Ltd) |
| 7394 | Lyrica 200mg capsules (Pfizer Ltd) |
| 18290 | Marsilid 25mg Tablet (Roche Products Ltd) |
| 12207 | Isocarboxazid 10mg tablets |
| 41731 | Isocarboxazid 10mg Tablet (Cambridge Laboratories Ltd) |
| 10787 | Parnate 10mg Tablet (Goldshield Pharmaceuticals Ltd) |
| 25945 | Iproniazid 25mg |
| 41654 | Tranylcypromine 10mg tablets (AMCo) |
| 12503 | Marplan 10mg Tablet (Cambridge Laboratories Ltd) |
| 3349 | Nardil 15mg tablets (Archimedes Pharma UK Ltd) |
| 4321 | Phenelzine 15mg tablets |
| 3783 | Tranylcypromine 10mg tablets |
| 21793 | Azilect 1mg tablets (Teva UK Ltd) |
| 7040 | Rasagiline 1mg tablets |
| 53543 | Zispin SolTab 30mg orodispersible tablets (Necessity Supplies Ltd) |
| 6488 | Mirtazapine 30mg orodispersible tablets |
| 60843 | Sunveniz XL 75mg tablets (Sun Pharmaceuticals UK Ltd) |
| 40160 | Mirtazapine 30mg tablets (Actavis UK Ltd) |
| 39360 | Venlafaxine 150mg modified-release tablets |
| 54342 | Mirtazapine 15mg tablets (Medreich Plc) |
| 42600 | Vexarin XL 75mg capsules (Generics (UK) Ltd) |
| 45806 | Venlafaxine 37.5mg modified-release tablets |
| 53321 | Mirtazapine 15mg/ml oral solution sugar free (A A H Pharmaceuticals Ltd) |
| 40494 | Agomelatine 25mg tablets |
| 12221 | Pacitron 500mg Tablet (Rorer Pharmaceuticals Ltd) |
| 3951 | Fluanxol 1mg tablets (Lundbeck Ltd) |
| 40917 | ViePax 75mg tablets (Dexcel-Pharma Ltd) |
| 40815 | Tardcaps XL 75mg capsules (IXL Pharma Ltd) |
| 47966 | Mirtazapine 15mg/ml oral solution sugar free (Rosemont Pharmaceuticals Ltd) |
| 51361 | Venlafaxine 37.5mg tablets (Ranbaxy (UK) Ltd) |
| 60449 | Venlafaxine 75mg tablets (A A H Pharmaceuticals Ltd) |
| 44936 | Venlaneo XL 150mg capsules (Kent Pharmaceuticals Ltd) |
| 40054 | Venlafaxine 225mg modified-release tablets |
| 1474 | Efexor XL 75mg capsules (Pfizer Ltd) |
| 44937 | Venlaneo XL 75mg capsules (Kent Pharmaceuticals Ltd) |
| 58837 | Venlafaxine 37.5mg modified-release capsules |
| 43968 | Foraven XL 75mg capsules (Forum Products Ltd) |
| 49511 | Venlablue XL 75mg capsules (Bluefish Pharmaceuticals AB) |
| 45664 | Depefex XL 150mg capsules (Chiesi Ltd) |
| 2356 | Reboxetine 4mg tablets |
| 54792 | Mirtazapine 30mg tablets (Alliance Healthcare (Distribution) Ltd) |
| 59753 | Sunveniz XL 150mg tablets (Sun Pharmaceuticals UK Ltd) |
| 43248 | Mirtazapine 15mg orodispersible tablets (Focus Pharmaceuticals Ltd) |
| 46668 | Mirtazapine 15mg tablets (Arrow Generics Ltd) |
| 58681 | Venladex XL 75mg tablets (Dexcel-Pharma Ltd) |
| 47945 | Mirtazapine 30mg tablets (A A H Pharmaceuticals Ltd) |
| 59953 | Mirtazapine 15mg tablets (Almus Pharmaceuticals Ltd) |
| 45959 | Depefex XL 75mg capsules (Chiesi Ltd) |
| 39809 | Tifaxin XL 150mg capsules (Genus Pharmaceuticals Ltd) |
| 59954 | Mirtazapine 45mg tablets (Almus Pharmaceuticals Ltd) |
| 50592 | Fluanxol 1mg tablets (Sigma Pharmaceuticals Plc) |
| 4422 | Tryptophan 500mg tablets |
| 56662 | Venlafaxine 37.5mg tablets (A A H Pharmaceuticals Ltd) |
| 40048 | ViePax XL 75mg tablets (Dexcel-Pharma Ltd) |
| 9534 | Nefazodone Starter pack |
| 48698 | Mirtazapine 15mg orodispersible tablets sugar free |
| 16154 | Mirtazapine 15mg/ml oral solution sugar free |
| 59694 | Mirtazapine 30mg orodispersible tablets (Phoenix Healthcare Distribution Ltd) |
| 43234 | Mirtazapine 45mg orodispersible tablets (Teva UK Ltd) |
| 61547 | Mirtazapine 15mg/ml oral solution sugar free (DE Pharmaceuticals) |
| 41299 | Politid XL 75mg capsules (Actavis UK Ltd) |
| 43250 | Mirtazapine 30mg orodispersible tablets (A A H Pharmaceuticals Ltd) |
| 40049 | ViePax XL 150mg tablets (Dexcel-Pharma Ltd) |
| 301 | Venlafaxine 37.5mg tablets |
| 59923 | Venlafaxine 37.5mg tablets (Bristol Laboratories Ltd) |
| 58726 | Venladex XL 150mg tablets (Dexcel-Pharma Ltd) |
| 55501 | Venlafaxine 150mg Modified-release capsule (Hillcross Pharmaceuticals Ltd) |
| 15163 | Edronax 4mg tablets (Pfizer Ltd) |
| 43246 | Mirtazapine 15mg orodispersible tablets (Genus Pharmaceuticals Ltd) |
| 13237 | Venlafaxine 37.5mg/5ml oral suspension |
| 43242 | Mirtazapine 15mg tablets (Genus Pharmaceuticals Ltd) |
| 5611 | Optimax 500mg tablets (Merck Serono Ltd) |
| 60538 | Mirtazapine 30mg tablets (DE Pharmaceuticals) |
| 50892 | Zispin SolTab 15mg orodispersible tablets (Necessity Supplies Ltd) |
| 59593 | Fluanxol 500microgram tablets (Lexon (UK) Ltd) |
| 54747 | Optimax 500mg capsules (Merck Serono Ltd) |
| 53699 | Mirtazapine 15mg tablets (Actavis UK Ltd) |
| 55424 | Venlafaxine |
| 57751 | Tonpular XL 150mg capsules (Wockhardt UK Ltd) |
| 60370 | Zispin SolTab 15mg orodispersible tablets (Mawdsley-Brooks & Company Ltd) |
| 48199 | Ranfaxine XL 75mg capsules (Ranbaxy (UK) Ltd) |
| 2275 | Flupentixol 500microgram tablets |
| 43235 | Mirtazapine 45mg orodispersible tablets (A A H Pharmaceuticals Ltd) |
| 6481 | Mirtazapine 45mg orodispersible tablets |
| 60549 | Venlafaxine 150mg modified-release capsules (Kent Pharmaceuticals Ltd) |
| 742 | Mirtazapine 30mg tablets |
| 1222 | Venlafaxine 75mg tablets |
| 3953 | Fluanxol 500microgram tablets (Lundbeck Ltd) |
| 43241 | Mirtazapine 15mg orodispersible tablets (Aurobindo Pharma Ltd) |
| 40514 | Venaxx XL 150mg capsules (AMCo) |
| 52516 | Alventa XL 150mg capsules (Consilient Health Ltd) |
| 6854 | Mirtazapine 45mg tablets |
| 51280 | Efexor XL 150mg capsules (Waymade Healthcare Plc) |
| 6795 | Mirtazapine 15mg tablets |
| 54644 | Mirtazapine 15mg tablets (Pfizer Ltd) |
| 6421 | Mirtazapine 15mg orodispersible tablets |
| 61236 | Bonilux XL 150mg capsules (Sandoz Ltd) |
| 15268 | Zispin SolTab 45mg orodispersible tablets (Merck Sharp & Dohme Ltd) |
| 39770 | Tifaxin XL 75mg capsules (Genus Pharmaceuticals Ltd) |
| 40517 | Vexarin XL 150mg capsules (Generics (UK) Ltd) |
| 50934 | Venlafaxine 150mg/5ml oral solution |
| 4726 | Zispin 30mg tablets (Organon Laboratories Ltd) |
| 41033 | Rodomel XL 75mg capsules (Teva UK Ltd) |
| 43239 | Mirtazapine 15mg tablets (A A H Pharmaceuticals Ltd) |
| 5710 | Efexor XL 150mg capsules (Pfizer Ltd) |
| 49820 | Mirtazapine 45mg orodispersible tablets sugar free |
| 6846 | Zispin SolTab 15mg orodispersible tablets (Merck Sharp & Dohme Ltd) |
| 53648 | Mirtazapine 30mg orodispersible tablets (Actavis UK Ltd) |
| 40059 | Venlalic XL 75mg tablets (DB Ashbourne Ltd) |
| 40407 | Venlalic XL 225mg tablets (DB Ashbourne Ltd) |
| 56457 | Venlafaxine 75mg tablets (Teva UK Ltd) |
| 33337 | Mirtazapine 45mg tablets (A A H Pharmaceuticals Ltd) |
| 43257 | Mirtazapine 15mg tablets (Teva UK Ltd) |
| 623 | Efexor 37.5mg tablets (Wyeth Pharmaceuticals) |
| 40295 | Valdoxan 25mg tablets (Servier Laboratories Ltd) |
| 470 | Venlafaxine 75mg modified-release capsules |
| 43237 | Mirtazapine 15mg orodispersible tablets (Teva UK Ltd) |
| 55482 | Mirtazapine 15mg orodispersible tablets (Generics (UK) Ltd) |
| 52716 | Tonpular XL 75mg capsules (Wockhardt UK Ltd) |
| 40277 | Vensir XL 75mg capsules (Morningside Healthcare Ltd) |
| 43673 | Politid XL 150mg capsules (Actavis UK Ltd) |
| 54686 | Tryptophan 500mg capsules |
| 53326 | Venlafaxine 75mg/5ml oral solution |
| 41314 | Rodomel XL 150mg capsules (Teva UK Ltd) |
| 43203 | Venlafaxine 75mg modified-release capsules (Sandoz Ltd) |
| 59035 | Venlablue XL 37.5mg capsules (Bluefish Pharmaceuticals AB) |
| 43236 | Mirtazapine 45mg orodispersible tablets (Actavis UK Ltd) |
| 58625 | Mirtazapine 45mg tablets (Actavis UK Ltd) |
| 50081 | Venlablue XL 150mg capsules (Bluefish Pharmaceuticals AB) |
| 600 | Flupentixol 1mg tablets |
| 40764 | ViePax 37.5mg tablets (Dexcel-Pharma Ltd) |
| 20504 | Optimax wv Tablet (E. Merck) |
| 45818 | Venlalic XL 37.5mg tablets (DB Ashbourne Ltd) |
| 51699 | Venlafaxine 37.5mg/5ml oral solution |
| 52074 | Alventa XL 75mg capsules (Consilient Health Ltd) |
| 56209 | Mirtazapine 30mg tablets (Phoenix Healthcare Distribution Ltd) |
| 39359 | Venlafaxine 75mg modified-release tablets |
| 43253 | Mirtazapine 15mg orodispersible tablets (A A H Pharmaceuticals Ltd) |
| 10083 | Zispin SolTab 30mg orodispersible tablets (Merck Sharp & Dohme Ltd) |
| 40515 | Venaxx XL 75mg capsules (AMCo) |
| 60895 | Venlafaxine 37.5mg tablets (Teva UK Ltd) |
| 61856 | Mirtazapine 15mg orodispersible tablets (Consilient Health Ltd) |
| 40817 | Tardcaps XL 150mg capsules (IXL Pharma Ltd) |
| 59563 | Venlafaxine 75mg modified-release capsules (Kent Pharmaceuticals Ltd) |
| 9182 | Efexor 75mg tablets (Wyeth Pharmaceuticals) |
| 43256 | Mirtazapine 45mg orodispersible tablets (Focus Pharmaceuticals Ltd) |
| 40062 | Venlalic XL 150mg tablets (DB Ashbourne Ltd) |
| 54012 | Mirtazapine 15mg orodispersible tablets sugar free (Sandoz Ltd) |
| 58291 | Mirtazapine 15mg orodispersible tablets (Pfizer Ltd) |
| 43247 | Mirtazapine 45mg orodispersible tablets (Genus Pharmaceuticals Ltd) |
| 8844 | Tryptophan with ascorbic acid and pyridoxine powder |
| 40092 | Vensir XL 150mg capsules (Morningside Healthcare Ltd) |
| 2654 | Venlafaxine 150mg modified-release capsules |
| 43334 | Venlafaxine 150mg modified-release capsules (Sandoz Ltd) |
| 41747 | Moclobemide 150mg tablets (Teva UK Ltd) |
| 5832 | Manerix 300mg tablets (Meda Pharmaceuticals Ltd) |
| 2883 | Moclobemide 150mg tablets |
| 5187 | Moclobemide 300mg tablets |
| 9206 | Manerix 150mg tablets (Meda Pharmaceuticals Ltd) |
| 19183 | Fluoxetine 20mg capsules (A A H Pharmaceuticals Ltd) |
| 54826 | Sertraline 150mg/5ml oral suspension |
| 42803 | Fluoxetine 20mg/5ml oral solution (IVAX Pharmaceuticals UK Ltd) |
| 48220 | Prozac 20mg capsules (Lexon (UK) Ltd) |
| 252 | Prozac 20mg/5ml liquid (Eli Lilly and Company Ltd) |
| 45329 | Fluoxetine 20mg capsules (Actavis UK Ltd) |
| 43519 | Citalopram 40mg Tablet (Neo Laboratories Ltd) |
| 54081 | Sertraline 25mg/5ml oral suspension |
| 35112 | Seroxat 10mg tablets (GlaxoSmithKline UK Ltd) |
| 34603 | Citalopram 40mg tablets (Generics (UK) Ltd) |
| 815 | Cipramil 40mg/ml drops (Lundbeck Ltd) |
| 2880 | Fluvoxamine 50mg tablets |
| 34419 | Paroxetine 20mg tablets (A A H Pharmaceuticals Ltd) |
| 1712 | Cipramil 20mg tablets (Lundbeck Ltd) |
| 40892 | Paroxetine 20mg tablets (Genus Pharmaceuticals Ltd) |
| 6405 | Escitalopram 5mg tablets |
| 52824 | Citalopram 10mg tablets (PLIVA Pharma Ltd) |
| 60619 | Fluoxetine 20mg/5ml oral solution (Kent Pharmaceuticals Ltd) |
| 42387 | Sertraline 50mg tablets (Actavis UK Ltd) |
| 22 | Fluoxetine 20mg capsules |
| 59193 | Citalopram 10mg tablets (Ranbaxy (UK) Ltd) |
| 3601 | Seroxat 20mg/10ml liquid (GlaxoSmithKline UK Ltd) |
| 34356 | Citalopram 20mg tablets (A A H Pharmaceuticals Ltd) |
| 59358 | Fluoxetine 20mg capsules (Milpharm Ltd) |
| 33410 | Fluoxetine 20mg capsules (Zentiva) |
| 55537 | Seroxat 30mg tablets (Lexon (UK) Ltd) |
| 32401 | Sertraline 50mg tablets (A A H Pharmaceuticals Ltd) |
| 36893 | Fluoxetine 20mg/5ml oral solution sugar free |
| 3861 | Cipramil 10mg tablets (Lundbeck Ltd) |
| 48026 | Citalopram 20mg tablets (Almus Pharmaceuticals Ltd) |
| 53394 | Citalopram 20mg tablets (Alliance Healthcare (Distribution) Ltd) |
| 55023 | Paroxetine 20mg tablets (Medreich Plc) |
| 32848 | Citalopram 10mg tablets (Actavis UK Ltd) |
| 2290 | Fluvoxamine 100mg tablets |
| 45224 | Fluoxetine 20mg capsules (Sandoz Ltd) |
| 4297 | Dutonin 200mg tablets (Bristol-Myers Squibb Pharmaceuticals Ltd) |
| 42660 | Citalopram 10mg tablets (Almus Pharmaceuticals Ltd) |
| 40726 | Escitalopram 20mg/ml oral drops sugar free |
| 30258 | Fluoxetine 20mg/5ml oral solution (Teva UK Ltd) |
| 3391 | Dutonin 100mg tablets (Bristol-Myers Squibb Pharmaceuticals Ltd) |
| 44861 | Fluvoxamine 100mg tablets (Actavis UK Ltd) |
| 34587 | Paroxetine 30mg tablets (A A H Pharmaceuticals Ltd) |
| 55033 | Citalopram 40mg tablets (DE Pharmaceuticals) |
| 56292 | Citalopram 40mg/ml oral drops sugar free (Actavis UK Ltd) |
| 20152 | Escitalopram 10mg/ml oral drops sugar free |
| 45247 | Fluoxetine 20mg capsules (Fannin UK Ltd) |
| 57936 | Citalopram 40mg/ml oral drops sugar free (A A H Pharmaceuticals Ltd) |
| 45915 | Sertraline 50mg tablets (Almus Pharmaceuticals Ltd) |
| 34970 | Citalopram 20mg tablets (Niche Generics Ltd) |
| 527 | Paroxetine 10mg/5ml oral suspension sugar free |
| 34216 | Fluoxetine 20mg/5ml oral solution (A A H Pharmaceuticals Ltd) |
| 52354 | Citalopram 20mg tablets (DE Pharmaceuticals) |
| 34498 | Citalopram 10mg Tablet (Neo Laboratories Ltd) |
| 58476 | Citalopram 20mg tablets (Aurobindo Pharma Ltd) |
| 46926 | Citalopram 40mg tablets (Zentiva) |
| 14740 | Oxactin 20mg capsules (Discovery Pharmaceuticals Ltd) |
| 7328 | Sertraline 50mg/5ml oral suspension |
| 34456 | Fluoxetine 20mg capsules (Teva UK Ltd) |
| 4554 | Nefazodone 100mg tablets |
| 49165 | Citalopram 10mg tablets (Alliance Healthcare (Distribution) Ltd) |
| 34466 | Citalopram 40mg tablets (Sandoz Ltd) |
| 60534 | Fluoxetine 20mg dispersible tablets sugar free |
| 841 | Seroxat 20mg tablets (GlaxoSmithKline UK Ltd) |
| 45223 | Citalopram 40mg tablets (Niche Generics Ltd) |
| 58723 | Sertraline 50mg tablets (Accord Healthcare Ltd) |
| 52408 | Citalopram 10mg tablets (Kent Pharmaceuticals Ltd) |
| 34856 | Fluoxetine 60mg capsules (Generics (UK) Ltd) |
| 6360 | Cipralex 20mg tablets (Lundbeck Ltd) |
| 32899 | Paroxetine 20mg tablets (Actavis UK Ltd) |
| 34849 | Fluoxetine 20mg capsules (Tillomed Laboratories Ltd) |
| 61335 | Prozac 20mg capsules (Mawdsley-Brooks & Company Ltd) |
| 37256 | Prozep 20mg/5ml oral solution (Chemidex Pharma Ltd) |
| 2408 | Cipramil 40mg tablets (Lundbeck Ltd) |
| 57532 | Prozac 20mg capsules (Waymade Healthcare Plc) |
| 48045 | Fluvoxamine 100mg tablets (A A H Pharmaceuticals Ltd) |
| 58664 | Sertraline 50mg tablets (Generics (UK) Ltd) |
| 59650 | Citalopram 10mg tablets (Aurobindo Pharma Ltd) |
| 785 | Cipralex 5mg tablets (Lundbeck Ltd) |
| 418 | Prozac 20mg capsules (Eli Lilly and Company Ltd) |
| 34294 | Fluoxetine 20mg capsules (IVAX Pharmaceuticals UK Ltd) |
| 26016 | Citalopram 20mg tablets (Sandoz Ltd) |
| 60839 | Citalopram 40mg tablets (Almus Pharmaceuticals Ltd) |
| 1612 | Lustral 50mg tablets (Pfizer Ltd) |
| 29756 | Paxoran 20mg Tablet (Ranbaxy (UK) Ltd) |
| 50 | Paroxetine 20mg tablets |
| 4352 | Lustral 100mg tablets (Pfizer Ltd) |
| 62335 | Olena 20mg dispersible tablets (AMCo) |
| 56009 | Citalopram 20mg tablets (Arrow Generics Ltd) |
| 603 | Escitalopram 10mg tablets |
| 45316 | Fluoxetine 20mg capsules (Wockhardt UK Ltd) |
| 34966 | Citalopram 20mg tablets (Teva UK Ltd) |
| 52100 | Citalopram 10mg tablets (Arrow Generics Ltd) |
| 38890 | Fluoxetine 20mg Capsule (Milpharm Ltd) |
| 34586 | Citalopram 10mg tablets (A A H Pharmaceuticals Ltd) |
| 488 | Sertraline 50mg tablets |
| 1397 | Paroxetine 30mg tablets |
| 56355 | Citalopram 10mg tablets (Waymade Healthcare Plc) |
| 727 | Sertraline 100mg tablets |
| 59600 | Sertraline 100mg tablets (Almus Pharmaceuticals Ltd) |
| 36746 | Citalopram 40mg tablets (A A H Pharmaceuticals Ltd) |
| 54827 | Citalopram 10mg/5ml oral suspension |
| 19470 | Fluoxetine 20mg capsules (Ranbaxy (UK) Ltd) |
| 34413 | Citalopram 10mg tablets (Zentiva) |
| 43518 | Fluvoxamine 100mg tablets (IVAX Pharmaceuticals UK Ltd) |
| 4907 | Prozac 60mg capsules (Eli Lilly and Company Ltd) |
| 55488 | Sertraline 50mg tablets (Teva UK Ltd) |
| 4770 | Citalopram 40mg tablets |
| 44944 | Sertraline 100mg tablets (Teva UK Ltd) |
| 60962 | Fluoxetine 20mg capsules (Alliance Healthcare (Distribution) Ltd) |
| 34499 | Citalopram 10mg tablets (Sandoz Ltd) |
| 34871 | Citalopram 20mg tablets (Actavis UK Ltd) |
| 45286 | Citalopram 10mg tablets (Niche Generics Ltd) |
| 6218 | Escitalopram 20mg tablets |
| 476 | Citalopram 10mg tablets |
| 41062 | Cipralex 20mg/ml oral drops (Lundbeck Ltd) |
| 1575 | Seroxat 30mg tablets (GlaxoSmithKline UK Ltd) |
| 60888 | Citalopram 10mg tablets (Sigma Pharmaceuticals Plc) |
| 46977 | Citalopram 40mg tablets (Actavis UK Ltd) |
| 49519 | Sertraline 100mg/5ml oral suspension |
| 33071 | Felicium 20mg capsules (Opus Pharmaceuticals Ltd) |
| 4011 | Nefazodone 200mg tablets |
| 34722 | Citalopram 20mg Tablet (Neo Laboratories Ltd) |
| 34822 | Citalopram 20mg tablets (Zentiva) |
| 54933 | Sertraline 100mg tablets (PLIVA Pharma Ltd) |
| 53787 | Citalopram 10mg tablets (Bristol Laboratories Ltd) |
| 32546 | Paxoran 10mg Tablet (Ranbaxy (UK) Ltd) |
| 41528 | Citalopram 10mg tablets (Teva UK Ltd) |
| 67 | Citalopram 20mg tablets |
| 4075 | Fluoxetine 60mg capsules |
| 513 | Citalopram 40mg/ml oral drops sugar free |
| 60138 | Fluoxetine 20mg orodispersible tablets sugar free |
| 60568 | Citalopram 20mg tablets (Waymade Healthcare Plc) |
| 34202 | Fluoxetine 20mg capsules (Genus Pharmaceuticals Ltd) |
| 648 | Cipralex 10mg tablets (Lundbeck Ltd) |
| 29786 | Ranflutin 20mg capsules (Ranbaxy (UK) Ltd) |
| 62155 | Fluoxetine 20mg capsules (Phoenix Healthcare Distribution Ltd) |
| 26056 | Cipralex 10mg/ml oral drops (Lundbeck Ltd) |
| 33978 | Paroxetine 20mg tablets (Generics (UK) Ltd) |
| 52607 | Citalopram 20mg tablets (Bristol Laboratories Ltd) |
| 34288 | Fluoxetine 20mg capsules (Generics (UK) Ltd) |
| 34351 | Paroxetine 20mg tablets (IVAX Pharmaceuticals UK Ltd) |
| 59288 | Paroxetine 10mg tablets (Actavis UK Ltd) |
| 34436 | Citalopram 10mg tablets (Generics (UK) Ltd) |
| 2897 | Faverin 50mg tablets (Abbott Healthcare Products Ltd) |
| 45304 | Citalopram 40mg tablets (Teva UK Ltd) |
| 33720 | Citalopram 10mg tablets (IVAX Pharmaceuticals UK Ltd) |
| 35021 | Paroxetine 10mg tablets |
| 42107 | Fluoxetine 20mg capsules (Niche Generics Ltd) |
| 40165 | Paroxetine 30mg tablets (Actavis UK Ltd) |
| 2548 | Fluoxetine 20mg/5ml oral solution |
| 33779 | Prozit 20mg/5ml oral solution (Pinewood Healthcare) |
| 34415 | Citalopram 20mg tablets (Generics (UK) Ltd) |
| 12123 | Faverin 100mg tablets (Abbott Healthcare Products Ltd) |
| 55146 | Sertraline 100mg tablets (A A H Pharmaceuticals Ltd) |
| 61503 | Sertraline 100mg tablets (Actavis UK Ltd) |
| 42499 | Fluoxetine 10mg tablets |
| 74 | Dosulepin 75mg tablets |
| 4218 | Lofepramine 70mg/5ml oral suspension sugar free |
| 182 | Tryptizol 10mg/ml Injection (Merck Sharp & Dohme Ltd) |
| 41597 | Clomipramine 50mg capsules (IVAX Pharmaceuticals UK Ltd) |
| 3194 | Clomipramine 10mg capsules |
| 38274 | Clomipramine 50mg/5ml oral suspension |
| 56703 | Lofepramine 70mg tablets (Sandoz Ltd) |
| 46970 | Amitriptyline 50mg tablets (IVAX Pharmaceuticals UK Ltd) |
| 3083 | Mianserin 10mg tablets |
| 57226 | Trazodone 25mg/5ml oral suspension |
| 6054 | Dosulepin 25mg/5ml oral solution sugar free |
| 43024 | Dosulepin 100mg/5ml oral solution |
| 14534 | Limbitrol 5 Capsule (Roche Products Ltd) |
| 15632 | Dothapax 75 tablets (Ashbourne Pharmaceuticals Ltd) |
| 41709 | Trazodone 100mg capsules (Teva UK Ltd) |
| 7059 | Doxepin 75mg capsules |
| 8640 | Allegron 25mg tablets (King Pharmaceuticals Ltd) |
| 34503 | Amitriptyline 25mg tablets (IVAX Pharmaceuticals UK Ltd) |
| 41609 | Trazodone 50mg capsules (Teva UK Ltd) |
| 19186 | Dosulepin 75mg tablets (Actavis UK Ltd) |
| 34580 | Trazodone 100mg capsules (A A H Pharmaceuticals Ltd) |
| 11956 | Norval 20mg Tablet (Bencard) |
| 8928 | Surmontil 10mg tablets (Sanofi) |
| 34578 | Lofepramine 70mg tablets (IVAX Pharmaceuticals UK Ltd) |
| 34950 | Lofepramine 70mg tablets (Actavis UK Ltd) |
| 3554 | Doxepin 25mg capsules |
| 57978 | Trimipramine 25mg tablets (Waymade Healthcare Plc) |
| 7894 | Anafranil SR 75mg tablets (Novartis Pharmaceuticals UK Ltd) |
| 32457 | Butriptyline 50mg tablets |
| 29339 | Trazodone 50mg capsules (Generics (UK) Ltd) |
| 12129 | Sinequan 25mg capsules (Pfizer Ltd) |
| 1730 | Trazodone 100mg capsules |
| 29875 | Dosulepin 25mg capsules (Generics (UK) Ltd) |
| 27476 | Iprindole hc 15mg |
| 12309 | Viloxazine hcl 50mg tablets |
| 34058 | Dosulepin 75mg tablets (Teva UK Ltd) |
| 10948 | Dosulepin 75mg/5ml oral solution sugar free |
| 34421 | Trazodone 50mg capsules (Zentiva) |
| 8719 | Anafranil 25mg/5ml syrup (Novartis Pharmaceuticals UK Ltd) |
| 14398 | Asendis 50mg Tablet (Wyeth Pharmaceuticals) |
| 34672 | Lofepramine 70mg tablets (Sterwin Medicines) |
| 24700 | Prondol 15mg Tablet (Wyeth Pharmaceuticals) |
| 34470 | Trazodone 150mg tablets (Zentiva) |
| 58450 | Feprapax 70mg tablets (Ashbourne Pharmaceuticals Ltd) |
| 45318 | Clomipramine 50mg capsules (A A H Pharmaceuticals Ltd) |
| 21357 | Asendis 100mg Tablet (Wyeth Pharmaceuticals) |
| 4411 | Amoxapine 150mg tablets |
| 34641 | Dosulepin 25mg capsules (Sovereign Medical Ltd) |
| 45226 | Trimipramine 25mg tablets (A A H Pharmaceuticals Ltd) |
| 25444 | Lomont 70mg/5ml oral suspension (Rosemont Pharmaceuticals Ltd) |
| 84 | Dosulepin 25mg capsules |
| 2532 | Surmontil 25mg tablets (Sanofi) |
| 45737 | Dosulepin 25mg/5ml Oral solution (Rosemont Pharmaceuticals Ltd) |
| 42734 | Dosulepin 75mg tablets (Almus Pharmaceuticals Ltd) |
| 23426 | Dosulepin 25mg capsules (A A H Pharmaceuticals Ltd) |
| 4310 | Trimipramine 10mg tablets |
| 35493 | Sinepin 50mg capsules (Marlborough Pharmaceuticals Ltd) |
| 595 | Amitriptyline 25mg / Perphenazine 2mg tablets |
| 21157 | Thaden 75mg tablets (Opus Pharmaceuticals Ltd) |
| 19181 | Trazodone 100mg capsules (Generics (UK) Ltd) |
| 53161 | Clomipramine 50mg/5ml oral solution |
| 3842 | Doxepin 10mg capsules |
| 34046 | Lofepramine 70mg tablets (A A H Pharmaceuticals Ltd) |
| 12368 | Norval 10mg Tablet (Bencard) |
| 62620 | Clomipramine 10mg capsules (Generics (UK) Ltd) |
| 1169 | Prothiaden 25mg capsules (Teofarma) |
| 31826 | Dosulepin 75mg tablets (IVAX Pharmaceuticals UK Ltd) |
| 2039 | Trimipramine 25mg tablets |
| 61842 | Trazodone 50mg/5ml oral solution |
| 34245 | Clomipramine 25mg capsules (A A H Pharmaceuticals Ltd) |
| 59931 | Trazodone 50mg/5ml oral solution sugar free (A A H Pharmaceuticals Ltd) |
| 1940 | Dothapax 25 capsules (Ashbourne Pharmaceuticals Ltd) |
| 29857 | Trazodone 150mg tablets (Teva UK Ltd) |
| 1208 | Triptafen tablets (AMCo) |
| 3183 | Nortriptyline 10mg tablets |
| 41563 | Clomipramine 25mg capsules (IVAX Pharmaceuticals UK Ltd) |
| 21820 | Prepadine 25mg capsules (Teva UK Ltd) |
| 5073 | Doxepin 50mg capsules |
| 56229 | Lofepramine 70mg/5ml oral solution |
| 7468 | Bolvidon 10mg Tablet (Organon Laboratories Ltd) |
| 14519 | Sinequan 75mg capsules (Pfizer Ltd) |
| 18342 | Amitriptyline 25mg / Chlordiazepoxide 10mg capsules |
| 3925 | Clomipramine 50mg capsules |
| 8144 | Bolvidon 20mg Tablet (Organon Laboratories Ltd) |
| 41627 | Lofepramine 70mg Tablet (Teva UK Ltd) |
| 2093 | Gamanil 70mg tablets (Merck Serono Ltd) |
| 8585 | Bolvidon 30mg Tablet (Organon Laboratories Ltd) |
| 41628 | Clomipramine 10mg capsules (IVAX Pharmaceuticals UK Ltd) |
| 43561 | Clomipramine 10mg capsules (Teva UK Ltd) |
| 4874 | Molipaxin 50mg capsules (Zentiva) |
| 44853 | Dosulepin 25mg capsules (Kent Pharmaceuticals Ltd) |
| 34525 | Dosulepin 75mg tablets (Generics (UK) Ltd) |
| 4194 | Molipaxin 100mg capsules (Zentiva) |
| 45350 | Clomipramine 25mg capsules (Teva UK Ltd) |
| 60929 | Protriptyline 5mg tablets |
| 42228 | Trimipramine 10mg tablets (A A H Pharmaceuticals Ltd) |
| 30983 | Trazodone 150mg tablets (Generics (UK) Ltd) |
| 19779 | Amitriptyline 10mg/ml injection |
| 55137 | Trazodone 150mg/5ml oral suspension |
| 114 | Lofepramine 70mg tablets |
| 15380 | Asendis 25mg Tablet (Wyeth Pharmaceuticals) |
| 6442 | Trazodone 50mg/5ml oral solution sugar free |
| 20571 | Fluphenazine with nortriptyline 500microgramswith10mg Tablet |
| 8720 | Clomipramine 25mg/5ml oral solution |
| 27733 | Iprindole hc 30mg |
| 2320 | Prothiaden 75mg tablets (Teofarma) |
| 47363 | Mianserin 20mg Tablet (Berk Pharmaceuticals Ltd) |
| 7677 | Allegron 10mg tablets (King Pharmaceuticals Ltd) |
| 34866 | Clomipramine 10mg capsules (A A H Pharmaceuticals Ltd) |
| 4003 | Molipaxin 150mg tablets (Zentiva) |
| 30376 | Thaden 25mg capsules (Opus Pharmaceuticals Ltd) |
| 53808 | Trimipramine 10mg tablets (Phoenix Healthcare Distribution Ltd) |
| 12111 | Vivalan 50mg Tablet (AstraZeneca UK Ltd) |
| 4020 | Trazodone 150mg tablets |
| 21081 | Amitriptyline 12.5mg / Chlordiazepoxide 5mg capsules |
| 50722 | Dosulepin 25mg/5ml oral solution |
| 3657 | Anafranil 25mg capsules (Novartis Pharmaceuticals UK Ltd) |
| 11963 | Limbitrol 10 Capsule (Roche Products Ltd) |
| 8661 | Clomipramine 75mg modified-release tablets |
| 3670 | Clomipramine 25mg capsules |
| 61657 | Trazodone 75mg/5ml oral solution |
| 35258 | Sinepin 25mg capsules (Marlborough Pharmaceuticals Ltd) |
| 31824 | Dosulepin 25mg capsules (IVAX Pharmaceuticals UK Ltd) |
| 3196 | Trimipramine 50mg capsules |
| 34223 | Dosulepin 25mg capsules (Teva UK Ltd) |
| 43534 | Lofepramine 70mg/5ml Oral suspension (Rosemont Pharmaceuticals Ltd) |
| 3355 | Trazodone 50mg capsules |
| 12125 | Sinequan 50mg capsules (Pfizer Ltd) |
| 31672 | Prondol 30mg Tablet (Wyeth Pharmaceuticals) |
| 24723 | Asendis 150mg Tablet (Wyeth Pharmaceuticals) |
| 7515 | Anafranil 10mg capsules (Novartis Pharmaceuticals UK Ltd) |
| 12192 | Norval 30mg Tablet (Bencard) |
| 12227 | Butriptyline 25mg tablets |
| 53187 | Clomipramine 50mg capsules (Kent Pharmaceuticals Ltd) |
| 18932 | Evadyne 25mg Tablet (Wyeth Pharmaceuticals) |
| 32121 | Dosulepin 75mg tablets (A A H Pharmaceuticals Ltd) |
| 17319 | Amoxapine 25mg tablets |
| 2531 | Surmontil 50mg capsules (Sanofi) |
| 55138 | Trazodone 250mg/5ml oral solution |
| 60591 | Lofepramine 70mg tablets (Teva UK Ltd) |
| 6255 | Mianserin 30mg tablets |
| 41710 | Trazodone 100mg capsules (Zentiva) |
| 34745 | Dosulepin 25mg capsules (Actavis UK Ltd) |
| 57926 | Dosulepin 75mg/5ml oral solution |
| 8174 | Molipaxin 50mg/5ml oral liquid (Sanofi) |
| 40777 | Doxepin 25mg/5ml oral suspension |
| 51758 | Prothiaden 25mg capsules (Stephar (U.K.) Ltd) |
| 7693 | Anafranil 50mg capsules (Novartis Pharmaceuticals UK Ltd) |
| 34003 | Trazodone 50mg capsules (A A H Pharmaceuticals Ltd) |
| 45233 | Amitriptyline 10mg tablets (IVAX Pharmaceuticals UK Ltd) |
| 21819 | Prepadine 75mg tablets (Teva UK Ltd) |
| 34643 | Dosulepin 25mg capsules (Almus Pharmaceuticals Ltd) |
| 7910 | Tofranil 25mg tablets (Novartis Pharmaceuticals UK Ltd) |
| 33164 | Dosulepin 25mg capsules (Sandoz Ltd) |
| 10413 | Sinequan 10mg capsules (Pfizer Ltd) |
| 19168 | Dosulepin 25mg/5ml mixture |
| 33074 | Praminil 10mg Tablet (DDSA Pharmaceuticals Ltd) |
| 34872 | Imipramine 25mg Tablet (C P Pharmaceuticals Ltd) |
| 2579 | Tofranil 10mg Tablet (Novartis Pharmaceuticals UK Ltd) |
| 8726 | Tryptizol 10mg Tablet (Merck Sharp & Dohme Ltd) |
| 11187 | Protriptyline 10mg tablet |
| 17183 | Aventyl 10mg Capsule (Eli Lilly and Company Ltd) |
| 7979 | Pertofran 25mg Tablet (Novartis Pharmaceuticals UK Ltd) |
| 34197 | Amitriptyline 25mg Tablet (Berk Pharmaceuticals Ltd) |
| 7816 | Concordin 5 Tablet (Merck Sharp & Dohme Ltd) |
| 42394 | Amitriptyline 25mg Tablet (Crosspharma Ltd) |
| 27008 | Domical 50mg Tablet (Berk Pharmaceuticals Ltd) |
| 7755 | Concordin 10 Tablet (Merck Sharp & Dohme Ltd) |
| 45242 | Amitriptyline 10mg Tablet (Sussex Pharmaceutical Ltd) |
| 7751 | Tryptizol 25mg Tablet (Merck Sharp & Dohme Ltd) |
| 26213 | Domical 10mg Tablet (Berk Pharmaceuticals Ltd) |
| 22070 | Amitriptyline 10mg/5ml Oral solution (Rosemont Pharmaceuticals Ltd) |
| 7981 | Desipramine 25mg tablets |
| 12549 | Aventyl 10mg/5ml Liquid (Eli Lilly and Company Ltd) |
| 8878 | Tryptizol 10mg/5ml sugar free Oral solution (Merck Sharp and Dohme Ltd) |
| 34916 | Amitriptyline 10mg Tablet (Berk Pharmaceuticals Ltd) |
| 39145 | Nortriptyline 10mg/5ml Liquid |
| 8332 | Tryptizol 50mg Tablet (Merck Sharp & Dohme Ltd) |
| 4118 | Nortriptyline 10mg Capsule |
| 24680 | Elavil 10mg Tablet (DDSA Pharmaceuticals Ltd) |
| 32439 | Amitriptyline 25mg Tablet (Sussex Pharmaceutical Ltd) |
| 8831 | Tryptizol mr 75mg Modified-release capsule (Merck Sharp & Dohme Ltd) |
| 2525 | Amitriptyline 75mg modified-release capsules |
| 34474 | Amitriptyline 25mg Tablet (Regent Laboratories Ltd) |
| 41729 | Amitriptyline 25mg Tablet (Celltech Pharma Europe Ltd) |
| 7678 | Nortriptyline 25mg Capsule |
| 7756 | Protriptyline 5mg tablet |
| 3777 | Amitriptyline 10mg/5ml sugar free oral solution |
| 12353 | Aventyl 25mg Capsule (Eli Lilly and Company Ltd) |
| 40396 | Amitriptyline 50mg Tablet (Berk Pharmaceuticals Ltd) |
| 48065 | Amitriptyline oral solution |
| 20026 | Domical 25mg Tablet (Berk Pharmaceuticals Ltd) |
| 24134 | Amitriptyline 25mg tablets (Kent Pharmaceuticals Ltd) |
| 24152 | Amitriptyline 10mg tablets (Teva UK Ltd) |
| 42078 | Amitriptyline 25mg tablets (Almus Pharmaceuticals Ltd) |
| 33624 | Amitriptyline 50mg tablets (Teva UK Ltd) |
| 57972 | Amitriptyline 10mg tablets (Alliance Healthcare (Distribution) Ltd) |
| 60355 | Amitriptyline 25mg tablets (Phoenix Healthcare Distribution Ltd) |
| 34782 | Amitriptyline 25mg tablets (A A H Pharmaceuticals Ltd) |
| 1888 | Amitriptyline 50mg tablets |
| 6312 | Amitriptyline 25mg/5ml oral solution sugar free |
| 24141 | Amitriptyline 10mg tablets (Actavis UK Ltd) |
| 24145 | Amitriptyline 25mg tablets (Actavis UK Ltd) |
| 59161 | Amitriptyline 10mg tablets (Waymade Healthcare Plc) |
| 24147 | Amitriptyline 25mg tablets (Teva UK Ltd) |
| 34251 | Amitriptyline 50mg/5ml oral solution sugar free (Rosemont Pharmaceuticals Ltd) |
| 52867 | Amitriptyline 10mg tablets (Accord Healthcare Ltd) |
| 4690 | Amitriptyline 50mg/5ml oral solution sugar free |
| 34634 | Amitriptyline 50mg tablets (Actavis UK Ltd) |
| 46801 | Amitriptyline 10mg/5ml oral solution |
| 49 | Amitriptyline 25mg tablets |
| 61835 | Amitriptyline 10mg tablets (DE Pharmaceuticals) |
| 55491 | Amitriptyline 10mg tablets (Almus Pharmaceuticals Ltd) |
| 57107 | Amitriptyline 10mg tablets (Phoenix Healthcare Distribution Ltd) |
| 59820 | Amitriptyline 50mg/5ml oral solution sugar free (Wockhardt UK Ltd) |
| 60410 | Amitriptyline 25mg/5ml oral solution sugar free (Wockhardt UK Ltd) |
| 83 | Amitriptyline 10mg tablets |
| 34401 | Amitriptyline 10mg tablets (Wockhardt UK Ltd) |
| 54877 | Amitriptyline 25mg tablets (Accord Healthcare Ltd) |
| 34274 | Amitriptyline 50mg tablets (A A H Pharmaceuticals Ltd) |
| 46818 | Amitriptyline 10mg/5ml oral suspension |
| 34182 | Amitriptyline 50mg tablets (Kent Pharmaceuticals Ltd) |
| 55139 | Amitriptyline 25mg tablets (Alliance Healthcare (Distribution) Ltd) |
| 34731 | Amitriptyline 10mg tablets (Kent Pharmaceuticals Ltd) |
| 34129 | Amitriptyline 25mg tablets (Wockhardt UK Ltd) |
| 34224 | Amitriptyline 25mg/5ml oral solution sugar free (Rosemont Pharmaceuticals Ltd) |
| 34107 | Amitriptyline 50mg tablets (Wockhardt UK Ltd) |
| 33090 | Amitriptyline 10mg tablets (A A H Pharmaceuticals Ltd) |
| 1310 | Imipramine 10mg tablets |
| 1809 | Imipramine 25mg tablets |
| 3903 | Nortriptyline 25mg tablets |
| 34355 | Imipramine 25mg tablets (Actavis UK Ltd) |
| 34222 | Imipramine 10mg tablets (Actavis UK Ltd) |
| 55970 | Nortriptyline 10mg tablets (King Pharmaceuticals Ltd) |
| 34813 | Imipramine 25mg tablets (A A H Pharmaceuticals Ltd) |
| 41408 | Imipramine 25mg tablets (Teva UK Ltd) |
| 32863 | Imipramine 10mg tablets (Teva UK Ltd) |
| 41681 | Imipramine 10mg tablets (A A H Pharmaceuticals Ltd) |
| 56501 | Tofranil 25mg tablets (Lexon (UK) Ltd) |
| 48216 | Nortriptyline 25mg tablets (A A H Pharmaceuticals Ltd) |
| 42247 | Imipramine 25mg/5ml oral solution sugar free |

## Antipsychotic medication prescription (BNF Chapter 4.2 Drugs used in psychoses and related disorders)

Anti-psychotic medication presciption code list

| Product code | Product name |
| --- | --- |
| 8445 | Stelabid Tablet (GlaxoSmithKline Consumer Healthcare) |
| 10514 | Fluphenazine decanoate 100mg/ml Injection |
| 14966 | Flupentixol 20mg/1ml solution for injection ampoules |
| 57170 | Psytixol 100mg/1ml solution for injection ampoules (Generics (UK) Ltd) |
| 33780 | Modecate 25mg/1ml solution for injection ampoules (Sanofi) |
| 59816 | Psytixol 50mg/0.5ml solution for injection ampoules (Generics (UK) Ltd) |
| 12340 | Piportil 50mg/ml Depot injection (JHC Healthcare Ltd) |
| 2276 | Flupentixol 40mg/2ml solution for injection ampoules |
| 17190 | Fluphenazine enanthate 25mg/ml Injection |
| 2136 | Depixol 20mg/ml Injection (Lundbeck Ltd) |
| 8044 | Redeptin 2mg/ml Injection (Janssen-Cilag Ltd) |
| 10944 | Pipotiazine palmitate 50mg/ml depot injection |
| 60782 | Psytixol 20mg/1ml solution for injection ampoules (Generics (UK) Ltd) |
| 2155 | Depixol -conc 100mg/ml Injection (Lundbeck Ltd) |
| 19283 | Depixol 20mg/1ml solution for injection ampoules (Lundbeck Ltd) |
| 41970 | Fluphenazine decanoate 25mg/1ml solution for injection ampoules (Hospira UK Ltd) |
| 35445 | Modecate 50mg/2ml solution for injection ampoules (Sanofi) |
| 35235 | Piportil Depot 50mg/1ml solution for injection ampoules (Sanofi) |
| 46436 | Xeplion 75mg/0.75ml suspension for injection pre-filled syringes (Janssen-Cilag Ltd) |
| 18155 | Flupentixol 50mg/0.5ml solution for injection ampoules |
| 9022 | Fluphenazine decanoate 25mg/ml Injection |
| 14130 | Depixol Low Volume 200mg/1ml solution for injection ampoules (Lundbeck Ltd) |
| 35530 | Fluphenazine decanoate 12.5mg/0.5ml solution for injection ampoules |
| 18175 | Flupentixol 100mg/1ml solution for injection ampoules |
| 35065 | Fluphenazine decanoate 25mg/1ml solution for injection ampoules |
| 35723 | Fluphenazine decanoate 50mg/2ml solution for injection ampoules |
| 35684 | Pipotiazine 50mg/1ml solution for injection ampoules |
| 10827 | Fluspirilene 2mg/ml Injection |
| 35122 | Modecate 12.5mg/0.5ml solution for injection ampoules (Sanofi) |
| 36394 | Pipotiazine 100mg/2ml solution for injection ampoules |
| 14889 | Depixol Conc 100mg/1ml solution for injection ampoules (Lundbeck Ltd) |
| 57762 | Psytixol 40mg/2ml solution for injection ampoules (Generics (UK) Ltd) |
| 46351 | Paliperidone 150mg/1.5ml suspension for injection pre-filled syringes |
| 46447 | Paliperidone 100mg/1ml suspension for injection pre-filled syringes |
| 25835 | Moditen enanthate 25mg/ml Injection (Sanofi-Synthelabo Ltd) |
| 35455 | Modecate Concentrate 100mg/1ml solution for injection ampoules (Sanofi) |
| 46224 | Paliperidone 50mg/0.5ml suspension for injection pre-filled syringes |
| 46556 | Paliperidone 75mg/0.75ml suspension for injection pre-filled syringes |
| 3926 | Modecate 25mg/ml Injection (Sanofi-Synthelabo Ltd) |
| 18197 | Depixol Conc 50mg/0.5ml solution for injection ampoules (Lundbeck Ltd) |
| 35487 | Modecate Concentrate 50mg/0.5ml solution for injection ampoules (Sanofi) |
| 14839 | Flupentixol 200mg/1ml solution for injection ampoules |
| 35488 | Piportil Depot 100mg/2ml solution for injection ampoules (Sanofi) |
| 1733 | Flupentixol decanoate 20mg/ml Injection |
| 47162 | Xeplion 50mg/0.5ml suspension for injection pre-filled syringes (Janssen-Cilag Ltd) |
| 41971 | Fluphenazine decanoate 25mg/ml Injection (Antigen Pharmaceuticals) |
| 8712 | Flupentixol decanoate 100mg/ml Injection |
| 35176 | Fluphenazine decanoate 100mg/1ml solution for injection ampoules |
| 46435 | Xeplion 150mg/1.5ml suspension for injection pre-filled syringes (Janssen-Cilag Ltd) |
| 46434 | Xeplion 100mg/1ml suspension for injection pre-filled syringes (Janssen-Cilag Ltd) |
| 12128 | Modecate concentrate 100mg/ml Injection (Sanofi-Synthelabo Ltd) |
| 2156 | Depixol 40mg/2ml solution for injection ampoules (Lundbeck Ltd) |
| 35391 | Fluphenazine decanoate 50mg/0.5ml solution for injection ampoules |
| 14576 | Zuclopenthixol acetate 50mg/ml oily injection |
| 15598 | Thioridazine 100mg/5ml sugar free Oral solution |
| 17050 | Sertindole 20mg tablets |
| 16223 | Roxiam 300mg Capsule (AstraZeneca UK Ltd) |
| 23034 | Remoxipride 75mg capsule |
| 33493 | Sparine 100mg Tablet (Wyeth Pharmaceuticals) |
| 40782 | Levomepromazine 6mg Tablet |
| 1218 | Thioridazine 25mg tablets |
| 19900 | Sertindole 16mg tablets |
| 5762 | Clopixol acuphase 50mg/ml Oily injection (Lundbeck Ltd) |
| 16103 | Olanzapine 20mg Orodispersible tablet |
| 21047 | Triperidol 0.5mg Tablet (Lagap) |
| 12193 | Sparine 25mg Tablet (Wyeth Pharmaceuticals) |
| 27148 | Orap 10mg Tablet (Janssen-Cilag Ltd) |
| 6023 | Olanzapine 10mg Orodispersible tablet |
| 10780 | Promazine 50mg/5ml oral solution |
| 30088 | Zoleptil 25 tablets (Movianto UK Ltd) |
| 28759 | Zotepine 100mg tablets |
| 1192 | Thioridazine 10mg tablets |
| 7833 | Neulactil 2.5mg Tablet (JHC Healthcare Ltd) |
| 55870 | Quetiapine oral liquid |
| 27211 | Integrin 40mg Tablet (Sanofi-Synthelabo Ltd) |
| 25336 | Zotepine 50mg tablets |
| 42816 | Thioridazine 50mg/5ml Oral solution (Rosemont Pharmaceuticals Ltd) |
| 12445 | Remoxipride 300mg capsule |
| 16998 | Sertindole 12mg tablets |
| 10107 | Quetiapine Starter Pack |
| 25966 | Serdolect 4mg tablets (Lundbeck Ltd) |
| 12666 | Sertindole 4mg tablets |
| 41995 | Promazine 50mg/ml Injection (Genus Pharmaceuticals Ltd) |
| 21339 | Veractil 25mg Tablet (Rhone-Poulenc Rorer Ltd) |
| 8031 | Neulactil 10mg Tablet (JHC Healthcare Ltd) |
| 43654 | Promazine 50mg/ml injection |
| 3021 | Thioridazine 100mg tablets |
| 1314 | Thioridazine 50mg tablets |
| 15472 | Pericyazine 25mg tablet |
| 9515 | Zoleptil 50 tablets (Movianto UK Ltd) |
| 21027 | Triperidol 1mg Tablet (Lagap) |
| 6412 | Olanzapine 5mg Orodispersible tablet |
| 3226 | Sparine 50mg Tablet (Wyeth Pharmaceuticals) |
| 30111 | Chlorprothixene 50mg tablets |
| 62387 | Lurasidone 37mg tablets |
| 13311 | Sparine 50mg/ml Injection (Wyeth Pharmaceuticals) |
| 15395 | Promazine 12.5mg/5ml oral solution |
| 15161 | Promazine 50mg/ml injection |
| 3605 | Thioridazine 25mg/5ml oral solution |
| 55890 | Promazine 50mg/5ml Liquid (Rosemont Pharmaceuticals Ltd) |
| 21744 | Anquil 250microgram Tablet (Concord Pharmaceuticals Ltd) |
| 45860 | Thioridazine 100mg Tablet (IVAX Pharmaceuticals UK Ltd) |
| 46945 | Promazine 25mg Tablet (Biorex Laboratories Ltd) |
| 31063 | Serdolect 12mg tablets (Lundbeck Ltd) |
| 2801 | Thioridazine 10mg/5ml Oral solution |
| 28147 | Taractan 15mg Tablet (Roche Products Ltd) |
| 21064 | Neulactil 25mg Tablet (JHC Healthcare Ltd) |
| 3227 | Sparine 50mg/5ml Liquid (Wyeth Pharmaceuticals) |
| 23162 | Serdolect 16mg tablets (Lundbeck Ltd) |
| 17399 | Thioridazine 50mg/5ml Oral solution |
| 6838 | Olanzapine 15mg Orodispersible tablet |
| 23659 | Trifluperidol 0.5mg Tablet |
| 14610 | Promazine 50mg/5ml oral solution sugar free |
| 10405 | Thioridazine 25mg/5ml sugar free Oral solution |
| 17504 | Zotepine 25mg tablets |
| 8637 | Pimozide 10mg tablet |
| 19016 | Roxiam 150mg Capsule (AstraZeneca UK Ltd) |
| 34905 | Thioridazine 25mg Tablet (IVAX Pharmaceuticals UK Ltd) |
| 8881 | Remoxipride 150mg capsule |
| 35787 | Thioridazine 50mg Tablet (IVAX Pharmaceuticals UK Ltd) |
| 28231 | Levinan 6mg Tablet (Link Pharmaceuticals Ltd) |
| 3197 | Promazine 100mg tablet |
| 62517 | Lurasidone 74mg tablets |
| 5212 | Fluphenazine 1mg tablets |
| 48077 | Roxiam ir 75mg Capsule (AstraZeneca UK Ltd) |
| 47361 | Thioridazine 10mg/5ml Oral solution (Rosemont Pharmaceuticals Ltd) |
| 8921 | Integrin 10mg Capsule (Sanofi-Synthelabo Ltd) |
| 62463 | Latuda 37mg tablets (Sunovion Pharmaceuticals Europe Ltd) |
| 5597 | Moditen 1mg tablets (Sanofi) |
| 22814 | Trifluperidol 1mg Tablet |
| 8771 | Largactil 10mg Tablet (Hawgreen Ltd) |
| 10434 | Largactil 25mg/5ml Oral solution (Hawgreen Ltd) |
| 28679 | Dozic 2mg/ml Oral solution (Rosemont Pharmaceuticals Ltd) |
| 3772 | Largactil 50mg Tablet (Hawgreen Ltd) |
| 11213 | Haloperidol 2mg/5ml sugar free Oral solution |
| 43020 | Haloperidol Oral solution |
| 7479 | Stelazine 1mg/ml Injection (Goldshield Pharmaceuticals Ltd) |
| 34039 | Haloperidol 1mg/ml Liquid (Rosemont Pharmaceuticals Ltd) |
| 13105 | Haloperidol 2mg/ml Oral solution |
| 8506 | Chlorpromazine 100mg suppository |
| 5192 | Haloperidol 1mg/5ml sugar free Oral solution |
| 4401 | Prochlorperazine maleate 10mg modified release capsule |
| 2814 | Largactil 25mg Tablet (Hawgreen Ltd) |
| 42000 | Haloperidol 2mg/ml Liquid (Rosemont Pharmaceuticals Ltd) |
| 55871 | Haloperidol 2mg/ml Liquid (Hillcross Pharmaceuticals Ltd) |
| 10435 | Haloperidol 10mg/ml Oral solution |
| 9975 | Haloperidol 1mg/ml sugar free Oral solution |
| 25909 | Perphenazine 4mg/5ml Oral solution sugar free |
| 8689 | Vertigon spansule 10 10mg Spansule (GlaxoSmithKline Consumer Healthcare) |
| 19002 | Largactil 100mg Suppository (Rhone-Poulenc Rorer Ltd) |
| 15438 | Prochlorperazine maleate 15mg modified release capsul |
| 14987 | Perphenazine 2mg/5ml oral solution sugar free |
| 34272 | Haloperidol 5mg/ml Injection (Antigen Pharmaceuticals) |
| 18668 | Trifluoperazine 10mg/ml concentrate |
| 8537 | Trifluoperazine 1mg/ml Injection |
| 17227 | Chloractil 25mg Tablet (DDSA Pharmaceuticals Ltd) |
| 3233 | Haloperidol 2mg/ml sugar free Liquid |
| 54458 | Prochlorperazine 5mg Tablet (Teva UK Ltd) |
| 17087 | Perphenazine 5mg/ml injection |
| 25653 | Chloractil 50mg Tablet (DDSA Pharmaceuticals Ltd) |
| 500 | Buccastem 3mg Tablet (Reckitt Benckiser Healthcare (UK) Ltd) |
| 24494 | Haldol 10mg/ml Liquid (Janssen-Cilag Ltd) |
| 37871 | Chlorpromazine 25mg/5ml Oral solution (Rosemont Pharmaceuticals Ltd) |
| 7493 | Largactil 100mg Tablet (Hawgreen Ltd) |
| 228 | Fentazin 5mg/ml Injection (Goldshield Pharmaceuticals Ltd) |
| 37705 | Chlorpromazine 100mg/5ml suspension |
| 2620 | Haloperidol 1mg/ml Oral solution |
| 7593 | Stemetil 5mg/5ml Oral solution (Castlemead Healthcare Ltd) |
| 32051 | Haloperidol 5mg Tablet (Generics (UK) Ltd) |
| 17849 | Vertigon spansule 15 15mg Spansule (GlaxoSmithKline Consumer Healthcare) |
| 3246 | Stemetil 12.5mg/ml Injection (Castlemead Healthcare Ltd) |
| 41645 | Chlorpromazine 25mg/ml Injection (Antigen Pharmaceuticals) |
| 28862 | Chloractil 100mg Tablet (DDSA Pharmaceuticals Ltd) |
| 8311 | Chlorpromazine 25mg/ml injection |
| 18289 | Stelazine 10mg/ml Concentrate (Goldshield Pharmaceuticals Ltd) |
| 17379 | Haloperidol 1.5mg/5ml sugar free Oral solution |
| 15418 | Largactil forte 100mg/5ml Oral suspension (Hawgreen Ltd) |
| 512 | Stemetil 5mg Tablet (Castlemead Healthcare Ltd) |
| 42807 | Haloperidol 500microgram Tablet (Lagap) |
| 4234 | Haloperidol 5mg/ml Injection |
| 41546 | Haloperidol 1mg/ml Liquid (Hillcross Pharmaceuticals Ltd) |
| 5510 | Prochlorperazine mesilate 12.5mg/ml injection |
| 6523 | Haldol 5mg/ml Injection (Janssen-Cilag Ltd) |
| 3775 | Zuclopenthixol decanoate 200mg/ml oily injection |
| 3774 | Clopixol 200mg/ml Oily injection (Lundbeck Ltd) |
| 24053 | Sulparex 200mg Tablet (E R Squibb and Sons Ltd) |
| 3356 | Parstelin Tablet (GlaxoSmithKline Consumer Healthcare) |
| 3955 | Tranylcypromine with trifluoperazine Tablet |
| 24890 | Trifluoperazine with tranylcypromine 1mg + 10mg Tablet |
| 55620 | Flupentixol Liquid |
| 1453 | Triptafen m 2mg+10mg Tablet (Goldshield Pharmaceuticals Ltd) |
| 16323 | Perphenazine 2mg with Amitriptyline 10mg tablet |
| 6894 | Perphenazine 2mg with Amitriptyline 25mg tablet |
| 12402 | Camcolit 250 tablets (Essential Pharma Ltd) |
| 12403 | Camcolit 400 modified-release tablets (Essential Pharma Ltd) |
| 872 | Priadel 400mg modified-release tablets (Sanofi) |
| 22018 | Li-Liquid 509mg/5ml oral solution (Rosemont Pharmaceuticals Ltd) |
| 51401 | Priadel 400mg modified-release tablets (Necessity Supplies Ltd) |
| 56435 | Lithium carbonate 200mg/5ml oral suspension |
| 3359 | Priadel 200mg modified-release tablets (Sanofi) |
| 25344 | Lithium carbonate 400mg Modified-release tablet (Approved Prescription Services Ltd) |
| 47167 | Asenapine 10mg sublingual tablets sugar free |
| 8827 | Lithium carbonate 300mg Modified-release tablet |
| 53459 | Priadel 400mg modified-release tablets (DE Pharmaceuticals) |
| 15388 | Phasal 300mg Tablet (Lagap) |
| 47280 | Asenapine 5mg sublingual tablets sugar free |
| 10937 | Lithium citrate 509mg/5ml oral solution |
| 10809 | Priadel 520mg/5ml liquid (Sanofi) |
| 12648 | Lithium citrate 1.018g/5ml oral solution |
| 11491 | Lithium citrate 520mg/5ml oral solution sugar free |
| 1447 | Lithium carbonate 400mg modified-release tablets |
| 56427 | Lithium citrate 509mg/5ml oral solution (Cubic Pharmaceuticals Ltd) |
| 59345 | Sycrest 5mg sublingual tablets (Lundbeck Ltd) |
| 14954 | Lithium carbonate 450mg modified-release tablets |
| 3352 | Liskonum 450mg modified-release tablets (GlaxoSmithKline UK Ltd) |
| 25345 | Li-Liquid 1.018g/5ml oral solution (Rosemont Pharmaceuticals Ltd) |
| 8041 | Lithium carbonate 250mg tablets |
| 760 | Lithium carbonate 200mg modified-release tablets |
| 46972 | Carbamazepine 200mg Tablet (IVAX Pharmaceuticals UK Ltd) |
| 46888 | Carbamazepine 200mg Modified-release tablet (Generics (UK) Ltd) |
| 32900 | Carbamazepine sr 200mg Tablet (IVAX Pharmaceuticals UK Ltd) |
| 41726 | Carbamazepine 100mg Tablet (IVAX Pharmaceuticals UK Ltd) |
| 53188 | Carbamazepine 500mg/5ml Oral suspension (Martindale Pharmaceuticals Ltd) |
| 2823 | Tegretol retard 200mg Modified-release tablet (Novartis Pharmaceuticals UK Ltd) |
| 47294 | Carbamazepine sr 400mg Tablet (IVAX Pharmaceuticals UK Ltd) |
| 2824 | Tegretol retard 400mg Modified-release tablet (Novartis Pharmaceuticals UK Ltd) |
| 34958 | Carbamazepine 100mg Tablet (Berk Pharmaceuticals Ltd) |
| 40403 | Carbamazepine 400mg Modified-release tablet (Lagap) |
| 43451 | Carbamazepine 400mg Modified-release tablet (Generics (UK) Ltd) |
| 37584 | Episenta 500mg modified-release granules sachets (Desitin Pharma Ltd) |
| 53211 | Valproic acid 500mg/5ml oral solution |
| 37800 | Carbamazepine 200mg Modified-release tablet (Lagap) |
| 36634 | Episenta 1000mg modified-release granules sachets (Desitin Pharma Ltd) |
| 30509 | Timonil retard 400mg Modified-release tablet (C P Pharmaceuticals Ltd) |
| 7064 | Depakote 250mg gastro-resistant tablets (Sanofi) |
| 6305 | Valproic acid 500mg gastro-resistant tablets |
| 9759 | Depakote 500mg gastro-resistant tablets (Sanofi) |
| 5848 | Valproic acid 250mg gastro-resistant tablets |
| 8519 | Chlorpromazine 100mg/5ml oral solution |
| 4232 | Nozinan 25mg tablets (Sanofi) |
| 34810 | Sulpiride 200mg tablets (Wockhardt UK Ltd) |
| 47013 | Haloperidol 1mg/5ml oral suspension |
| 31537 | Zuclopenthixol acetate 100mg/2ml solution for injection ampoules |
| 38540 | Haldol 5mg/1ml solution for injection ampoules (Janssen-Cilag Ltd) |
| 31171 | Chlorpromazine 50mg tablets (A A H Pharmaceuticals Ltd) |
| 5821 | Pimozide 4mg tablets |
| 56862 | Chlorpromazine 25mg/5ml syrup (Rosemont Pharmaceuticals Ltd) |
| 13600 | Zuclopenthixol 25mg tablets |
| 9686 | Zuclopenthixol 10mg tablets |
| 23678 | Haldol 10mg tablets (Janssen-Cilag Ltd) |
| 31172 | Chlorpromazine 50mg tablets (Teva UK Ltd) |
| 2621 | Haloperidol 5mg tablets |
| 4434 | Chlorpromazine 50mg/5ml oral solution |
| 1319 | Clopixol 10mg tablets (Lundbeck Ltd) |
| 44186 | Chlorpromazine 25mg/5ml oral solution (A A H Pharmaceuticals Ltd) |
| 58702 | Largactil 100mg tablets (Sanofi) |
| 46960 | Chlorpromazine 100mg tablets (IVAX Pharmaceuticals UK Ltd) |
| 7390 | Levomepromazine 6mg tablets |
| 13902 | Neulactil Forte syrup (Sanofi) |
| 42895 | Haloperidol 5mg tablets (Teva UK Ltd) |
| 3952 | Chlorpromazine 25mg/5ml oral solution |
| 5014 | Levomepromazine 25mg tablets |
| 13484 | Serenace 10mg tablets (Teva UK Ltd) |
| 8153 | Serenace 2mg/ml liquid (Teva UK Ltd) |
| 36771 | Haloperidol 250micrograms/5ml oral suspension |
| 12224 | Zuclopenthixol decanoate 500mg/1ml solution for injection ampoules |
| 18352 | Sulpitil 200mg tablets (Pfizer Ltd) |
| 58703 | Largactil 50mg tablets (Sanofi) |
| 588 | Chlorpromazine 25mg tablets |
| 53951 | Levomepromazine 6.25mg/5ml oral solution |
| 31796 | Benquil 250microgram tablets (Concord Pharmaceuticals Ltd) |
| 7514 | Largactil 50mg/2ml solution for injection ampoules (Sanofi) |
| 43520 | Haloperidol 1.5mg tablets (Teva UK Ltd) |
| 9190 | Chlorpromazine 25mg/5ml oral solution sugar free |
| 8979 | Serenace 1.5mg tablets (Teva UK Ltd) |
| 34668 | Chlorpromazine 25mg tablets (Teva UK Ltd) |
| 45880 | Haloperidol 5mg/5ml oral solution sugar free |
| 12073 | Clopixol Conc 500mg/1ml solution for injection ampoules (Lundbeck Ltd) |
| 60942 | Haloperidol 5mg/5ml oral solution |
| 18181 | Sulpor 200mg/5ml oral solution (Rosemont Pharmaceuticals Ltd) |
| 57550 | Largactil 25mg/5ml syrup (Sanofi) |
| 58492 | Chlorpromazine 100mg tablets (Waymade Healthcare Plc) |
| 2540 | Benperidol 250microgram tablets |
| 2154 | Chlorpromazine 100mg tablets |
| 60719 | Levomepromazine 5mg/5ml oral solution |
| 34903 | Haloperidol 5mg tablets (IVAX Pharmaceuticals UK Ltd) |
| 43522 | Sulpiride 200mg tablets (Teva UK Ltd) |
| 17634 | Promazine 50mg/5ml oral solution |
| 43423 | Sulpiride 200mg tablets (A A H Pharmaceuticals Ltd) |
| 12921 | Haldol 2mg/ml oral solution (Janssen-Cilag Ltd) |
| 840 | Fentazin 2mg tablets (AMCo) |
| 4442 | Nozinan 25mg/1ml solution for injection ampoules (Sanofi) |
| 34693 | Chlorpromazine 25mg tablets (Thornton & Ross Ltd) |
| 60450 | Promazine 25mg tablets (A A H Pharmaceuticals Ltd) |
| 49207 | Haloperidol 2mg/5ml oral solution |
| 22606 | Chlorpromazine 25mg/1ml solution for injection ampoules |
| 5712 | Depixol 3mg tablets (Lundbeck Ltd) |
| 2419 | Haloperidol 500microgram capsules |
| 61083 | Levomepromazine 25mg/1ml solution for injection ampoules (A A H Pharmaceuticals Ltd) |
| 47365 | Anquil 250microgram tablets (Archimedes Pharma UK Ltd) |
| 35929 | Chlorpromazine 50mg/2ml solution for injection ampoules |
| 5707 | Flupentixol 3mg tablets |
| 60250 | Levomepromazine 3mg/5ml oral solution |
| 2972 | Promazine 25mg tablets |
| 31175 | Chlorpromazine 25mg tablets (A A H Pharmaceuticals Ltd) |
| 9247 | Sulpiride 400mg tablets |
| 28355 | Zuclopenthixol decanoate 200mg/1ml solution for injection ampoules |
| 40390 | Promazine 25mg/5ml syrup (Rosemont Pharmaceuticals Ltd) |
| 36101 | Clopixol Acuphase 50mg/1ml solution for injection ampoules (Lundbeck Ltd) |
| 52846 | Nozinan 25mg/1ml solution for injection ampoules (Lexon (UK) Ltd) |
| 3348 | Chlorpromazine 50mg tablets |
| 7834 | Pericyazine 2.5mg tablets |
| 8903 | Sulpiride 200mg/5ml oral solution sugar free |
| 6443 | Promazine 25mg/5ml oral solution |
| 55011 | Largactil 25mg tablets (Sanofi) |
| 34736 | Chlorpromazine 100mg tablets (Teva UK Ltd) |
| 38262 | Haloperidol 5mg/1ml solution for injection ampoules |
| 40881 | Neulactil 10mg tablets (Sanofi) |
| 15047 | Orap 4mg tablets (Janssen-Cilag Ltd) |
| 7919 | Fentazin 4mg tablets (AMCo) |
| 52050 | Haloperidol 1.5mg/5ml oral suspension |
| 34630 | Chlorpromazine 50mg tablets (Thornton & Ross Ltd) |
| 8032 | Pericyazine 10mg tablets |
| 31184 | Chlorpromazine 25mg tablets (IVAX Pharmaceuticals UK Ltd) |
| 13483 | Serenace 20mg tablets (Teva UK Ltd) |
| 22660 | Haldol 5mg tablets (Janssen-Cilag Ltd) |
| 12387 | Haloperidol 20mg tablets |
| 34339 | Haloperidol 1.5mg tablets (A A H Pharmaceuticals Ltd) |
| 47149 | Haloperidol 1mg/5ml oral solution |
| 24270 | Zuclopenthixol acetate 50mg/1ml solution for injection ampoules |
| 53649 | Haloperidol 2mg/5ml oral suspension |
| 12707 | Zuclopenthixol 2mg tablets |
| 329 | Haloperidol 1.5mg tablets |
| 12195 | Pericyazine 10mg/5ml oral solution |
| 41675 | Sulpiride 200mg tablets (IVAX Pharmaceuticals UK Ltd) |
| 10666 | Dolmatil 200mg tablets (Sanofi) |
| 32838 | Haloperidol 1.5mg tablets (IVAX Pharmaceuticals UK Ltd) |
| 3228 | Promazine 50mg tablets |
| 22049 | Clopixol 200mg/1ml solution for injection ampoules (Lundbeck Ltd) |
| 5545 | Serenace 500microgram capsules (Teva UK Ltd) |
| 49606 | Levinan 6mg tablets (Archimedes Pharma UK Ltd) |
| 41732 | Promazine 50mg tablets (Teva UK Ltd) |
| 24069 | Dolmatil 400mg tablets (Sanofi) |
| 59938 | Levomepromazine 25mg/1ml solution for injection ampoules (Wockhardt UK Ltd) |
| 2135 | Sulpiride 200mg tablets |
| 9347 | Clopixol 25mg tablets (Lundbeck Ltd) |
| 6064 | Levomepromazine 25mg/1ml solution for injection ampoules |
| 31538 | Clopixol Acuphase 100mg/2ml solution for injection ampoules (Lundbeck Ltd) |
| 38089 | Promazine 50mg/5ml syrup (Rosemont Pharmaceuticals Ltd) |
| 7436 | Serenace 5mg/1ml solution for injection ampoules (IVAX Pharmaceuticals UK Ltd) |
| 475 | Haloperidol 10mg tablets |
| 13368 | Clopixol 2mg tablets (Lundbeck Ltd) |
| 55848 | Haloperidol 5mg/1ml solution for injection ampoules (AMCo) |
| 47808 | Haloperidol 10mg/5ml oral solution sugar free (A A H Pharmaceuticals Ltd) |
| 45810 | Haloperidol 10mg/5ml oral solution sugar free |
| 61153 | Chlorpromazine 25mg tablets (Phoenix Healthcare Distribution Ltd) |
| 45281 | Chlorpromazine 100mg/5ml oral solution (Rosemont Pharmaceuticals Ltd) |
| 13338 | Serenace 5mg tablets (Teva UK Ltd) |
| 6134 | Dozic 5mg/5ml oral solution (Rosemont Pharmaceuticals Ltd) |
| 39830 | Neulactil 2.5mg tablets (Sanofi) |
| 10565 | Haloperidol decanoate 50mg/1ml solution for injection ampoules |
| 15814 | Haloperidol decanoate 100mg/1ml solution for injection ampoules |
| 12386 | Haldol decanoate 100mg/1ml solution for injection ampoules (Janssen-Cilag Ltd) |
| 2094 | Haldol decanoate 50mg/1ml solution for injection ampoules (Janssen-Cilag Ltd) |
| 43420 | Prochlorperazine 5mg tablets (Dr Reddy's Laboratories (UK) Ltd) |
| 57605 | Stelazine 1mg tablets (Lexon (UK) Ltd) |
| 51551 | Stemetil 5mg tablets (Mawdsley-Brooks & Company Ltd) |
| 32064 | Prochlorperazine 5mg tablets (Actavis UK Ltd) |
| 32122 | Prochlorperazine 12.5mg/1ml solution for injection ampoules (AMCo) |
| 55382 | Trifluoperazine 1mg/5ml oral solution sugar free (AMCo) |
| 32876 | Prochlorperazine 5mg tablets (Teva UK Ltd) |
| 54429 | Stemetil 5mg tablets (Waymade Healthcare Plc) |
| 61592 | Prochlorperazine 5mg tablets (Almus Pharmaceuticals Ltd) |
| 41663 | Trifluoperazine 5mg tablets (A A H Pharmaceuticals Ltd) |
| 34344 | Prochlorperazine 5mg tablets (A A H Pharmaceuticals Ltd) |
| 32772 | Prochlorperazine 5mg tablets (Generics (UK) Ltd) |
| 1318 | Stelazine 1mg tablets (Mercury Pharma Group Ltd) |
| 1234 | Stemetil 25mg suppositories (Sanofi) |
| 50462 | Stemetil 5mg tablets (DE Pharmaceuticals) |
| 32551 | Prochlorperazine 5mg tablets (IVAX Pharmaceuticals UK Ltd) |
| 55038 | Prochlorperazine 5mg tablets (Sigma Pharmaceuticals Plc) |
| 40001 | Stemetil 5mg/5ml syrup (Sanofi) |
| 227 | Stemetil 5mg suppositories (Sanofi) |
| 51579 | Stemetil 5mg tablets (Sigma Pharmaceuticals Plc) |
| 39887 | Stemetil 5mg tablets (Sanofi) |
| 1434 | Prochlorperazine 5mg suppositories |
| 49170 | Stemetil 5mg tablets (Lexon (UK) Ltd) |
| 1990 | Prochlorperazine 25mg suppositories |
| 85 | Prochlorperazine 5mg tablets |
| 40162 | Trifluoperazine 1mg tablets (A A H Pharmaceuticals Ltd) |
| 1857 | Trifluoperazine 1mg tablets |
| 29948 | Stelazine Forte 1mg/ml oral solution (Mercury Pharma Group Ltd) |
| 1245 | Trifluoperazine 5mg tablets |
| 62115 | Prochlorperazine 5mg tablets (Genesis Pharmaceuticals Ltd) |
| 8985 | Stelazine 1mg/5ml syrup (Mercury Pharma Group Ltd) |
| 13607 | Proziere 5mg tablets (Ashbourne Pharmaceuticals Ltd) |
| 1316 | Stelazine 5mg tablets (Mercury Pharma Group Ltd) |
| 60908 | Chlorpromazine 10mg capsules |
| 6036 | Prochlorperazine 5mg/5ml oral solution |
| 11531 | Trifluoperazine 5mg/5ml oral solution sugar free |
| 14364 | Prochlorperazine 12.5mg/1ml solution for injection ampoules |
| 13145 | Trifluoperazine 1mg/5ml oral solution sugar free |
| 14356 | Stemetil 12.5mg/1ml solution for injection ampoules (Sanofi) |
| 609 | Perphenazine 2mg tablets |
| 2157 | Perphenazine 4mg tablets |
| 4876 | Amisulpride 50mg tablets |
| 40587 | Clozapine 200mg tablets |
| 38912 | Quetiapine 200mg modified-release tablets |
| 38885 | Quetiapine 50mg modified-release tablets |
| 16908 | Risperdal Consta 25mg powder and solvent for suspension for injection vials (Janssen-Cilag Ltd) |
| 58936 | Tenprolide XL 50mg tablets (Actavis UK Ltd) |
| 46764 | Quetiapine 12.5mg/5ml oral solution |
| 58935 | Tenprolide XL 400mg tablets (Actavis UK Ltd) |
| 8047 | Clozapine 100mg tablets |
| 46889 | Amisulpride 25mg/5ml oral solution |
| 59215 | Quetiapine 150mg tablets (Ranbaxy (UK) Ltd) |
| 667 | Risperidone 500microgram tablets |
| 44326 | Seroquel XL 150mg tablets (AstraZeneca UK Ltd) |
| 49696 | Quetiapine 25mg/5ml oral solution |
| 38937 | Seroquel XL 300mg tablets (AstraZeneca UK Ltd) |
| 45444 | Denzapine 50mg/ml oral suspension (Britannia Pharmaceuticals Ltd) |
| 26544 | Solian 100 tablets (Sanofi) |
| 631 | Risperdal 500microgram tablets (Janssen-Cilag Ltd) |
| 57217 | Risperidone 1mg tablets (Generics (UK) Ltd) |
| 40779 | Quetiapine 100mg/5ml oral solution |
| 53552 | Quetiapine 25mg tablets (Zentiva) |
| 57612 | Seroquel XL 400mg tablets (Lexon (UK) Ltd) |
| 44024 | Quetiapine 150mg modified-release tablets |
| 47302 | Zaponex 100mg tablets (Teva UK Ltd) |
| 57034 | Sondate XL 200mg tablets (Teva UK Ltd) |
| 11938 | Amisulpride 25mg/5ml oral suspension |
| 21199 | Denzapine 100mg tablets (Britannia Pharmaceuticals Ltd) |
| 61747 | Ebesque XL 300mg tablets (DB Ashbourne Ltd) |
| 41714 | Amisulpride 50mg tablets (Zentiva) |
| 30487 | Denzapine 25mg tablets (Britannia Pharmaceuticals Ltd) |
| 14789 | Risperdal Consta 37.5mg powder and solvent for suspension for injection vials (Janssen-Cilag Ltd) |
| 38080 | Abilify 1mg/ml oral solution (Otsuka Pharmaceuticals (U.K.) Ltd) |
| 57412 | Sondate XL 400mg tablets (Teva UK Ltd) |
| 16575 | Aripiprazole 1mg/ml oral solution |
| 38906 | Quetiapine 300mg modified-release tablets |
| 55661 | Risperidone 1mg tablets (Kent Pharmaceuticals Ltd) |
| 46871 | Quetiapine 12.5mg/5ml oral suspension |
| 56387 | Risperidone 1mg/ml oral solution sugar free (Alliance Healthcare (Distribution) Ltd) |
| 49699 | Abilify 5mg tablets (Sigma Pharmaceuticals Plc) |
| 38010 | Abilify 15mg orodispersible tablets (Otsuka Pharmaceuticals (U.K.) Ltd) |
| 2787 | Risperidone 4mg tablets |
| 55625 | Amisulpride 50mg/5ml oral suspension |
| 5927 | Amisulpride 400mg tablets |
| 54483 | Sondate XL 300mg tablets (Teva UK Ltd) |
| 56215 | Quetiapine 50mg/5ml oral solution |
| 18013 | Seroquel 100mg tablets (AstraZeneca UK Ltd) |
| 58821 | Quetiapine 25mg tablets (Dr Reddy's Laboratories (UK) Ltd) |
| 16425 | Risperidone 37.5mg powder and solvent for suspension for injection vials |
| 9794 | Quetiapine 200mg tablets |
| 16006 | Risperdal Quicklet 2mg orodispersible tablets (Janssen-Cilag Ltd) |
| 16489 | Risperidone 50mg powder and solvent for suspension for injection vials |
| 14858 | Abilify 15mg tablets (Otsuka Pharmaceuticals (U.K.) Ltd) |
| 9475 | Risperdal 4mg tablets (Janssen-Cilag Ltd) |
| 56647 | Quetiapine 300mg tablets (Arrow Generics Ltd) |
| 38914 | Seroquel XL 200mg tablets (AstraZeneca UK Ltd) |
| 18132 | Abilify 5mg tablets (Otsuka Pharmaceuticals (U.K.) Ltd) |
| 35548 | Risperdal Quicklet 3mg orodispersible tablets (Janssen-Cilag Ltd) |
| 40586 | Clozapine 50mg tablets |
| 11799 | Risperdal 6mg tablets (Janssen-Cilag Ltd) |
| 46677 | Risperidone 500microgram tablets (Actavis UK Ltd) |
| 14112 | Clozaril 100mg tablets (Novartis Pharmaceuticals UK Ltd) |
| 36954 | Invega 6mg modified-release tablets (Janssen-Cilag Ltd) |
| 35953 | Risperdal Quicklet 4mg orodispersible tablets (Janssen-Cilag Ltd) |
| 51558 | Amisulpride 12.5mg/5ml oral suspension |
| 51240 | Risperidone 125micrograms/5ml oral solution |
| 46705 | Abilify 9.75mg/1.3ml solution for injection vials (Otsuka Pharmaceuticals (U.K.) Ltd) |
| 59829 | Risperidone 3mg tablets (A A H Pharmaceuticals Ltd) |
| 61075 | Abilify Maintena 400mg powder and solvent for suspension for injection vials (Otsuka Pharmaceuticals (U.K.) Ltd) |
| 6373 | Risperidone 1mg orodispersible tablets sugar free |
| 21709 | Seroquel 300mg tablets (AstraZeneca UK Ltd) |
| 2786 | Risperidone 6mg tablets |
| 35141 | Risperidone 3mg orodispersible tablets sugar free |
| 41428 | Denzapine 200mg tablets (Britannia Pharmaceuticals Ltd) |
| 16434 | Risperidone 25mg powder and solvent for suspension for injection vials |
| 5039 | Quetiapine 100mg tablets |
| 16768 | Solian 50 tablets (Sanofi) |
| 6864 | Seroquel 200mg tablets (AstraZeneca UK Ltd) |
| 60842 | Quetiapine 100mg tablets (Alliance Healthcare (Distribution) Ltd) |
| 58067 | Quetiapine 125mg/5ml oral suspension |
| 6573 | Aripiprazole 15mg tablets |
| 38913 | Seroquel XL 50mg tablets (AstraZeneca UK Ltd) |
| 57613 | Seroquel XL 50mg tablets (Sigma Pharmaceuticals Plc) |
| 38840 | Quetiapine 400mg modified-release tablets |
| 61748 | Ebesque XL 200mg tablets (DB Ashbourne Ltd) |
| 11828 | Risperidone 2mg orodispersible tablets sugar free |
| 16986 | Risperdal Quicklet 1mg orodispersible tablets (Janssen-Cilag Ltd) |
| 34927 | Amisulpride 200mg tablets (Zentiva) |
| 62202 | Amisulpride 50mg tablets (A A H Pharmaceuticals Ltd) |
| 52076 | Amisulpride 12.5mg/5ml oral solution |
| 47233 | Zaponex 25mg tablets (Teva UK Ltd) |
| 7382 | Risperidone 500microgram orodispersible tablets sugar free |
| 6524 | Amisulpride 100mg tablets |
| 37717 | Paliperidone 3mg modified-release tablets |
| 11821 | Risperdal Quicklet 500microgram orodispersible tablets (Janssen-Cilag Ltd) |
| 7039 | Quetiapine 300mg tablets |
| 4820 | Risperdal 1mg tablets (Janssen-Cilag Ltd) |
| 29879 | Abilify 30mg tablets (Otsuka Pharmaceuticals (U.K.) Ltd) |
| 8046 | Clozapine 25mg tablets |
| 40932 | Quetiapine 100mg/5ml oral suspension |
| 35589 | Risperidone 4mg orodispersible tablets sugar free |
| 14859 | Seroquel 25mg tablets (AstraZeneca UK Ltd) |
| 6482 | Amisulpride 100mg/ml oral solution sugar free |
| 14813 | Seroquel 150mg tablets (AstraZeneca UK Ltd) |
| 5071 | Amisulpride 200mg tablets |
| 52940 | Sondate XL 50mg tablets (Teva UK Ltd) |
| 58425 | Seroquel XL 50mg tablets (DE Pharmaceuticals) |
| 37501 | Paliperidone 9mg modified-release tablets |
| 58822 | Risperidone 4mg tablets (Almus Pharmaceuticals Ltd) |
| 36116 | Paliperidone 6mg modified-release tablets |
| 62531 | Atrolak XL 400mg tablets (Accord Healthcare Ltd) |
| 6561 | Aripiprazole 10mg tablets |
| 1321 | Risperidone 2mg tablets |
| 4992 | Solian 200 tablets (Sanofi) |
| 51178 | Quetiapine 50mg/5ml oral suspension |
| 31576 | Solian 100mg/ml oral solution (Sanofi) |
| 46969 | Amisulpride 200mg tablets (A A H Pharmaceuticals Ltd) |
| 45839 | Quetiapine 25mg/5ml oral suspension |
| 57114 | Abilify 5mg tablets (Mawdsley-Brooks & Company Ltd) |
| 9659 | Risperdal 2mg tablets (Janssen-Cilag Ltd) |
| 5219 | Risperidone 3mg tablets |
| 14344 | Aripiprazole 5mg tablets |
| 47832 | Risperidone 500microgram tablets (A A H Pharmaceuticals Ltd) |
| 5283 | Quetiapine 25mg tablets |
| 61746 | Ebesque XL 400mg tablets (DB Ashbourne Ltd) |
| 5262 | Risperdal 1mg/ml liquid (Janssen-Cilag Ltd) |
| 61650 | Aripiprazole 400mg powder and solvent for suspension for injection vials |
| 61575 | Ebesque XL 50mg tablets (DB Ashbourne Ltd) |
| 14767 | Risperdal Consta 50mg powder and solvent for suspension for injection vials (Janssen-Cilag Ltd) |
| 17958 | Clozaril 25mg tablets (Novartis Pharmaceuticals UK Ltd) |
| 31098 | Aripiprazole 15mg orodispersible tablets sugar free |
| 42242 | Clozapine 50mg/ml oral suspension sugar free |
| 39237 | Seroquel XL 400mg tablets (AstraZeneca UK Ltd) |
| 59548 | Risperidone 2mg tablets (Alliance Healthcare (Distribution) Ltd) |
| 37606 | Abilify 10mg orodispersible tablets (Otsuka Pharmaceuticals (U.K.) Ltd) |
| 41070 | Denzapine 50mg tablets (Britannia Pharmaceuticals Ltd) |
| 32076 | Aripiprazole 10mg orodispersible tablets sugar free |
| 38375 | Aripiprazole 9.75mg/1.3ml solution for injection vials |
| 54346 | Risperidone 1mg tablets (Teva UK Ltd) |
| 24358 | Abilify 10mg tablets (Otsuka Pharmaceuticals (U.K.) Ltd) |
| 41702 | Amisulpride 100mg tablets (Zentiva) |
| 1320 | Risperidone 1mg tablets |
| 9340 | Risperdal 3mg tablets (Janssen-Cilag Ltd) |
| 16561 | Aripiprazole 30mg tablets |
| 302 | Risperidone 1mg/ml oral solution sugar free |
| 6109 | Solian 400 tablets (Sanofi) |
| 51444 | Risperdal Consta 50mg powder and solvent for suspension for injection vials (Waymade Healthcare Plc) |
| 5040 | Quetiapine 150mg tablets |
| 46422 | Olanzapine embonate 300mg powder and solvent for suspension for injection vials |
| 43914 | Olanzapine embonate 210mg powder and solvent for suspension for injection vials |
| 55268 | Zypadhera 300mg powder and solvent for suspension for injection vials (Eli Lilly and Company Ltd) |
| 47093 | Olanzapine 20mg orodispersible tablets sugar free |
| 47103 | Olanzapine 15mg orodispersible tablets sugar free |
| 55622 | Olanzapine 10mg orodispersible tablets |
| 47063 | Olanzapine 10mg orodispersible tablets sugar free |
| 29540 | Olanzapine 20mg tablets |
| 56265 | Olanzapine 20mg oral lyophilisates sugar free |
| 59143 | Olanzapine 2.5mg tablets (Teva UK Ltd) |
| 47098 | Olanzapine 5mg orodispersible tablets sugar free |
| 50214 | Olanzapine 5mg orodispersible tablets |
| 16407 | Zyprexa 15mg Velotabs (Eli Lilly and Company Ltd) |
| 14717 | Zyprexa 5mg Velotabs (Eli Lilly and Company Ltd) |
| 2656 | Olanzapine 2.5mg tablets |
| 21964 | Zyprexa 7.5mg tablets (Eli Lilly and Company Ltd) |
| 57616 | Olanzapine 20mg tablets (Teva UK Ltd) |
| 1249 | Olanzapine 10mg tablets |
| 13888 | Zyprexa 10mg Velotabs (Eli Lilly and Company Ltd) |
| 36163 | Zyprexa 20mg tablets (Eli Lilly and Company Ltd) |
| 61103 | Olanzapine 15mg oral lyophilisates sugar free |
| 52001 | Olanzapine 2.5mg tablets (Aspire Pharma Ltd) |
| 56143 | Olanzapine 15mg orodispersible tablets |
| 55667 | Olanzapine 15mg tablets (Actavis UK Ltd) |
| 57270 | Olanzapine 2.5mg/5ml oral suspension |
| 53848 | Zalasta 5mg orodispersible tablets (Consilient Health Ltd) |
| 5653 | Olanzapine 7.5mg tablets |
| 3281 | Olanzapine 5mg tablets |
| 23431 | Olanzapine 10mg powder for solution for injection vials |
| 58854 | Olanzapine 10mg tablets (Actavis UK Ltd) |
| 13820 | Zyprexa 10mg tablets (Eli Lilly and Company Ltd) |
| 58147 | Olanzapine 10mg tablets (Zentiva) |
| 53556 | Olanzapine 10mg oral lyophilisates sugar free |
| 18453 | Zyprexa 2.5mg tablets (Eli Lilly and Company Ltd) |
| 45953 | Zyprexa 10mg powder for solution for injection vials (Eli Lilly and Company Ltd) |
| 19976 | Zyprexa 15mg tablets (Eli Lilly and Company Ltd) |
| 56072 | Olanzapine 20mg orodispersible tablets |
| 33883 | Zyprexa 20mg Velotabs (Eli Lilly and Company Ltd) |
| 18024 | Zyprexa 5mg tablets (Eli Lilly and Company Ltd) |
| 57160 | Olanzapine 5mg oral lyophilisates sugar free |
| 6850 | Olanzapine 15mg tablets |

## Central nervous system (CNS) stimulant prescription (BNF Chapter 4.4 CNS simulants and drugs used for attention deficit hyperactivity disorder)

Prescription of CNS medication was rare in the CPRD (e.g. ≤1% prevalence) and therefore was included in the other psychotropic medications category.

CNS stimulant prescription code list

| Product name | Product code |
| --- | --- |
| 21068 | Villescon Tablet (Boehringer Ingelheim Ltd) |
| 6804 | Equasym 20mg tablets (Shire Pharmaceuticals Ltd) |
| 17588 | Strattera 60mg capsules (Eli Lilly and Company Ltd) |
| 21399 | Equasym xl 20mg Capsule (Celltech Pharma Europe Ltd) |
| 13238 | Dexamfetamine 1mg/ml oral liquid |
| 54504 | Methylphenidate 20mg modified-release tablets |
| 2027 | Pemoline 20mg tablets |
| 19289 | Volital 20mg Tablet (Laboratories For Applied Biology Ltd) |
| 61390 | Methylphenidate 50mg modified-release capsules |
| 13914 | Equasym 5mg tablets (Shire Pharmaceuticals Ltd) |
| 13946 | Equasym 10mg tablets (Shire Pharmaceuticals Ltd) |
| 6645 | Atomoxetine 25mg capsules |
| 35159 | Concerta XL 27mg tablets (Janssen-Cilag Ltd) |
| 35469 | Methylphenidate 27mg modified-release tablets |
| 47609 | Dexamfetamine 5mg modified-release capsules |
| 56576 | Elvanse 30mg capsules (Shire Pharmaceuticals Ltd) |
| 16949 | Strattera 25mg capsules (Eli Lilly and Company Ltd) |
| 61800 | Matoride XL 36mg tablets (Sandoz Ltd) |
| 31623 | Dexedrine 15mg Spansules (Imported (United States)) |
| 52233 | Methylphenidate 54mg modified-release tablets |
| 7100 | Atomoxetine 10mg capsules |
| 59365 | Strattera 100mg capsules (Eli Lilly and Company Ltd) |
| 56742 | Elvanse 70mg capsules (Shire Pharmaceuticals Ltd) |
| 17014 | Strattera 40mg capsules (Eli Lilly and Company Ltd) |
| 62613 | Xenidate XL 36mg tablets (Generics (UK) Ltd) |
| 6644 | Atomoxetine 60mg capsules |
| 23800 | Caffeine iodide liquid |
| 40279 | Atomoxetine 80mg capsules |
| 14331 | Equasym XL 30mg capsules (Shire Pharmaceuticals Ltd) |
| 57405 | Methylphenidate 5mg/5ml oral solution |
| 6643 | Atomoxetine 40mg capsules |
| 6326 | Modafinil 100mg tablets |
| 62584 | Tranquilyn 20mg tablets (Genesis Pharmaceuticals Ltd) |
| 17244 | Villescon Liquid (Boehringer Ingelheim Ltd) |
| 62445 | Dexamfetamine 5mg/5ml oral solution sugar free |
| 31972 | Prolintane hydrochloride with vitamins liquid |
| 55495 | Dexamfetamine with amfetamine 10mg with 10mg capsules |
| 51453 | Dexamfetamine 5mg/5ml oral solution |
| 41492 | Strattera 80mg capsules (Eli Lilly and Company Ltd) |
| 61144 | Medikinet XL 60mg capsules (Flynn Pharma Ltd) |
| 60988 | Medikinet XL 50mg capsules (Flynn Pharma Ltd) |
| 2679 | Ritalin 10mg tablets (Novartis Pharmaceuticals UK Ltd) |
| 11733 | Methylphenidate 20mg tablets |
| 55635 | Atomoxetine 100mg capsules |
| 18996 | Durophet 20mg Capsule (3M Health Care Ltd) |
| 35659 | Medikinet XL 20mg capsules (Flynn Pharma Ltd) |
| 19944 | Provigil 100mg tablets (Teva UK Ltd) |
| 58055 | Dexmethylphenidate 10mg modified-release capsules |
| 9738 | Dexamfetamine 5mg tablets |
| 14129 | Atomoxetine 18mg capsules |
| 49392 | Amfetamine 10mg / Dexamfetamine 10mg modified-release capsules |
| 37658 | Medikinet XL 40mg capsules (Flynn Pharma Ltd) |
| 7101 | Methylphenidate 5mg tablets |
| 56336 | Elvanse 50mg capsules (Shire Pharmaceuticals Ltd) |
| 16185 | Dexamfetamine 15mg modified-release capsules |
| 58678 | Concerta 54mg modified-release tablets (Imported (Belgium)) |
| 47099 | Dexamfetamine with amfetamine 10mg with 10mg modified-release capsules |
| 576 | Methylphenidate 10mg tablets |
| 35515 | Methylphenidate 40mg modified-release capsules |
| 62487 | Matoride XL 54mg tablets (Sandoz Ltd) |
| 6169 | Methylphenidate 36mg modified-release tablets |
| 14119 | Strattera 10mg capsules (Eli Lilly and Company Ltd) |
| 48029 | Caffeine oral solution |
| 53527 | Equasym XL 30mg capsules (Waymade Healthcare Plc) |
| 13212 | Methylphenidate 10mg modified-release capsules |
| 47679 | Dexamfetamine 15mg modified-release capsules |
| 58691 | Methylphenidate 10mg/5ml oral solution |
| 23173 | Tranquilyn 10mg tablets (Genesis Pharmaceuticals Ltd) |
| 55169 | Lisdexamfetamine 50mg capsules |
| 6868 | Equasym XL 20mg capsules (Shire Pharmaceuticals Ltd) |
| 14848 | Methylphenidate 30mg modified-release capsules |
| 36628 | Medikinet XL 10mg capsules (Flynn Pharma Ltd) |
| 57786 | Lisdexamfetamine 70mg capsules |
| 24116 | Durophet 12.5mg Capsule (3M Health Care Ltd) |
| 46593 | Medikinet XL 5mg capsules (Flynn Pharma Ltd) |
| 18832 | Strattera 18mg capsules (Eli Lilly and Company Ltd) |
| 13175 | Modafinil 200mg tablets |
| 56713 | Ritalin-SR 20mg tablets (Imported (United States)) |
| 11536 | Methylphenidate 20mg modified-release capsules |
| 35658 | Medikinet XL 30mg capsules (Flynn Pharma Ltd) |
| 37097 | Medikinet 5mg tablets (Flynn Pharma Ltd) |
| 14814 | Provigil 200mg tablets (Teva UK Ltd) |
| 55747 | Elvanse 30mg capsules (Shire Pharmaceuticals Ltd) |
| 54804 | Equasym XL 10mg capsules (DE Pharmaceuticals) |
| 47481 | Dexamfetamine 10mg modified-release capsules |
| 46607 | Methylphenidate 5mg modified-release capsules |
| 23161 | Tranquilyn 5mg tablets (Genesis Pharmaceuticals Ltd) |
| 18998 | Durophet 7.5mg Capsule (3M Health Care Ltd) |
| 6107 | Methylphenidate 18mg modified-release tablets |
| 14512 | Dexedrine 5mg tablets (Auden McKenzie (Pharma Division) Ltd) |
| 52461 | Equasym XL 10mg capsules (Waymade Healthcare Plc) |
| 37237 | Medikinet 10mg tablets (Flynn Pharma Ltd) |
| 5810 | Concerta XL 18mg tablets (Janssen-Cilag Ltd) |
| 14346 | Equasym XL 10mg capsules (Shire Pharmaceuticals Ltd) |
| 36910 | Medikinet 20mg tablets (Flynn Pharma Ltd) |
| 5811 | Concerta XL 36mg tablets (Janssen-Cilag Ltd) |
| 55987 | Lisdexamfetamine 30mg capsules |

## Dementia medication prescription (BNF Chapter 4.11 Dementia drugs)

Prescription of dementia medication was rare in the CPRD (e.g. ≤1% prevalence) and therefore was included in the other psychotropic medications category.

Dementia medication code list

| Product code | Product name |
| --- | --- |
| 39363 | Ebixa 20mg tablets (Lundbeck Ltd) |
| 58937 | Exelon 13.3mg/24hours transdermal patches (Novartis Pharmaceuticals UK Ltd) |
| 6225 | Memantine 10mg tablets |
| 11751 | Rivastigmine 3mg capsules |
| 7329 | Galantamine 20mg/5ml oral solution sugar free |
| 60723 | Rivastigmine 6mg capsules (Waymade Healthcare Plc) |
| 58780 | Voleze 9.5mg/24hours transdermal patches (Focus Pharmaceuticals Ltd) |
| 39362 | Ebixa tablets treatment initiation pack (Lundbeck Ltd) |
| 56631 | Rivastigmine 13.3mg/24hours transdermal patches |
| 37132 | Rivastigmine 9.5mg/24hours transdermal patches |
| 56771 | Rivastigmine 3mg capsules (Dr Reddy's Laboratories (UK) Ltd) |
| 20404 | Exelon 4.5mg capsules (Novartis Pharmaceuticals UK Ltd) |
| 57171 | Erastig 9.5mg/24hours transdermal patches (Teva UK Ltd) |
| 61676 | Donepezil 1mg/ml oral solution sugar free |
| 24088 | Reminyl XL 24mg capsules (Shire Pharmaceuticals Ltd) |
| 11635 | Galantamine 12mg tablets |
| 60192 | Galantex XL 8mg capsules (Creo Pharma Ltd) |
| 57627 | Erastig 4.6mg/24hours transdermal patches (Teva UK Ltd) |
| 11654 | Galantamine 8mg tablets |
| 2930 | Donepezil 5mg tablets |
| 5616 | Exelon 6mg capsules (Novartis Pharmaceuticals UK Ltd) |
| 58969 | Rivastigmine 4.6mg/24hours transdermal patches (A A H Pharmaceuticals Ltd) |
| 48482 | Galsya XL 8mg capsules (Consilient Health Ltd) |
| 48442 | Donepezil 5mg orodispersible tablets |
| 55928 | Exelon 4.5mg capsules (Waymade Healthcare Plc) |
| 53882 | Rivastigmine 2mg/ml oral solution |
| 58709 | Donepezil 10mg tablets (A A H Pharmaceuticals Ltd) |
| 59871 | Donepezil 10mg/5ml oral suspension |
| 55720 | Gatalin XL 24mg capsules (Aspire Pharma Ltd) |
| 7361 | Galantamine 24mg modified-release capsules |
| 61476 | Acumor XL 24mg capsules (Generics (UK) Ltd) |
| 57139 | Ebixa 10mg tablets (DE Pharmaceuticals) |
| 62164 | Alzest 9.5mg/24hours transdermal patches (Dr Reddy's Laboratories (UK) Ltd) |
| 53922 | Donepezil 10mg orodispersible tablets (Consilient Health Ltd) |
| 36848 | Aricept Evess 5mg orodispersible tablets (Eisai Ltd) |
| 60493 | Galantex XL 24mg capsules (Creo Pharma Ltd) |
| 29288 | Reminyl 4mg/ml oral solution (Shire Pharmaceuticals Ltd) |
| 9966 | Ebixa 5mg/pump actuation oral solution (Lundbeck Ltd) |
| 58947 | Donepezil 10mg tablets (Accord Healthcare Ltd) |
| 5247 | Aricept 10mg tablets (Eisai Ltd) |
| 11716 | Exelon 3mg capsules (Novartis Pharmaceuticals UK Ltd) |
| 61920 | Luventa XL 8mg capsules (Fontus Health Ltd) |
| 10187 | Galantamine 4mg tablets |
| 37444 | Exelon 4.6mg/24hours transdermal patches (Novartis Pharmaceuticals UK Ltd) |
| 18587 | Reminyl XL 8mg capsules (Shire Pharmaceuticals Ltd) |
| 56421 | Gatalin XL 8mg capsules (Aspire Pharma Ltd) |
| 10255 | Galantamine 8mg modified-release capsules |
| 4597 | Rivastigmine 1.5mg capsules |
| 9854 | Reminyl 4mg tablets (Shire Pharmaceuticals Ltd) |
| 61385 | Nemdatine 10mg tablets (Actavis UK Ltd) |
| 11546 | Exelon 1.5mg capsules (Novartis Pharmaceuticals UK Ltd) |
| 14309 | Galantamine 16mg modified-release capsules |
| 37188 | Aricept Evess 10mg orodispersible tablets (Eisai Ltd) |
| 5334 | Reminyl 12mg tablets (Shire Pharmaceuticals Ltd) |
| 56709 | Gatalin XL 16mg capsules (Aspire Pharma Ltd) |
| 2931 | Donepezil 10mg tablets |
| 61921 | Luventa XL 24mg capsules (Fontus Health Ltd) |
| 11827 | Rivastigmine 2mg/ml oral solution sugar free |
| 37957 | Exelon 9.5mg/24hours transdermal patches (Novartis Pharmaceuticals UK Ltd) |
| 48015 | Galsya XL 24mg capsules (Consilient Health Ltd) |
| 53842 | Aricept 5mg tablets (Waymade Healthcare Plc) |
| 18800 | Ebixa 10mg tablets (Lundbeck Ltd) |
| 59993 | Galantex XL 16mg capsules (Creo Pharma Ltd) |
| 36976 | Rivastigmine 4.6mg/24hours transdermal patches |
| 56600 | Donepezil 5mg tablets (Zentiva) |
| 35088 | Donepezil 10mg orodispersible tablets sugar free |
| 5400 | Aricept 5mg tablets (Eisai Ltd) |
| 59330 | Voleze 4.6mg/24hours transdermal patches (Focus Pharmaceuticals Ltd) |
| 18062 | Reminyl 8mg tablets (Shire Pharmaceuticals Ltd) |
| 11752 | Rivastigmine 4.5mg capsules |
| 38976 | Memantine 5mg+10mg+15mg+20mg Tablet |
| 35179 | Donepezil 5mg orodispersible tablets sugar free |
| 60107 | Donepezil 5mg tablets (Alliance Healthcare (Distribution) Ltd) |
| 20140 | Reminyl XL 16mg capsules (Shire Pharmaceuticals Ltd) |
| 39240 | Memantine 20mg tablets |
| 18556 | Exelon 2mg/ml oral solution (Novartis Pharmaceuticals UK Ltd) |
| 9786 | Rivastigmine 6mg capsules |
| 11837 | Memantine 10mg/ml oral solution sugar free |
| 48443 | Donepezil 10mg orodispersible tablets |
| 61618 | Nemdatine 20mg tablets (Actavis UK Ltd) |
| 12843 | Ginkyo 120mg tablets (Ceuta Healthcare Ltd) |
| 30120 | Ginkyo 50mg tablets (Ceuta Healthcare Ltd) |
| 61128 | HealthAid Ginko Vital (Biloba) 5g capsules (HealthAid Ltd) |

## Hypnotics and anxiolytics prescription (BNF Chapters 4.1 Hypnotics and anxiolytics)

Hypnotics and anxiolytics prescription code list

| Product code | Product name |
| --- | --- |
| 4439 | Libraxin Tablet (Roche Products Ltd) |
| 8742 | Clidinium bromide with Chlordiazepoxide tablets |
| 41988 | Chlordiazepoxide 10mg Tablet (DDSA Pharmaceuticals Ltd) |
| 17294 | Librium 10mg Tablet (ICN Pharmaceuticals France S.A.) |
| 34928 | Chlordiazepoxide 5mg Capsule (DDSA Pharmaceuticals Ltd) |
| 45275 | Buspirone 5mg tablets (Actavis UK Ltd) |
| 6516 | Chlordiazepoxide 25mg tablets |
| 28879 | Chlordiazepoxide 5mg Tablet (DDSA Pharmaceuticals Ltd) |
| 10789 | Nobrium 10mg Capsule (Roche Products Ltd) |
| 28360 | Ketazolam 30mg Capsule |
| 8913 | Librium 10mg Capsule (ICN Pharmaceuticals France S.A.) |
| 41574 | Chlordiazepoxide 10mg Capsule (Approved Prescription Services Ltd) |
| 41542 | Oxazepam 10mg Tablet (IVAX Pharmaceuticals UK Ltd) |
| 11958 | Lexotan 1.5mg Tablet (Roche Products Ltd) |
| 12452 | Anxon 15mg Capsule (Beecham Research Laboratories) |
| 4140 | Oxazepam 30mg Capsule |
| 10791 | Nobrium 5mg Capsule (Roche Products Ltd) |
| 22424 | Bromazepam 1.5mg tablets |
| 40386 | Chlordiazepoxide 25mg Tablet (DDSA Pharmaceuticals Ltd) |
| 21464 | Medazepam 5mg Capsule |
| 41629 | Chlordiazepoxide 10mg Capsule (DDSA Pharmaceuticals Ltd) |
| 12130 | Anxon 30mg Capsule (Beecham Research Laboratories) |
| 32231 | Librium 25mg Tablet (ICN Pharmaceuticals France S.A.) |
| 16169 | Librium 100mg Injection (Roche Products Ltd) |
| 8550 | Chlordiazepoxide 25mg tablets |
| 61443 | Buspirone 10mg tablets (A A H Pharmaceuticals Ltd) |
| 41606 | Chlordiazepoxide 5mg Capsule (Approved Prescription Services Ltd) |
| 2394 | Buspar 5mg tablets (IXL Pharma Ltd) |
| 9048 | Librium 5mg Tablet (ICN Pharmaceuticals France S.A.) |
| 23796 | Ketazolam 15mg Capsule |
| 25273 | Oxanid 10mg Tablet (M A Steinhard Ltd) |
| 41581 | Chlordiazepoxide 10mg Capsule (IVAX Pharmaceuticals UK Ltd) |
| 2828 | Meprobamate 400mg tablets |
| 43240 | Buspirone 5mg tablets (Generics (UK) Ltd) |
| 19941 | Bromazepam 3mg tablets |
| 12477 | Librium 5mg Capsule (ICN Pharmaceuticals France S.A.) |
| 5385 | Buspirone 10mg tablets |
| 4543 | Chlordiazepoxide 5mg tablets |
| 28880 | Buspirone 5mg Tablet (Galen Ltd) |
| 3147 | Chlordiazepoxide 10mg tablets |
| 8721 | Oxazepam 30mg Tablet |
| 9721 | Lexotan 3mg Tablet (Roche Products Ltd) |
| 40153 | Buspirone 10mg Tablet (Galen Ltd) |
| 59095 | Buspirone 5mg tablets (A A H Pharmaceuticals Ltd) |
| 9008 | Buspar 10mg tablets (IXL Pharma Ltd) |
| 25893 | Centrax 10mg Tablet (Parke-davis Research Laboratories) |
| 49504 | Buspar 10mg tablets (Lexon (UK) Ltd) |
| 25007 | Chlordiazepozide 100mg injection |
| 3574 | Buspirone 5mg tablets |
| 10790 | Medazepam 10mg Capsule |
| 41601 | Oxazepam 15mg Tablet (IVAX Pharmaceuticals UK Ltd) |
| 46847 | Buspirone 10mg tablets (Actavis UK Ltd) |
| 8487 | Frisium 10mg Capsule (Aventis Pharma) |
| 3110 | Clobazam 10mg capsules |
| 8344 | Diazepam 5mg suppository |
| 23820 | Diazepam 20mg rectal tubes |
| 5793 | Stesolid 10mg Rectal tubes (Dumex Ltd) |
| 20514 | Valium 10mg Suppository (Roche Products Ltd) |
| 16610 | Valium 5mg/ml Injection (Roche Products Ltd) |
| 38410 | Diazepam 5mg Rectal tubes (Hillcross Pharmaceuticals Ltd) |
| 28698 | Valium 5mg Suppository (Roche Products Ltd) |
| 41689 | Diazepam 10mg Suppository (Sinclair IS Pharma Plc) |
| 16734 | Diazepam rectubes 20mg Rectal tubes (C P Pharmaceuticals Ltd) |
| 1909 | Trancopal 200mg Tablet (Sanofi-Synthelabo Ltd) |
| 12038 | Chlormezanone 200mg tablets |
| 12484 | Equanil 200mg Tablet (Wyeth Pharmaceuticals) |
| 3639 | Meprobamate 200mg Tablet |
| 12512 | Equanil 400mg Tablet (Wyeth Pharmaceuticals) |
| 24642 | Milonorm 400mg Tablet (Wallace Manufacturing Chemists Ltd) |
| 12264 | Seconal Sodium 100mg capsules (Flynn Pharma Ltd) |
| 26775 | Secobarbital sodium 50mg capsules |
| 8548 | Amobarbital sodium 200mg capsules |
| 12539 | Seconal Sodium 50mg capsules (Flynn Pharma Ltd) |
| 14416 | Sodium amytal 60mg Tablet (Flynn Pharma Ltd) |
| 14415 | Sodium Amytal 60mg capsules (Flynn Pharma Ltd) |
| 28146 | Amobarbital sodium 60mg capsules |
| 15583 | Cyclobarbitone calcium 200mg tablets |
| 35776 | Amobarbital sodium 250mg injection |
| 13470 | Sodium amytal 200mg Tablet (Flynn Pharma Ltd) |
| 27481 | Amytal 200mg Tablet (Flynn Pharma Ltd) |
| 4134 | Amobarbital 30mg tablets |
| 14613 | Secobarbital sodium 100mg capsules |
| 17102 | Amobarbital 15mg tablets |
| 23748 | Sodium amytal 250mg Injection (Flynn Pharma Ltd) |
| 8713 | Amytal 30mg Tablet (Flynn Pharma Ltd) |
| 15614 | Amobarbital 200mg tablets |
| 43261 | Sodium amytal 500mg Injection (Flynn Pharma Ltd) |
| 12010 | Amytal 15mg Tablet (Flynn Pharma Ltd) |
| 15615 | Amobarbital 100mg tablets |
| 13411 | Amytal 100mg Tablet (Flynn Pharma Ltd) |
| 221 | Tuinal 100mg Pulvules (Flynn Pharma Ltd) |
| 14414 | Amobarbital sodium 60mg tablets |
| 21460 | Amobarbital 50mg / Secobarbital sodium 50mg capsules |
| 13471 | Sodium Amytal 200mg capsules (Flynn Pharma Ltd) |
| 10311 | Thiopental 500mg powder for solution for injection vials |
| 52022 | Zimovane 7.5mg tablets (Lexon (UK) Ltd) |
| 58566 | Melatonin 4mg/5ml oral solution |
| 21437 | Loramet 1mg Capsule (Wyeth Pharmaceuticals) |
| 34408 | Nitrazepam 5mg tablets (A A H Pharmaceuticals Ltd) |
| 4017 | Chloral hydrate 500mg/5ml oral suspension |
| 48517 | Lormetazepam 1mg/5ml oral suspension |
| 52303 | Melatonin 20mg capsules |
| 34874 | Zopiclone 7.5mg tablets (Kent Pharmaceuticals Ltd) |
| 14210 | Melatonin 2.5mg/5ml oral suspension |
| 24321 | Paxane 30mg Capsule (M A Steinhard Ltd) |
| 3320 | Zimovane 7.5mg tablets (Sanofi) |
| 50115 | Melatonin 4mg capsules |
| 35 | Nitrazepam 5mg tablets |
| 60204 | Chloral hydrate 500mg/5ml oral solution sugar free |
| 18925 | Noludar 200mg Tablet (Roche Products Ltd) |
| 5058 | Zileze 3.75 tablets (Opus Pharmaceuticals Ltd) |
| 4632 | Chloral 200mg/5ml paediatric oral solution |
| 66 | Zopiclone 7.5mg tablets |
| 19073 | Chloral hydrate 500mg/5ml Mixture (Rosemont Pharmaceuticals Ltd) |
| 23205 | Dormonoct 1mg Tablet (Hoechst Marion Roussel) |
| 34770 | Nitrazepam 5mg Tablet (DDSA Pharmaceuticals Ltd) |
| 43445 | Zopiclone 7.5mg tablets (A A H Pharmaceuticals Ltd) |
| 42089 | Zolpidem 10mg Tablet (Winthrop Pharmaceuticals Ltd) |
| 58185 | Chloral hydrate 600mg/5ml oral solution BP |
| 55191 | Melatonin 6mg/5ml oral solution |
| 48436 | Melatonin 6mg capsules |
| 55100 | Melatonin 5mg/5ml oral solution (Drug Tariff Special Order) |
| 4018 | Chloral hydrate 1g/5ml oral suspension |
| 34292 | Lormetazepam 500microgram tablets (A A H Pharmaceuticals Ltd) |
| 1134 | Cloral betaine 707mg tablets |
| 14365 | Zopiclone 3.75mg/5ml oral suspension |
| 62501 | Melatonin 10mg/5ml oral solution |
| 18859 | Somnwell 707mg film coated Tablet (Huntley Pharmaceuticals Ltd) |
| 52487 | Melatonin 2mg/5ml oral solution |
| 3126 | Stilnoct 5mg tablets (Sanofi) |
| 33841 | Zolpidem 10mg tablets (Generics (UK) Ltd) |
| 34642 | Lormetazepam 500microgram tablets (Generics (UK) Ltd) |
| 36312 | Sodium oxybate 500mg/ml oral solution sugar free |
| 41697 | Zolpidem 5mg tablets (IVAX Pharmaceuticals UK Ltd) |
| 45225 | Chloral hydrate oral solution |
| 51578 | Chloral hydrate 200mg/5ml oral solution BP |
| 62006 | Chloral betaine 707mg tablets (A A H Pharmaceuticals Ltd) |
| 45353 | Zopiclone 7.5mg tablets (Sandoz Ltd) |
| 62574 | Chloral betaine 707mg tablets (Marlborough Pharmaceuticals Ltd) |
| 7786 | Mogadon 5mg Tablet (ICN Pharmaceuticals France S.A.) |
| 61763 | Chloral hydrate 300mg/5ml oral solution BP |
| 4187 | Zimovane LS 3.75mg tablets (Sanofi) |
| 34964 | Nitrazepam 5mg Tablet (Berk Pharmaceuticals Ltd) |
| 34555 | Nitrazepam 5mg tablets (Wockhardt UK Ltd) |
| 15492 | Nitrados 5mg Tablet (Rorer Pharmaceuticals Ltd) |
| 7924 | Nitrazepam 2.5mg/5ml oral suspension |
| 45975 | Melatonin 5mg/5ml oral solution |
| 31710 | Zolpidem 5mg tablets (A A H Pharmaceuticals Ltd) |
| 49576 | Melatonin 500microgram tablets |
| 32538 | Potassium bromide & valerian mxtire |
| 16993 | Melatonin 3mg modified-release capsules |
| 61477 | Zopiclone 7.5mg/5ml oral solution |
| 3928 | Welldorm 143.3mg/5ml elixir (Marlborough Pharmaceuticals Ltd) |
| 17663 | Melatonin 1mg tablets |
| 53064 | Melatonin 6mg/5ml oral suspension |
| 62411 | Melatonin 3mg orodispersible tablets |
| 55303 | Triazolam 0.125mg Tablet (Berk Pharmaceuticals Ltd) |
| 41596 | Loprazolam 1mg tablets (Zentiva) |
| 41822 | Triazolam 0.25mg Tablet (Berk Pharmaceuticals Ltd) |
| 45230 | Melatonin Capsule |
| 3357 | Lormetazepam 1mg Capsule |
| 31951 | Triclofos 500mg/5ml Oral solution (UCB Pharma Ltd) |
| 33663 | Zopiclone 7.5mg tablets (Generics (UK) Ltd) |
| 41539 | Zolpidem 10mg tablets (IVAX Pharmaceuticals UK Ltd) |
| 34612 | Zopiclone 3.75mg tablets (A A H Pharmaceuticals Ltd) |
| 30249 | Chloral 500mg capsules |
| 12898 | Chloral hydrate 500mg/5ml mixture BP 2000 |
| 2300 | Welldorm 707mg tablets (Marlborough Pharmaceuticals Ltd) |
| 38265 | Circadin 2mg modified-release tablets (Flynn Pharma Ltd) |
| 13023 | Melatonin 2.5mg capsules |
| 52683 | Melatonin 1mg/5ml oral suspension |
| 54717 | Melatonin 10mg/5ml oral suspension |
| 57937 | Zopiclone 3.75mg tablets (Almus Pharmaceuticals Ltd) |
| 34897 | Zopiclone 3.75mg tablets (Kent Pharmaceuticals Ltd) |
| 30056 | Zopiclone 3.75mg tablets (IVAX Pharmaceuticals UK Ltd) |
| 33045 | Zopiclone 7.5mg tablets (IVAX Pharmaceuticals UK Ltd) |
| 10513 | Triclofos 500mg/5ml oral solution |
| 27847 | Surem 5mg Capsule (Galen Ltd) |
| 35142 | Vytalonin 3mg Tablet (IDIS World Medicines) |
| 46799 | Zopiclone 3.75mg/5ml oral solution |
| 52289 | Melatonin 3mg/5ml oral suspension |
| 50258 | Melatonin 1mg/1ml oral liquid sugar free |
| 34516 | Lormetazepam 1mg tablets (Generics (UK) Ltd) |
| 30981 | Zolpidem 10mg tablets (A A H Pharmaceuticals Ltd) |
| 35810 | Xyrem 500mg/ml oral solution (UCB Pharma Ltd) |
| 18291 | Noctamid 1mg Tablet (Schering Health Care Ltd) |
| 52079 | Melatonin 2mg tablets |
| 34686 | Nitrazepam 5mg tablets (Teva UK Ltd) |
| 5916 | Zaleplon 5mg capsules |
| 5352 | Zaleplon 10mg capsules |
| 56393 | Melatonin 5mg tablets |
| 55892 | Chloral hydrate 1g/5ml oral solution BP |
| 46953 | Nitrazepam 5mg tablets (Ranbaxy (UK) Ltd) |
| 2407 | Nitrazepam 5mg Capsule |
| 721 | Zopiclone 3.75mg tablets |
| 34372 | Zopiclone 7.5mg tablets (PLIVA Pharma Ltd) |
| 15852 | Zileze 7.5 tablets (Opus Pharmaceuticals Ltd) |
| 57406 | Melatonin 1mg/5ml oral solution |
| 60006 | Chloral hydrate 250mg/5ml oral solution BP |
| 29219 | Zopiclone 3.75mg tablets (Actavis UK Ltd) |
| 24135 | Zopiclone 7.5mg tablets (Actavis UK Ltd) |
| 7571 | Triazolam 125microgram Tablet |
| 37325 | Remnos 10mg Tablet (DDSA Pharmaceuticals Ltd) |
| 3524 | Mogadon 5mg Capsule (Roche Products Ltd) |
| 14221 | Melatonin 1mg/ml sugar free Oral solution |
| 43560 | Zolpidem 10mg tablets (Teva UK Ltd) |
| 49196 | Melatonin 2mg/5ml oral suspension |
| 3686 | Nitrazepam 10mg Tablet |
| 19450 | Mogadon 5mg tablets (Meda Pharmaceuticals Ltd) |
| 2017 | Zolpidem 5mg tablets |
| 34823 | Zopiclone 7.5mg tablets (Teva UK Ltd) |
| 7099 | Melatonin 2mg capsules |
| 18976 | Methyprylone 200mg Tablet |
| 59640 | Zopiclone 7.5mg/5ml oral suspension |
| 41961 | Melatonin Tablet |
| 5150 | Loprazolam 1mg tablets |
| 3354 | Lormetazepam 1mg tablets |
| 9598 | Sonata 5mg capsules (Meda Pharmaceuticals Ltd) |
| 35224 | Melatonin 5mg capsules |
| 5306 | Sonata 10mg capsules (Meda Pharmaceuticals Ltd) |
| 9814 | Nitrazepam 5mg/5ml oral suspension |
| 14250 | Melatonin 3mg tablets |
| 3741 | Stilnoct 10mg tablets (Sanofi) |
| 10068 | Melatonin 1mg capsules |
| 14145 | Melatonin 3mg capsules |
| 2950 | Noctec 500mg Capsule (E R Squibb and Sons Ltd) |
| 26391 | Melatonin 10mg capsules |
| 30377 | Zopiclone 3.75mg tablets (Generics (UK) Ltd) |
| 34777 | Zopiclone 3.75mg tablets (Teva UK Ltd) |
| 41696 | Zolpidem 5mg tablets (Teva UK Ltd) |
| 34692 | Lormetazepam 1mg tablets (Genus Pharmaceuticals Ltd) |
| 34806 | Nitrazepam 5mg tablets (Generics (UK) Ltd) |
| 41385 | Nitrazepam 5mg tablets (Actavis UK Ltd) |
| 3580 | Chloral hydrate 143.3mg/5ml oral solution BP |
| 58692 | Melatonin 3mg/5ml oral solution |
| 55860 | Melatonin 3mg modified-release capsules (Imported (United States)) |
| 2404 | Triazolam 250microgram Tablet |
| 45783 | Melatonin 5mg/5ml oral suspension |
| 61678 | Nitrazepam 2mg/5ml oral suspension |
| 3687 | Lormetazepam 500microgram tablets |
| 34534 | Lormetazepam 1mg Tablet (Wyeth Pharmaceuticals) |
| 46909 | Triazolam (roi) 125microgram Tablet |
| 29869 | Zolpidem 5mg Tablet (Winthrop Pharmaceuticals Ltd) |
| 34361 | Lormetazepam 500microgram tablets (Genus Pharmaceuticals Ltd) |
| 5459 | Zolpidem 10mg tablets |
| 32796 | Potassium bromide & chloral mixture |
| 23874 | Somnite 2.5mg/5ml oral suspension (Norgine Pharmaceuticals Ltd) |
| 7748 | Dichloralphenazone 650mg tablets |
| 8758 | Dichloralphenazone 225mg/5ml oral solution |
| 45367 | Bio-Melatonin 3mg tablets (Imported (Denmark)) |
| 38208 | Melatonin 2mg modified-release tablets |
| 8624 | Butobarbital 100mg tablets |
| 13447 | Soneryl 100mg tablets (Flynn Pharma Ltd) |
| 55774 | Bromazepam 3mg tablets |
| 28703 | Evacalm 5mg Tablet (Unimed Pharmaceuticals Ltd) |
| 2401 | Valium 2mg Tablet (Roche Products Ltd) |
| 4338 | Valium 5mg Tablet (Roche Products Ltd) |
| 34892 | Diazepam 5mg Tablet (Berk Pharmaceuticals Ltd) |
| 28347 | Diazepam 2mg Tablet (Crosspharma Ltd) |
| 33070 | Solis 5mg Capsule (Galen Ltd) |
| 34561 | Diazepam 2mg Tablet (Regent Laboratories Ltd) |
| 9045 | Diazepam 1mg/5ml suspension |
| 10402 | Valium 10mg Tablet (Roche Products Ltd) |
| 3205 | Diazepam 5mg |
| 30321 | Valium 2mg/5ml Oral solution (Roche Products Ltd) |
| 34876 | Diazepam 2mg Tablet (Berk Pharmaceuticals Ltd) |
| 45135 | Diazepam 2mg Tablet (M & A Pharmachem Ltd) |
| 34681 | Diazepam 5mg Tablet (Crosspharma Ltd) |
| 3870 | Diazepam 2mg capsules |
| 19299 | Valium 5mg Capsule (Roche Products Ltd) |
| 20164 | Valium 2mg Capsule (Roche Products Ltd) |
| 54695 | Diazepam 10mg Tablet (M & A Pharmachem Ltd) |
| 36581 | Atensine 10mg Tablet (Rorer Pharmaceuticals Ltd) |
| 9111 | Diazepam 10mg capsules |
| 3582 | Phenergan 25mg tablets (Sanofi) |
| 49968 | Phenergan 25mg tablets (DE Pharmaceuticals) |
| 14955 | Phenergan Nightime 25mg tablets (Sanofi) |
| 3494 | Promethazine hydrochloride 25mg tablets |
| 47056 | Promethazine 50mg/2ml solution for injection ampoules |
| 55838 | Phenergan 25mg tablets (Lexon (UK) Ltd) |
| 58361 | Clomethiazole 31.5mg/ml oral solution sugar free (A A H Pharmaceuticals Ltd) |
| 3491 | Heminevrin 192mg capsules (AstraZeneca UK Ltd) |
| 59170 | Clomethiazole 192mg capsules (A A H Pharmaceuticals Ltd) |
| 2535 | Clomethiazole 157.5mg/5ml oral solution sugar free |
| 563 | Clomethiazole 192mg capsules |
| 3081 | Atarax 10mg/5ml Oral solution (Pfizer Ltd) |
| 43446 | Hydroxyzine Oral suspension |
| 683 | Atarax 25mg Tablet (Pfizer Ltd) |
| 1913 | Atarax 10mg Tablet (Pfizer Ltd) |
| 40472 | Atarax 25mg tablets (Alliance Pharmaceuticals Ltd) |
| 5139 | Nytol one a night 50mg Tablet (Stafford-Miller Ltd) |
| 11004 | Promethazine hydrochloride 20mg tablets |
| 38104 | Nytol 25mg Tablet (GlaxoSmithKline Consumer Healthcare) |
| 2750 | Nytol 25mg Tablet (Stafford-Miller Ltd) |
| 23044 | Nytol 25mg Tablet (Stafford-Miller Ltd) |
| 14989 | Diphenydramine 50mg capsules |
| 1546 | Hydroxyzine 25mg tablets |
| 21359 | Sominex 20mg tablets (Actavis UK Ltd) |
| 21090 | Medinex 10mg/5ml Oral solution (Wyeth Consumer Healthcare) |
| 40510 | Atarax 10mg tablets (Alliance Pharmaceuticals Ltd) |
| 17784 | Ucerax 25mg tablets (UCB Pharma Ltd) |
| 878 | Hydroxyzine 10mg tablets |
| 40470 | Sleep aid 50mg Tablet (A A H Pharmaceuticals Ltd) |
| 35846 | Promethazine 25mg/1ml solution for injection ampoules |
| 1610 | Phenergan 5mg/5ml elixir (Sanofi) |
| 62590 | Promethazine 25mg/5ml oral suspension |
| 918 | Phenergan 10mg tablets (Sanofi) |
| 35437 | Phenergan 25mg/1ml solution for injection ampoules (Sanofi) |
| 5561 | Promethazine hydrochloride 10mg tablets |
| 1998 | Promethazine 5mg/5ml oral solution sugar free |
| 31889 | Ziz 10mg tablets (Chatfield Laboratories) |
| 8464 | Meprobamate with bendroflumethiazide Tablet |
| 8303 | Tenavoid Tablet (Edwin Burgess Ltd) |

1. Thomas KH, Martin RM, Davies NM, Metcalfe C, Windmeijer F, Gunnell D. Smoking cessation treatment and risk of depression, suicide, and self harm in the Clinical Practice Research Datalink: prospective cohort study. BMJ: British Medical Journal. 2013;347:f5704.
